# Emotional Responses to Visual Art and Commercial Stimuli: Implications for Creativity and Aesthetics

**DOI:** 10.3389/fpsyg.2019.00014

**Published:** 2019-01-22

**Authors:** Mei-Chun Cheung, Derry Law, Joanne Yip, Christina W. Y. Wong

**Affiliations:** ^1^Department of Social Work, The Chinese University of Hong Kong, Shatin, Hong Kong; ^2^Institute of Textiles and Clothing, The Hong Kong Polytechnic University, Hung Hom, Hong Kong

**Keywords:** emotional responses, window displays, visual art, aesthetics, creativity, EEG

## Abstract

There is an ongoing debate about whether emotional responses to artworks are similar to those produced by the commercial stimuli experienced in everyday life. In this study, we evaluated the emotional responses to the visual art and commercial stimuli by using electroencephalography (EEG) to obtain an objective measure of emotional responses of the brain, namely the frontal alpha asymmetry. Positive frontal alpha asymmetry suggests positive emotional responses, and vice versa. The visual art stimuli consisted of 80 artistic and naturally colored paintings whereas the commercial stimuli consisted of 80 different window displays of fashion collections. The results revealed that positive frontal alpha asymmetry was elicited when the participants judged the visual art stimuli as either beautiful or not beautiful. For the commercial stimuli, positive frontal alpha asymmetry was observed when they were considered as beautiful, whereas negative frontal alpha asymmetry was exhibited toward those perceived as not beautiful. These findings suggest more positive emotional responses to the visual art stimuli, regardless of their aesthetics. However, favorable emotional responses were only elicited toward the commercial stimuli regarded as beautiful. The implications for the creative and aesthetic design of the commercial stimuli in Chinese society in influencing consumers’ emotional responses are discussed.

## Introduction

The visual arts, such as painting and sculpture, are the most prevalent forms of visual artistic expression in the West that are considered to be creative products (Kaufman et al., 2008; Lan and Kaufman, 2013). In addition, the visual arts are often associated with aesthetics; hence investigations into the neural processing involved in art appreciation has rapidly evolved as an objective and scientific approach to understanding aesthetics (Zaidel, 2010; Shimamura and Palmer, 2012; Chatterjee, 2014). Researchers have used the visual arts to identify the neural correlates of aesthetic appreciation (Vartanian and Goel, 2004; Lengger et al., 2007; Cela-Conde et al., 2009, 2013; Cupchik et al., 2009; Munar et al., 2012; Vessel et al., 2012; Ishizu and Zeki, 2013; Pang et al., 2013; Boccia et al., 2014; Gerger et al., 2014). By using magnetoencephalography and functional magnetic resonance imaging (fMRI), widely distributed brain regions have been identified as being associated with aesthetic appreciation of the visual arts; these regions overlap with the functionally connected brain networks involved in reward representation, affective motor planning, attention-related sensory processing, and evaluative judgments of social and moral cues (Leder and Nadal, 2014). These brain regions include the medial orbitofrontal cortex (Kawabata and Zeki, 2004; Ishizu and Zeki, 2011, 2013; Cela-Conde et al., 2013; Zeki et al., 2014); the fronto-median cortex (Jacobsen et al., 2006; Jacobs et al., 2012); the prefrontal cortex (Jacobsen et al., 2006); the posterior cingulate gyrus (Jacobsen et al., 2006; Jacobs et al., 2012); the left temporal pole (Jacobsen et al., 2006); the temporoparietal junction (Jacobsen et al., 2006); the left angular gyrus (Zeki et al., 2014); the left superior temporal gyrus (Zeki et al., 2014); and the amygdala (Jacobs et al., 2012). When stimuli are perceived to be beautiful, rather than ugly, additional brain regions are recruited in the orbitofrontal cortex (Jacobsen et al., 2006; Tsukiura and Cabeza, 2011; Zeki et al., 2014), the right amygdala (Di Dio et al., 2007), and the secondary visual cortex (Jacobs et al., 2012). Moreover, a delayed brain network is more synchronized during aesthetic judgment of beautiful stimuli compared with those that are not considered beautiful. The network consists of the medial occipital, the lateral occipital, the lateral posterior parietal, the medial parietal, the medial frontal, and the prefrontal regions of the left hemisphere (Cela-Conde et al., 2013).

Visual stimuli that have received academic attention include sculptures (Di Dio et al., 2007); faces (Roye et al., 2008; Chatterjee et al., 2009; Tsukiura and Cabeza, 2011; Zhang and Deng, 2012); textures (Jacobs et al., 2012); geometrical shapes (Jacobsen and Höfel, 2001, 2003; Jacobsen et al., 2006; Höfel and Jacobsen, 2007; de Tommaso et al., 2008); and even mathematical formulae (Zeki et al., 2014). However, despite extensive studies using a variety of different visual stimuli, few examine the neural processing of aesthetic experiences using the commercial stimuli. Using fMRI, significantly stronger brain activation in specific affective areas were found during aesthetic product presentation; those areas include the ventromedial prefrontal cortex, the nucleus accumbens, and the cingulate cortex (Reimann et al., 2010; Jiang and Cai, 2013). In addition, the commercial products with aesthetic packaging significantly induced an increase in the reaction time of the consumers’ choice responses (Reimann et al., 2010). The difference in the response time between non-aesthetic and aesthetic commercial products was also revealed in an event-related brain potential (ERP) study by Jiang and Cai (2013), in which less beautiful color combinations elicited higher P2 and P300 amplitudes than more beautiful ones. The results suggested that the negative emotional responses aroused by the less beautiful color were elicited at an early stage. It was also suggested, therefore, that aesthetic experiences with beautiful stimuli, regardless of whether these are artistic or commercial stimuli, involve a delayed dynamic and functionally integrated brain network, requiring a longer reaction time to make a judgment compared with that induced by stimuli that are not beautiful. In our previous study (Cheung et al., 2014), we demonstrated brain activities suggestive of an integrative process involved in attention with central executive processing, and more positive emotional responses when the personal-appearance styles were judged to be beautiful. In addition, aesthetic judgment engaged a delayed synchronized brain network, involving long-range coherence between the frontal and parietal regions in both hemispheres, and coherence between the two hemispheres in the frontal and central regions.

Unlike Western society which views a creative product as merely a creation, creative products in Chinese society can be regarded as the outcome of a creative industry (Wang et al., 2007), and as saleable goods that are associated with economic activities (Lan and Kaufman, 2013). Therefore, the present study uses fashion window displays as the commercial stimuli, as they combine both aesthetics and creativity (Pieters et al., 2010; Law et al., 2013), and are considered as creative products in China (Lan and Kaufman, 2013). Apart from increasing the attractiveness of the displayed products to draw the attention of customers, window displays differ from simple merchandise-focused displays that convey implicit messages (Diamond and Diamond, 2007). This is because customers need to rely on their own interpretation of the inferred message projected from the window displays for the overall feeling and perception of the products, store and brand, thereby determining the subsequent decision to shop; that is, whether they will enter the store (Sen et al., 2002; Oh and Petrie, 2012). Unlike artworks that mainly fulfill a hedonic need, window displays provide utilitarian motives, which induce approach-motivated reactions and goal-oriented behavior. Therefore, a comparison of the neural processing of aesthetic experiences and emotional responses to the visual arts and commercial stimuli can address the research gap in differentiating the elicited emotional responses from the art and aesthetics of everyday life by objective and scientific measures of neural activity in the brain, therefore substantiating theoretical knowledge of aesthetics and creative products in Western and Chinese culture. To the best of our knowledge, very few studies have been carried out that provide insight into the emotional responses to window displays.

Different emotions are associated with different EEG patterns in the frontal regions of the brain (Ekman et al., 1990; Fox, 1991). One of the well-documented measures of emotional responses is the frontal alpha asymmetry (see reviews by Coan and Allen, 2004; Harmon-Jones et al., 2010). This is calculated by subtracting the log-transformed absolute alpha power of the left frontal region from the analogous log-transformed alpha power of the right frontal region; i.e., log right minus log left (Davidson et al., 2000b). This asymmetry is proposed by Davidson (1998), who conducted a series of EEG studies using this asymmetry to reflect human mood states, and repeatedly identified a positive association between greater relative left-side activation in the anterior part of the frontal region and positive mood. Specifically, positive emotion is associated with greater relative left-sided frontal activation, as compared to right-sided (Davidson, 2000; Davidson et al., 2000a, 2003), whereas negative emotion is accompanied by greater relative right-sided frontal activation, as compared to left-sided (Davidson et al., 1990, 2000b; Davidson, 1992). As alpha power is inversely associated with brain activation, positive frontal alpha asymmetry that denotes greater alpha power on the right and less alpha power on the left suggests greater relative left-sided activation – that is, positive emotion. In contrast, negative frontal alpha asymmetry represents greater relative activation on the right, suggesting negative emotion. Our empirical and clinical studies also demonstrated that the frontal alpha asymmetry is effective and reliable in discriminating between positive and negative emotions (Chan et al., 2008, 2011; Cheung et al., 2014).

The current literature has proposed that emotions produced from viewing the arts are different from those experienced in everyday life (Frijda, 1988; Frijda and Schram, 1995; Scherer, 2005). For instance, Frijda’s emotion theory concluded that appraisal of artworks is not relevant to the current needs and goals of individuals (Frijda, 1988). Hence laypeople are psychologically removed (Cupchik, 2002), which results in less intense emotional responses. Motivational and goal-oriented reactions might also be reduced. However, Levinson (1996); Goldstein (2009), and others have argued that perceivers can indulge in their aesthetic experiences with art with comparable or even more intense emotional responses than those experienced in everyday life, as artworks are usually viewed in a safe and relaxed environment. Given that there is an ongoing debate about whether emotional responses to artworks are similar to those elicited by aesthetic objects found in everyday life, in this study we compare the neural processing of emotional responses to aesthetic experiences using two creative products: paintings versus fashion window displays. In particular, we use the frontal alpha asymmetry to investigate whether the brain is involved in a similar way in the emotional responses to the visual art and commercial stimuli that are regarded as beautiful and not beautiful.

## Materials and Methods

### Participants

Twenty university students (4 males, 16 females) with specialism in design or business were recruited from the Institute of Textiles and Clothing at The Hong Kong Polytechnic University and participated in the study voluntarily. The participants had a mean age of 21.15 years (SD ± 1.18), had spent 15 years in full-time education, and reported a negative history of neurological and psychiatric problems. They completed some fundamental courses in which their creativity and design skills were nurtured and had basic knowledge related to fashion business. Given their academic background, they were competent to make aesthetic judgments of visual art and commercial stimuli. The study followed the research guidelines of the Helsinki Declaration of the World Medical Association Assembly, and the research protocol was approved by the Human Subjects Ethics Sub-committee (HSESC) of The Hong Kong Polytechnic University. Informed consent was obtained from all participants prior to the study in accordance with institutional guidelines.

### Selection of Visual Stimuli

The visual art stimuli consisted of 80 artistic and naturally colored Western paintings. They downloaded from the classification of paintings in the ARTStor Digital Library^1^, which consists of more than one million images from 100s of different collections. Based on the painting styles, such as visual elements, movement or school, these stimuli were categorized into four types of painting with twenty in each group, as follows: (i) impressionist art; (ii) post-impressionist art; (iii) abstract art; and (iv) surrealist art. Of the 20 paintings in each group, 10 were considered to be beautiful and 10 were considered to be not beautiful, according to the criteria below. The commercial stimuli consisted of 80 different window displays, of which 40 were considered to be beautiful and 40 to be not beautiful. They were taken from a collection of fashion displays used in teaching and research (Law et al., 2012). The displays consisted of different clothing categories from classic wear to fashionable casual wear and mass market items. They were also representative of different types of customer segments, from the High Street shopper to those with more affluent tastes, and were thus an effective stimulus for measuring aesthetic-related issues. To emphasize the relevance of the commercial stimuli on the needs and goals of the viewer, mannequins were used in the fashion window displays because they are an important element for retailers in projecting a three-dimensional space in terms of fit and style when dressing a physical body (Oh and Petrie, 2012). In addition, mannequins provide non-verbal cues which attract attention more easily (Palermo and Rhodes, 2007) and can stimulate mental visualizations of wearing the displayed clothing (Elder and Krishna, 2012). Mannequins are therefore an important visual element for consumers (Kerfoot et al., 2003). The stimuli of paintings and fashion window displays can be found in the Supplementary Material.

Prior to the EEG recording, a pilot pool of 160 paintings (40 from each of the four categories) and 160 fashion window displays were initially chosen by an independent expert with arts background who was blind to the experiment. These stimuli were printed on sheets of A4 paper and were given to 20 anonymous university students who were not involved in the study, to rate whether they were perceived as beautiful or not beautiful. The most beautiful and “absolutely not” beautiful stimuli were chosen for the experiment, based on the highest consensus from this pilot pool of paintings and fashion window displays.

### EEG Recording

The EEG signals were collected from 64 silver/silver chloride (Ag/AgCl) sintered electrodes mounted on a stretch Lycra Quik-Cap (Compumedics Neuroscan, El Paso, TX, United States). The electrode placement was arranged in accordance with the international 10–10 system (Chatrian et al., 1985; American Electroencephalographic Society, 1994). A ground electrode was located on the forehead anterior to the Fz electrode and the linked-ears reference was adopted. Eye movements and blinking were recorded by vertical electrooculograms from the electrodes on the supraorbital and suborbital regions of the left eye, and horizontal electrooculograms from the electrodes on the outer canthi of both eyes. The impedance was less than 10 kΩ and homologous sites were within 1 kΩ of each other. A Neuroscan SynAmps2 amplifier unit (El Paso, TX, United States) with a bandpass filter of 0.05 to 200 Hz and a sampling rate of 1000 Hz was utilized to amplify the EEG signals.

### EEG Paradigm

**FIGURE 1:**
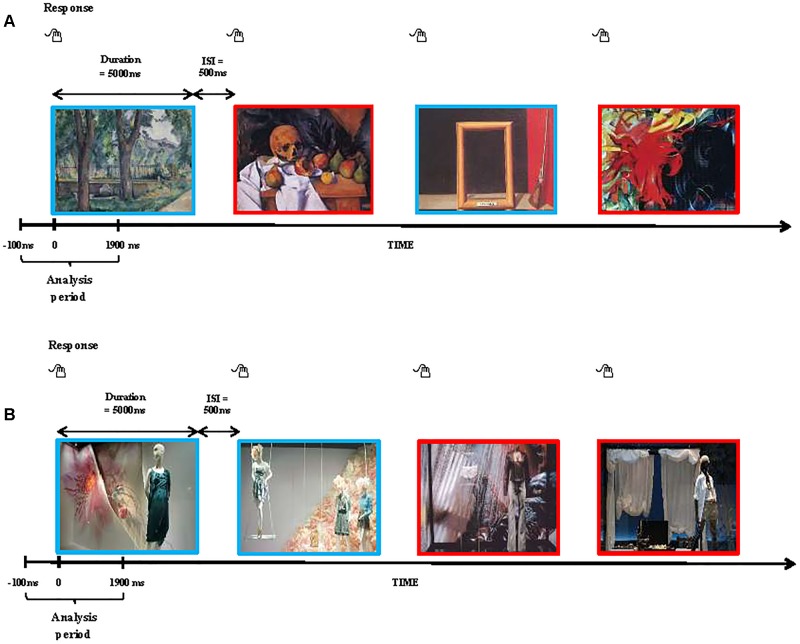
Experimental paradigm of visual stimulus. **(A)** Random presentation of paintings with 20 visual stimuli (10 beautiful and 10 not beautiful) in each of the four categories: (i) impressionist art; (ii) post-impressionist art; (iii) abstract art; and (iv) surrealist art. **(B)** Random presentation of fashion window displays (40 beautiful and 40 not beautiful). Participants were asked if they perceived the visual stimuli as either “beautiful” or “not beautiful” and responded by pressing a STIM2 Response Pad. Red rectangles indicate examples of visual stimuli perceived to be not beautiful and blue rectangles indicate examples of those perceived to be beautiful.

The EEG paradigm included one 4-min resting condition with eyes open as the baseline, and two experimental conditions (aesthetic judgment of paintings vs. fashion window displays). The sequence of the two experimental conditions was randomized and counterbalanced among the 20 participants. During the EEG recording, the participants performed aesthetic judgments of the 80 paintings and 80 fashion window displays, which were presented for 5 s with an inter-trial interval of 5.5 s. The visual stimuli were generated, controlled, and presented using software called STIM2 (Compumedics Neuroscan, El Paso, TX, United States). The participants were invited to assess the perceived visual stimuli as either “beautiful” or “not beautiful” by pressing the corresponding buttons of the STIM2 Response Pad. The stimuli of paintings and fashion windows displays were randomly presented (Figure 1). Besides EEG signals, the behavioral measures included the time taken by the participants to make aesthetic judgments and the number of visual stimuli perceived as beautiful or not beautiful. After two trials of the experimental conditions, the participants fully relaxed with eyes open for 4 min as the baseline measurement.

### EEG Processing

The EEG signals of the experimental conditions were imported into the EEGLAB software program to extract the EEG epochs that corresponded to the visual stimuli perceived as beautiful or not beautiful, according to aesthetic judgments of the participants. The epochs were spanned at intervals from 100 ms pre-stimulus to 1900 ms post-stimulus. The epochs of the visual stimuli and the EEG signals for the 4-min resting state were processed by the NeuroGuide (v2.7.9) software program to remove artifacts. Individual EEG files were first visually inspected for any gross abnormalities by a trained technician who was blind to the purpose of the study. Using an EEG template that was artifact-free for at least 10 s, the data were edited by the built-in automated artifact detection and rejection toolbox to remove all non-EEG artifacts, including eye movement and blink artifacts and drowsiness. Split-half and test–retest reliability tests were performed on the selected EEG segments with built-in tools in the NeuroGuide software. For the subsequent spectral analysis of fast Fourier transformation (FFT), we chose only those segments with minimum split-half and test–retest reliability ratios of 90%, and with at least 1 min in the resting state or at least 25 artifact-free epochs for “beautiful” and “not beautiful.” In the FFT, the EEG data from each channel were transformed into predefined frequency ranges.

The EEG data for the resting condition and the epochs for the visual stimuli were analyzed over 64 electrode placements in the alpha frequency band (8 to 12 Hz) to calculate the frontal alpha asymmetry (Chan et al., 2008, 2011; Cheung et al., 2014) by subtracting the log-transformed absolute alpha power of the left frontal region (F3) from the analogous log-transformed alpha power of the right frontal region (F4); i.e., log F4 minus log F3. Based on Davidson’s findings (Davidson, 1992, 1998, 2000; Davidson et al., 1990, 2000a,b, 2003), which associate the frontal alpha asymmetry with emotional responses, the frontal alpha asymmetry index was used in this study to compare the emotional responses between the baseline (resting with eyes open) and the experimental conditions (aesthetic judgment of paintings or fashion window displays). A positive asymmetry index – that is, higher alpha power on the right frontal region of the brain (F4) and lower alpha power on the left frontal region (F3) – represents a more positive emotional response. Conversely, a negative asymmetry index represents a more negative emotional response.

### Statistical Data Analysis

The statistical data analysis was carried out using SPSS Version 21.0 for Windows (SPSS, Inc., Chicago, IL, United States). The normal distribution of the data was confirmed by Kolmogorov–Smirnov tests and parametric statistics were used for comparisons. Comparisons of the EEG data during resting condition with EEG epochs for the “beautiful” and “not beautiful” stimuli were conducted through paired-sample *t*-tests with 19 degrees of freedom. Repeated measures analysis of variance (ANOVA) with two within-subject factors was conducted to compare the frontal alpha asymmetry across conditions and stimuli to detect any overall differences between related means [(Condition: beautiful vs. not beautiful) × (Stimuli: paintings vs. fashion window displays)]. To indicate the effect sizes, partial eta-squared ($\eta_{p}^{2}$) values were also reported for ANOVA tests. For significant factors from the ANOVAs, further *post hoc* comparisons were made using paired-sample *t*-tests with 19 degrees of freedom.

## Results

### Behavioral Performance

The participants endorsed 98.5% (or 78.8) of the 80 paintings as either beautiful or not beautiful. The remaining 1.5% (or 1.2) did not receive a response. Of the 80 fashion window displays, 97.9% (or 78.3) were endorsed as either beautiful or not beautiful, while 2.1% (or 1.7) did not receive a response. Table 1 shows the mean judgment latency (calculated only for the trials that had responses), number of visual stimuli perceived as beautiful and not beautiful, and their standard deviations. There were no significant differences between the judgment latency and number of paintings and fashion window displays perceived as beautiful or not beautiful (*p* > 0.05).

**Table 1:** Number of visual stimuli perceived to be beautiful or not beautiful and mean judgment latency using EEG experimental paradigm.

	Beautiful	Not beautiful	*t*-Test	*p*-Value	Cohen’s *d*
Paintings	38.45 ± 9.49	40.35 ± 9.75	−0.443	0.663	0.099
Fashion window displays	38.10 ± 13.89	40.25 ± 14.37	−0.344	0.735	0.015
Response time to paintings (ms)	1498.81 ± 364.98	1493.23 ± 423.06	0.068	0.947	0.077
Response time to fashion window displays (ms)	1573.71 ± 348.95	1561.83 ± 358.51	0.159	0.876	0.035

### Frontal Alpha Asymmetry

#### Visual Stimuli Versus Resting

The paired-sample *t*-tests revealed a positive frontal alpha asymmetry when the paintings were perceived to be beautiful (*M* = 0.048), in comparison with the resting condition (*M* = −0.003; *t* = 2.325, *p* = 0.031). Negative frontal alpha asymmetry was found when the fashion window displays were judged to be not beautiful (*M* = −0.067; *t* = −2.967, *p* = 0.008). There was no significant difference in the frontal alpha asymmetry between the resting condition and paintings perceived to be not beautiful, or between the resting condition and fashion window displays perceived to be beautiful.

#### Visual Art Versus Commercial Stimuli

A repeated measures ANOVA with two within-subject factors was conducted to compare the frontal alpha asymmetry [(Stimuli: paintings vs. fashion window displays) × (Condition: beautiful vs. not beautiful)]. The main effects of Stimuli [*F*(1,19) = 33.402, *p* = 0.000, $\eta_{p}^{2}$ = 0.637] and Condition [*F*(1,19) = 16.305, *p* = 0.001, $\eta_{p}^{2}$ = 0.462] and the interaction effect of Stimuli and Condition [*F*(1,19) = 5.113, *p* = 0.036, $\eta_{p}^{2}$ = 0.212] were all significant, which means that there were significant and condition-specific differences in the frontal alpha asymmetry between the paintings and fashion window displays. Subsequent *post hoc* pairwise comparisons showed that the frontal alpha asymmetry induced by the paintings (*M* = 0.043) was generally more positive and significantly higher than that induced by the fashion window displays (*M* = −0.013, with *p* = 0.000). In addition, the frontal alpha asymmetry induced by visual stimuli that were perceived to be beautiful (*M* = 0.044) was also more positive and significantly higher than that induced by visual stimuli that were perceived to be not beautiful (*M* = −0.015, with *p* < 0.001).

**FIGURE 2:**
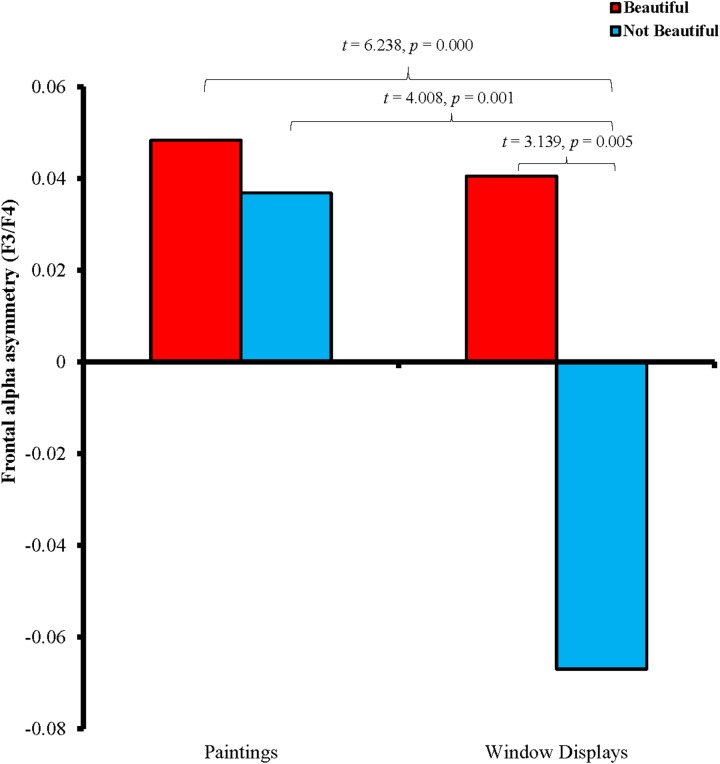
Frontal alpha asymmetry (log F4 minus log F3) of visual stimuli perceived to be beautiful or not beautiful. More positive frontal alpha asymmetry value was observed when participants perceived paintings and fashion window displays to be beautiful or paintings to be not beautiful, and more negative frontal alpha asymmetry value was exhibited when fashion window displays were perceived to be not beautiful.

As shown by the *post hoc* paired-sample *t*-test (Figure 2), there was no significant difference in the frontal alpha asymmetry between the paintings that were perceived to be beautiful (*M* = 0.048) and not beautiful (*M* = 0.037; *t* = 0.912, *p* = 0.373). However, the frontal alpha asymmetry of the fashion window displays that were judged to be beautiful (*M* = 0.041) was generally more positive than that of the fashion window displays that were perceived to be not beautiful (*M* = −0.067; *t* = 3.139, *p* = 0.005). Comparison between the paintings and fashion window displays indicated that there was no significant difference in the frontal alpha asymmetry between the paintings (*M* = 0.048) and fashion window displays (*M* = 0.041) that were perceived to be beautiful (*t* = 0.387, *p* = 0.703). However, the frontal alpha asymmetry of the fashion window displays that were perceived to be not beautiful (*M* = −0.067) was significantly lower than that of the paintings that were perceived to be beautiful (*M* = 0.048; *t* = 6.238, *p* = 0.000) and not beautiful (*M* = 0.037; *t* = 4.008, *p* = 0.001).

## Discussion

Aesthetic emotions, as modeled by both Chatterjee (2004) and Leder et al. (2004), are one of the outputs of aesthetic experiences. Using fMRI, several brain regions have been identified as being related to aesthetic emotions, including the temporal pole (Jacobsen et al., 2006), the bilateral insular cortex (Cupchik et al., 2009), the orbitofrontal cortex (Cela-Conde et al., 2004; Kawabata and Zeki, 2004), the caudate nucleus, and the anterior cingulate cortex (Vartanian and Goel, 2004). These results suggest that positive aesthetic experiences involve affective processes related to the reward value of the aesthetically judged stimuli. More functional activity has also been observed in the right amygdala, which has the primary role of processing emotional responses when images are perceived to be beautiful, compared to images that are regarded to be not beautiful (Di Dio et al., 2007). These fMRI studies focus on localizing the brain regions during aesthetic experiences, but cannot determine whether pleasurable emotional responses are elicited during aesthetic experiences. Given this limitation, we recently carried out a study that used the frontal alpha asymmetry as an index of mood states to examine whether aesthetic judgment of personal-appearance styles as beautiful resulted in a pleasurable experience for the perceiver (Cheung et al., 2014). We found that, compared to the resting condition, positive frontal alpha asymmetry, which points to a positive emotional response, was observed when the participants perceived the personal-appearance styles to be beautiful. On the other hand, negative frontal alpha asymmetry, which points to a negative emotional response, was found when the personal-appearance styles were regarded as not beautiful. As an extension to previous studies, the present study investigated and compared the emotional responses to the aesthetic experiences of the visual art stimuli (paintings) versus commercial stimuli found in everyday life (fashion window displays) to determine if emotional responses to the former were similar to those elicited by the latter. Using paintings as the visual art stimuli, the findings demonstrated that regardless of whether they were perceived to be beautiful, we found positive frontal alpha asymmetry, and no significant difference in the frontal alpha asymmetry between them. Compared to the resting condition, the frontal alpha asymmetry was significantly higher and more positive when the paintings were considered to be beautiful. Changes in the frontal alpha asymmetry are closely related to affective-state manipulation. Studies indicate that environmental stimuli triggering positive or approach-related emotions (such as joy) result in greater relative left frontal activation (more positive frontal alpha asymmetry) than environmental stimuli encouraging negative or withdrawal-related emotions (such as disgust, fear, and sadness). The latter emotions produce greater relative right frontal activation (more negative frontal alpha symmetry) (Davidson and Fox, 1982; Ekman et al., 1990; Coan et al., 2001). Therefore, positive affective responses or approach-related emotions were produced when the participants viewed the paintings. Moreover, aesthetic judgments of the visual art stimuli as beautiful or not beautiful produced similar emotional responses. Indeed, the findings of the positive emotional responses toward artworks negatively perceived are consistent with the findings of Gerger et al. (2014), who found that in an art context, negative stimuli are viewed more positively. Therefore, aesthetic experiences of creative products in an art context, such as paintings, facilitate more pleasurable emotional responses in the perceiver, and aesthetics does not seem to play a significant role in determining the emotional response. Even though the visual stimuli are perceived to be not beautiful, positive or approach-related emotions are still produced if the visual stimuli are viewed in an art context.

As compared to the paintings that are considered to be creative products in the West (Kaufman et al., 2008; Lan and Kaufman, 2013), the emotional response to the aesthetic experience of fashion window displays, which are considered to be products of the creative industry in China, was similar only when the visual stimuli were perceived to be beautiful. Positive frontal alpha asymmetry was found when the fashion window displays were perceived to be beautiful. Therefore, the results suggest that emotional responses to the visual art stimuli were similar to those to the commercial stimuli regarded as creative products in Chinese society only when the latter were considered to be beautiful. These findings are not consistent with those of Gerger et al. (2014), who found a slightly less positive reaction toward positive stimuli in the art context compared to a non-art context, thus suggesting distanced processing of the former (Cupchik, 2002). One possible reason for the discrepancy might be the perceived relevance during aesthetic judgments. In our study, the participants judged the visual art stimuli purely in term of aesthetics (i.e., beautiful versus not beautiful) and without subjective preferences, whereas the participants in Gerger et al. (2014) were asked to determine how much they liked the stimuli, thus incorporating their personal preferences and affecting the psychological distance toward the stimuli. Therefore, the intensity of the emotional response toward stimuli during aesthetic experiences may differ if both subjective judgments of likeableness and aesthetic judgments are taken into consideration, because the intensity of emotional responses toward aesthetics may not necessarily be equivalent to those involved in likeableness. Whether aesthetic responses and common emotions are mediated by a similar mechanism has not yet been resolved (Leder and Nadal, 2014). Given that subjective preference was not controlled in the present study, it would be worthwhile to investigate and compare the emotional responses during aesthetic judgments and subjective preferences.

In contrast to Gerger et al. (2014) findings, the present study found a significant difference in the frontal alpha asymmetry in judging fashion window displays that were not perceived to be beautiful, as compared to the paintings as well as fashion window displays that were considered to be beautiful. A more negative frontal alpha asymmetry was found when the fashion window displays were not perceived to be beautiful. This is consistent with the findings of our previous study (Cheung et al., 2014), in which a negative frontal alpha asymmetry was found when personal-appearance styles were judged to be not beautiful, thus suggesting a negative emotional state. The intention or behavior of the participants following the observed change in emotional state was not investigated in this or the previous study. Nevertheless, emotional responses are likely to elicit corresponding behaviors; that is, “coping” or “motivated” (approach or withdrawal) behavior (Harmon-Jones and Sigelman, 2001; Harmon-Jones et al., 2003). Therefore, if fashion window displays are perceived to be not beautiful, the perceiver will experience a negative or withdrawal-related emotional response, which therefore substantially affects consumer behavior, such as alienating them from entering the premises. If such displays are perceived as beautiful by a customer, they may trigger a positive or approach-related emotional response that would instead motivate the customer, such as facilitating their entry into a store and making a purchase. Therefore, our findings highlight the significance of aesthetically pleasing fashion displays in which both creativity and aesthetics play important roles in encouraging favorable emotional responses. Unlike the creative products in an art context in the West, products from the creative industry in Chinese society, like fashion, largely rely on aesthetics to elicit a more pleasurable emotional response in the perceiver. Given that the visual art stimuli can produce a more positive emotional response, incorporating art elements into fashion displays may facilitate more prominent positive or approach-related emotional responses.

One of the limitations of this study is the laboratory setting in investigating the aesthetic experiences of paintings and fashion window displays. There is growing evidence that there are differences between the processes that underlie aesthetic experiences in the laboratory and in real-life contexts. For instance, Brieber et al. (2014) compared aesthetic experiences in a museum and laboratory and found that the participants preferred the artworks, found them more interesting, and viewed them longer in a museum as opposed to a laboratory. Context is therefore an important modulating factor in the aesthetic experience of artworks and commercial stimuli. It would be worthwhile therefore to examine emotional responses in real-life contexts and compare them with laboratory results to gain a better idea of the differences in emotional responses. In addition, most of the fashion window displays used as visual stimuli in this study were new to the participants, whereas some of the paintings might have been more familiar as they could have encountered them during museum visits. Several studies have investigated the relationship between novelty and aesthetic experience in product design (e.g., Hekkert et al., 2003; Blijlevens et al., 2012; Hung and Chen, 2012). When the relationship between novelty and aesthetic preferences toward environmental stimuli is plotted, the result is an inverted U-shaped curve, which means that moderate levels of novelty are associated with the stimuli that are perceived to be the most beautiful. Therefore, the intensity of emotional responses to novel versus familiar stimuli may differ in art and non-art contexts and should be controlled in future experiments.

As in our previous study (Cheung et al., 2014), the present study found no significant difference in response time between “beautiful” and “not beautiful” judgments. Some studies have suggested early impression formation of aesthetic evaluation of the “not beautiful” pattern (Höfel and Jacobsen, 2007; Roye et al., 2008; Jiang and Cai, 2013), whereas larger decision times were found to be involved in aesthetic judgment of beautiful objects (Zhang and Deng, 2012). The discrepancy may be due to differences in the recording process. Electrophysiological responses revealed in ERP were utilized in these previous studies to directly record brain activity, whereas our study used a behavioral approach (pressing the response pad) to record the response time, thus resulting in less precision.

Finally, another limitation is related to uneven gender distribution, as only four males were recruited for the present study. Recent studies have demonstrated gender differences in the neural underpinning of aesthetic appreciation of environmental stimuli, such as attractive faces (Zhang and Deng, 2012), physical bodies (Cazzato et al., 2014), and artistic paintings or natural objects (Cela-Conde et al., 2009). In addition, females generally enjoy shopping to a greater extent than males, so fashion displays may arouse a more intense emotional response, both positively and negatively, than artworks. Therefore, as in our previous study (Cheung et al., 2014), a biased gender distribution in the present study (16 of the 20 participants were female) may have had a differential impact on emotional responses when the participants viewed the paintings and fashion displays.

## Author Contributions

M-CC, DL, and CW: conceptualization. M-CC and DL: methodology. M-CC: data analysis. M-CC, DL, CW, and JY: contribution of reagents, materials, and analysis tools. M-CC, DL, CW, and JY: manuscript preparation and revision. All authors have approved the work for publication.

## Conflict of Interest Statement

The authors declare that the research was conducted in the absence of any commercial or financial relationships that could be construed as a potential conflict of interest.

## Footnotes

1 www.artstor.com
