# Supplementary material for: Emotional Responses to Visual Art and Commercial Stimuli: Implications for Creativity and Aesthetics
Source: Front Psychol. 2019 Jan 22;10:14. doi: 10.3389/fpsyg.2019.00014 (PMC6349741; doi:10.3389/fpsyg.2019.00014)

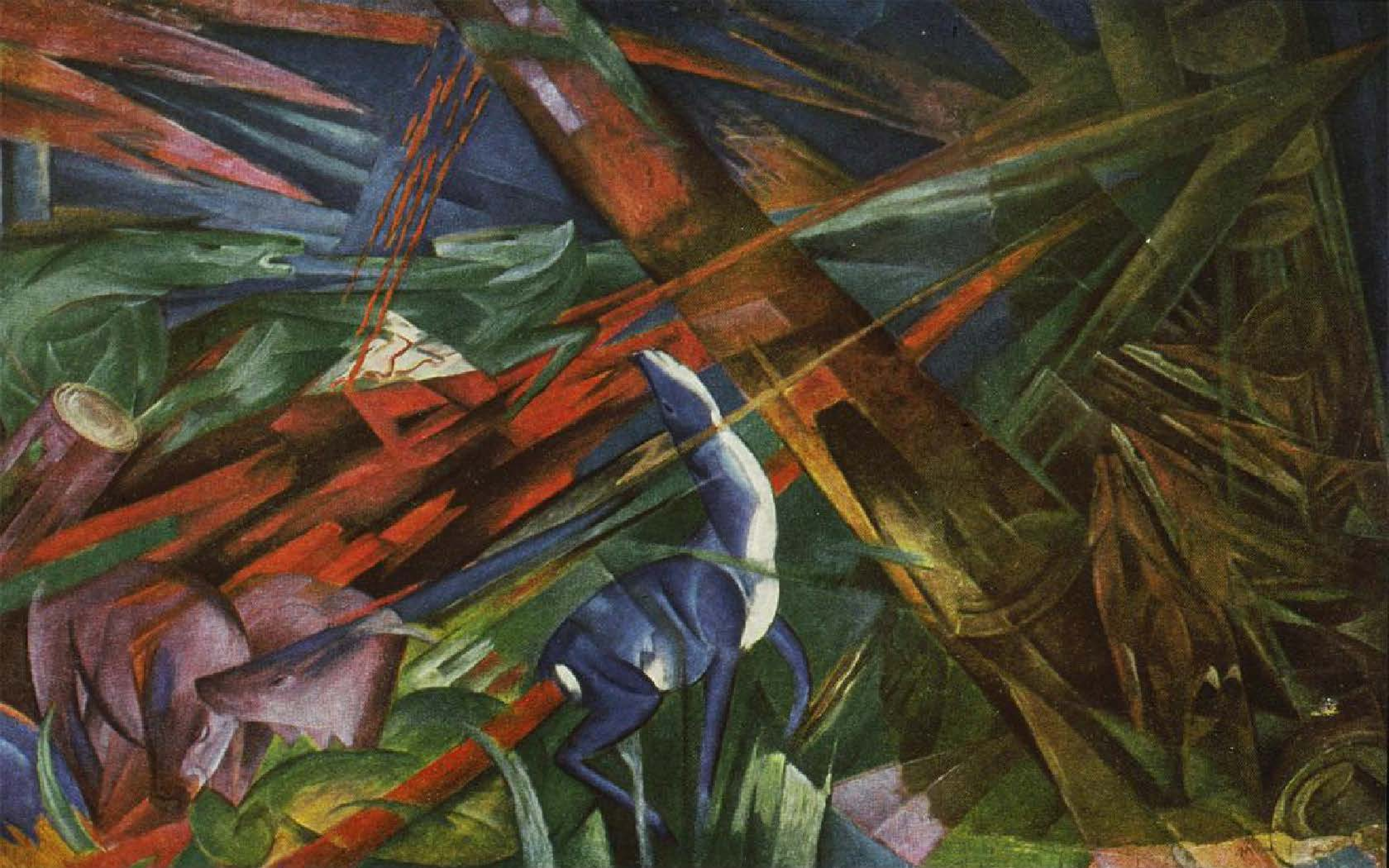

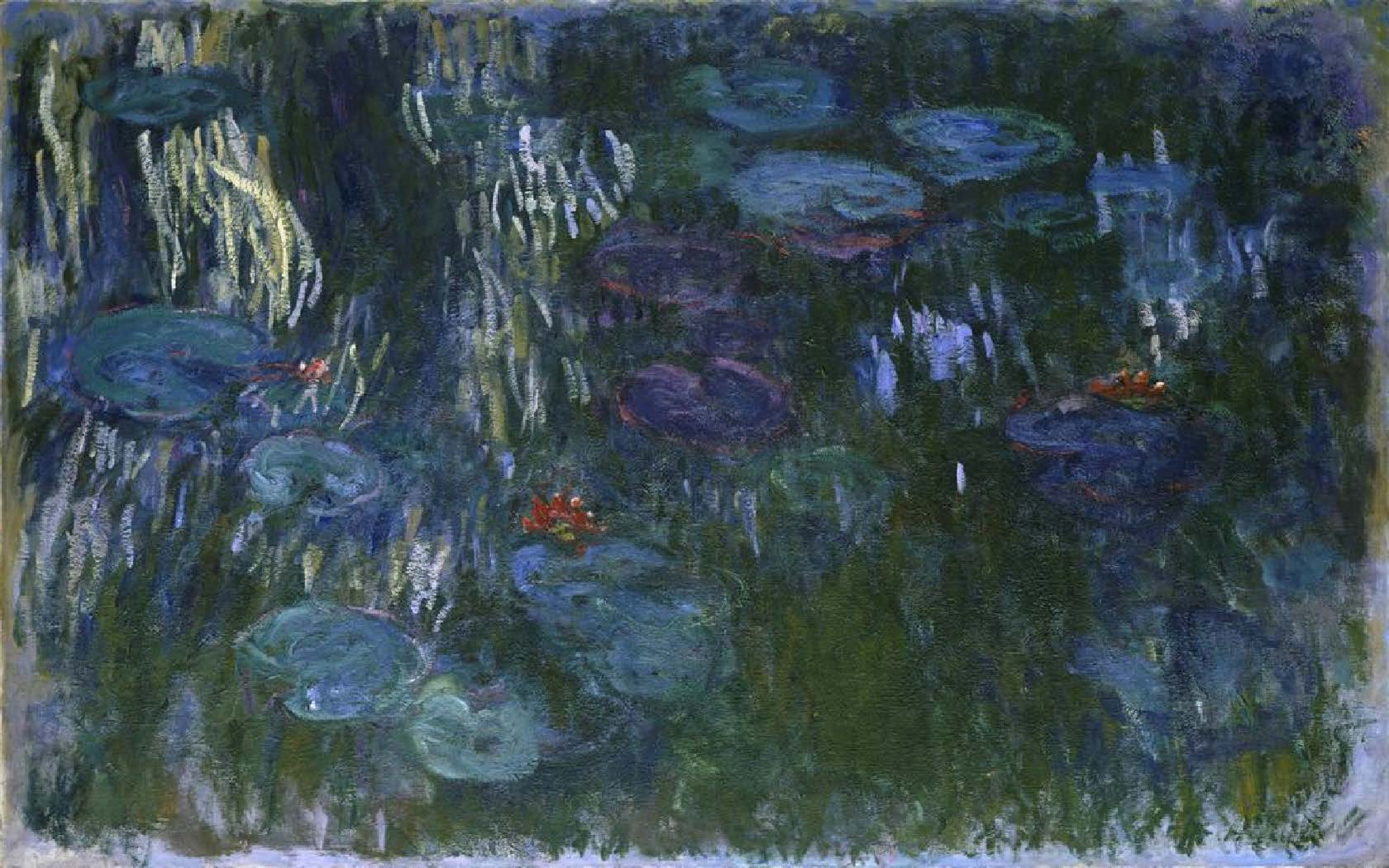

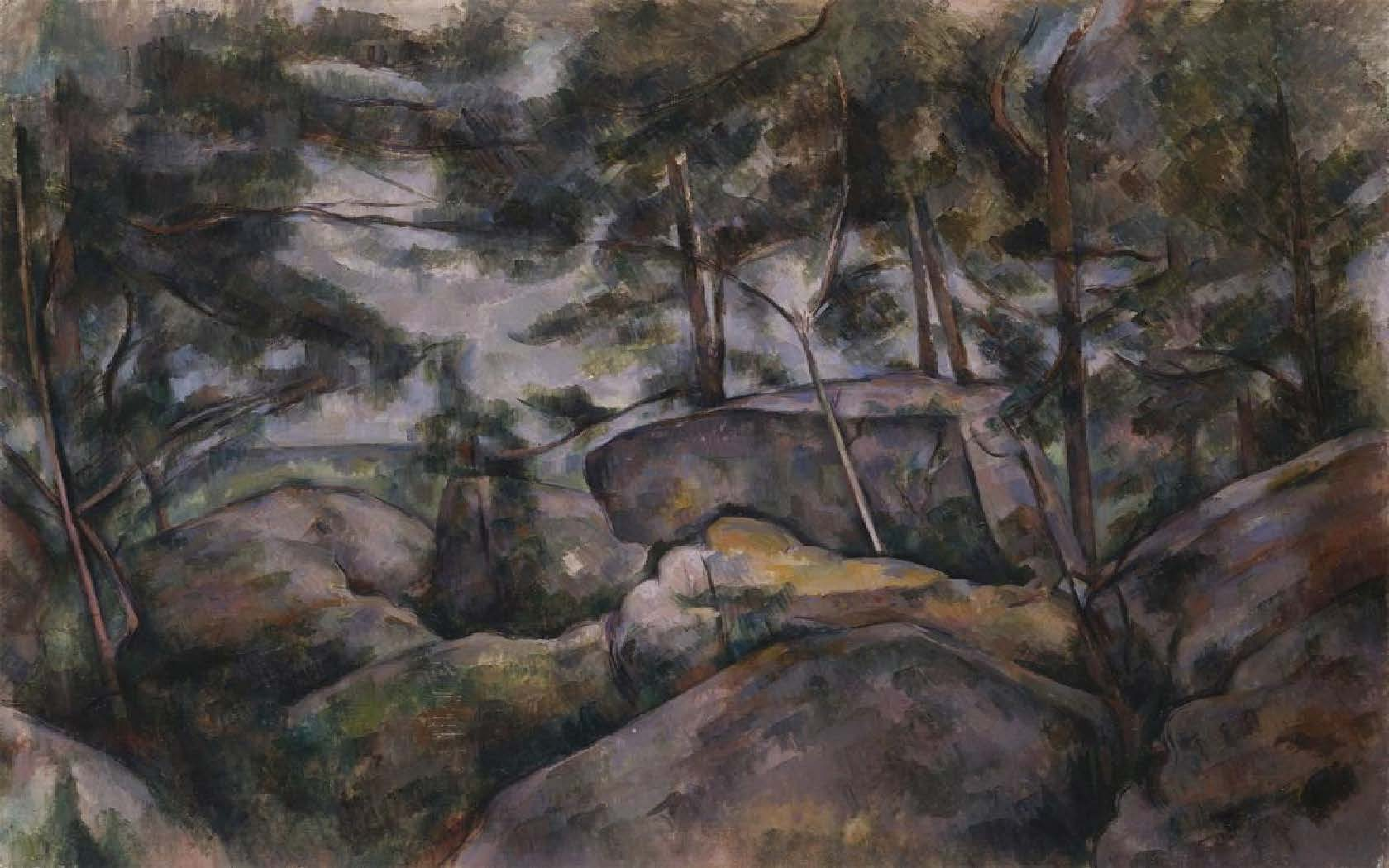

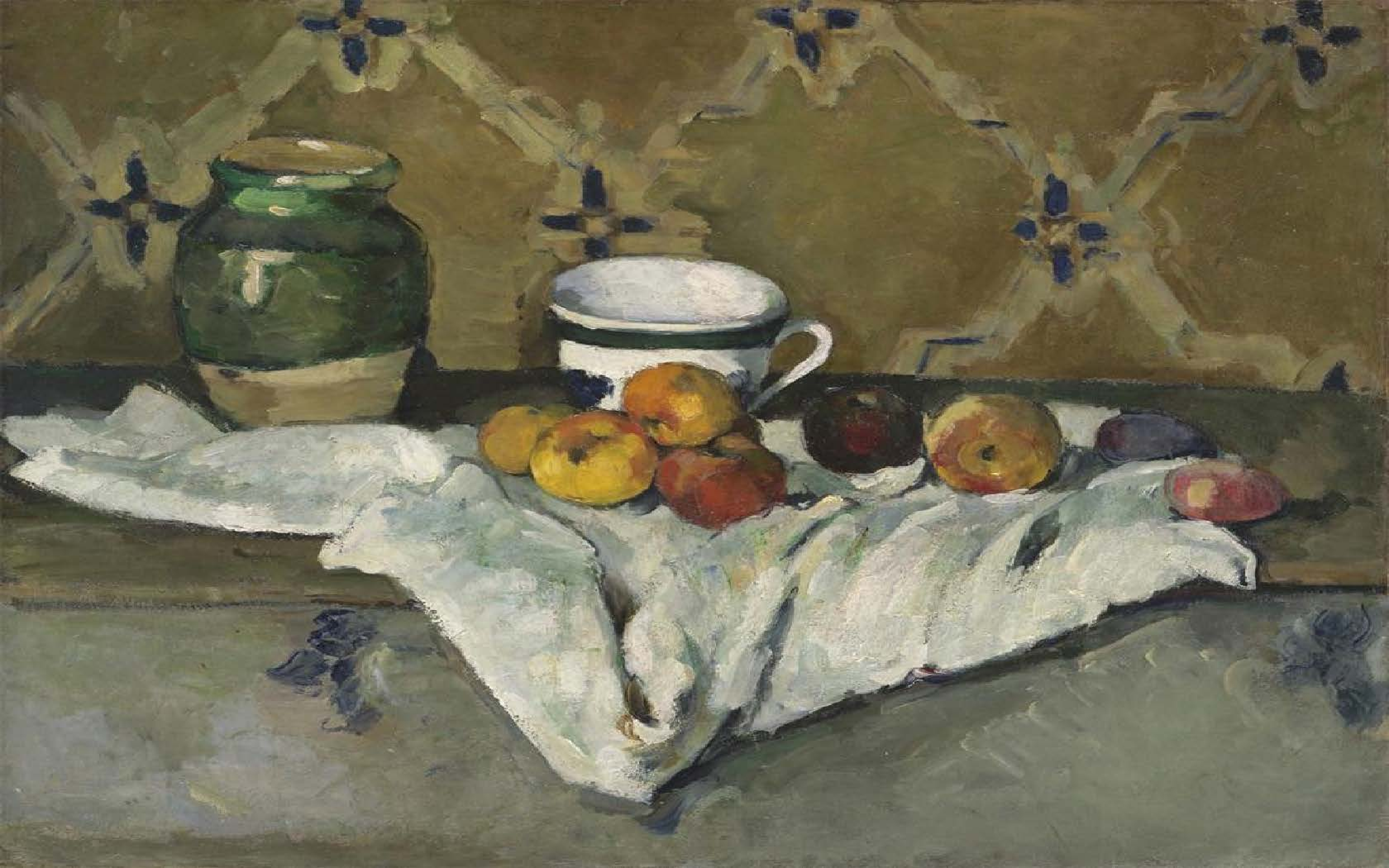

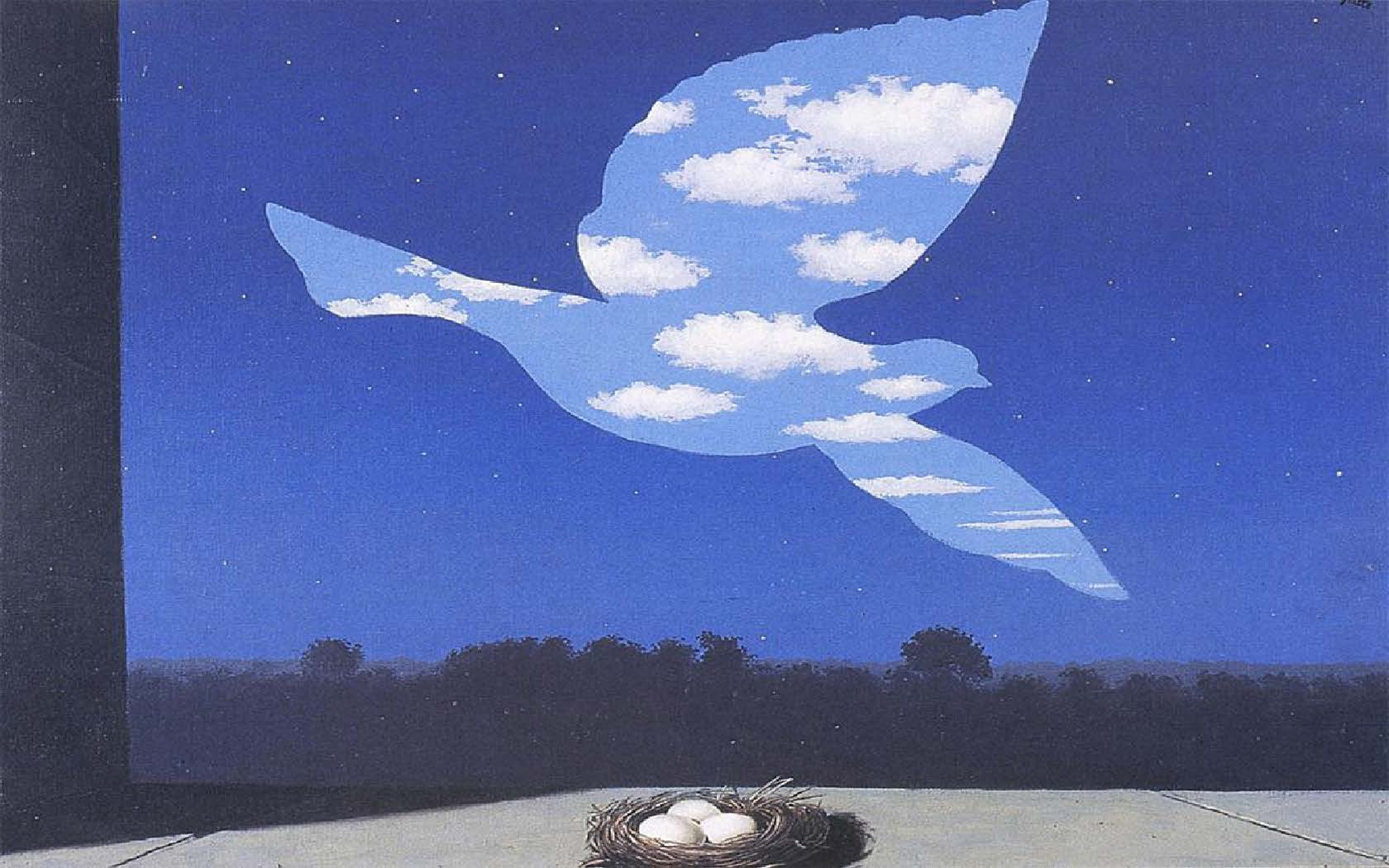

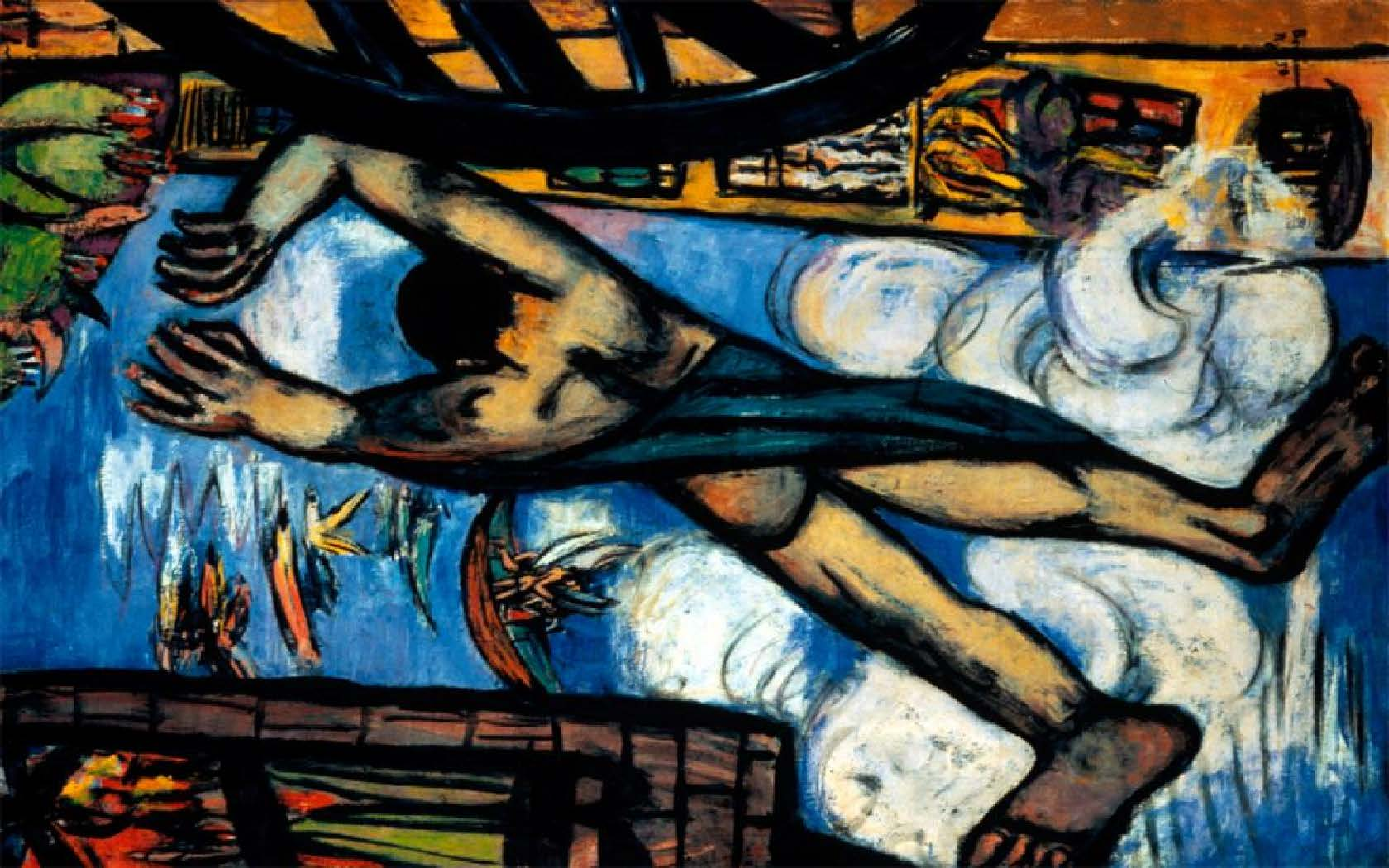

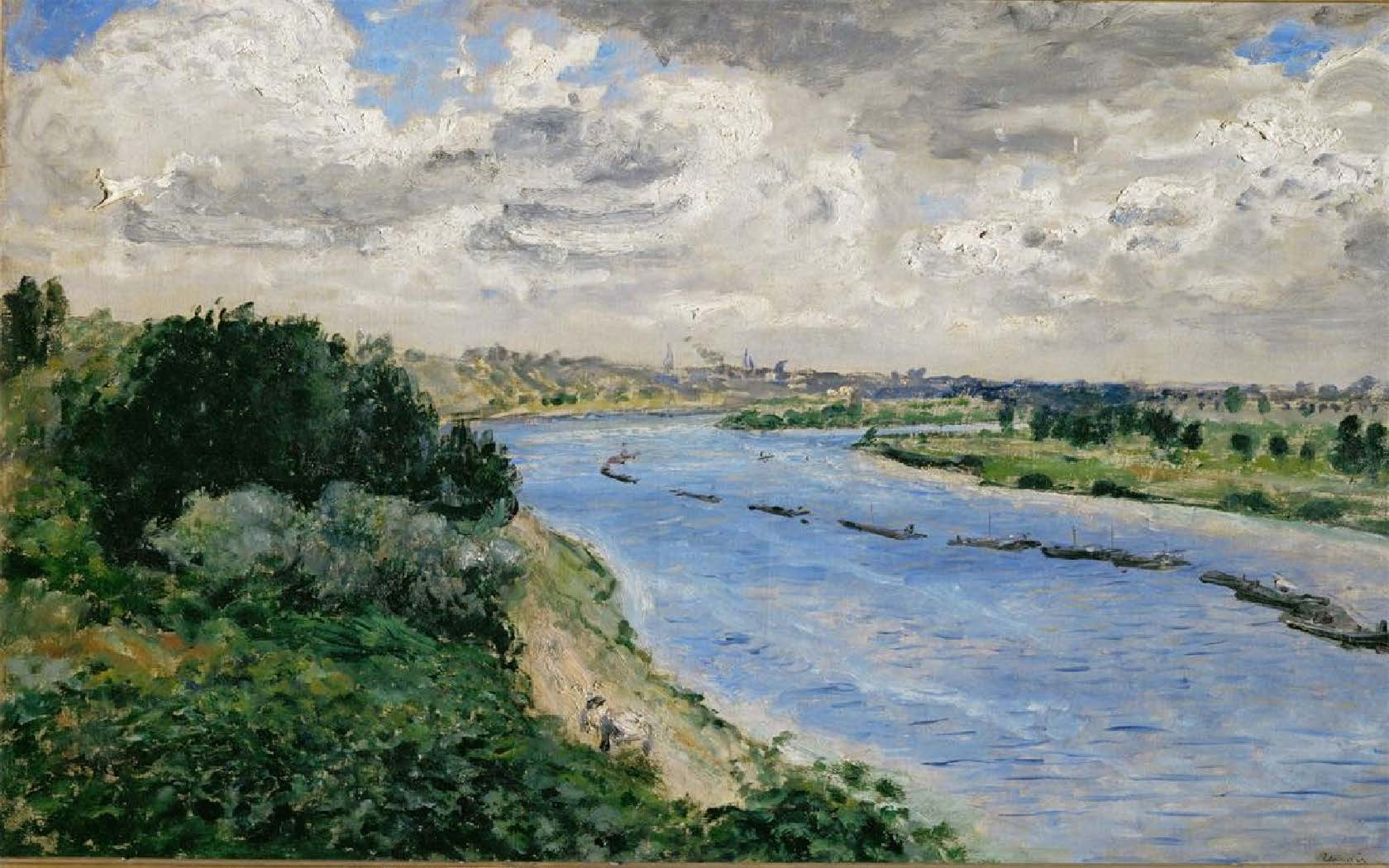

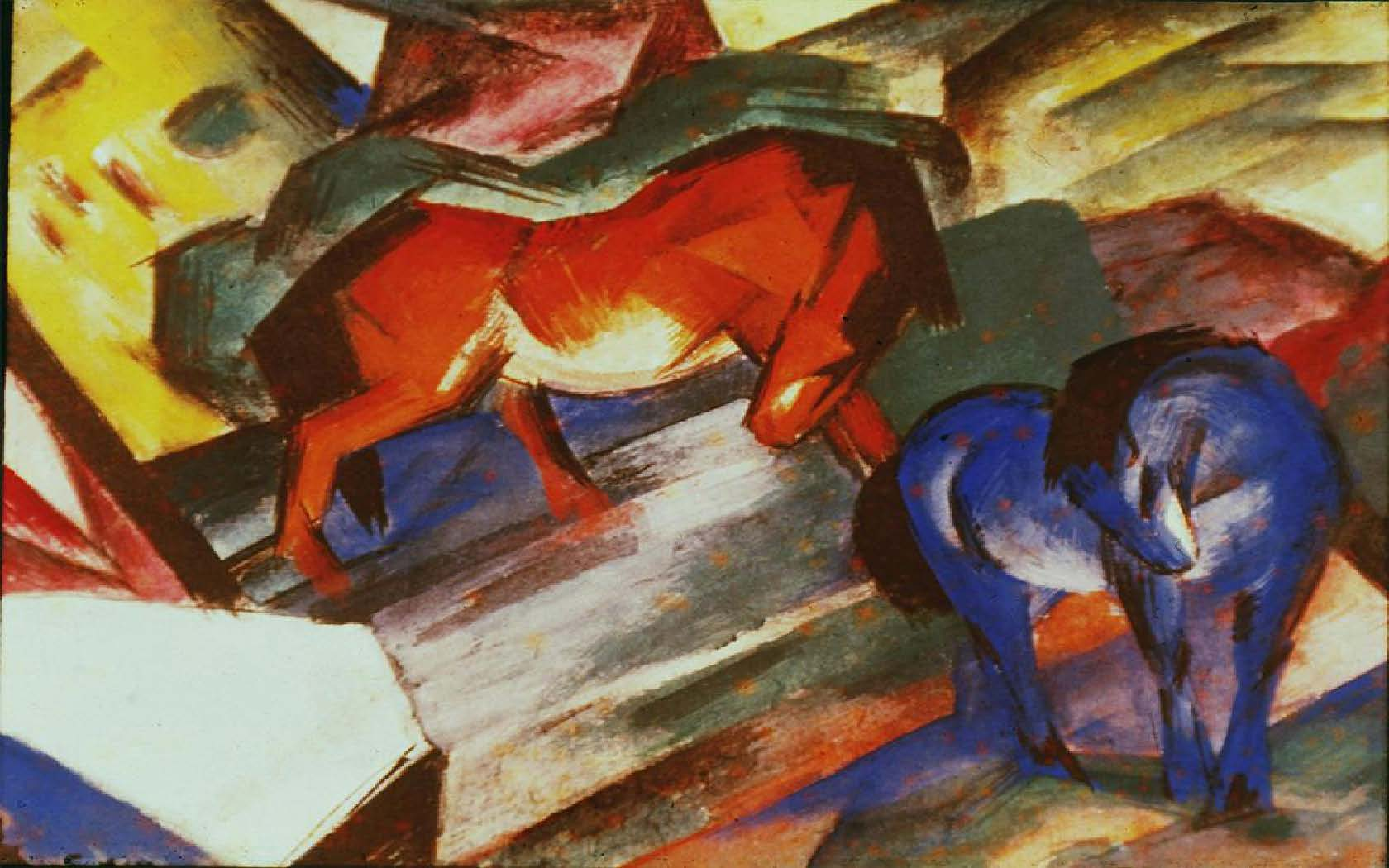

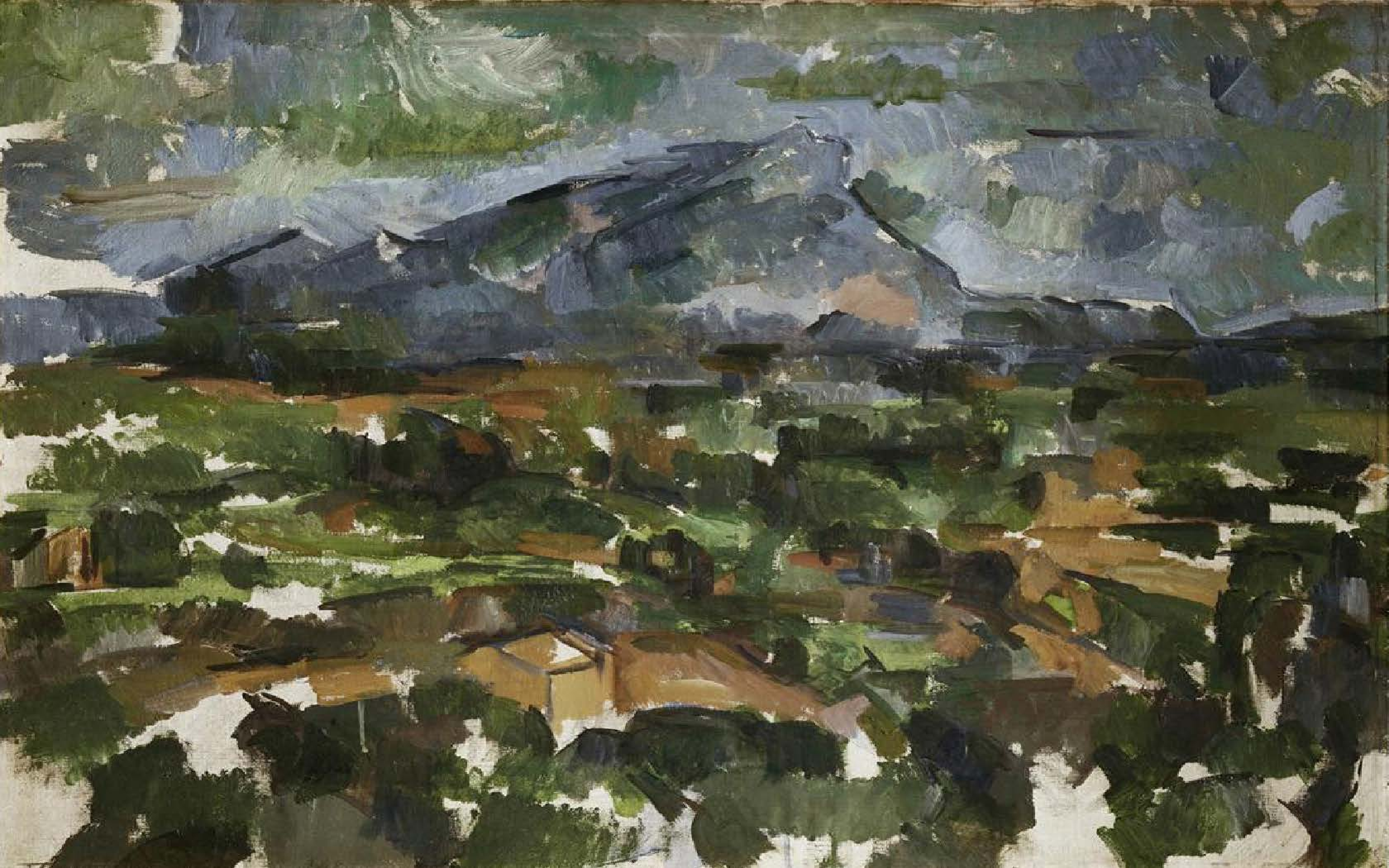

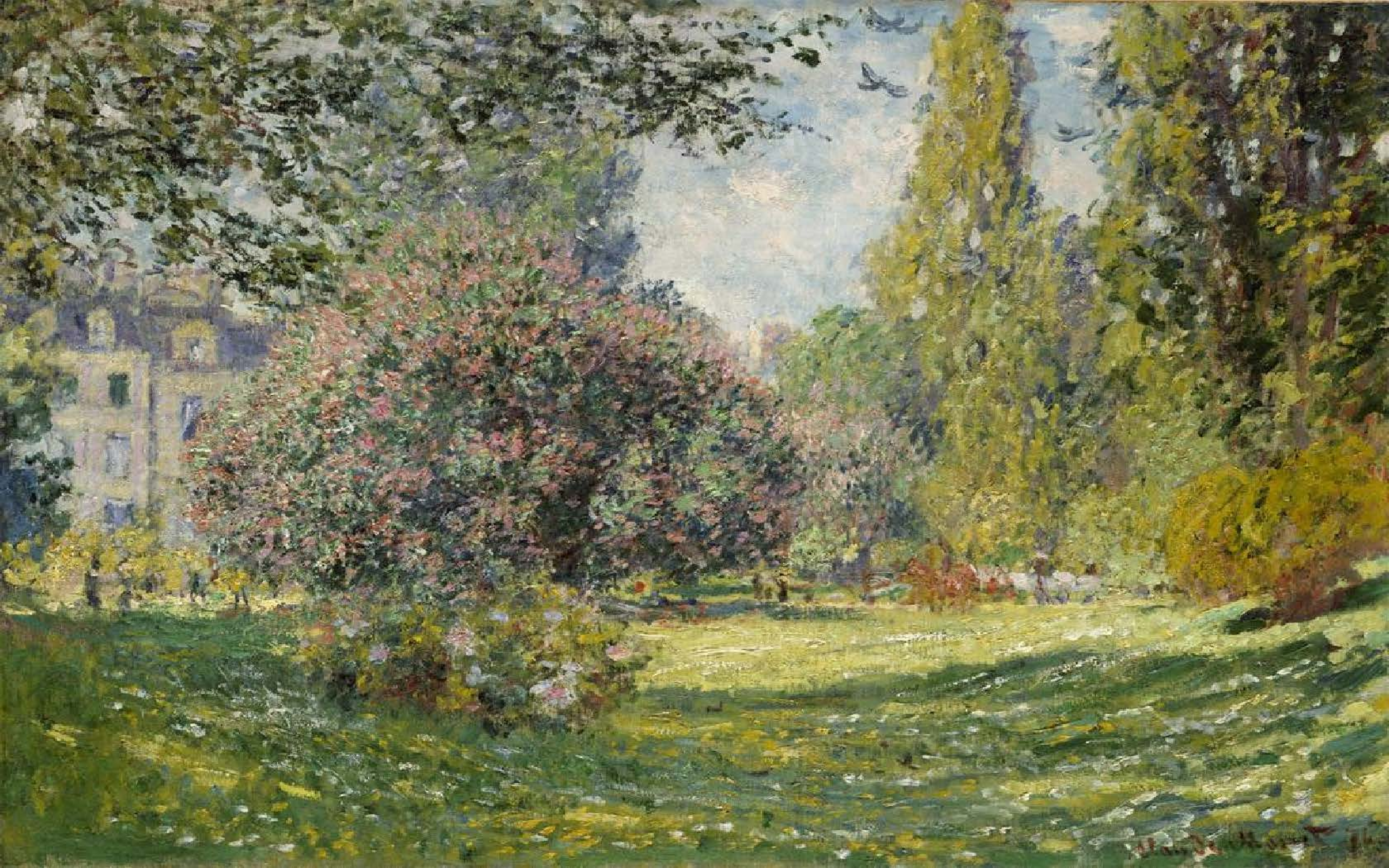

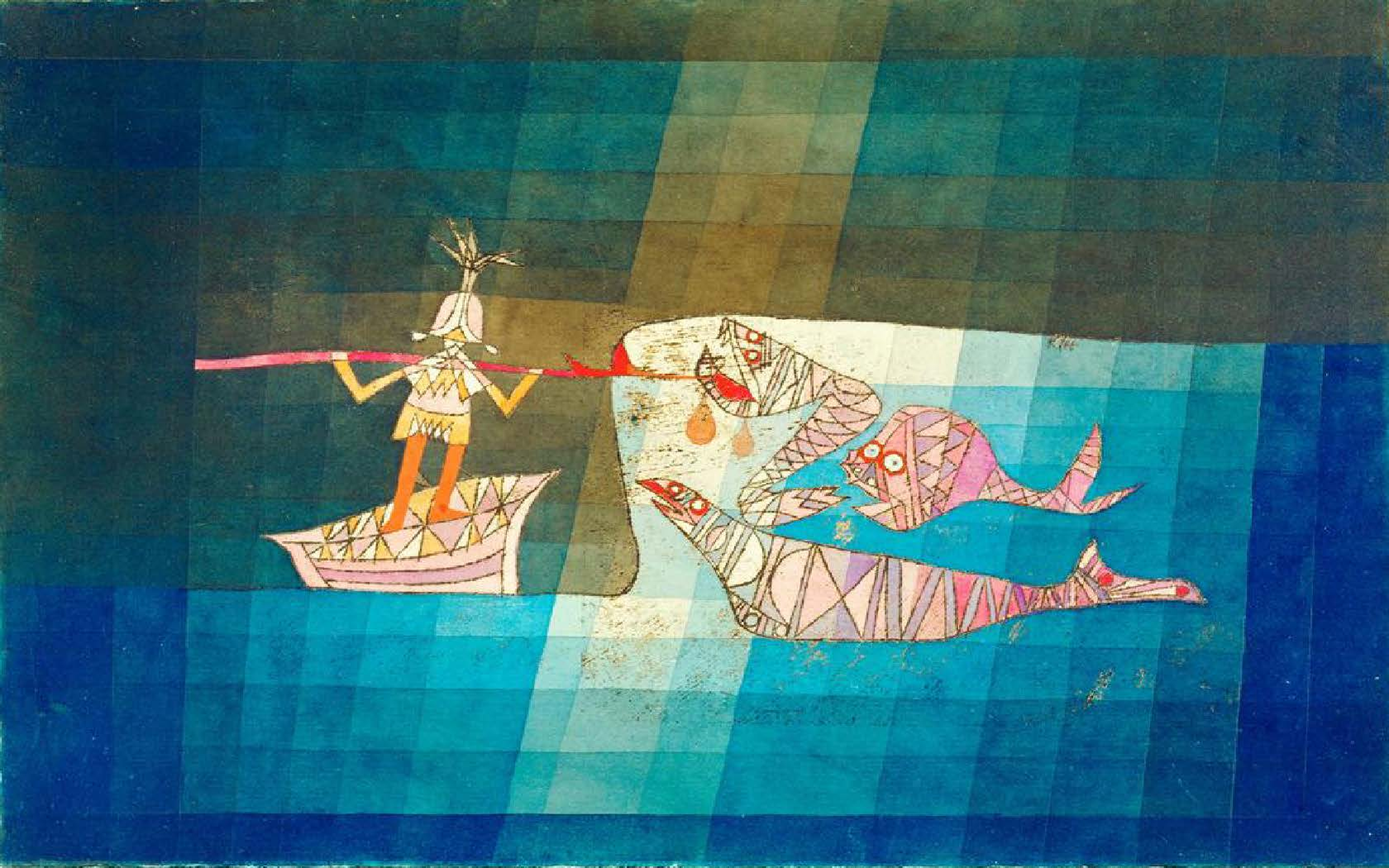

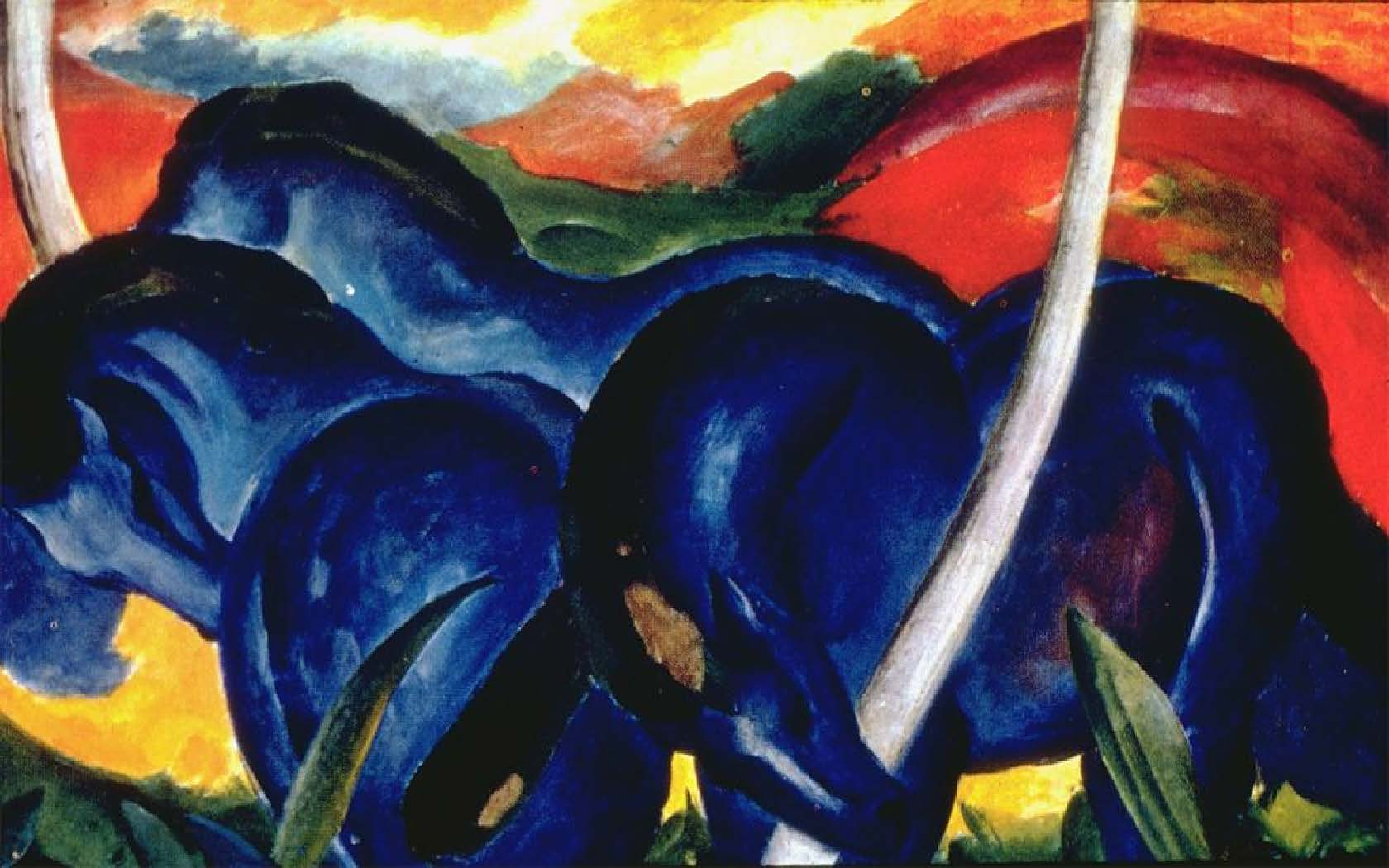

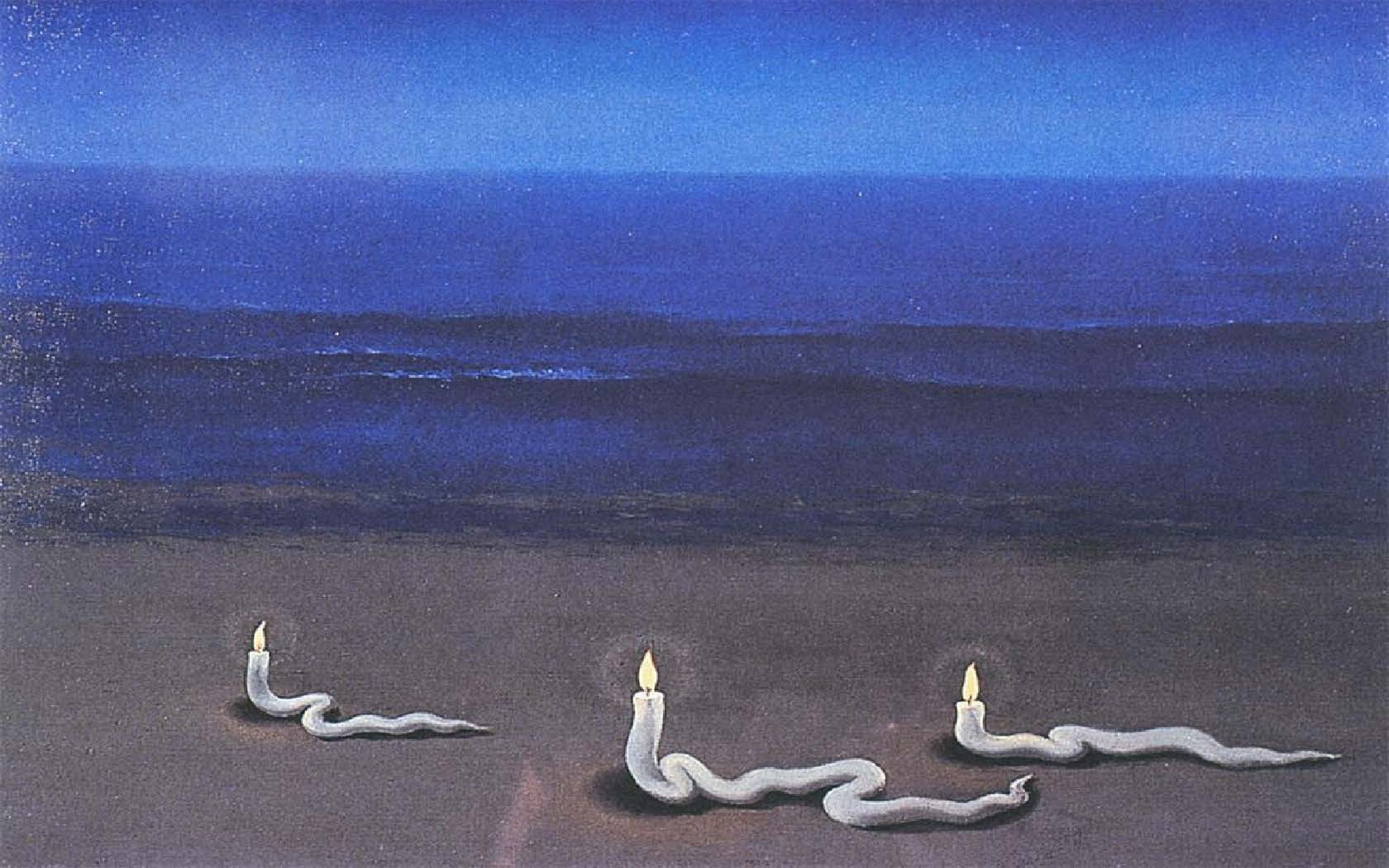

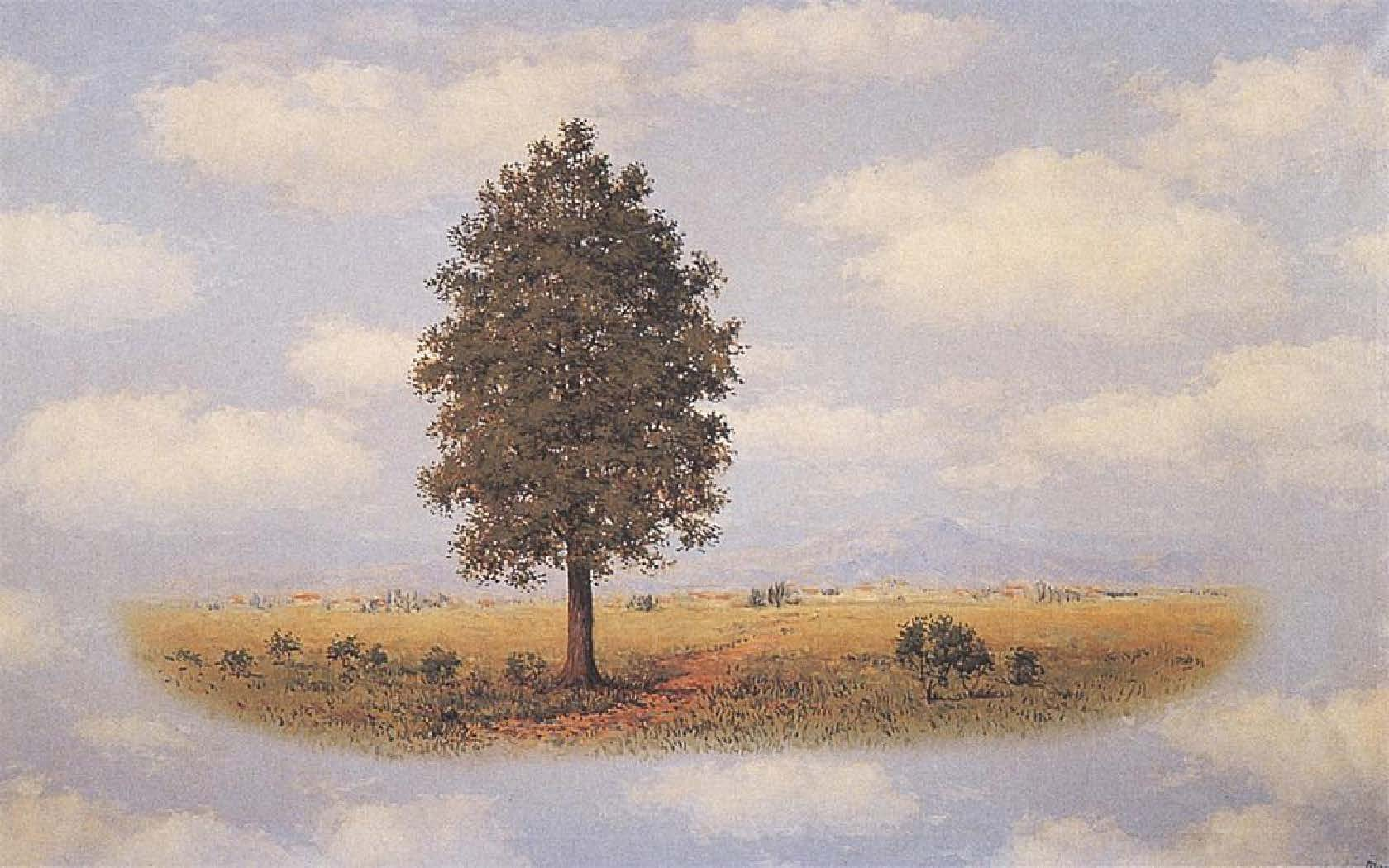

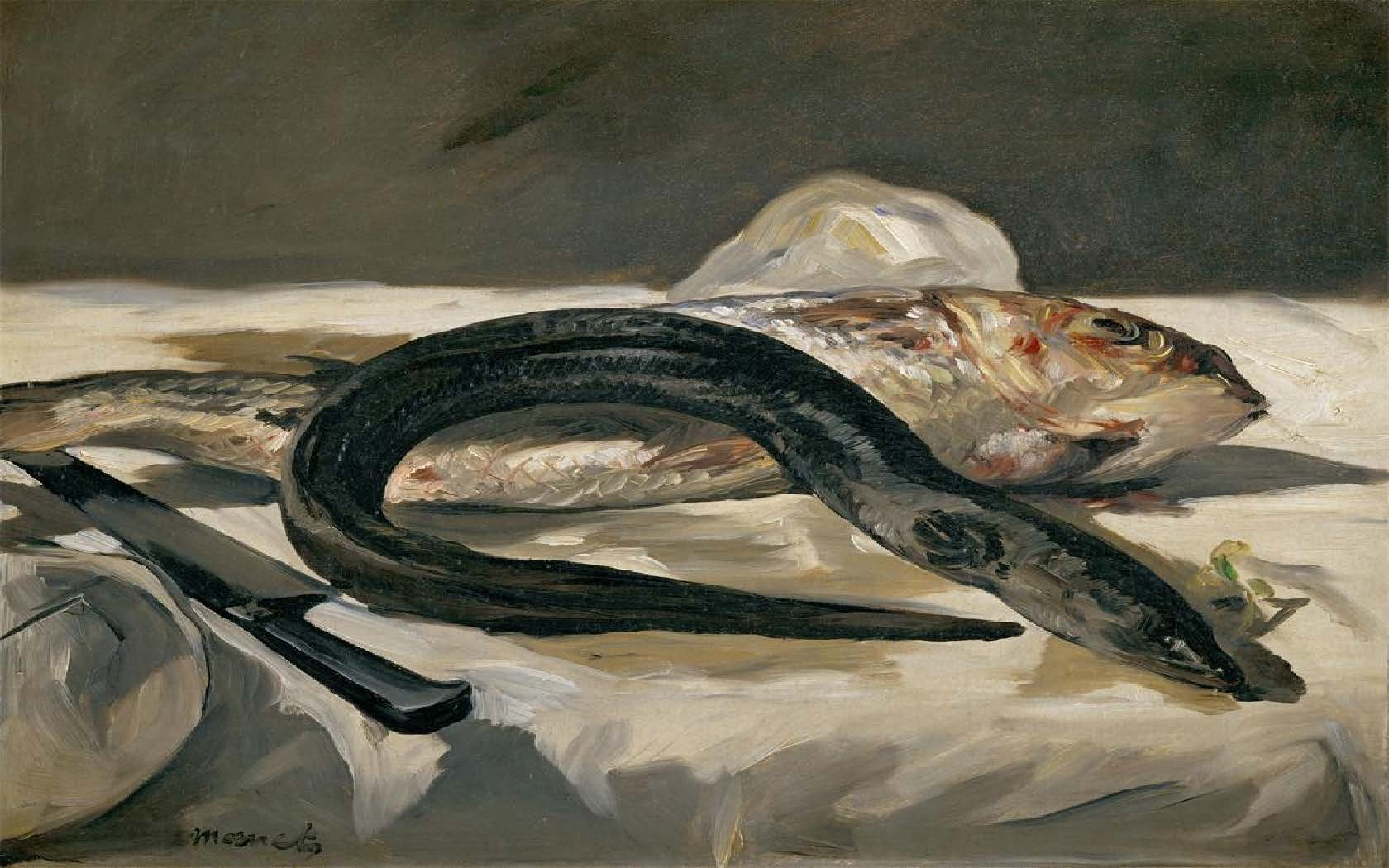
manet

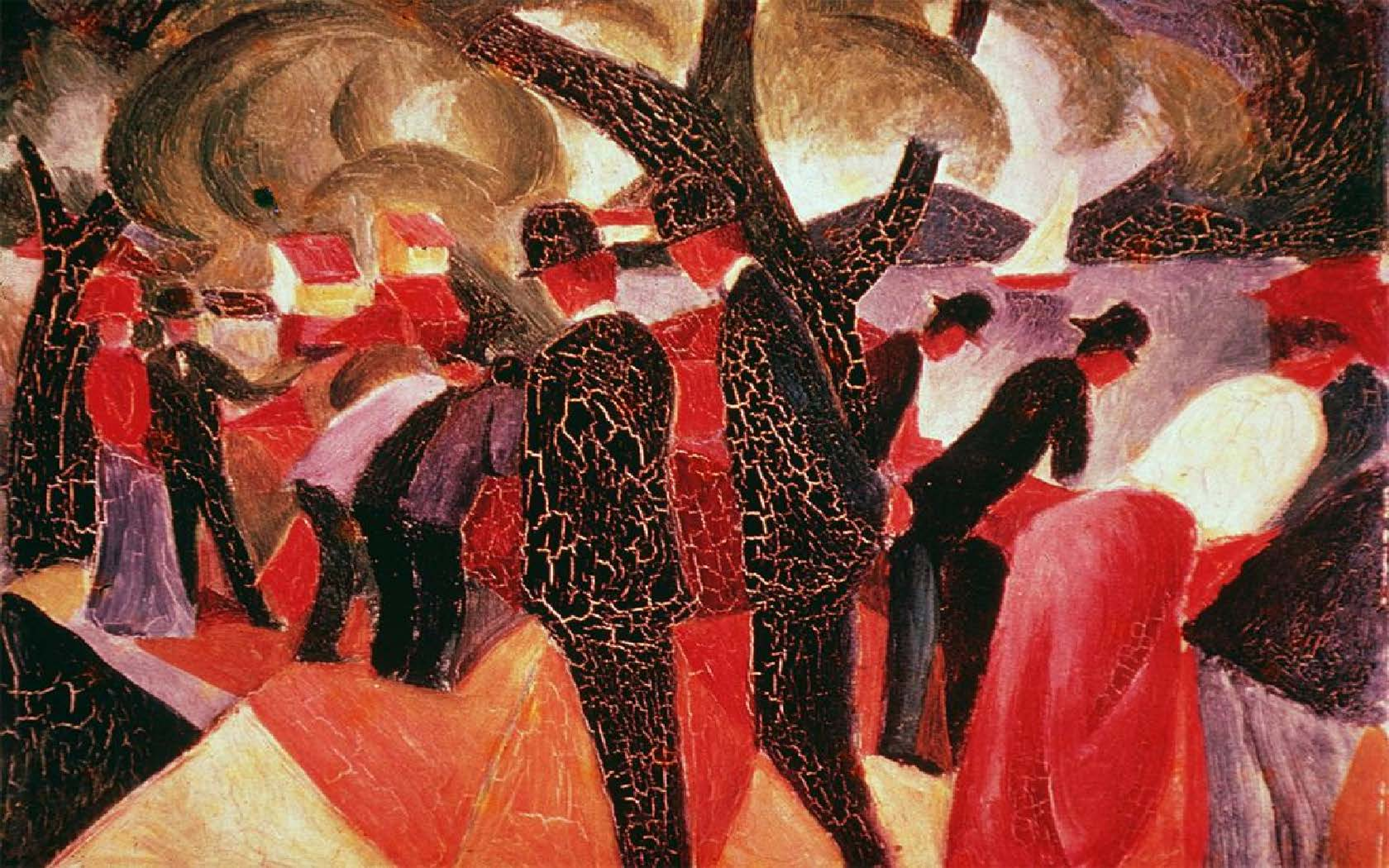

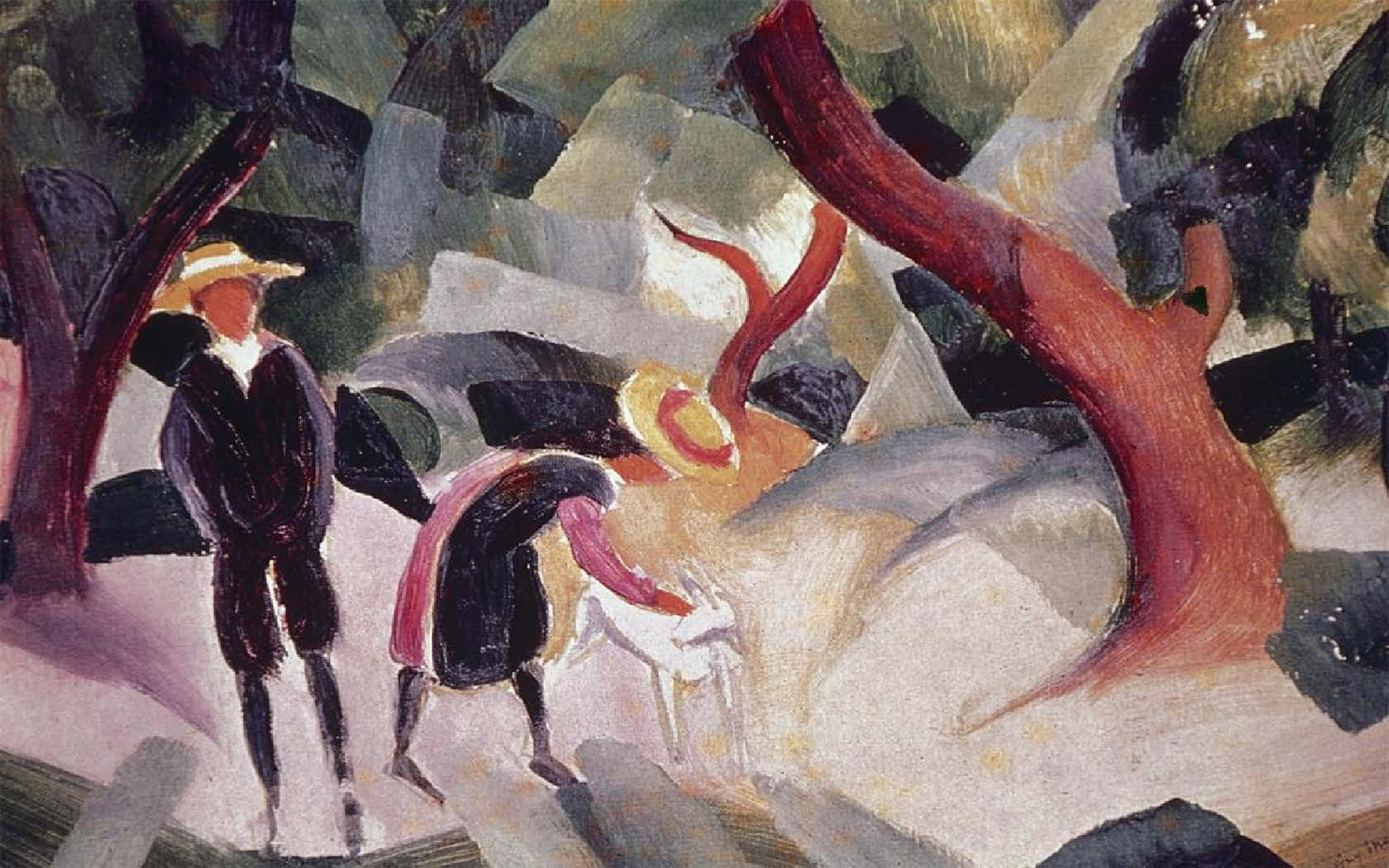

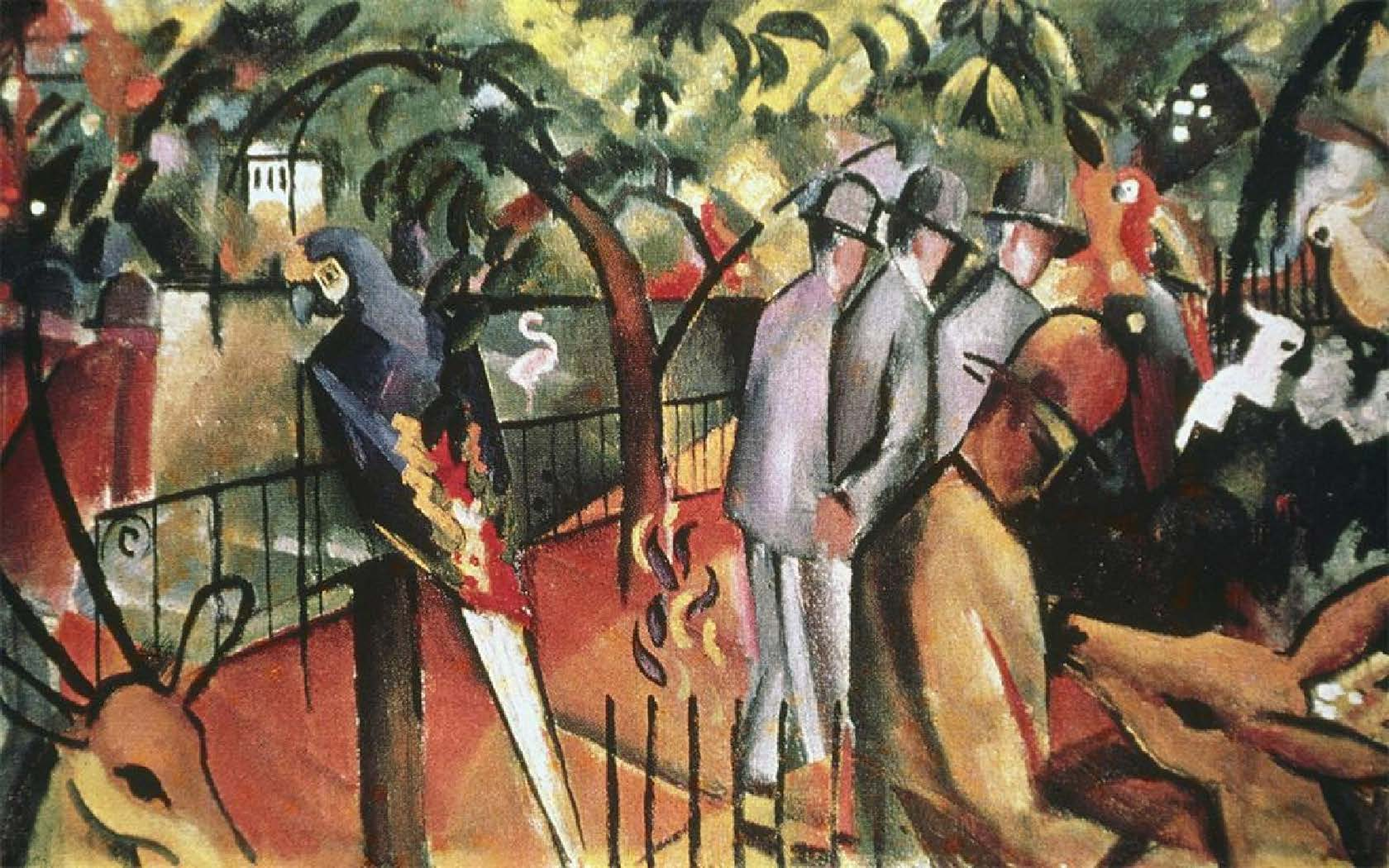

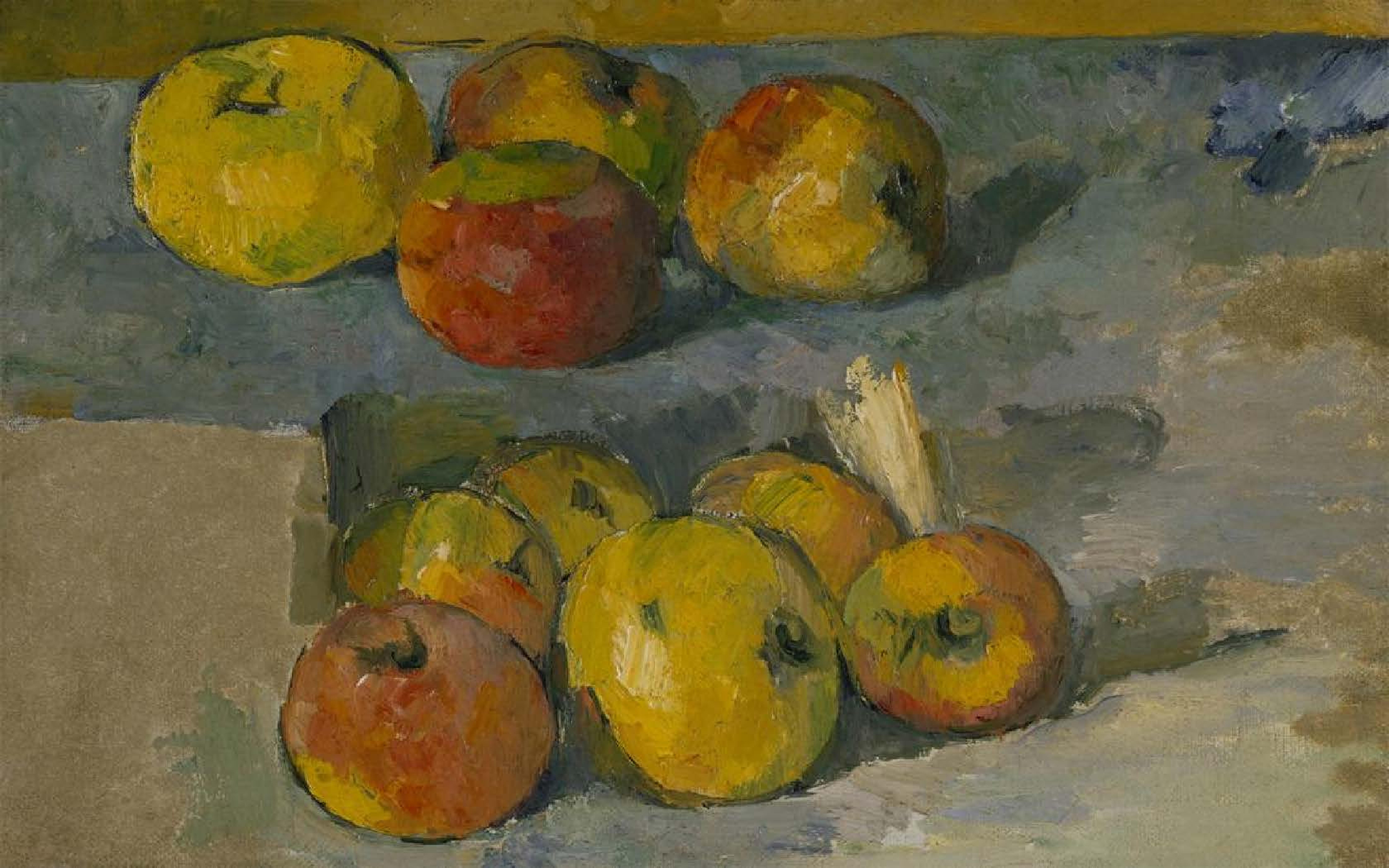

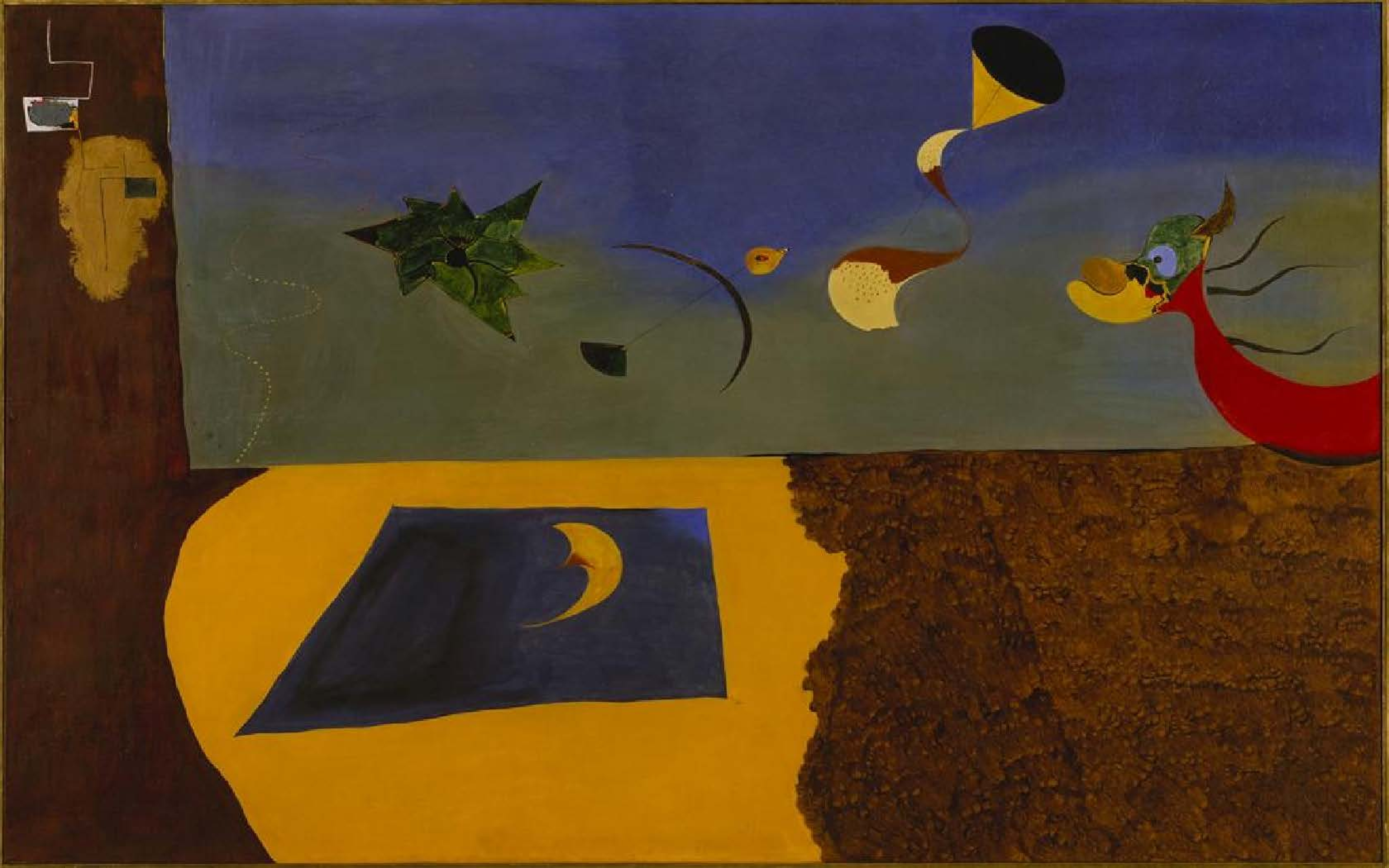

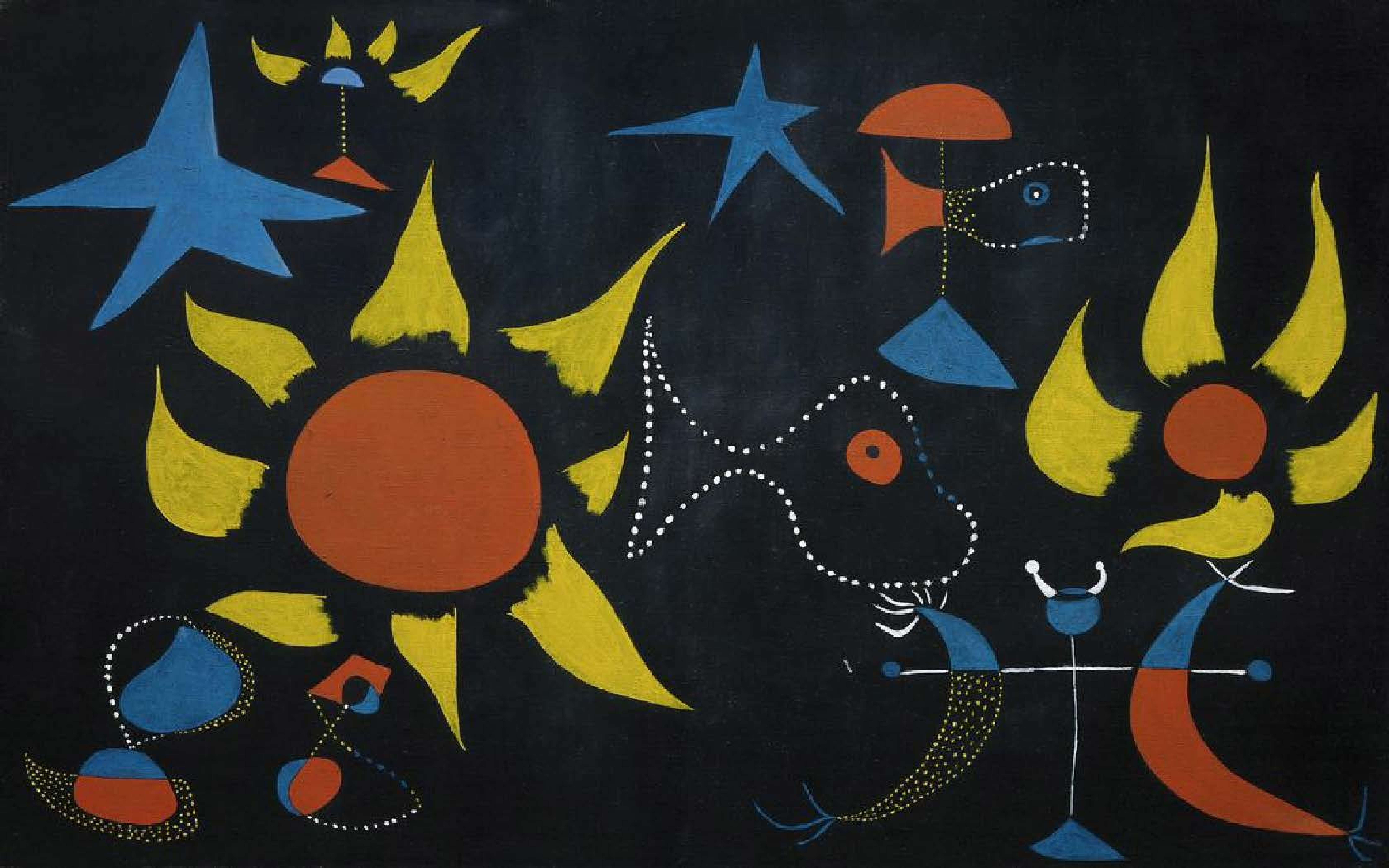

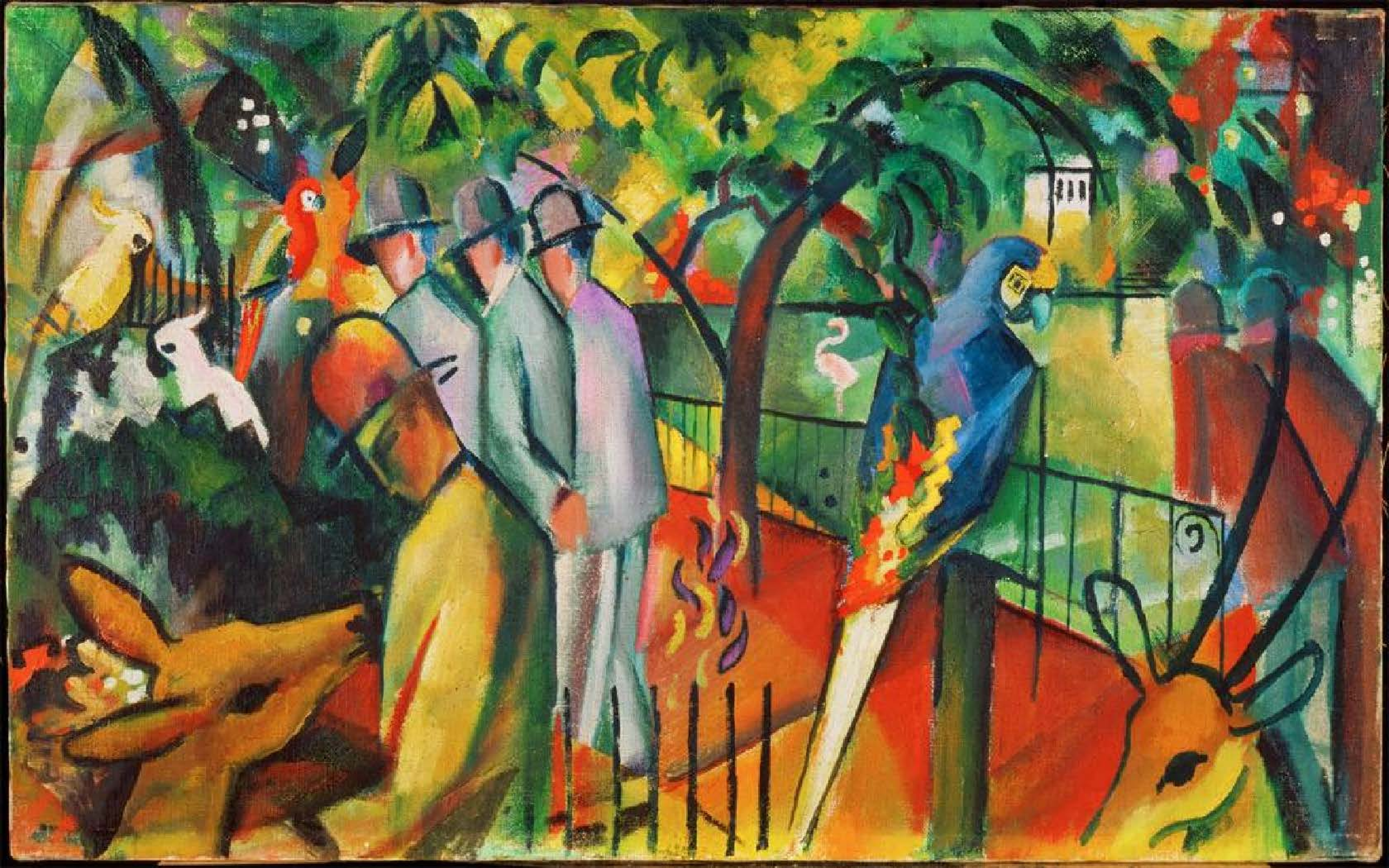

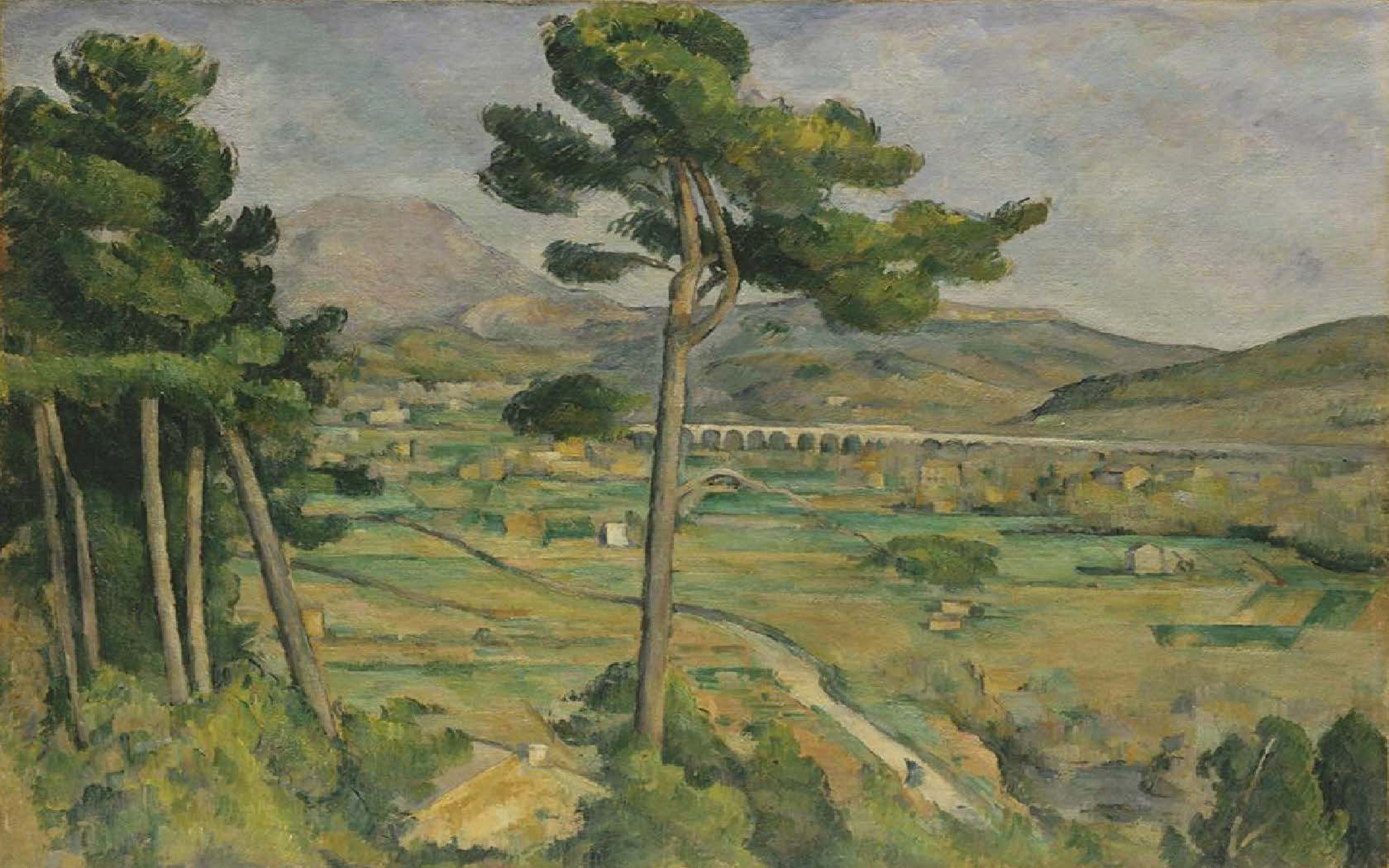

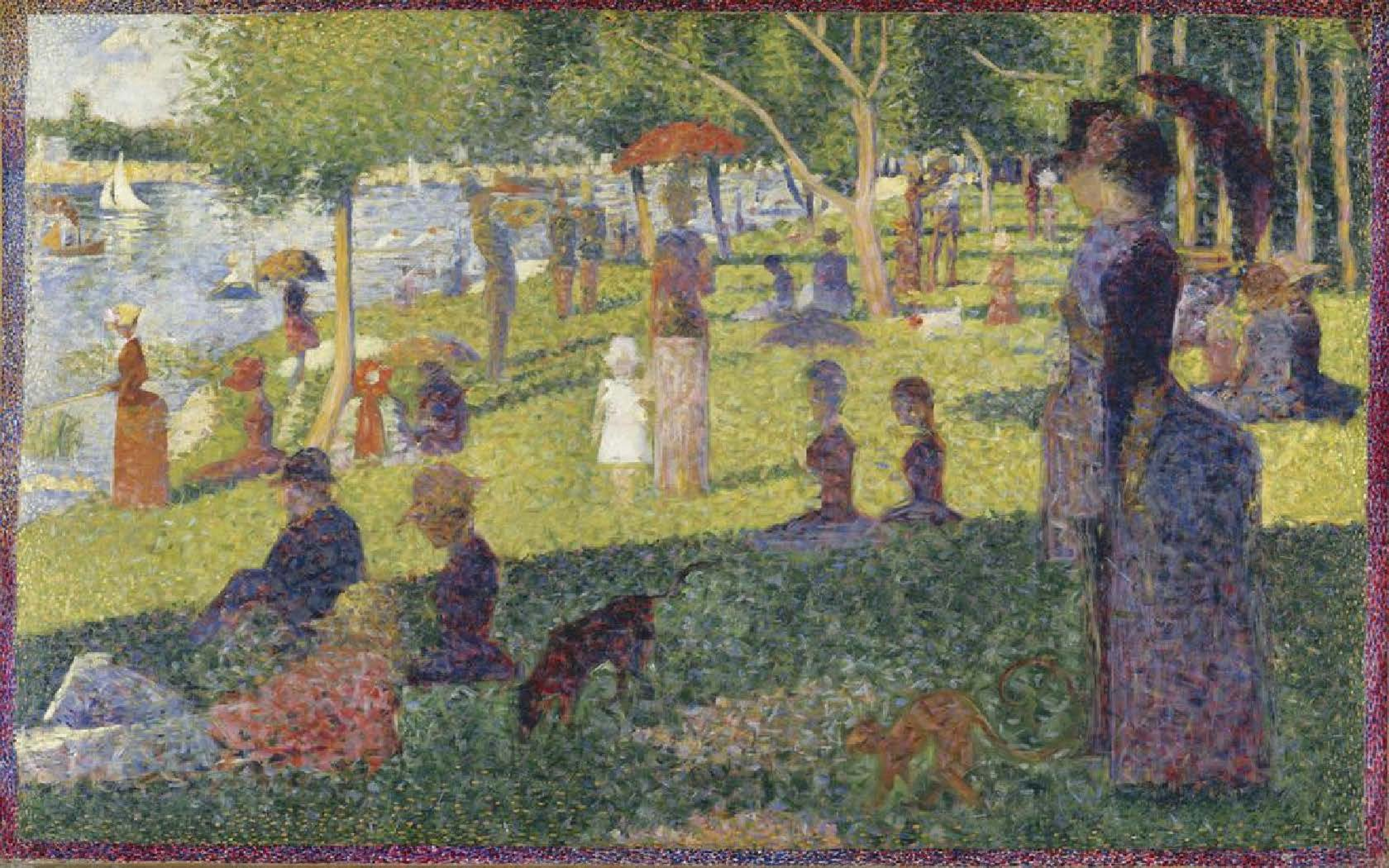

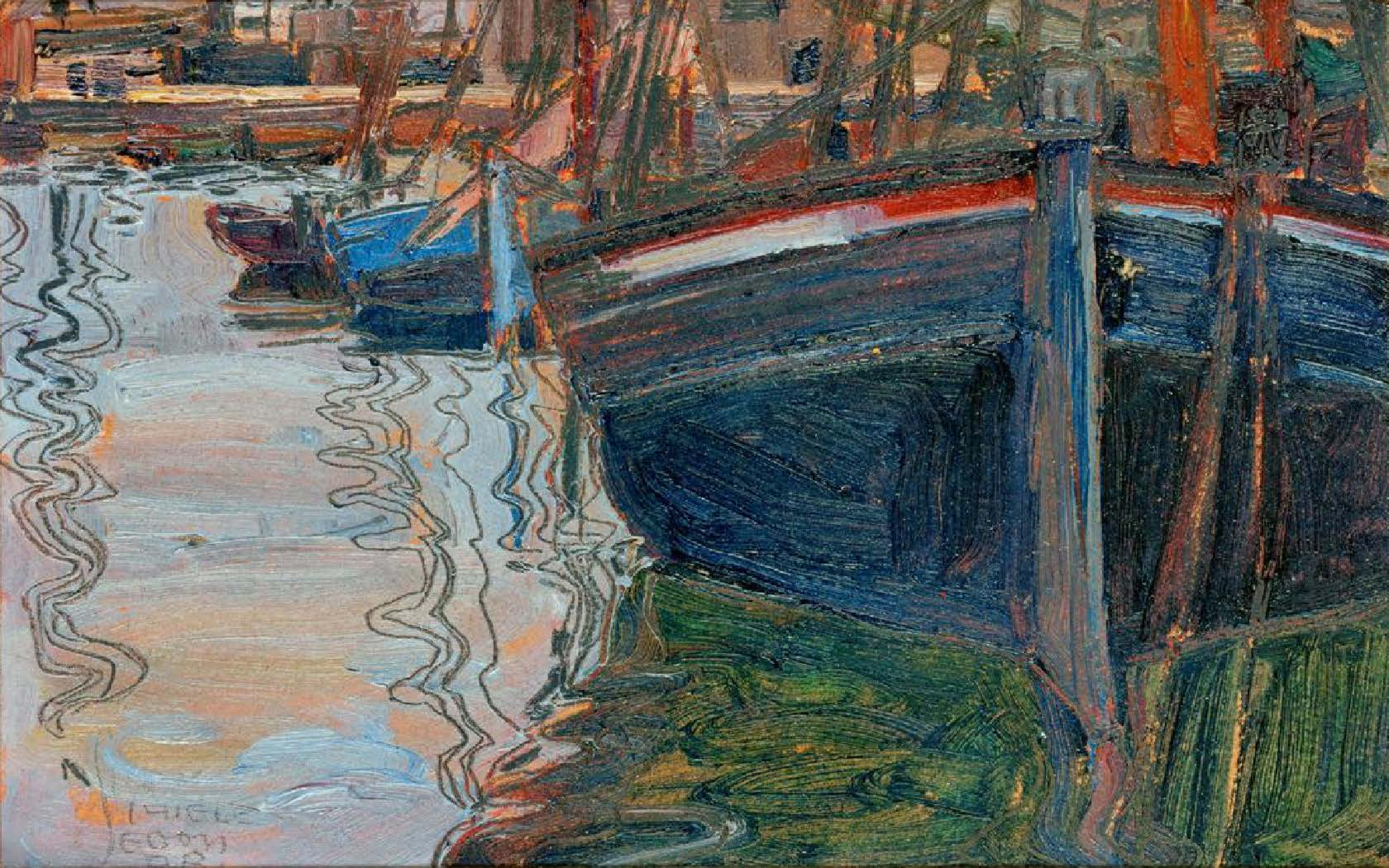

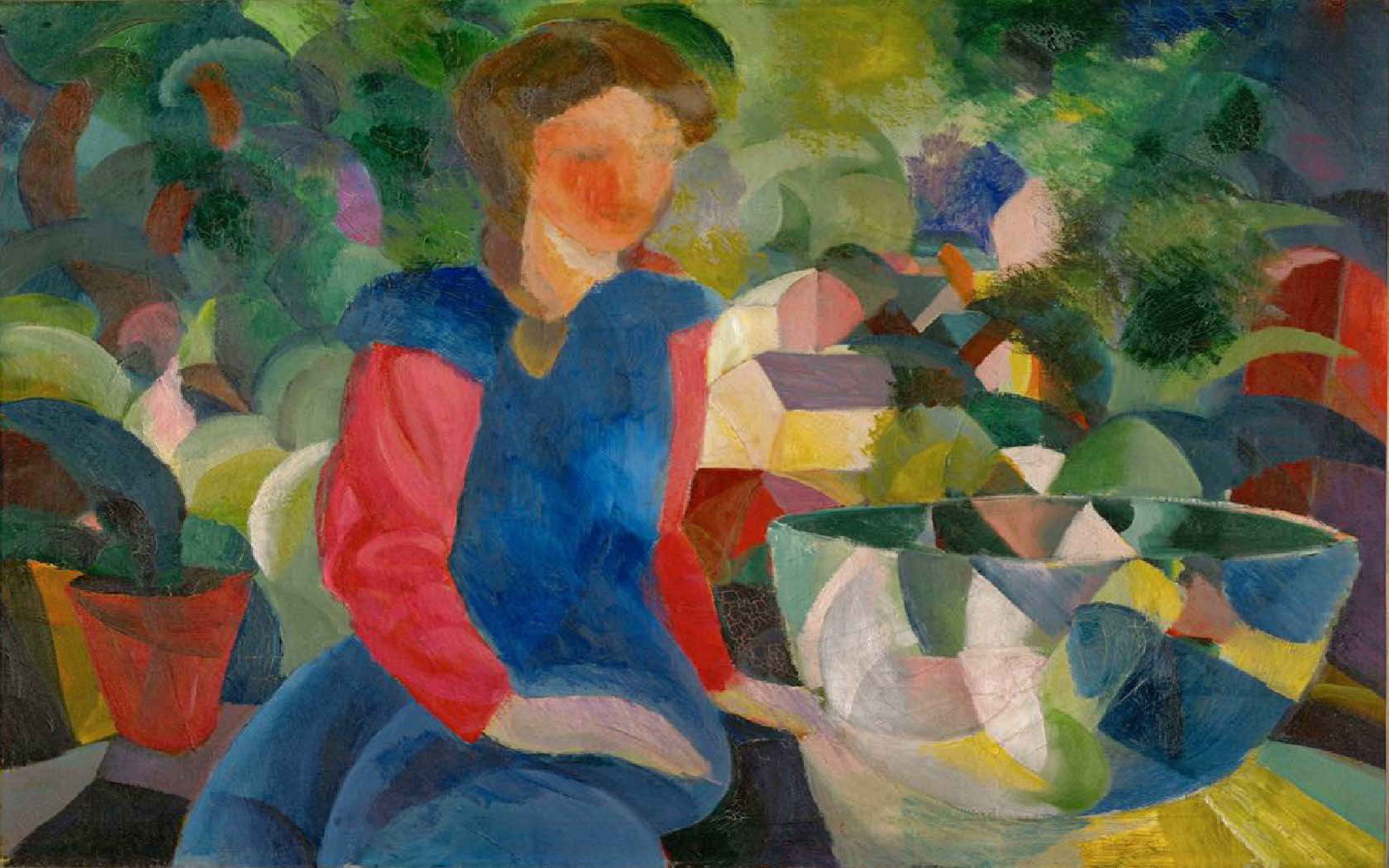

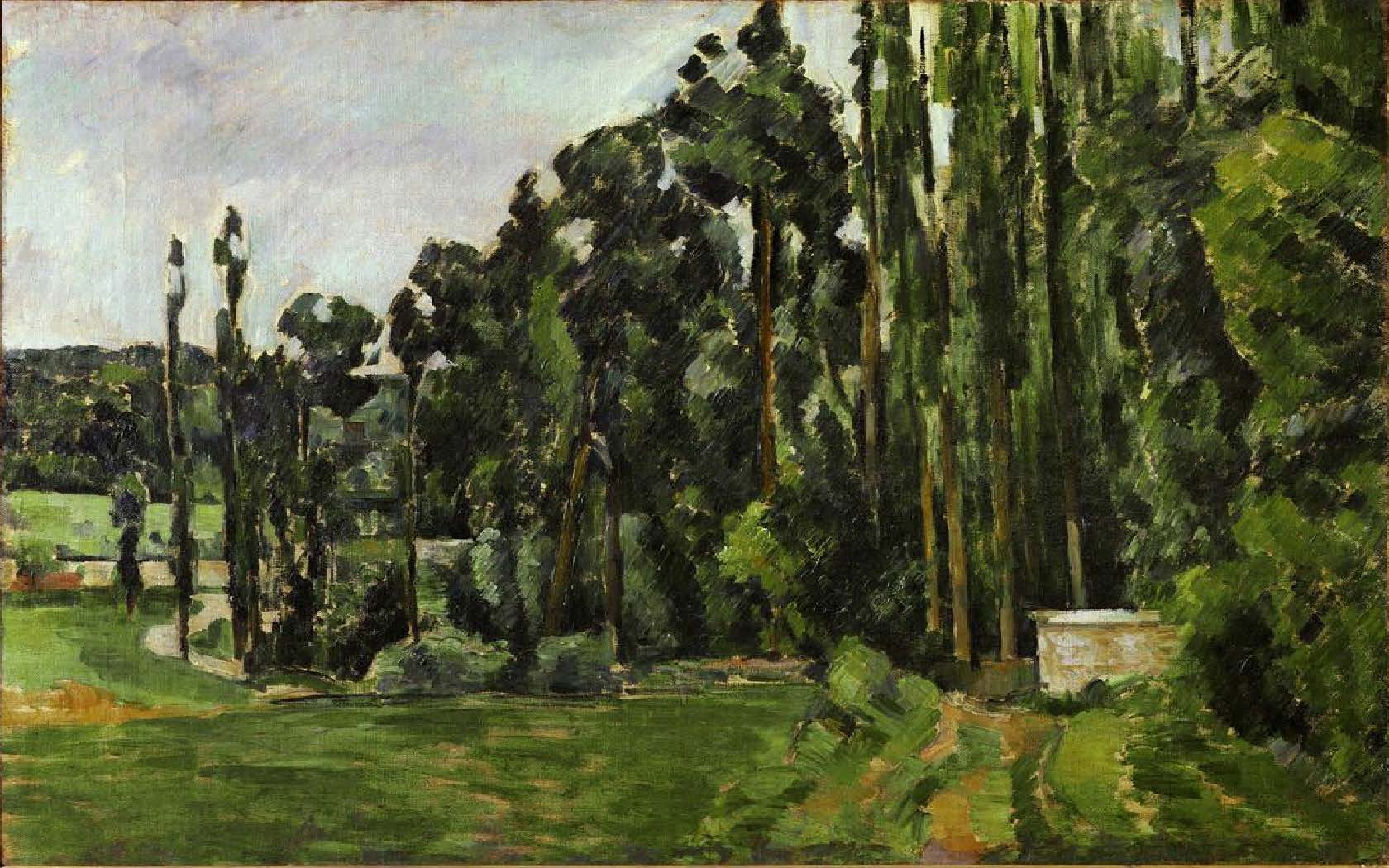

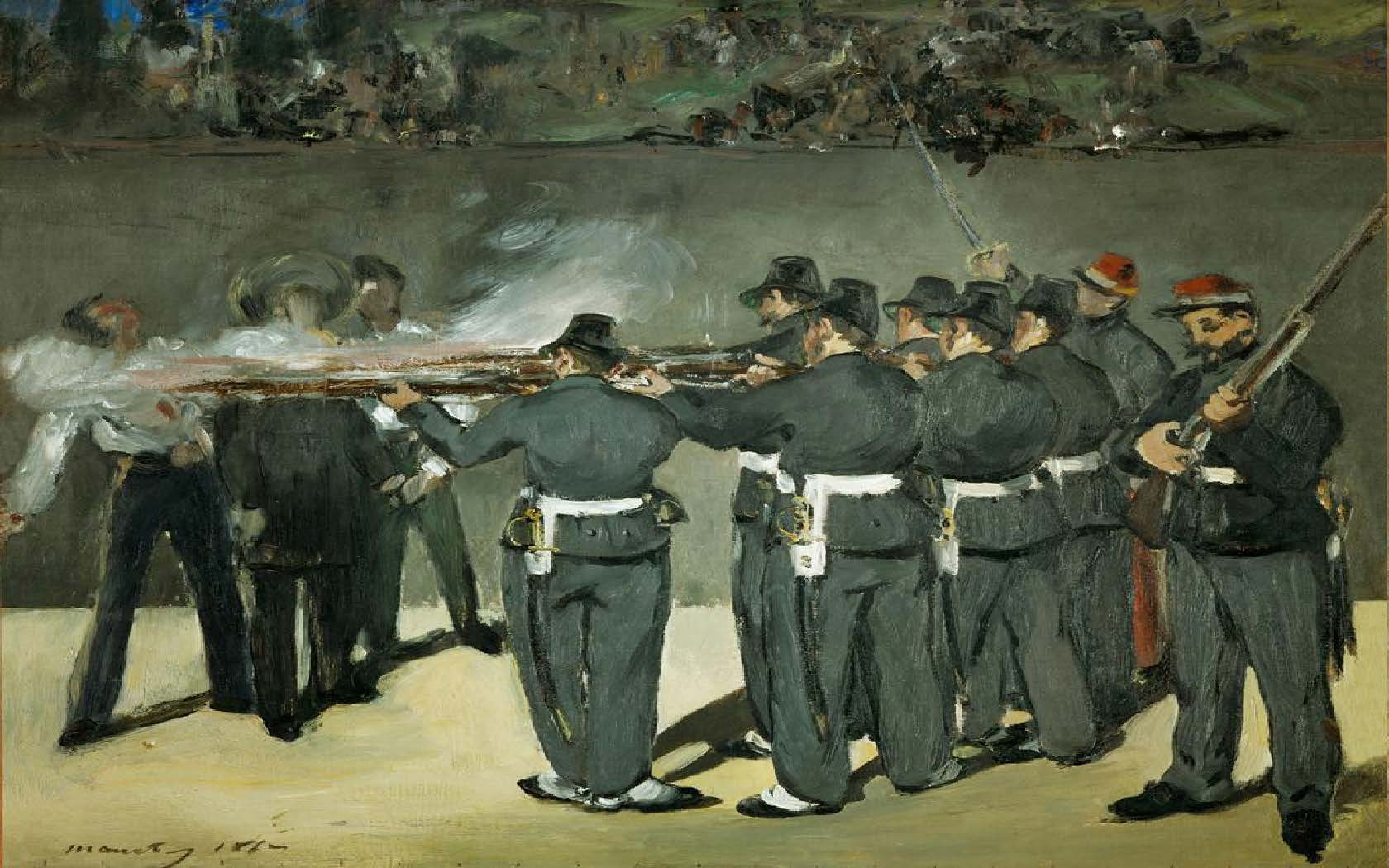

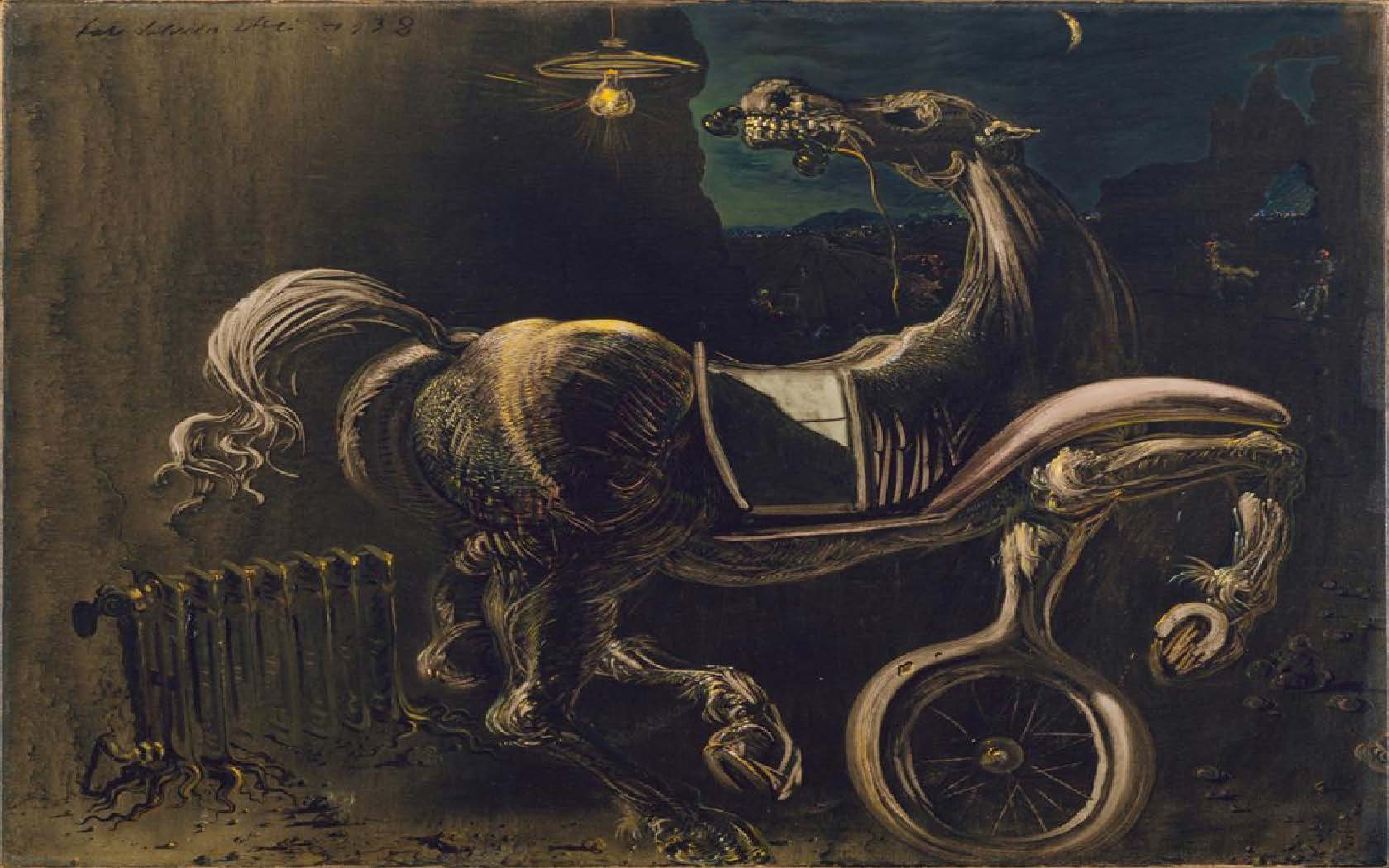

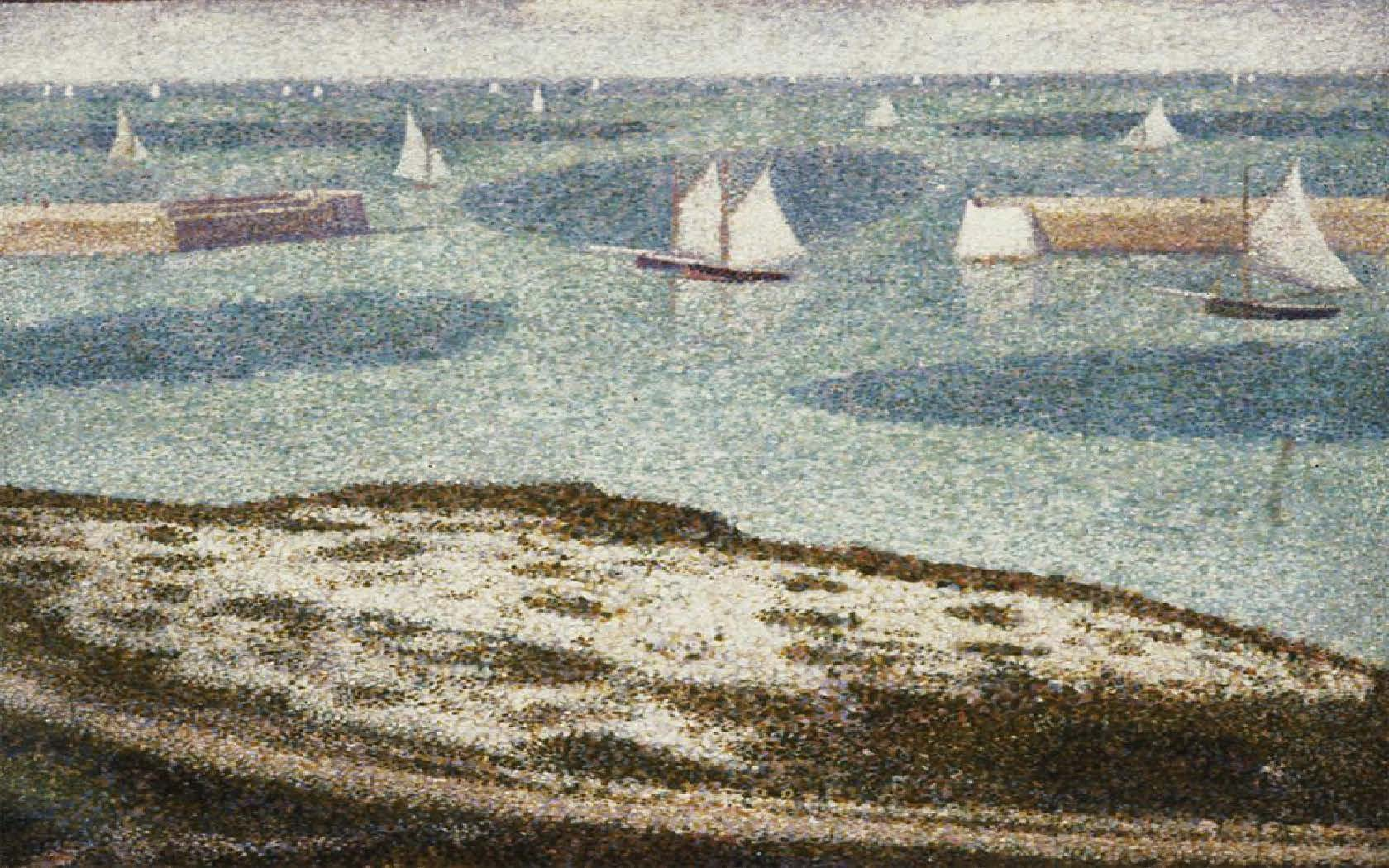

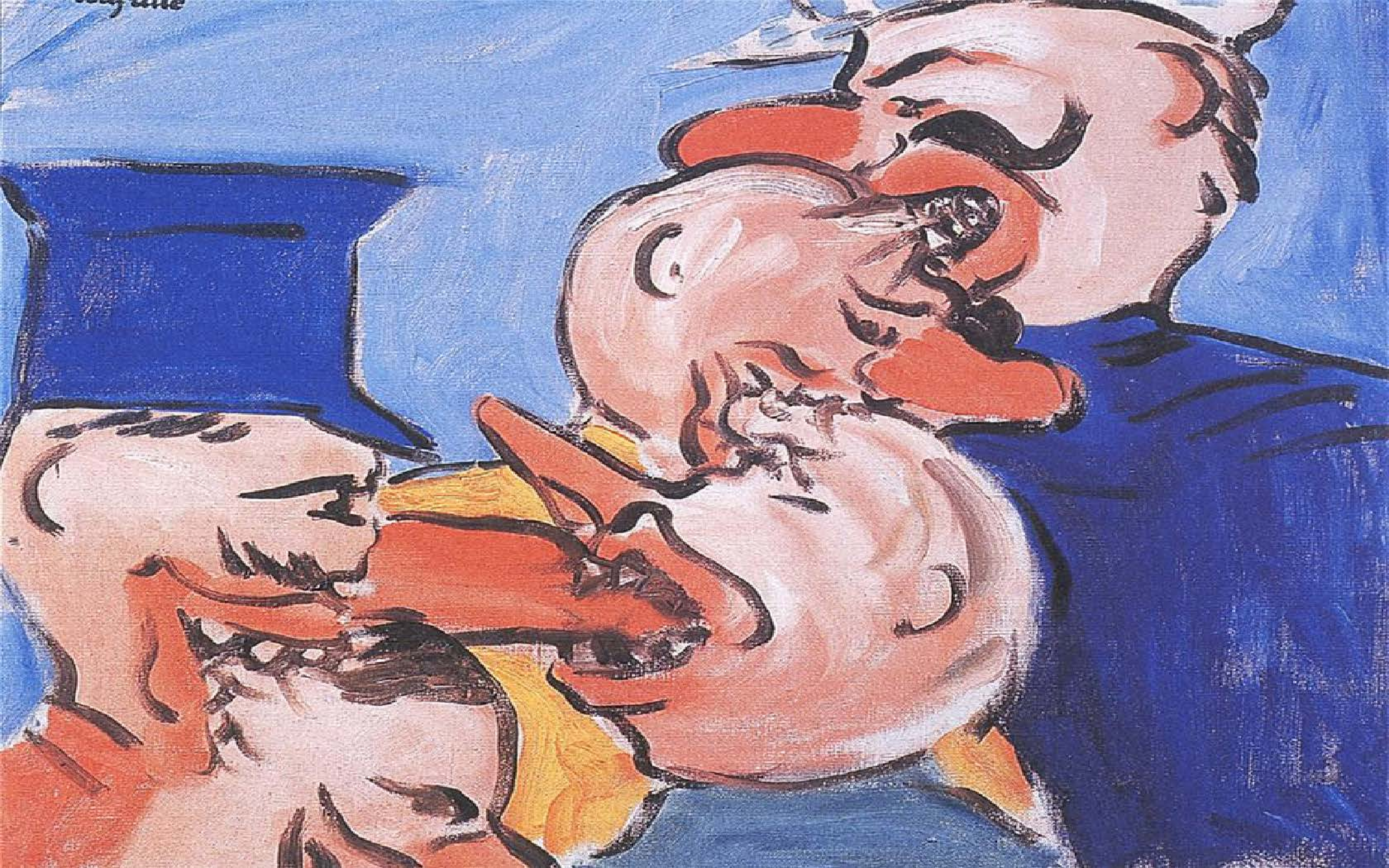

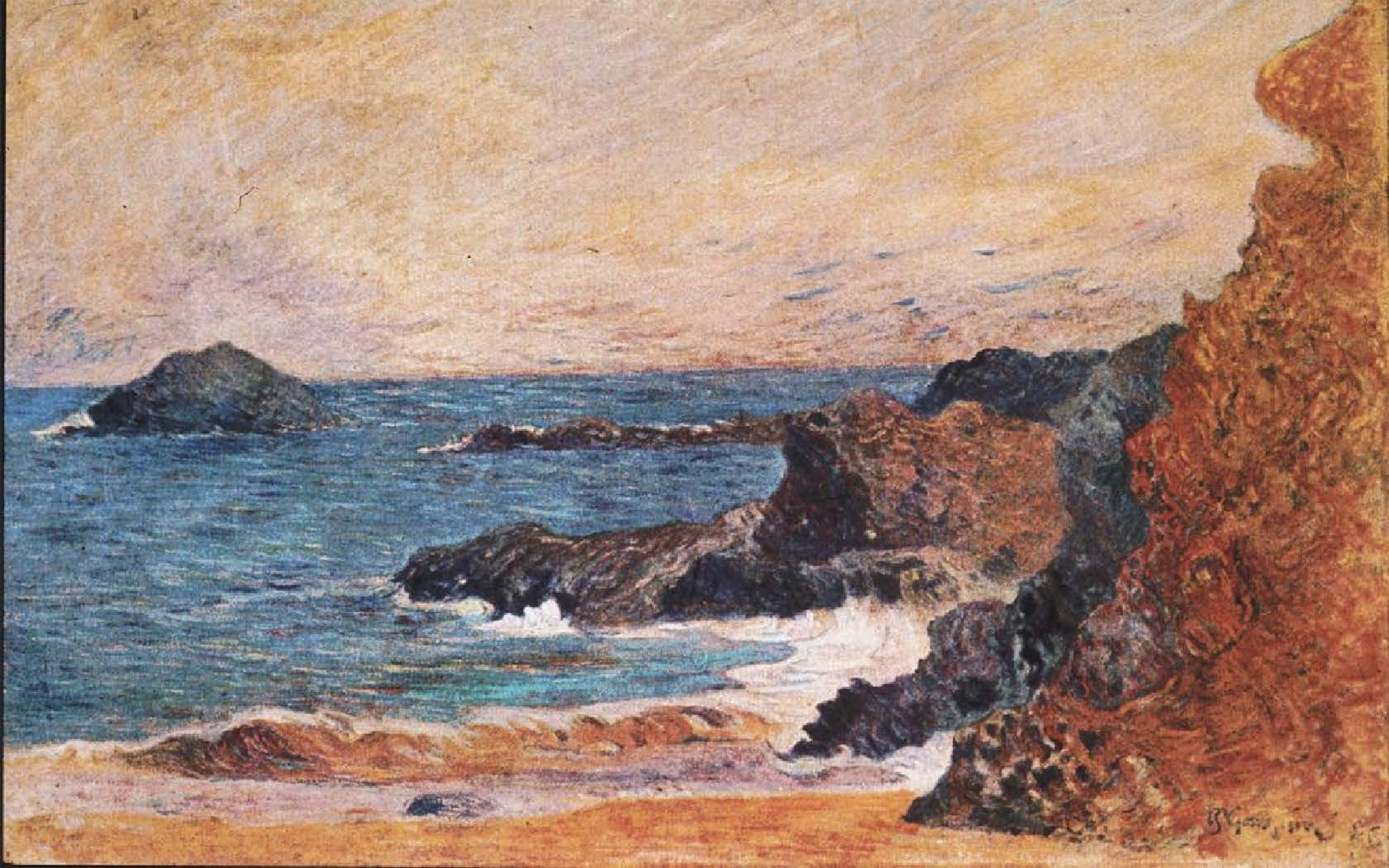

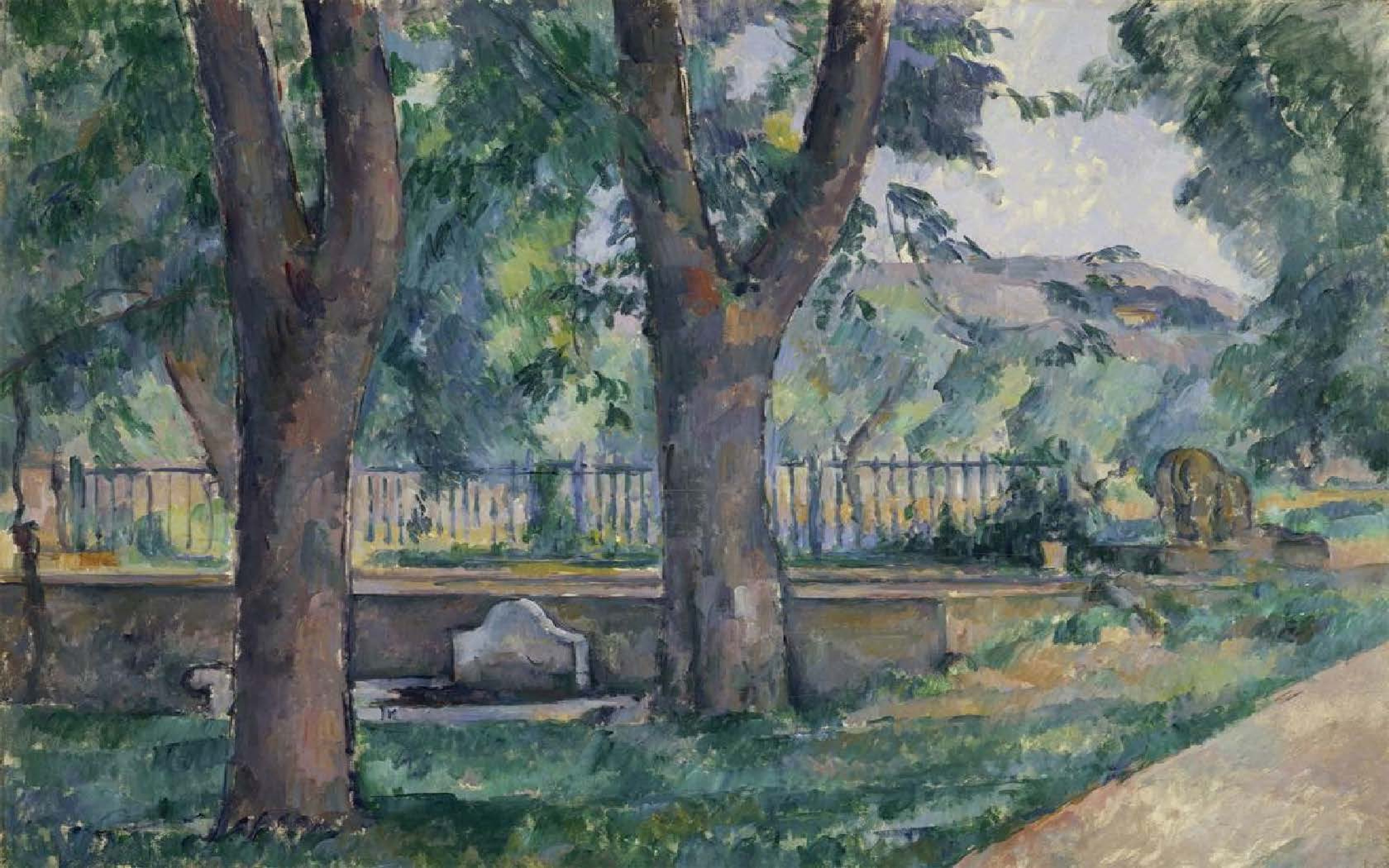

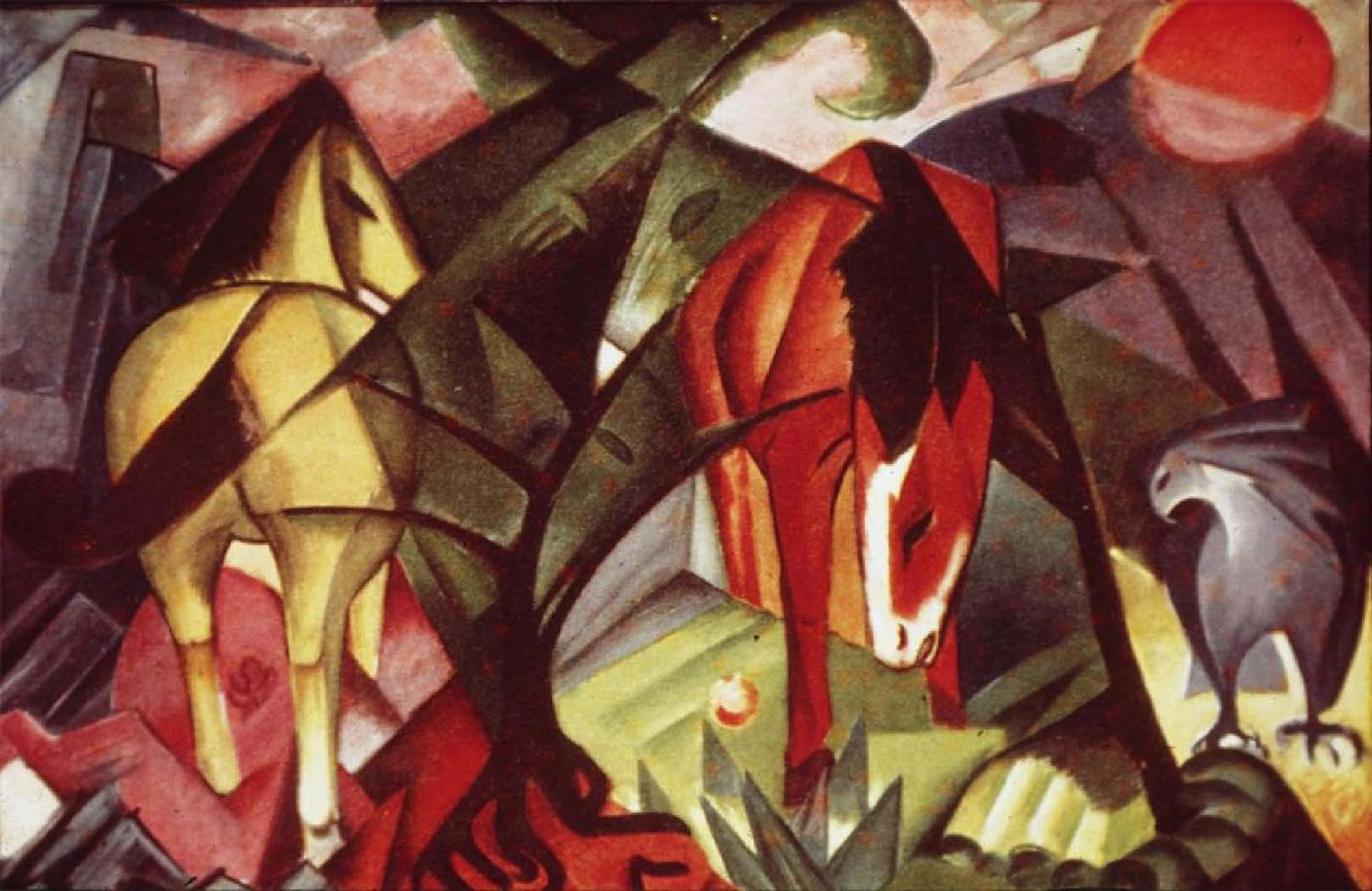

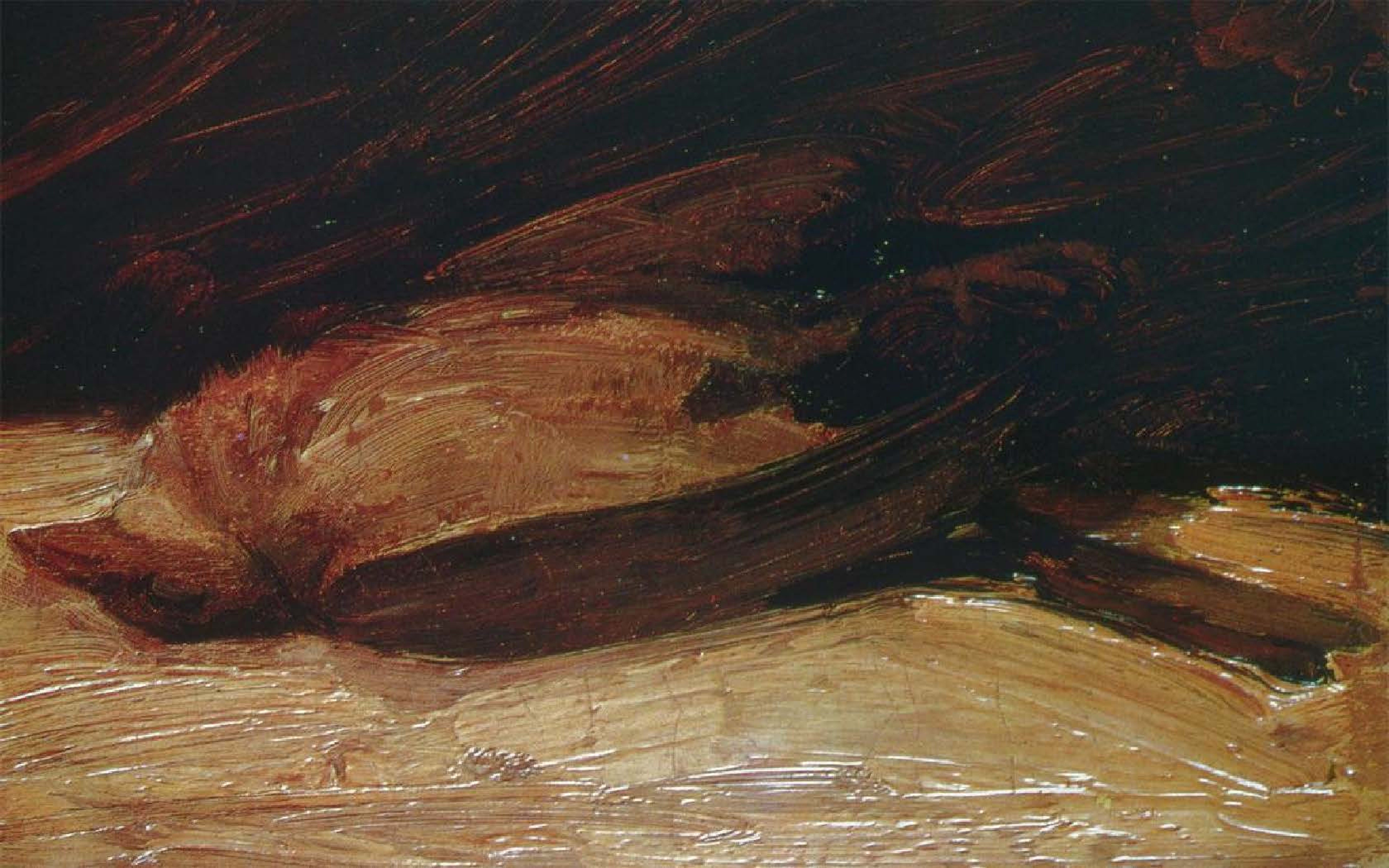

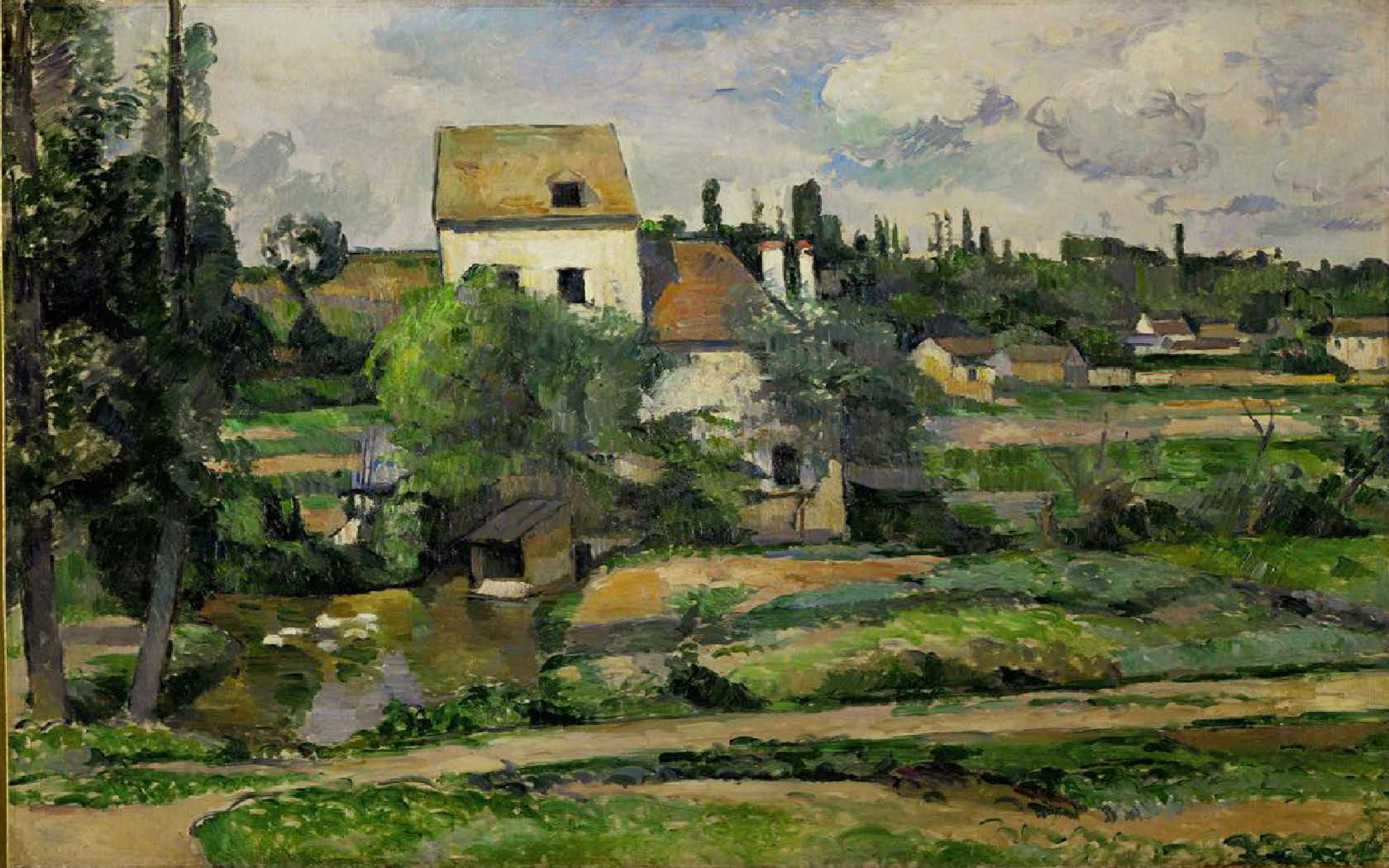

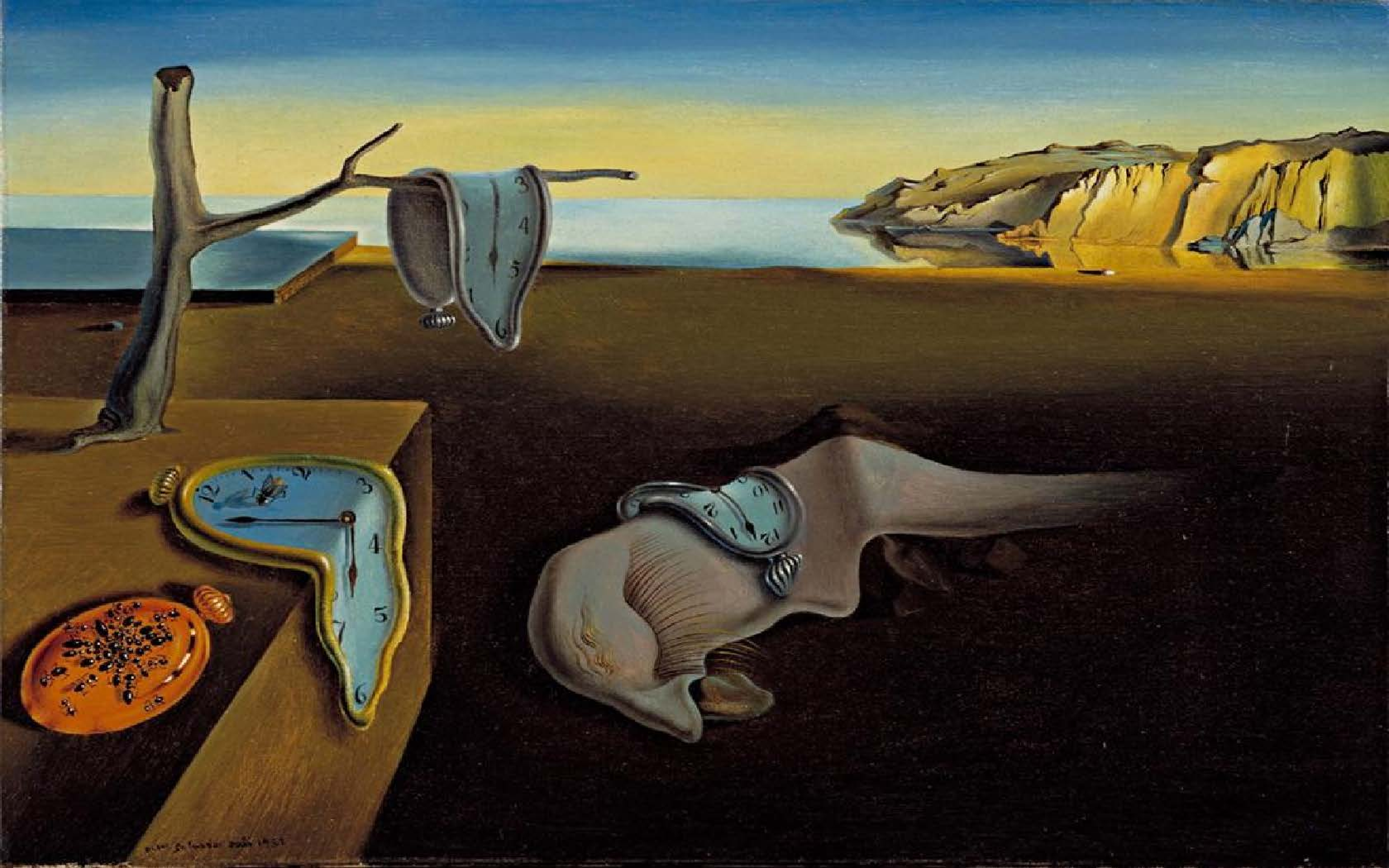

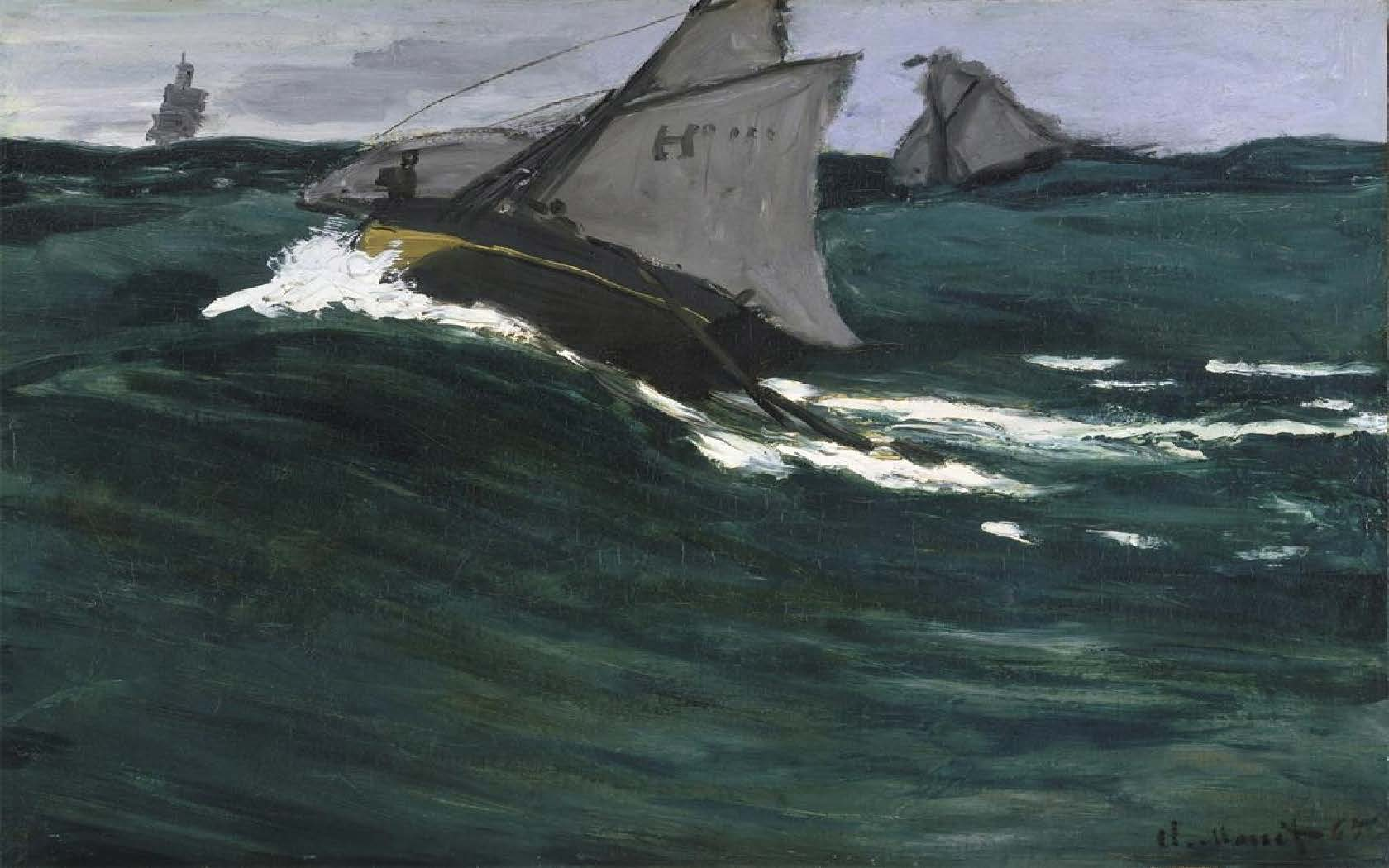

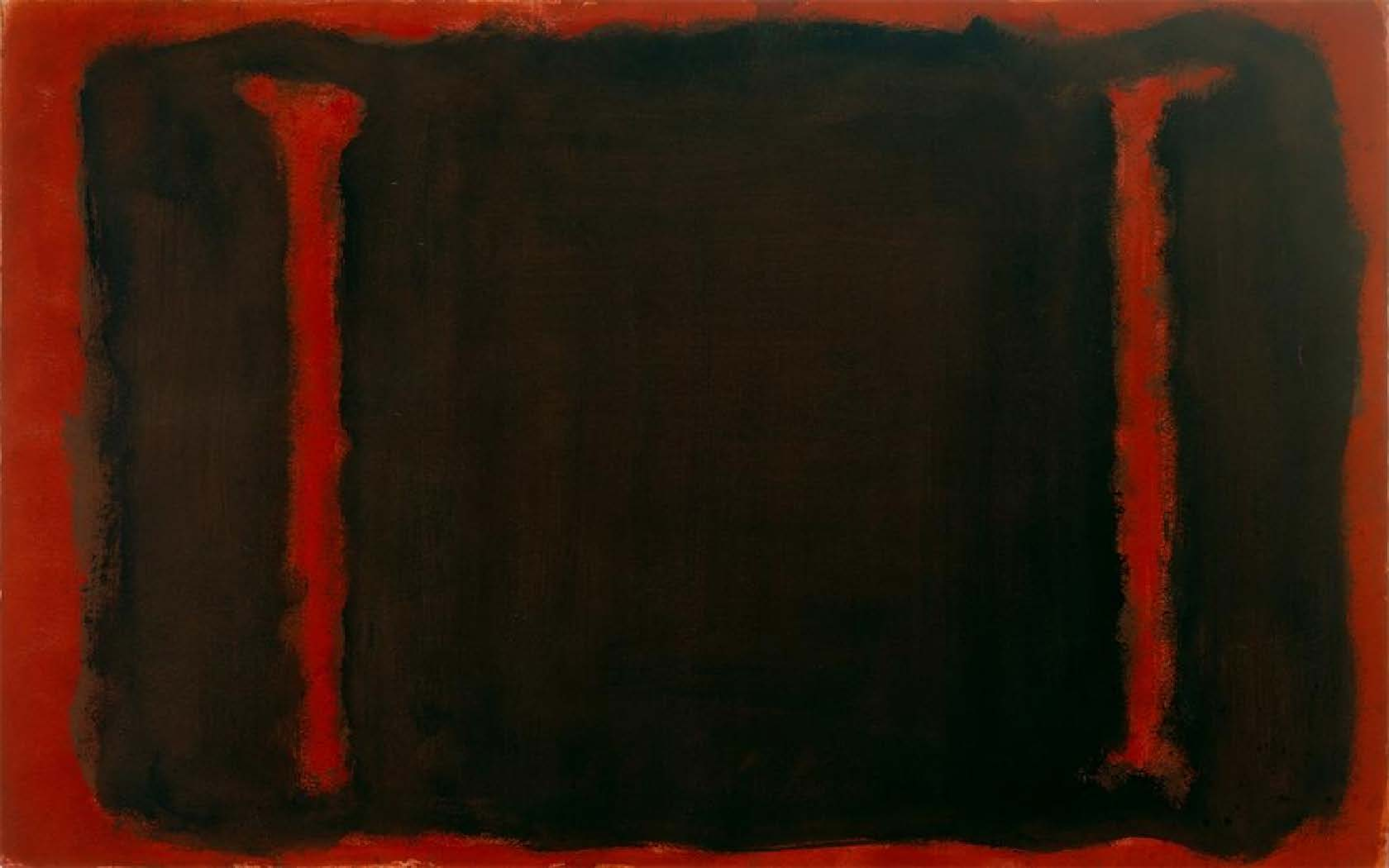

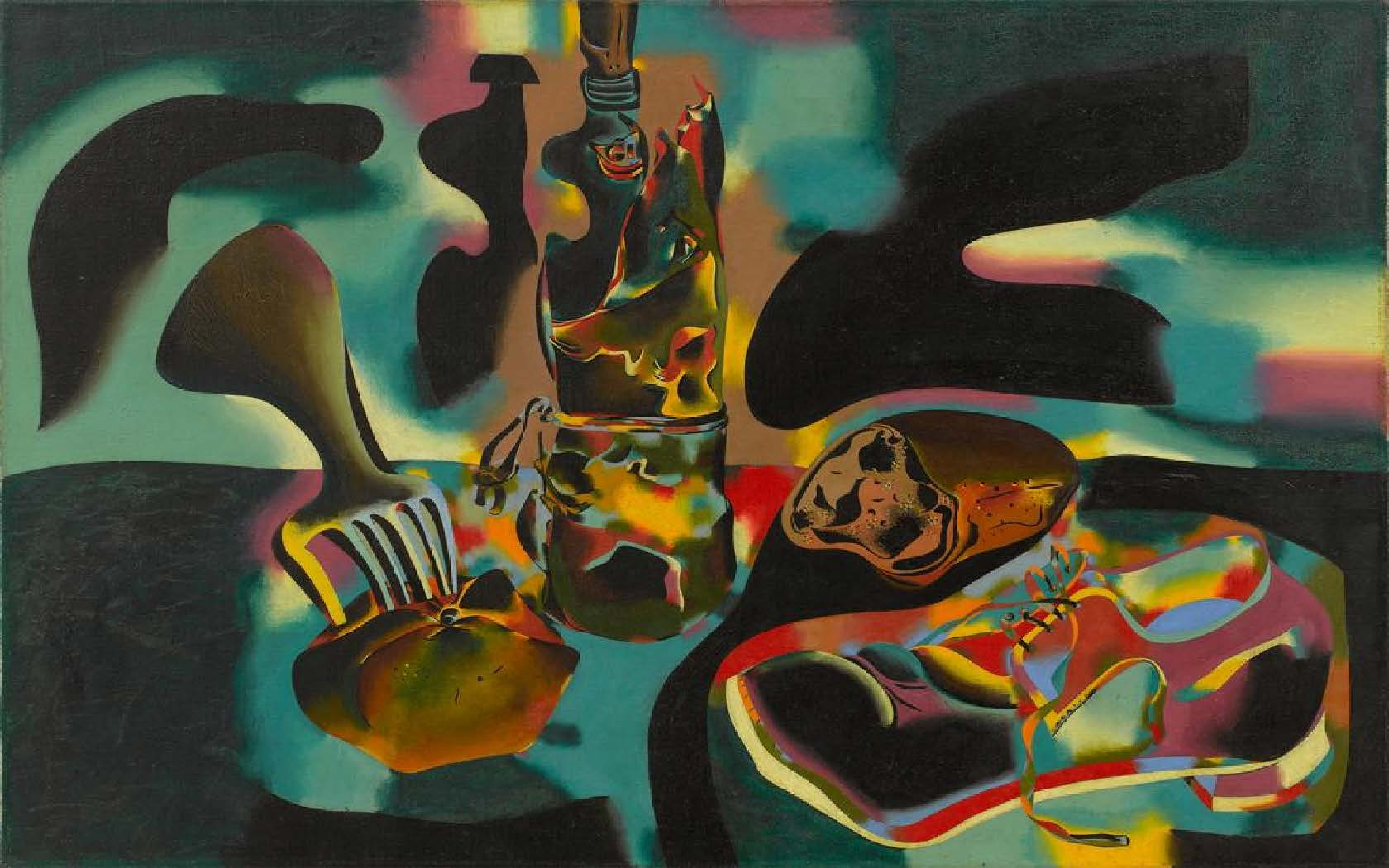

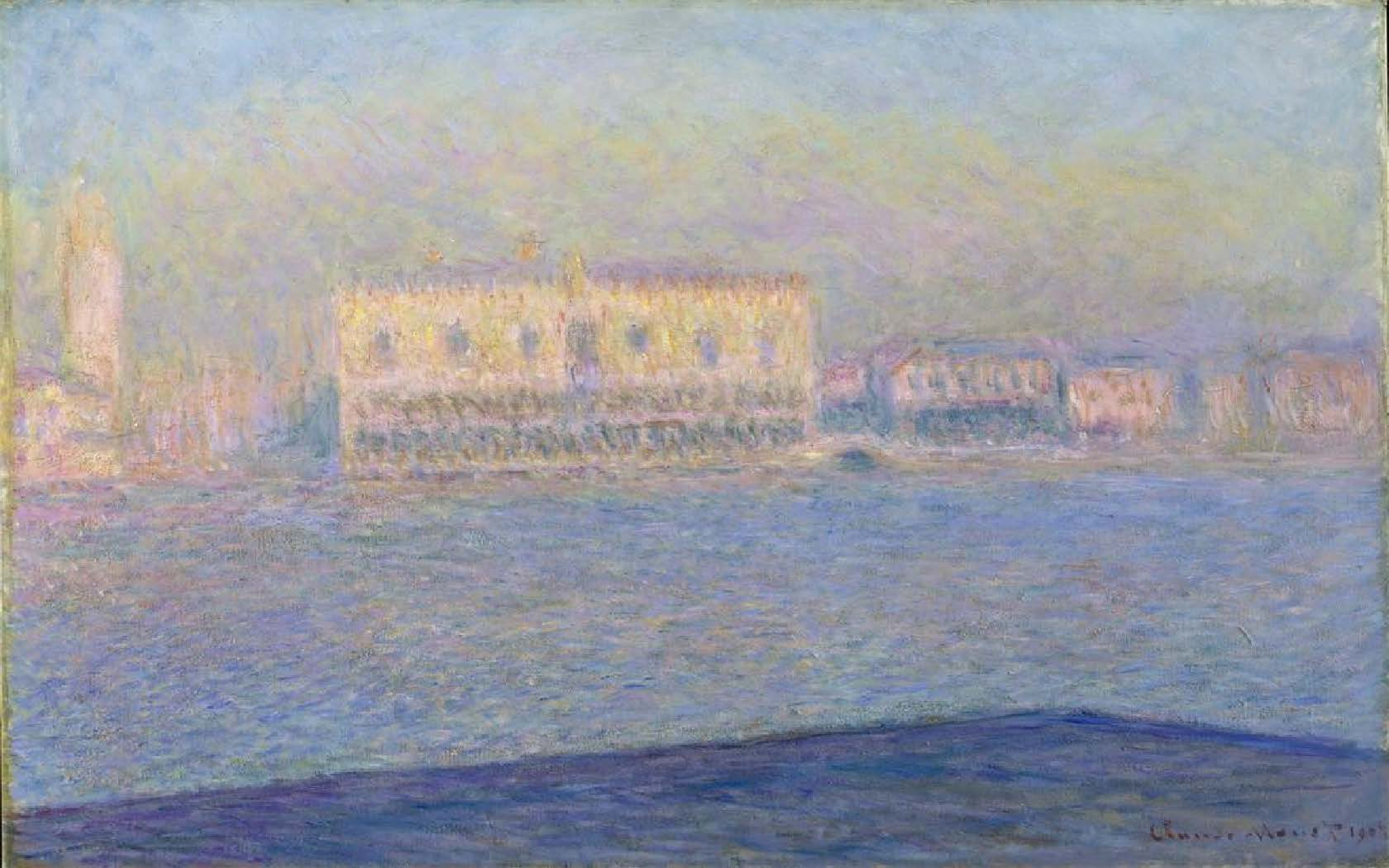

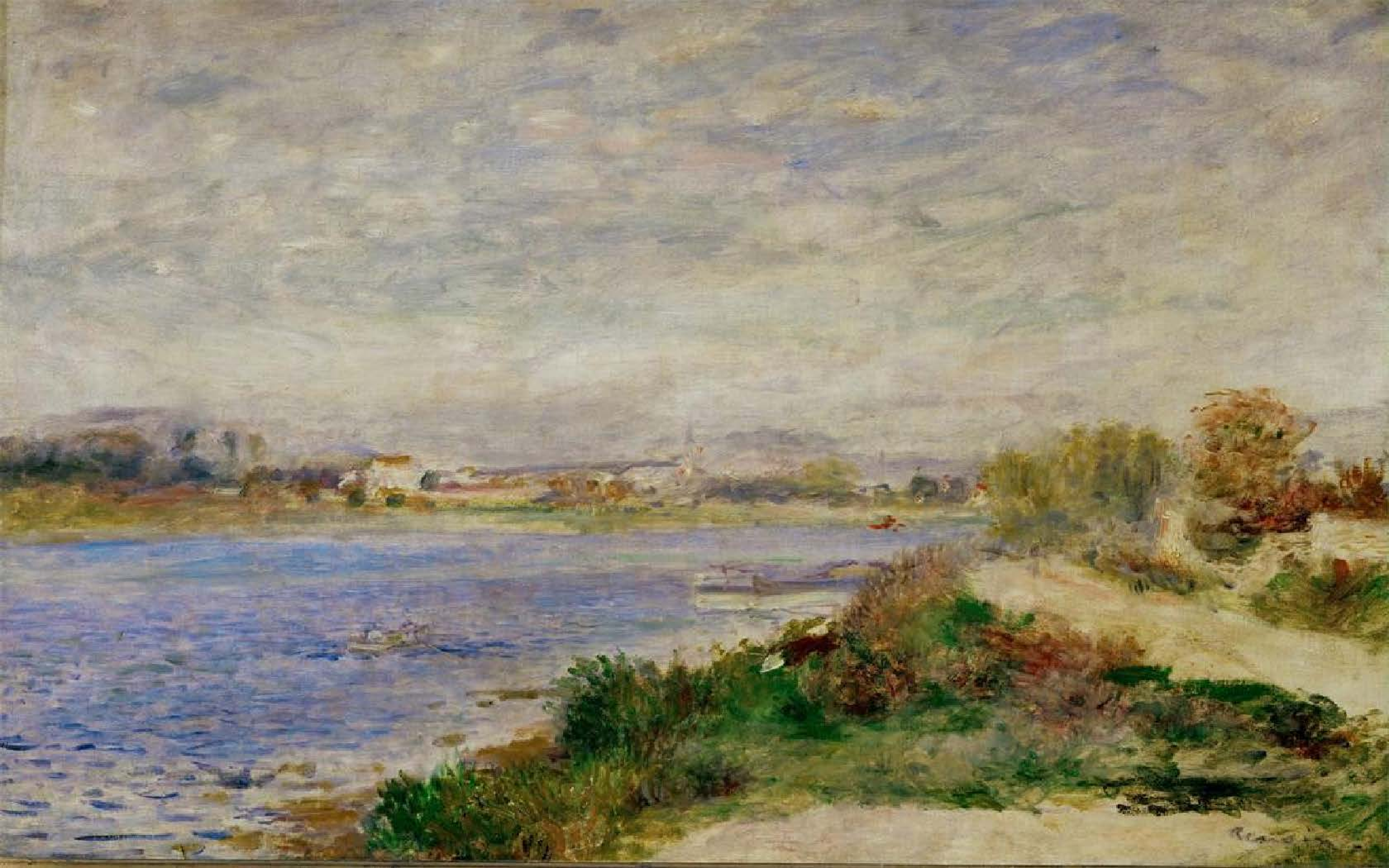

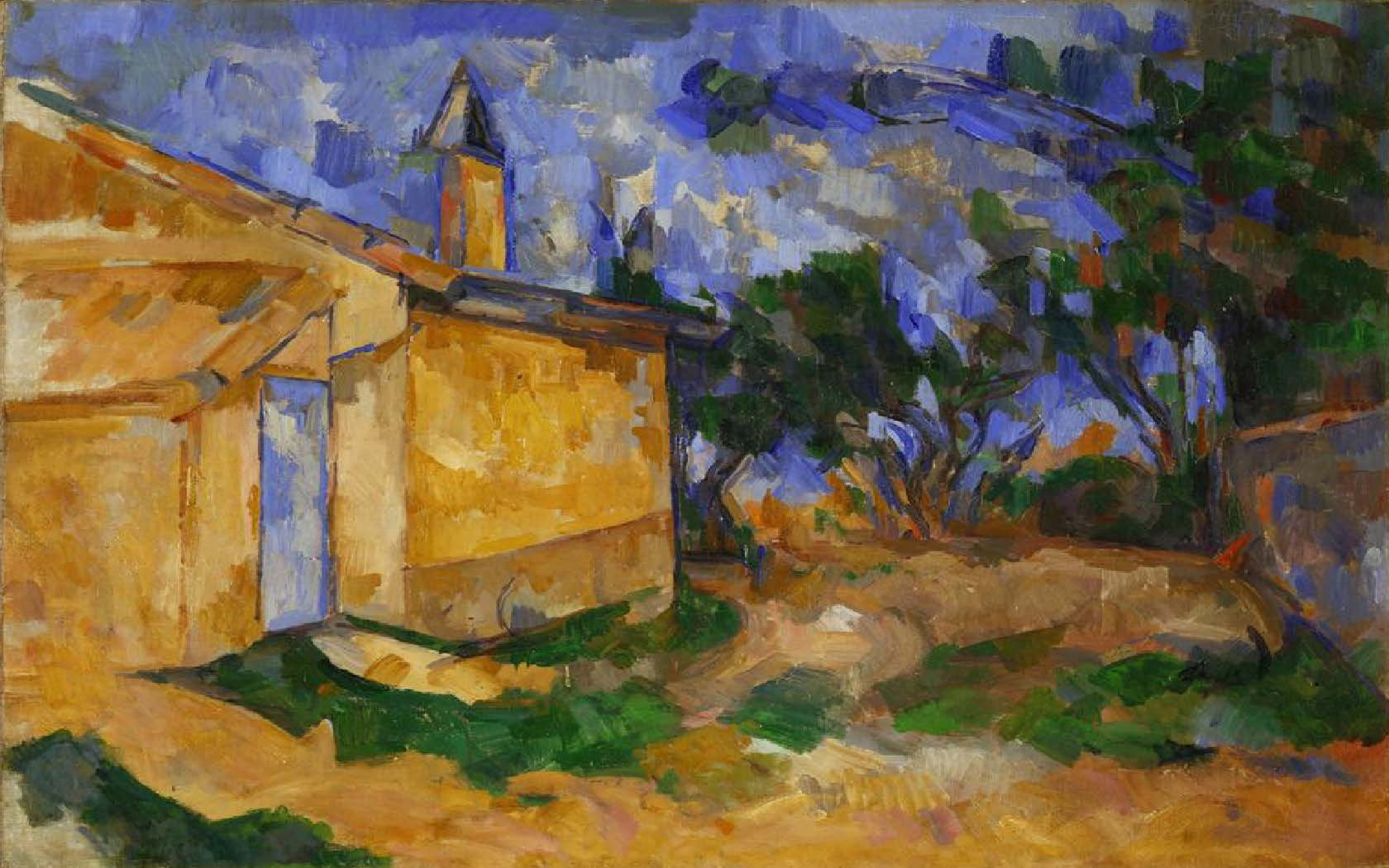

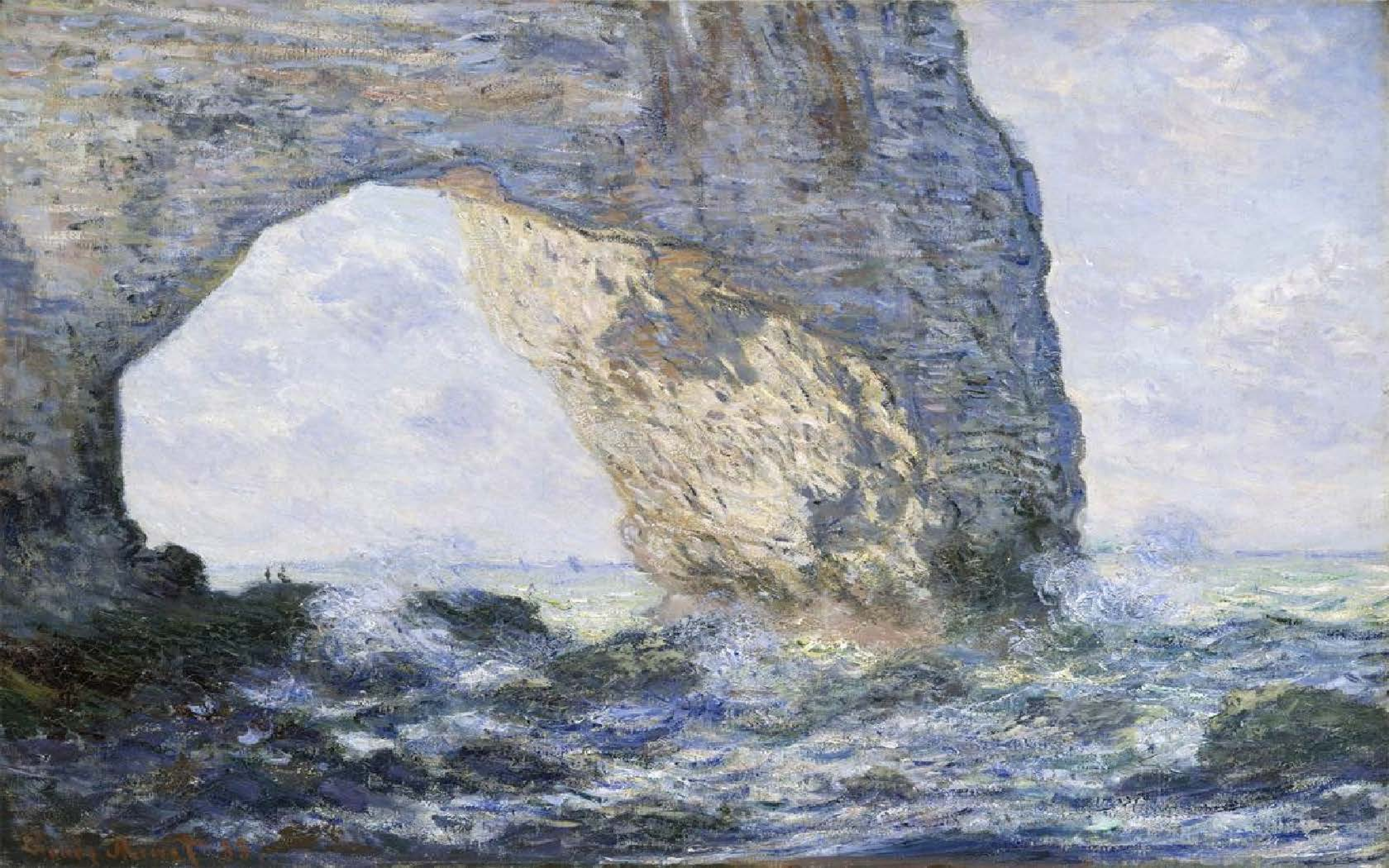

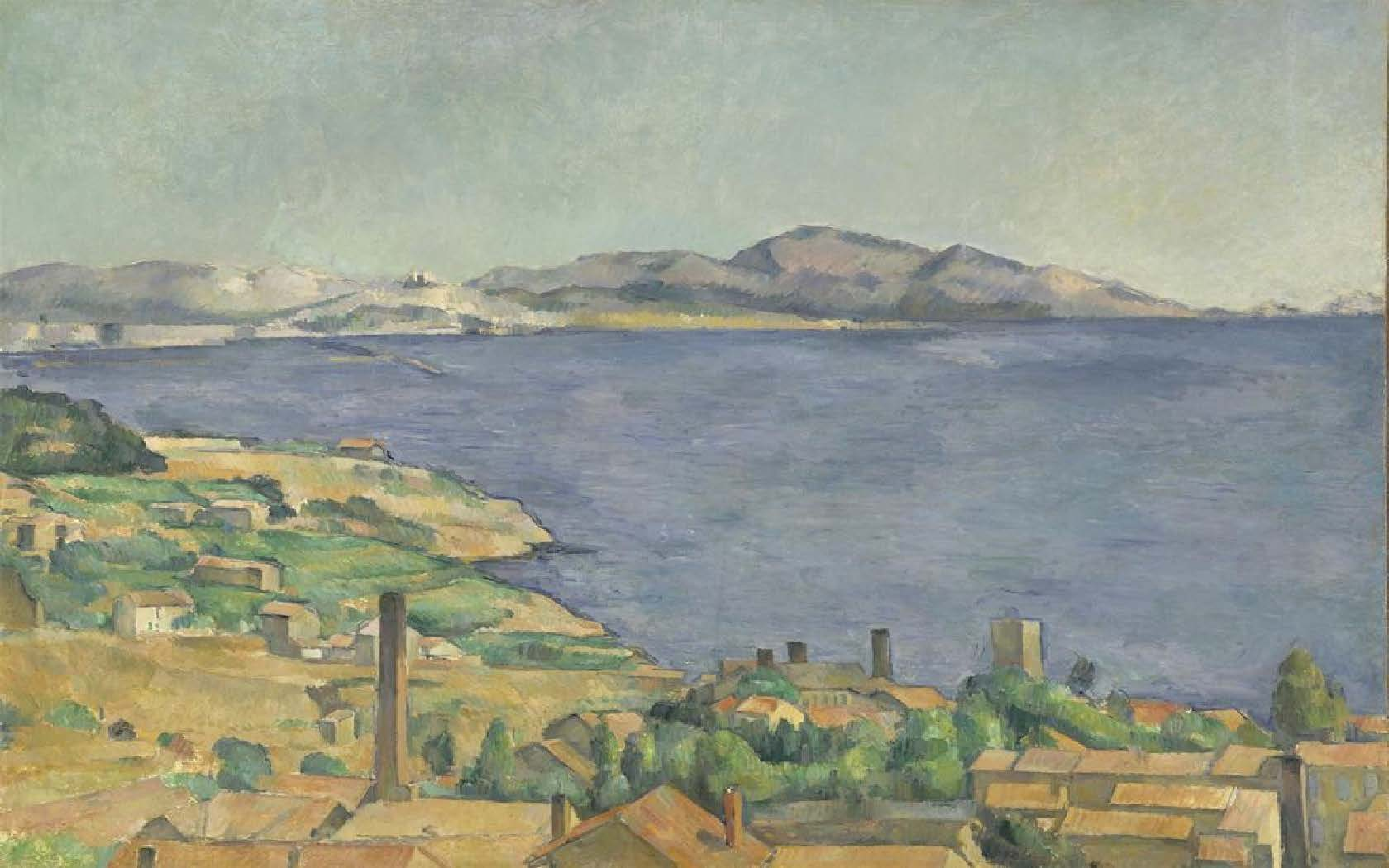

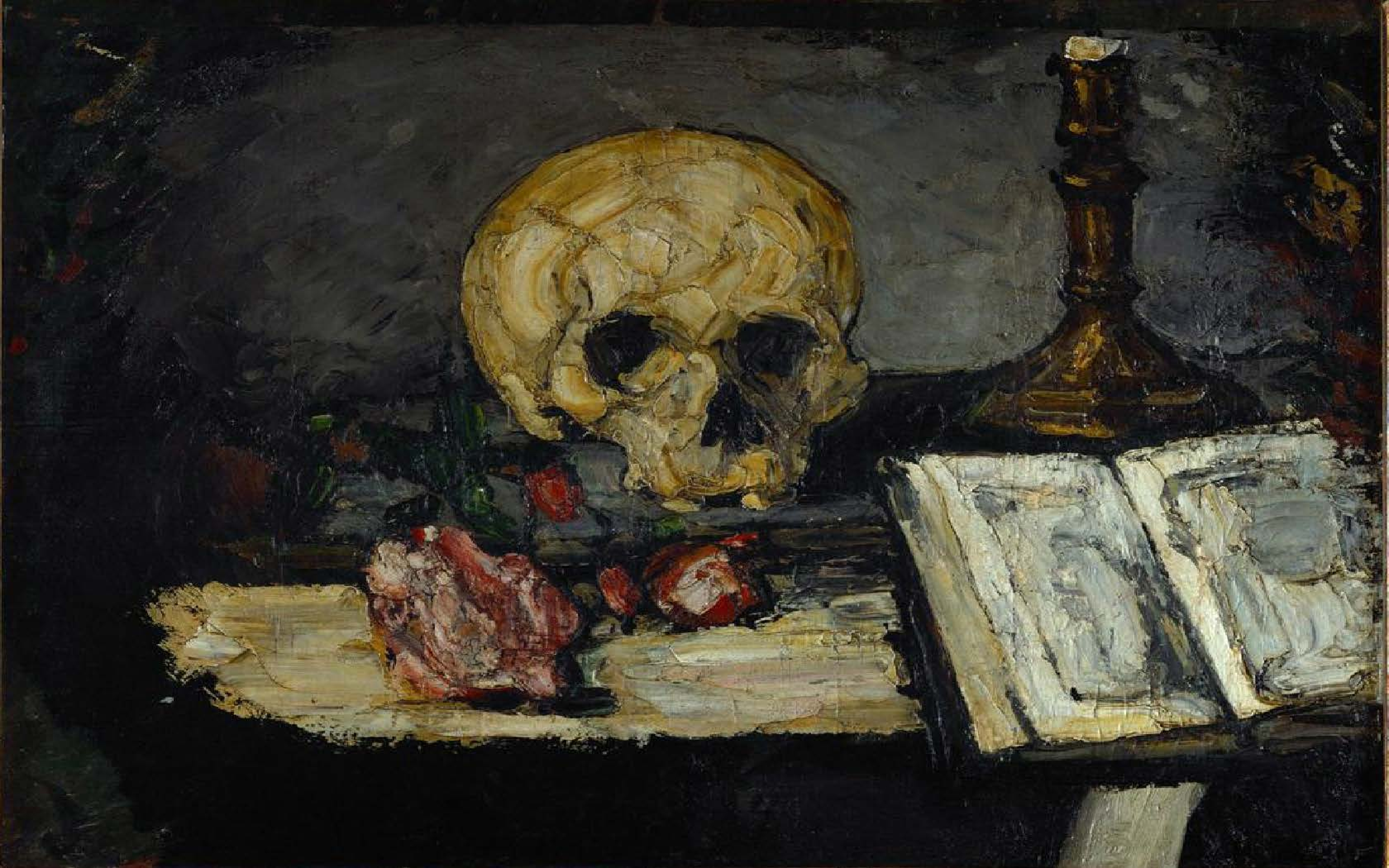

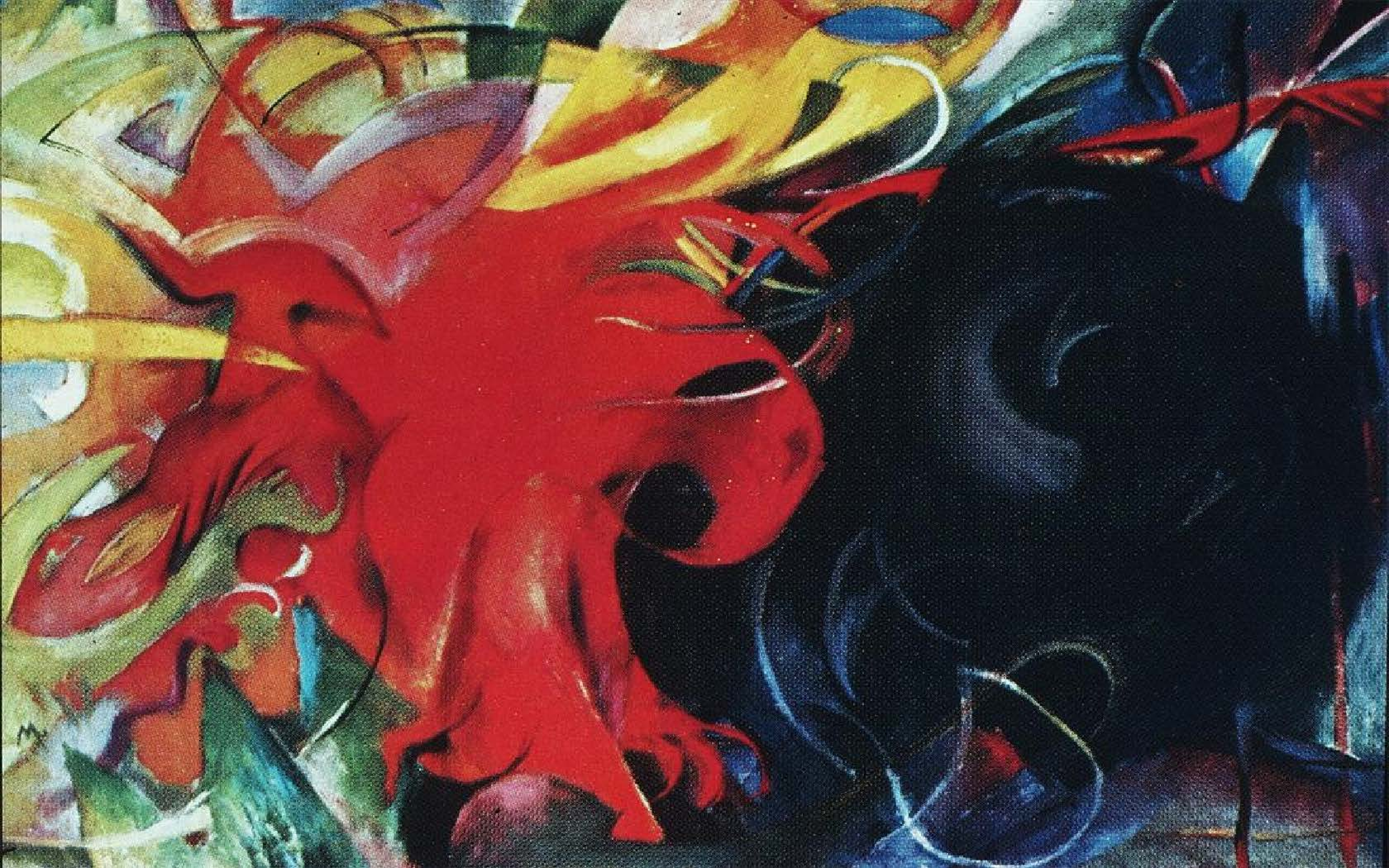

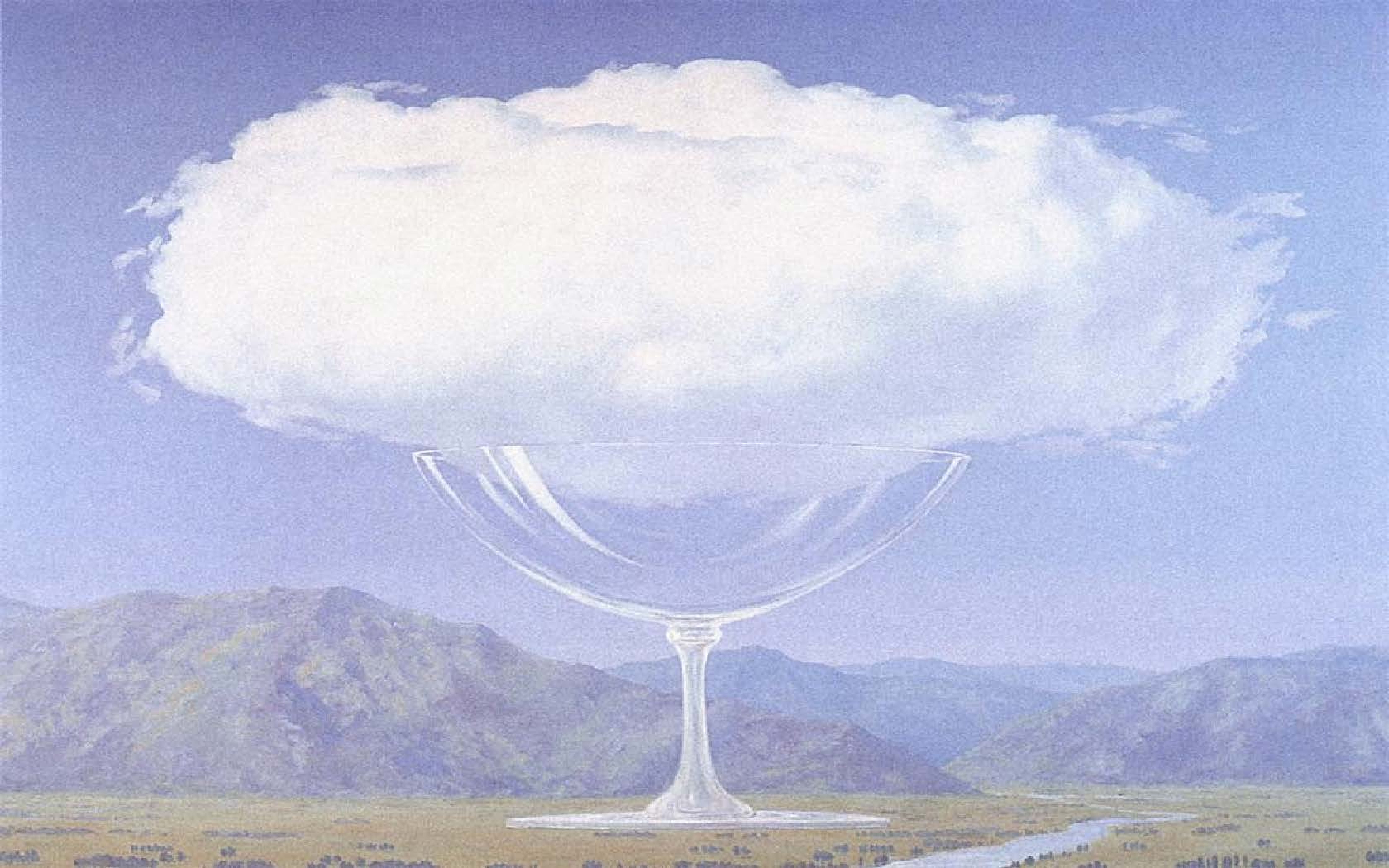

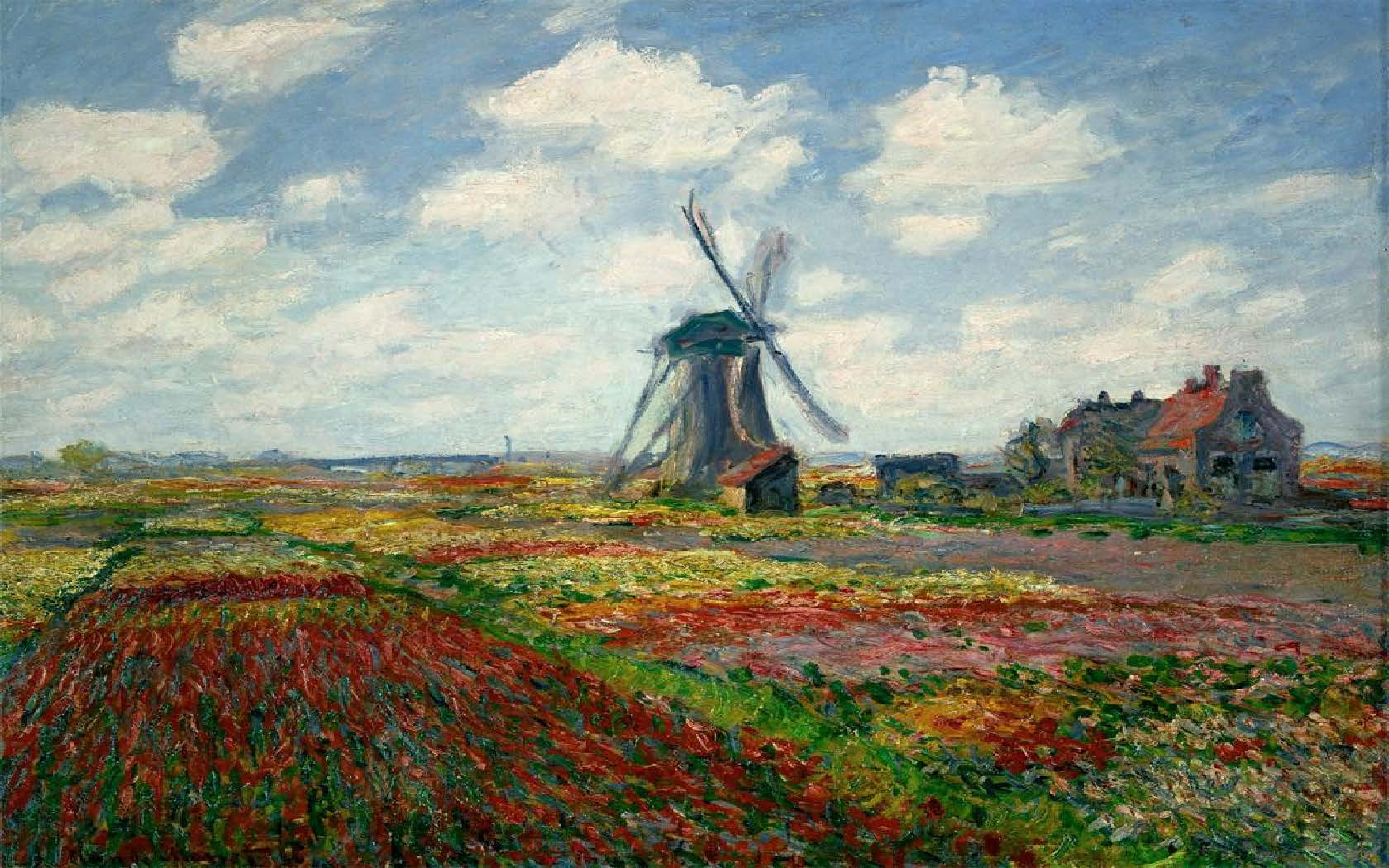

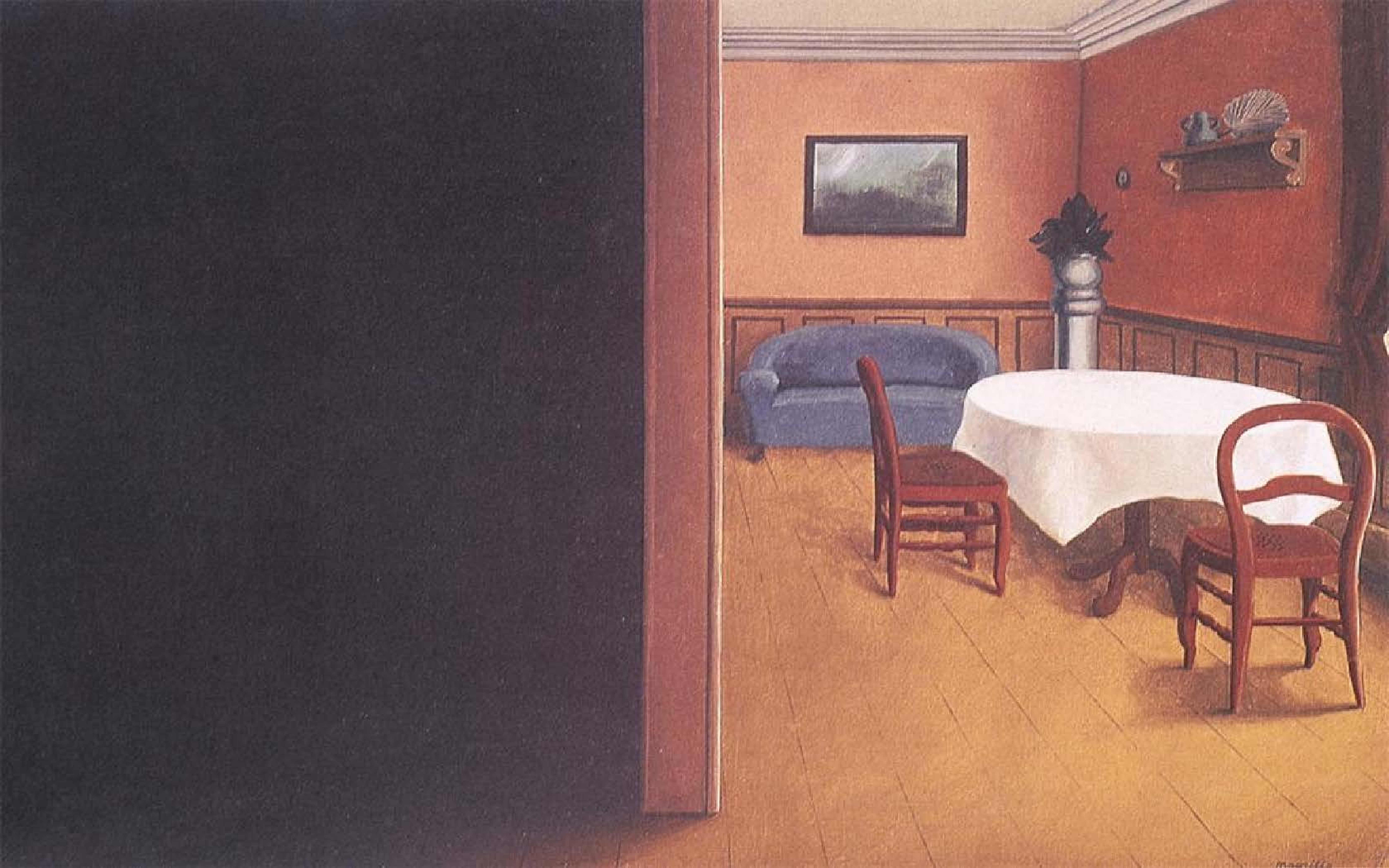

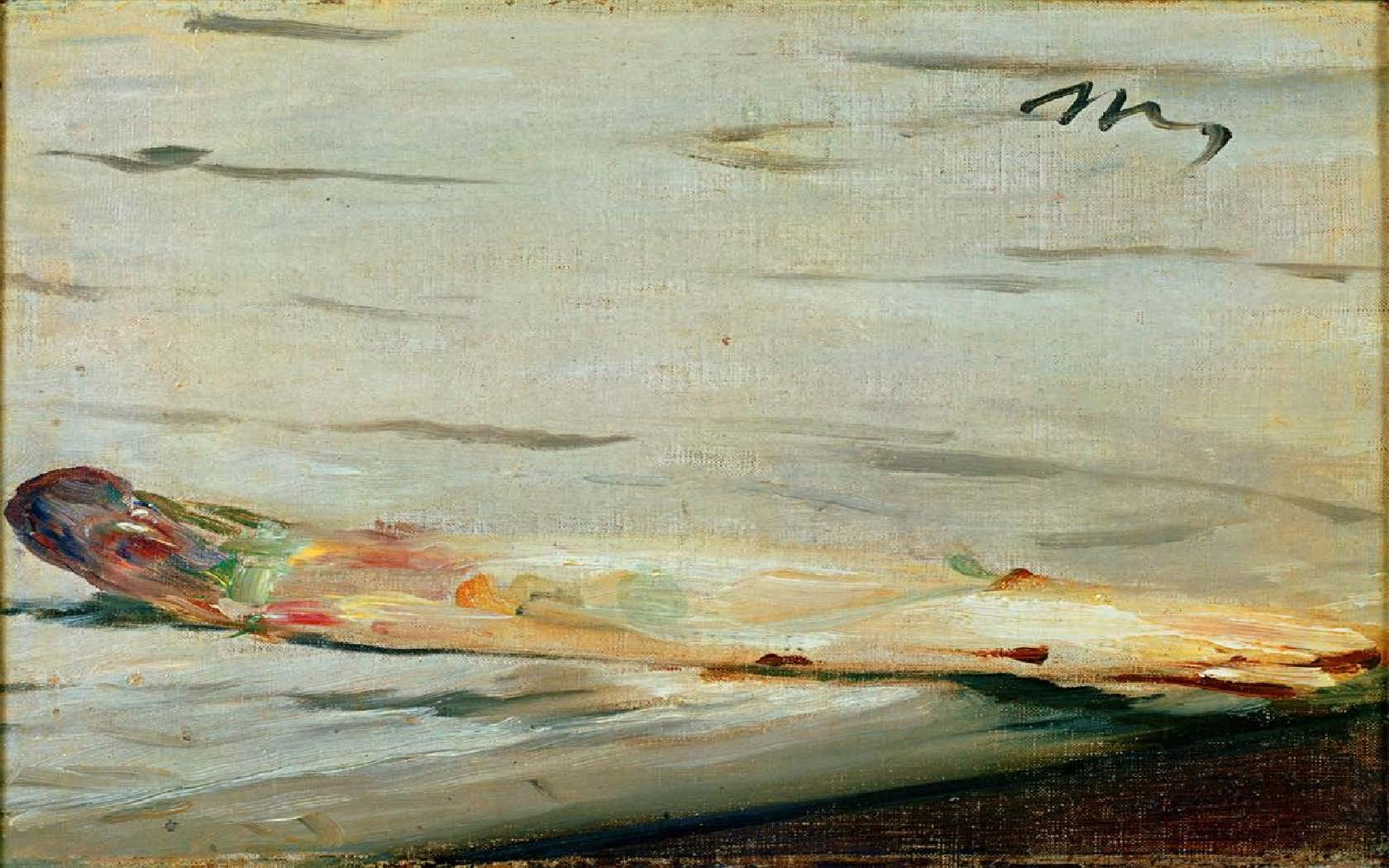

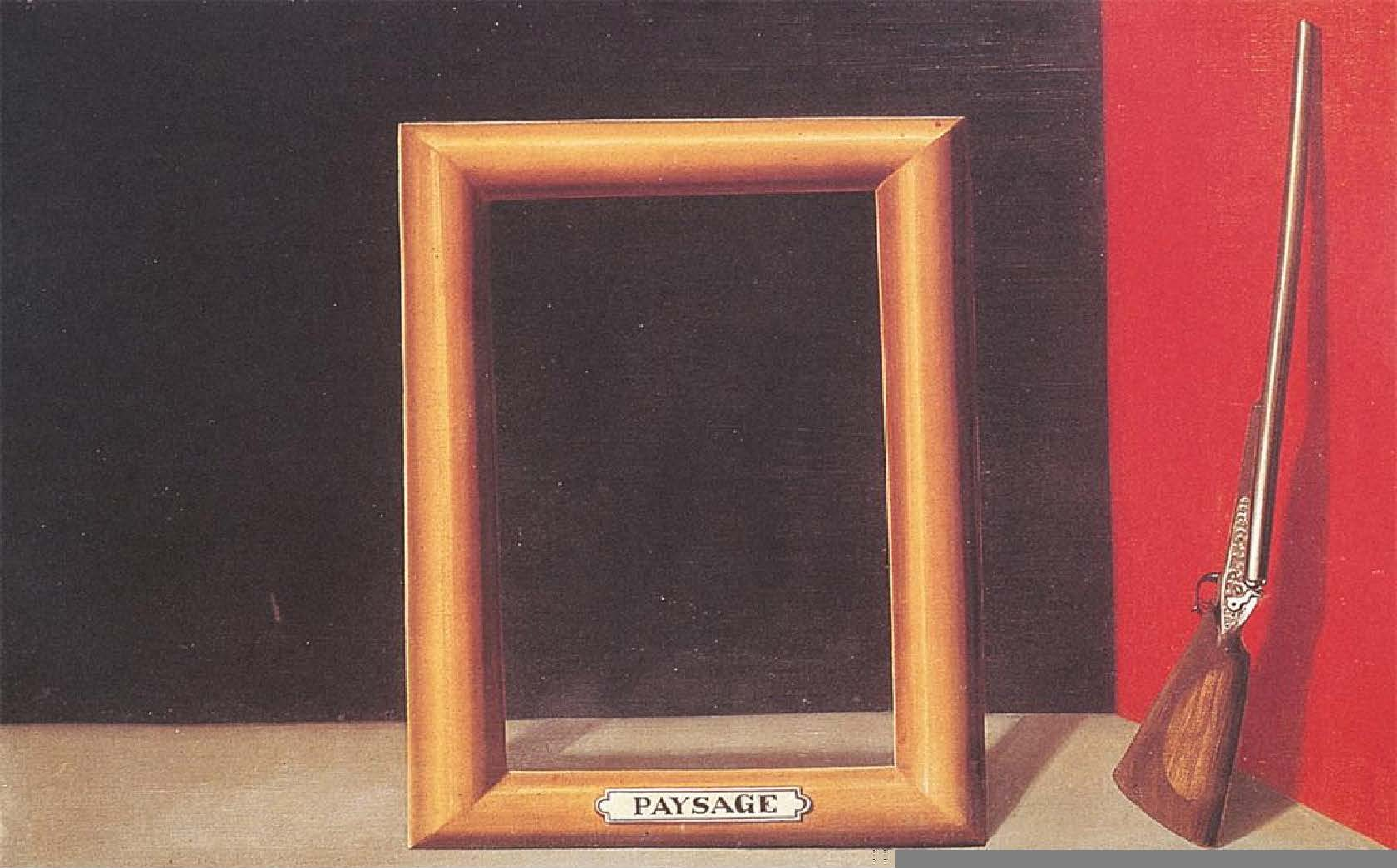
PAYSAGE

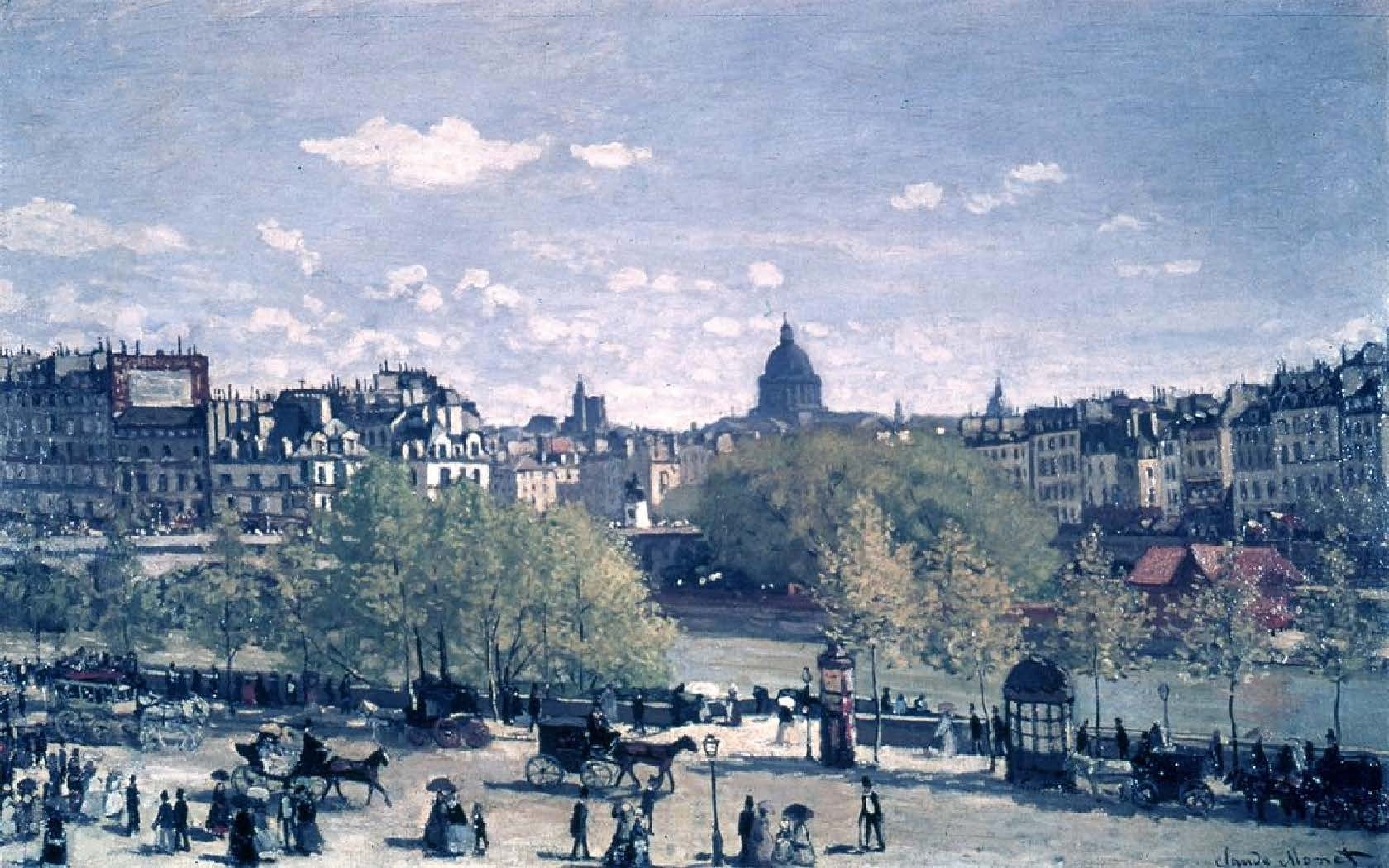
Claude Monet

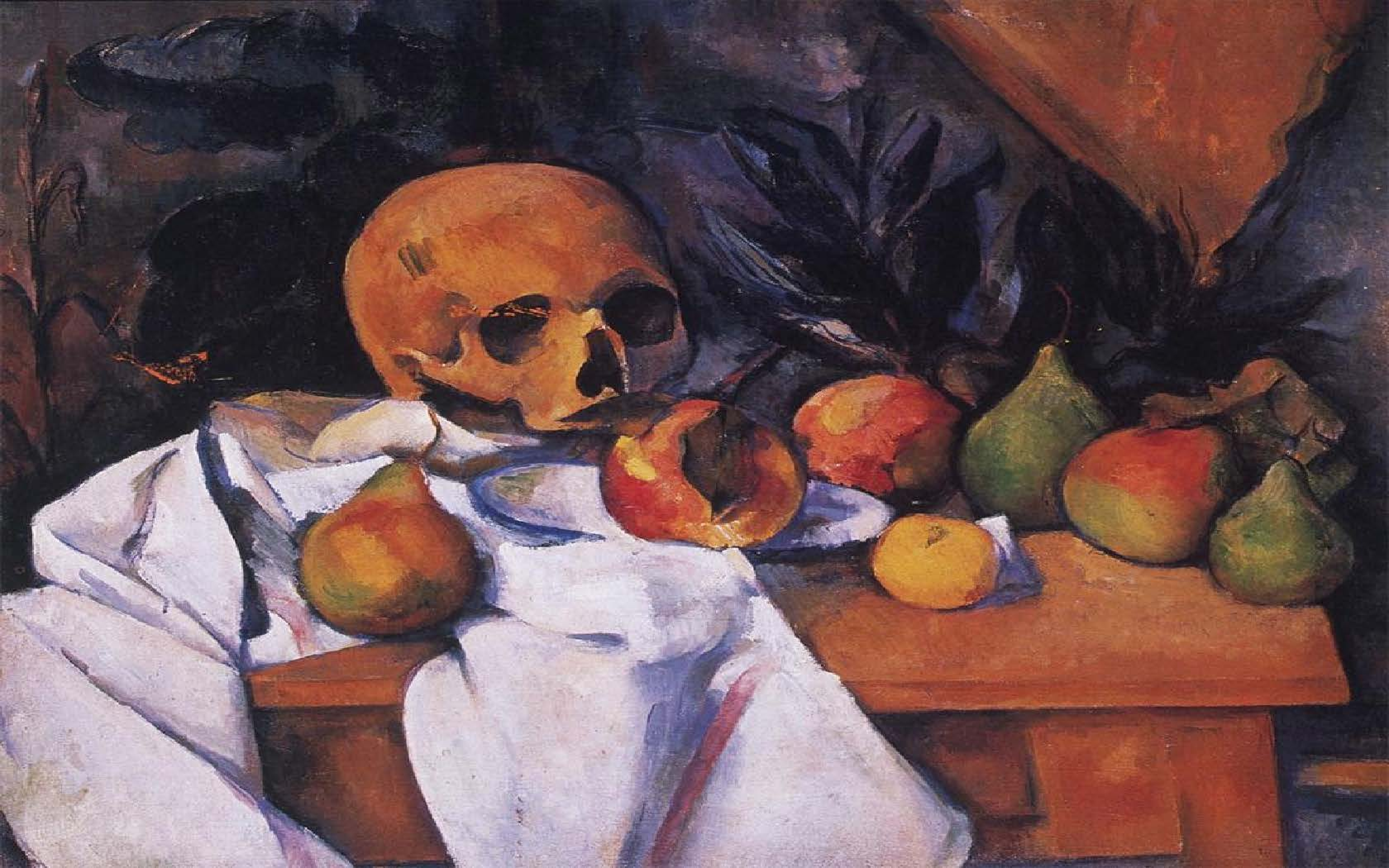

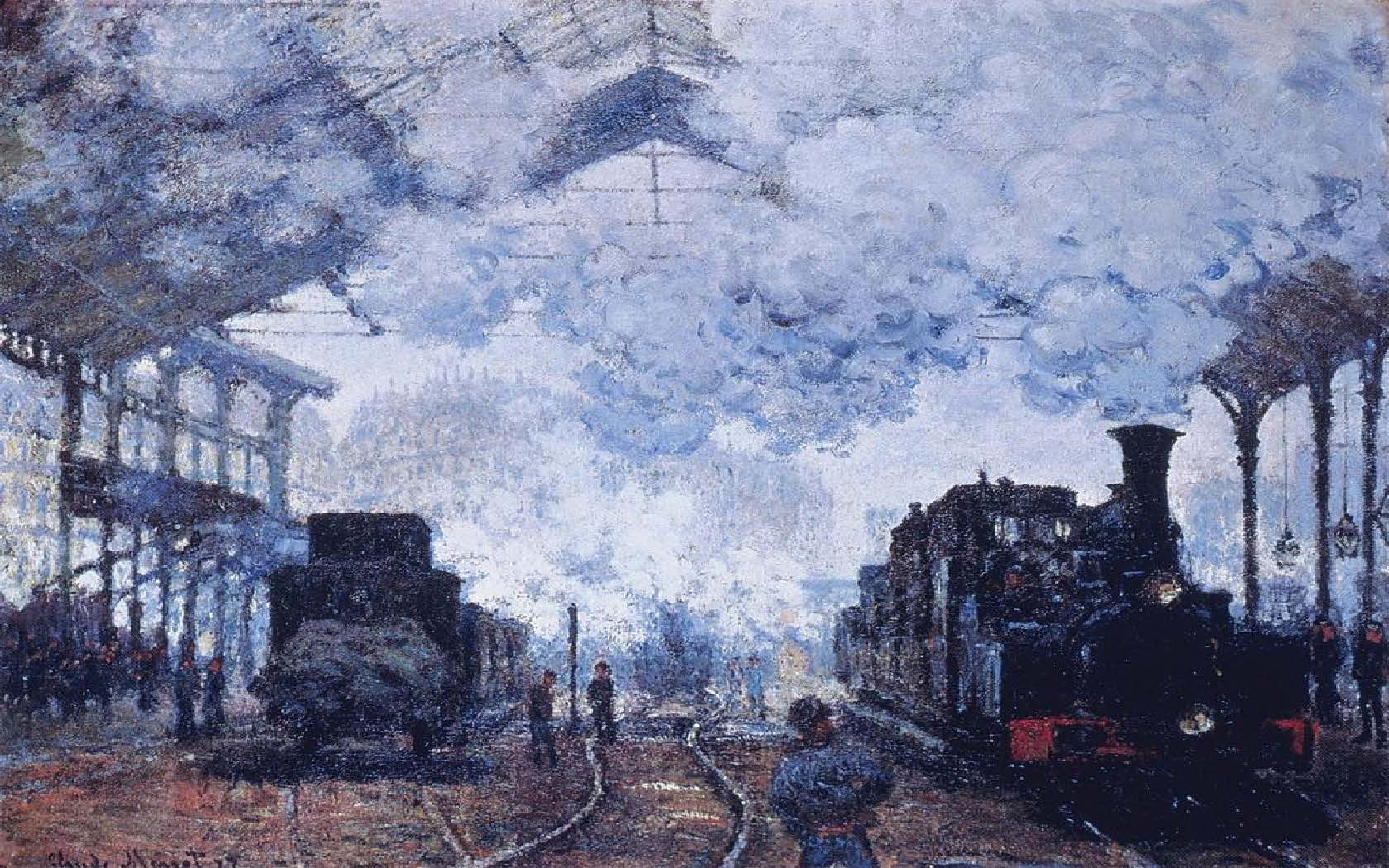

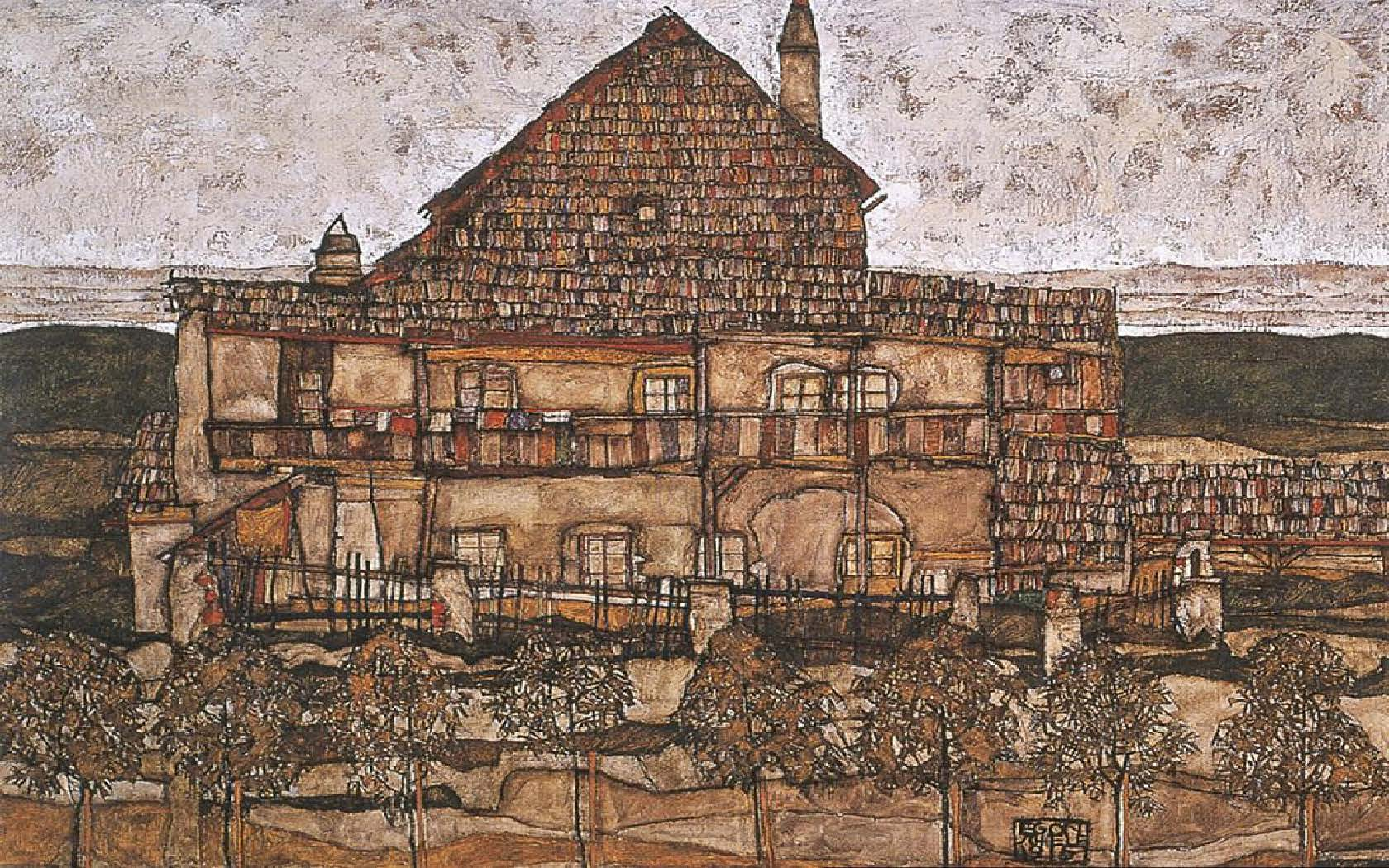

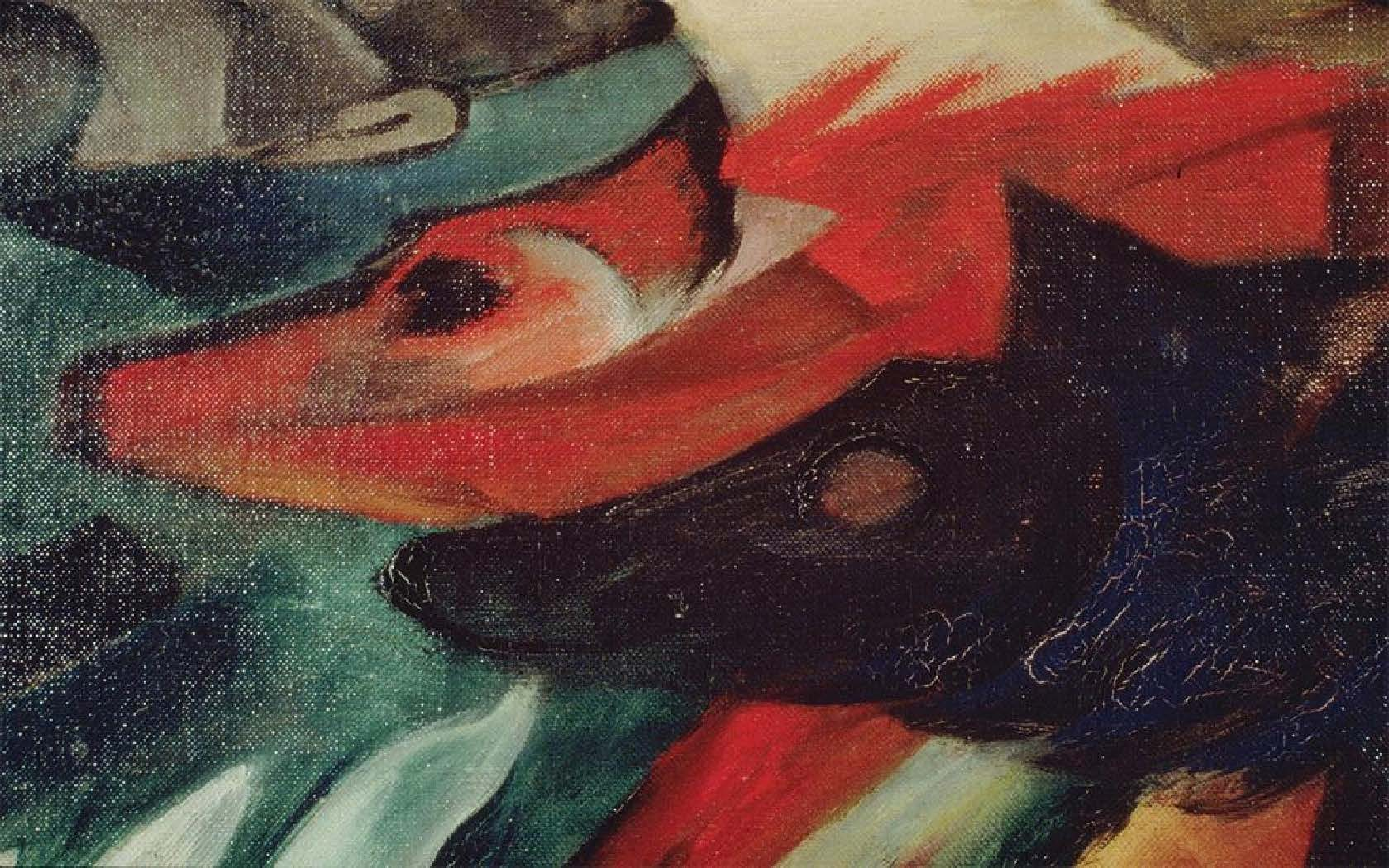

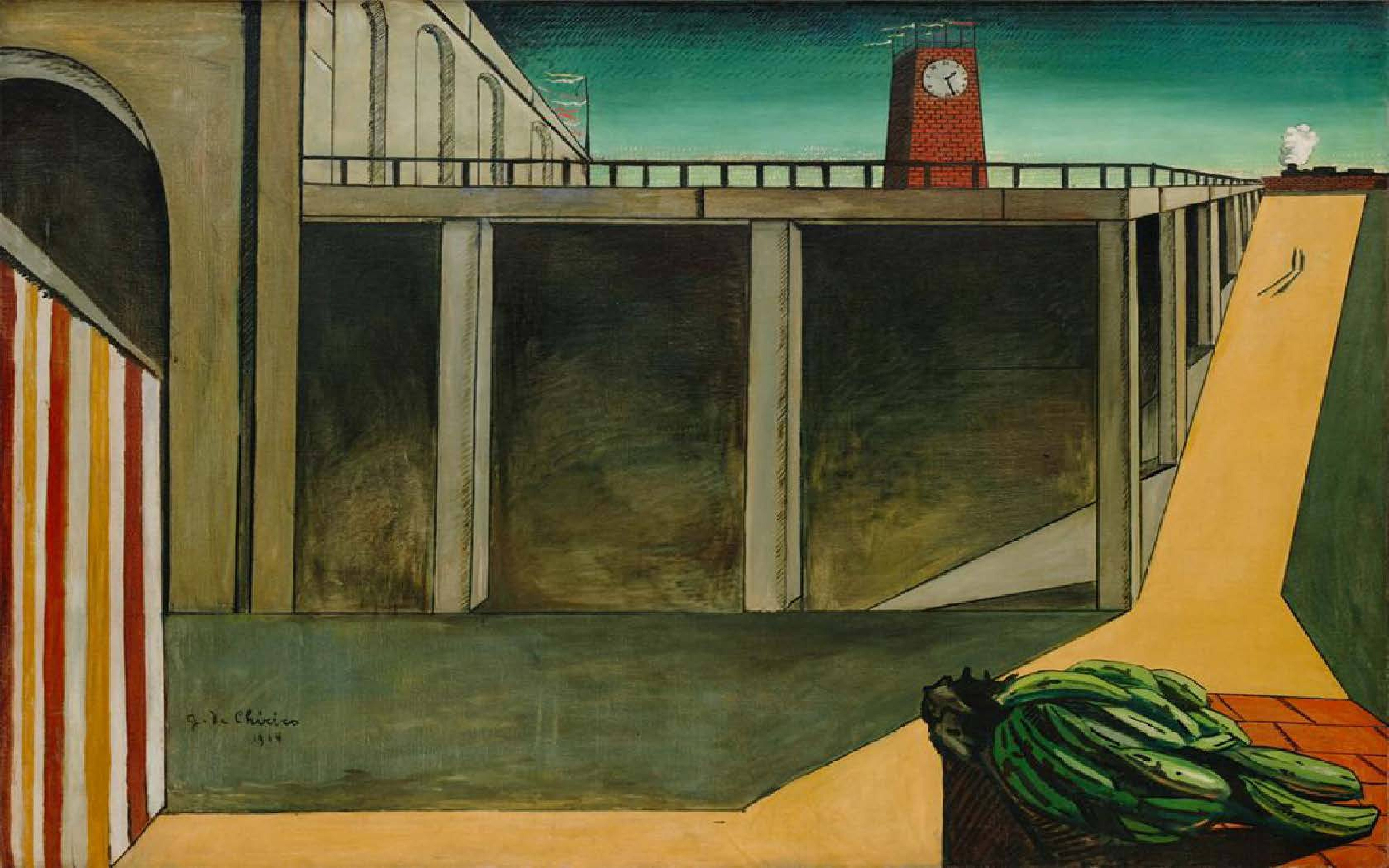
G. de Chirico
1914

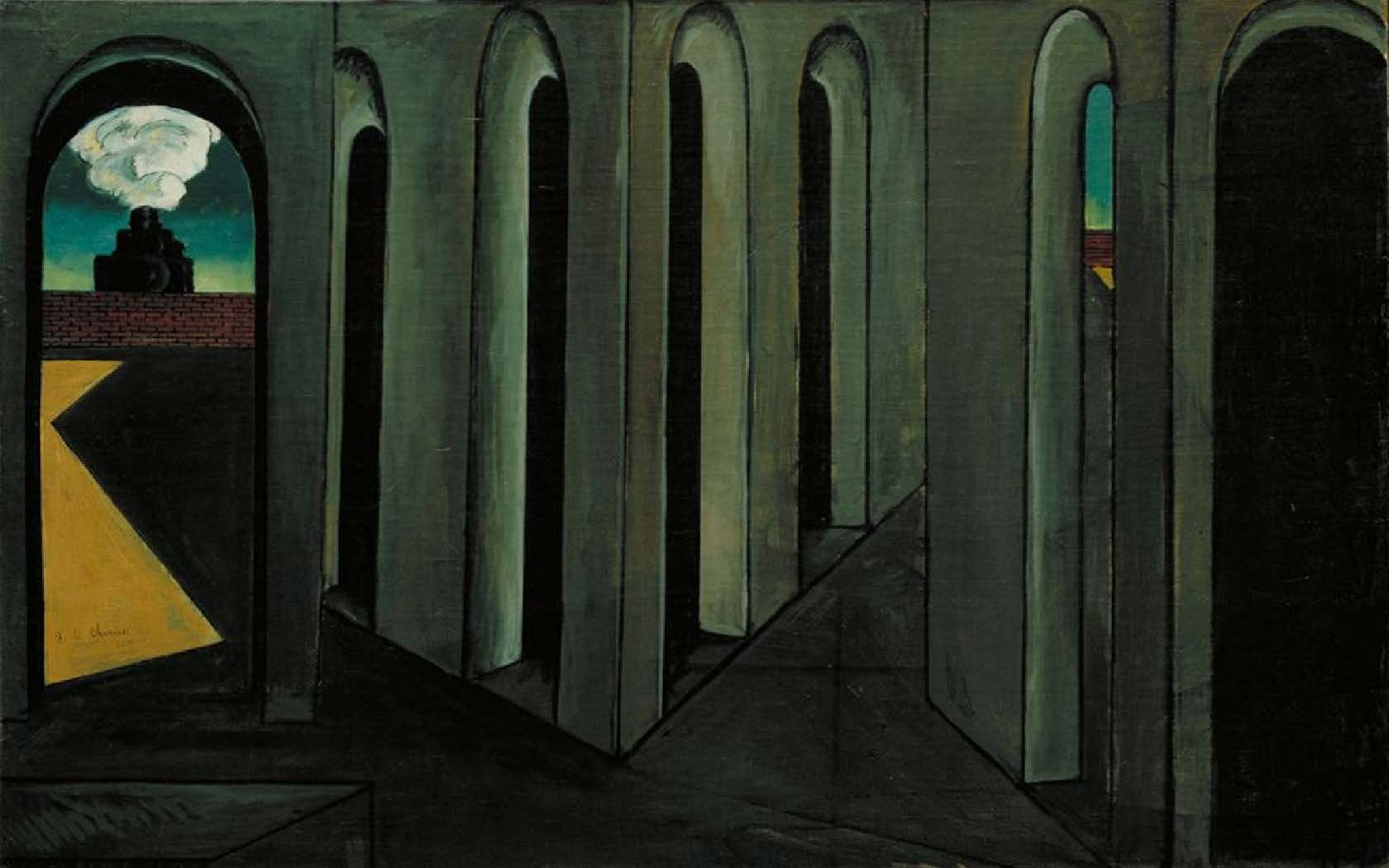

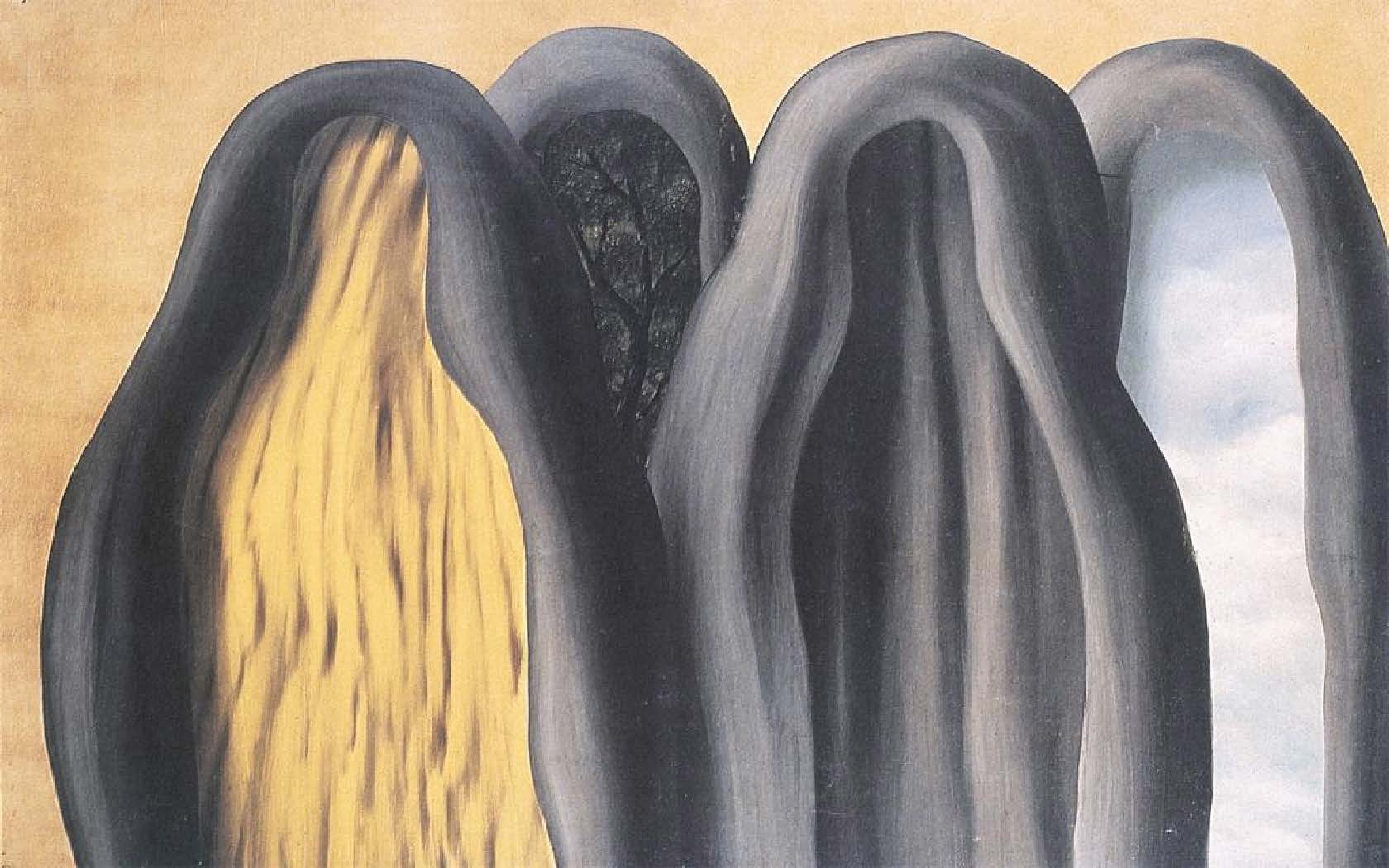

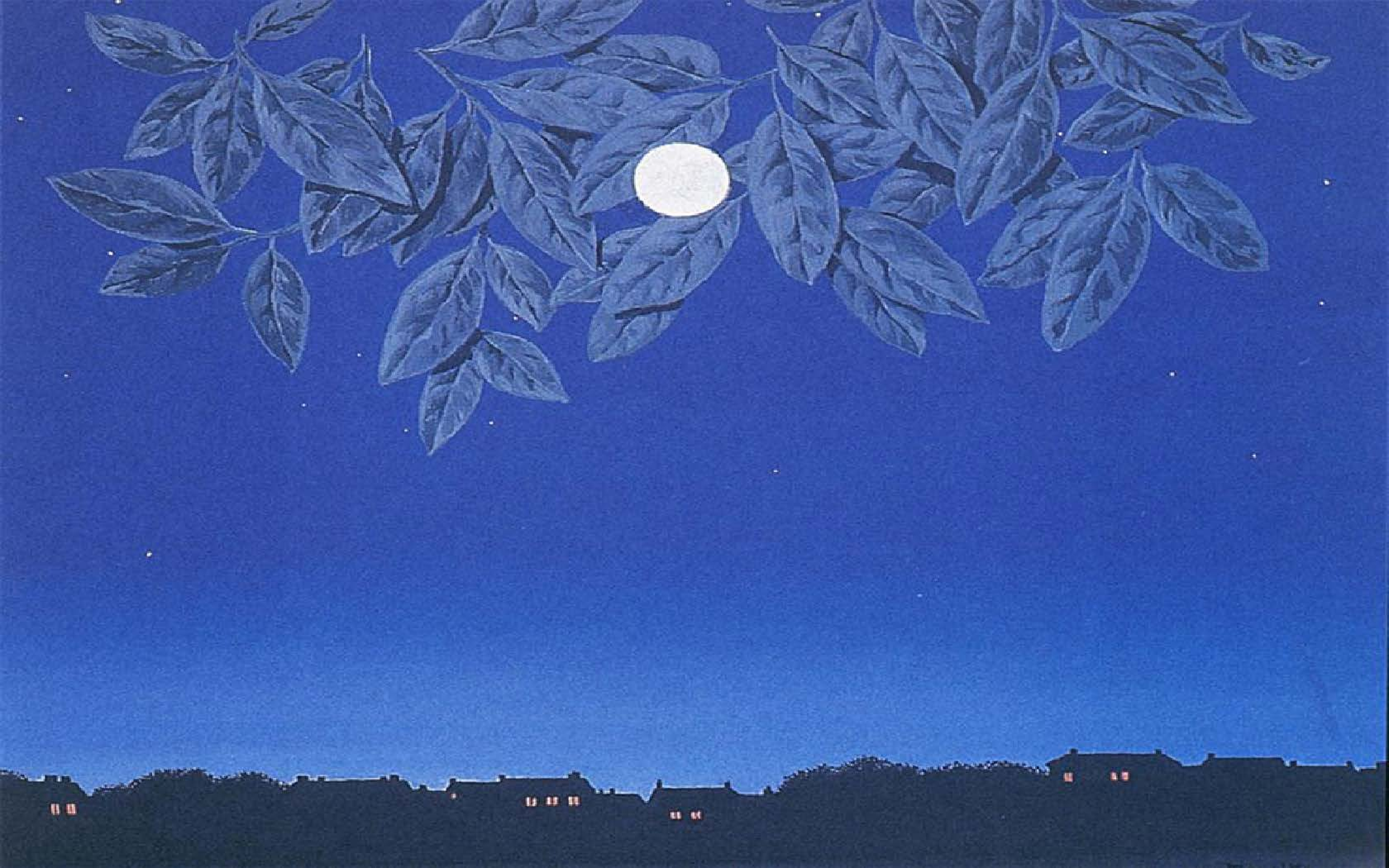

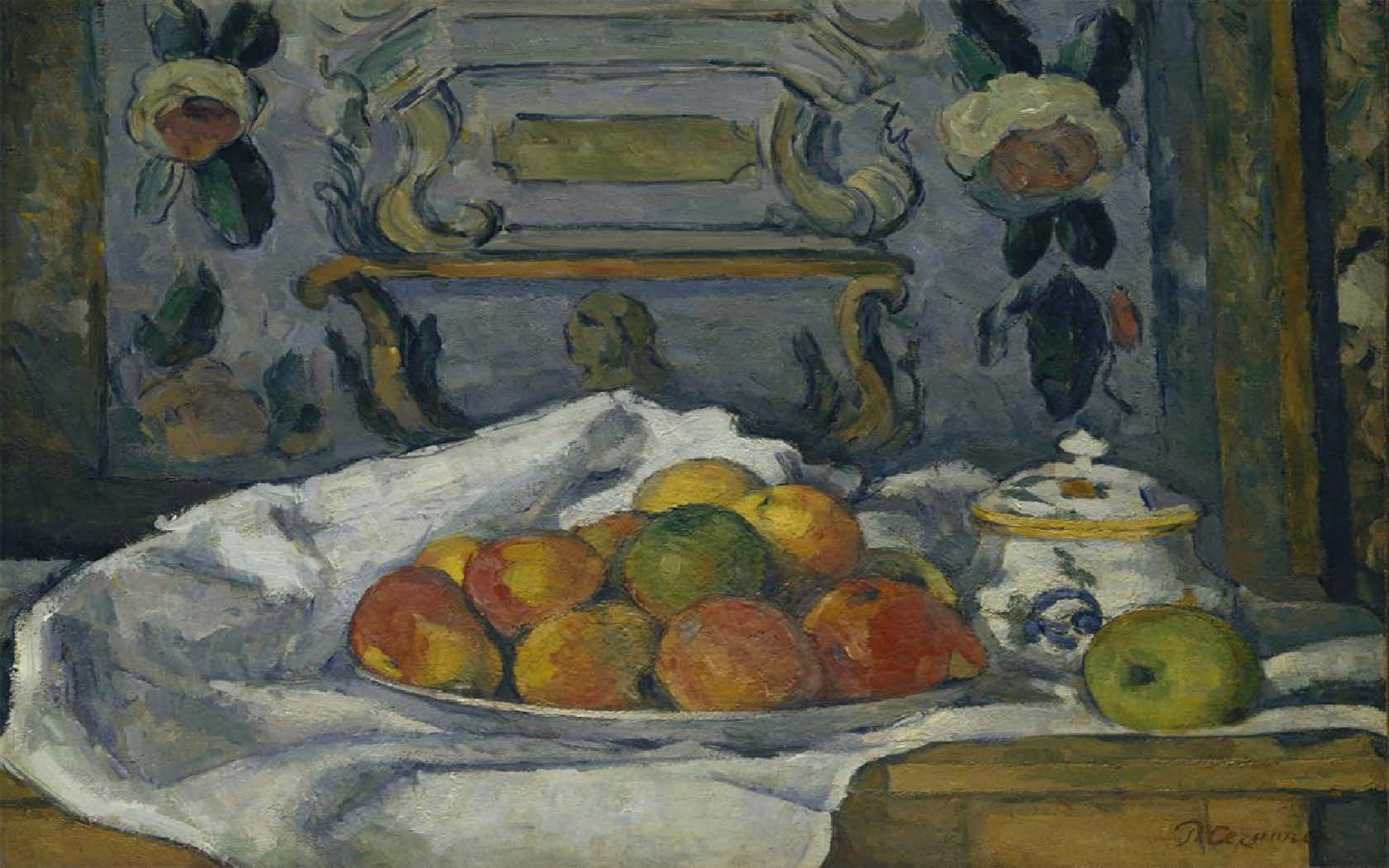

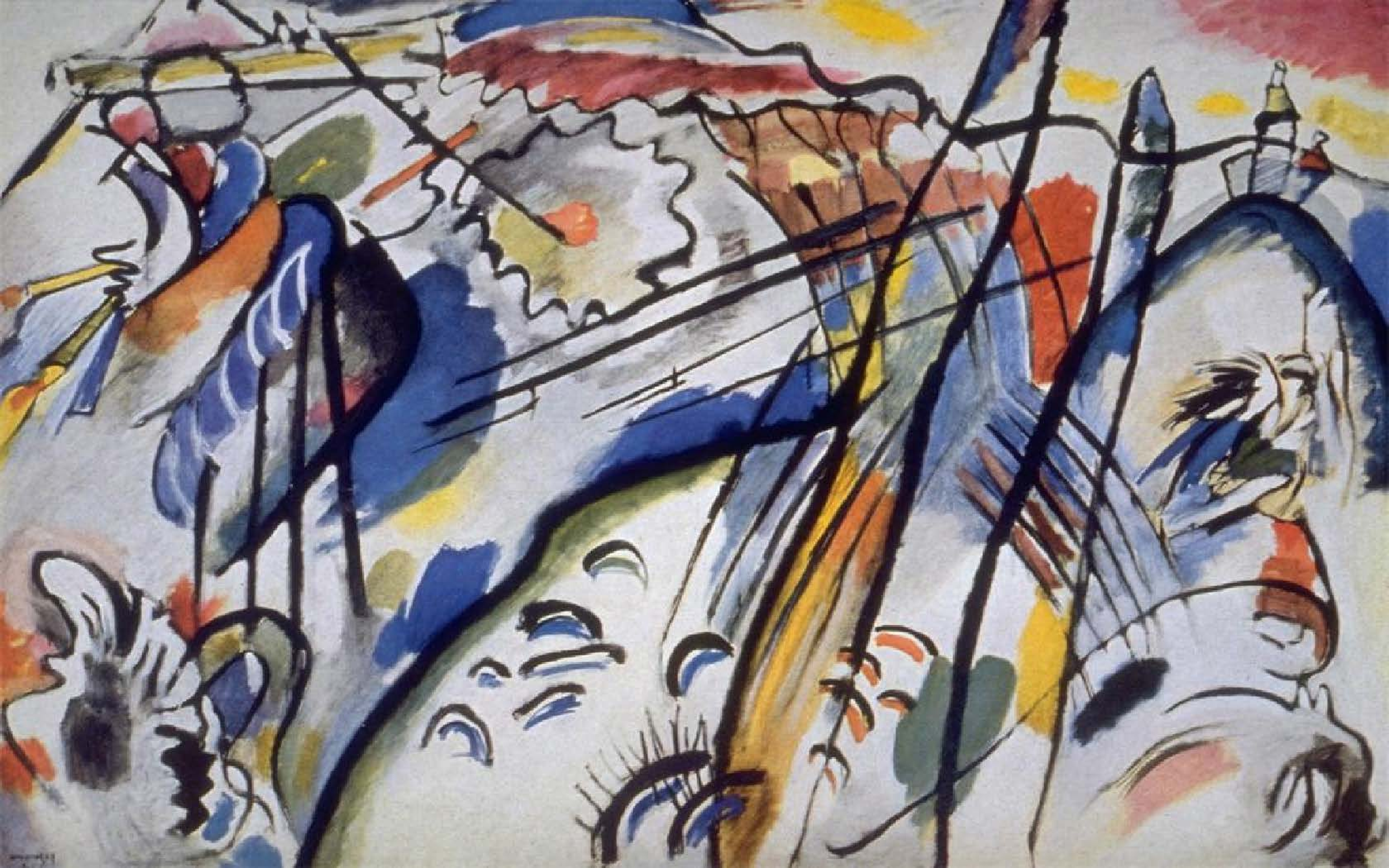

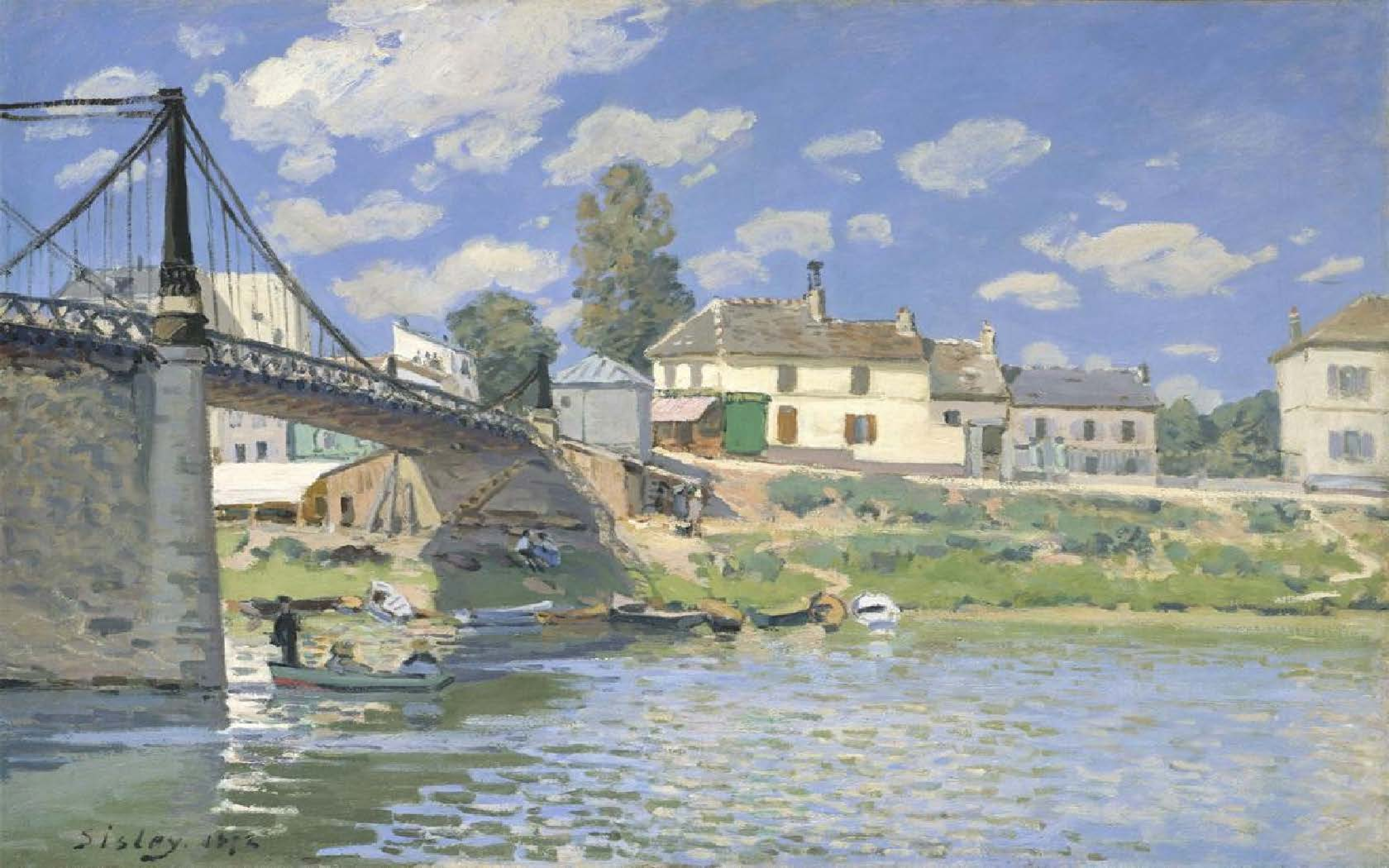
Sisley.

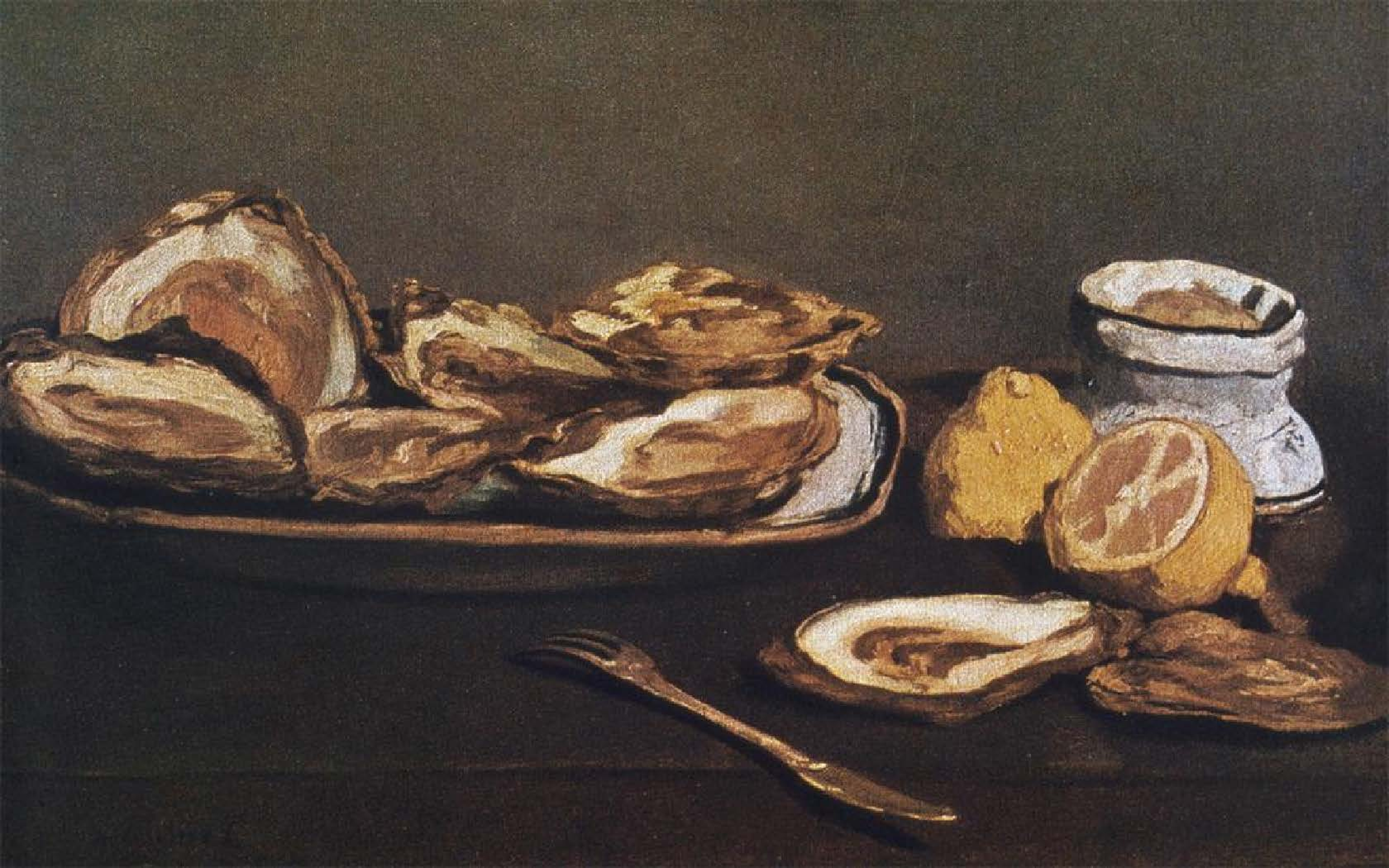

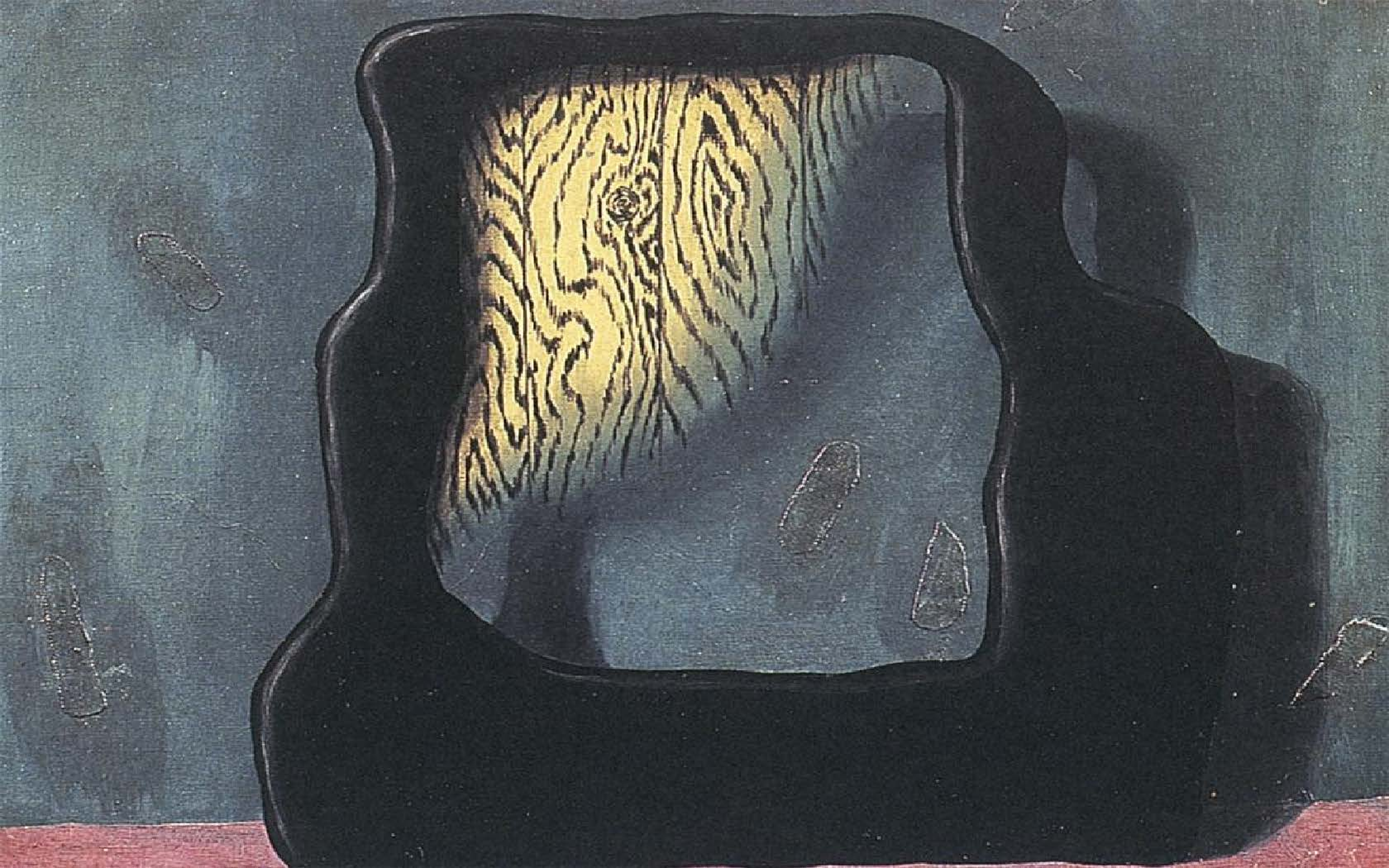

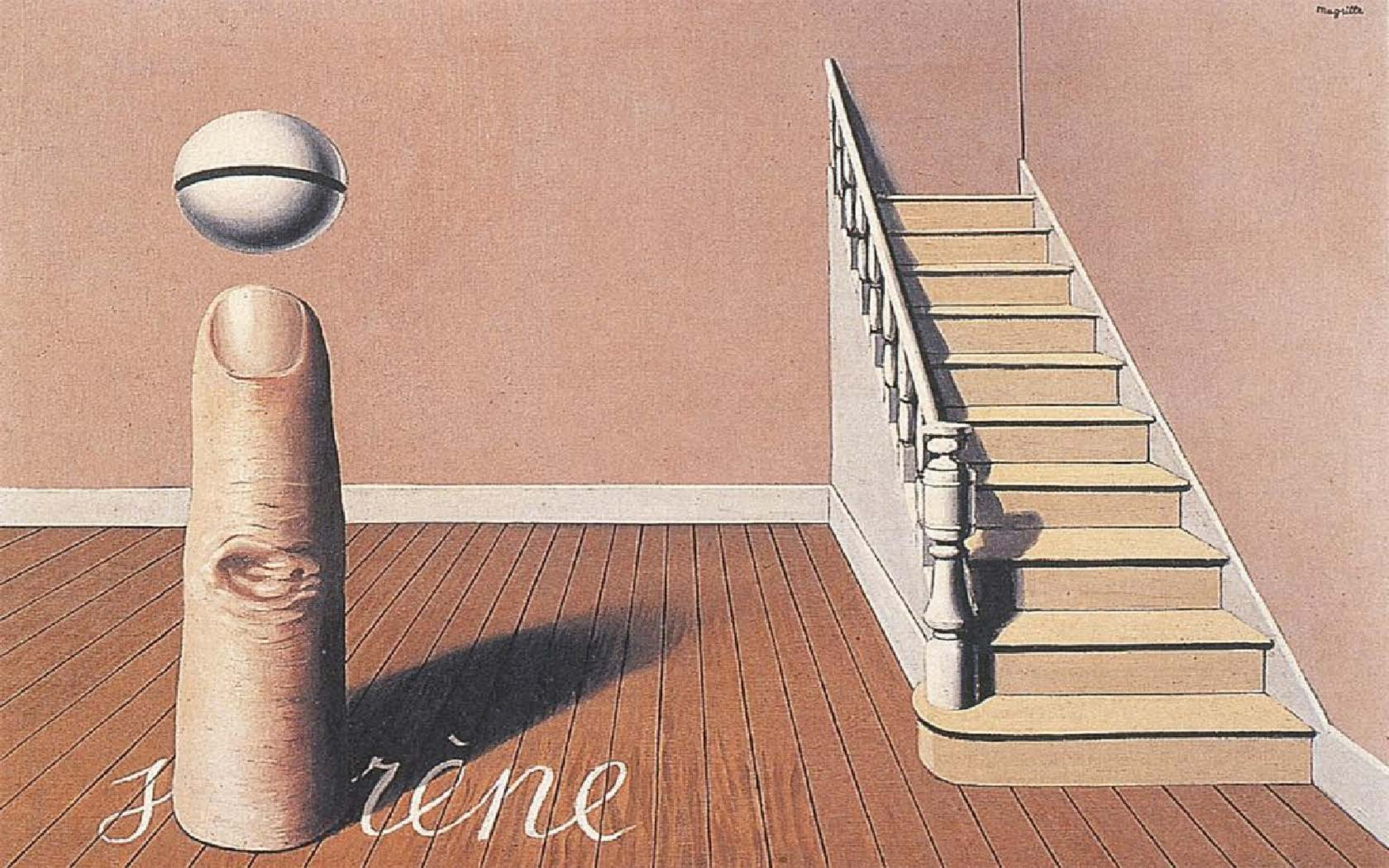
Magritte
Irène

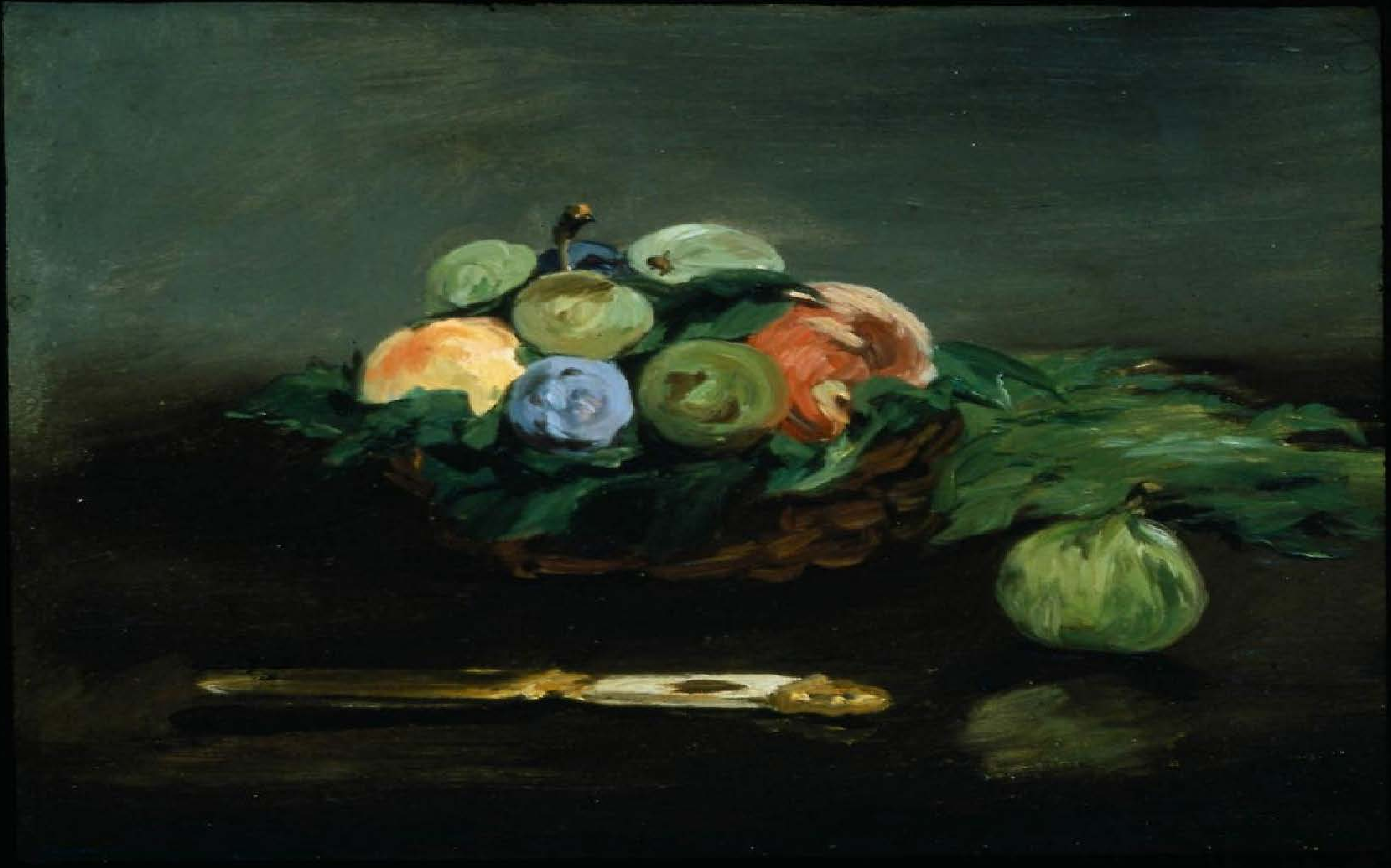

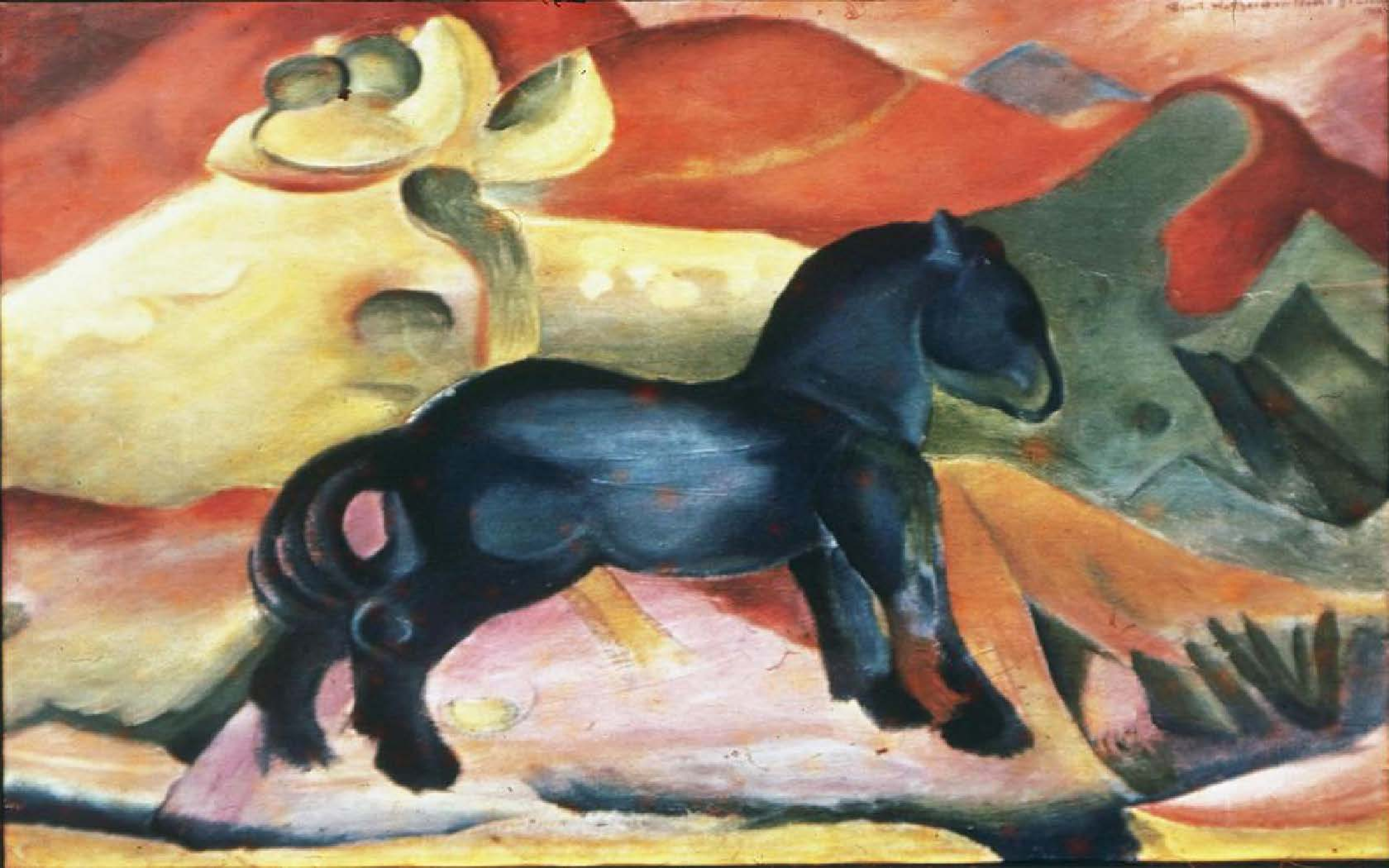

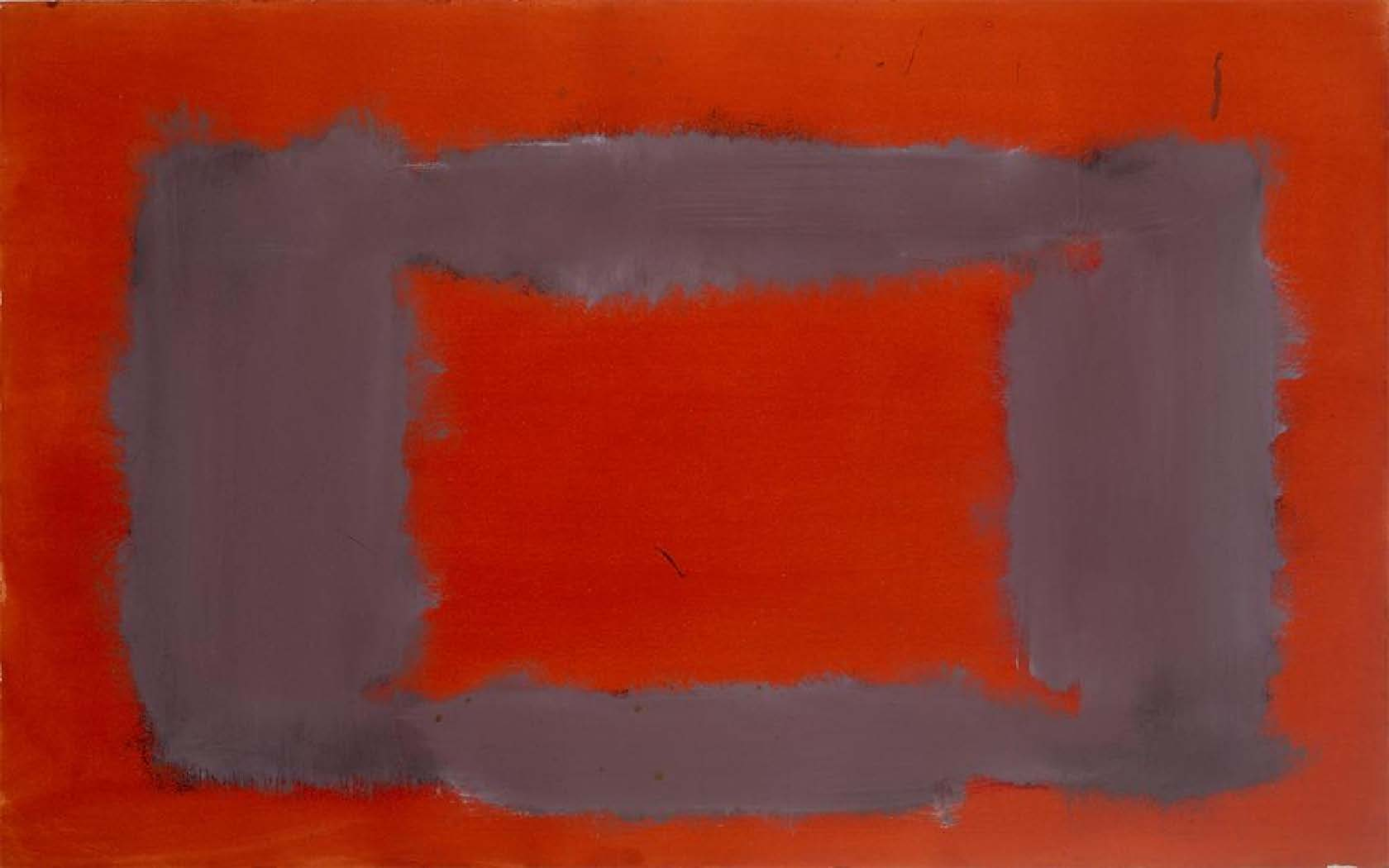

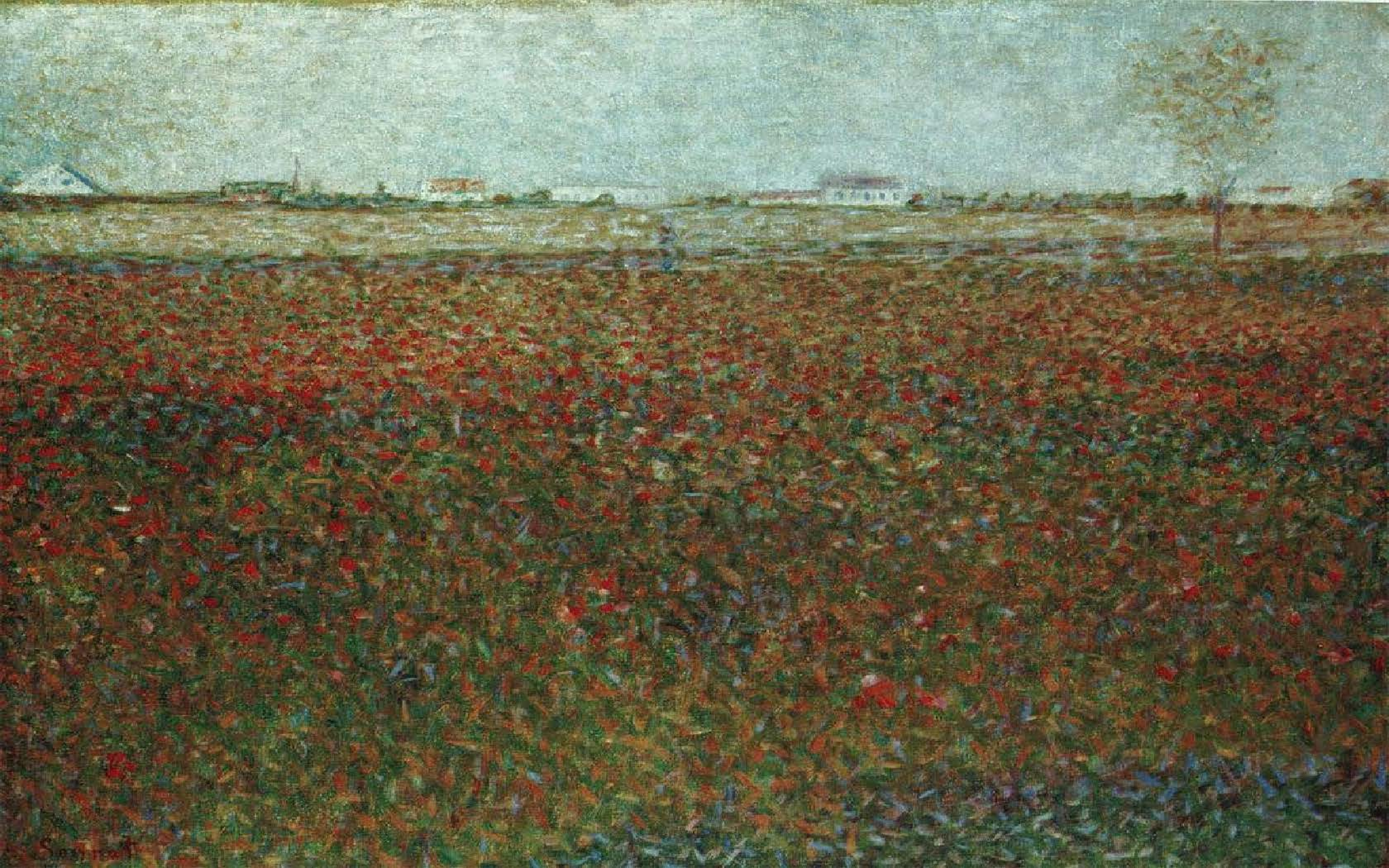

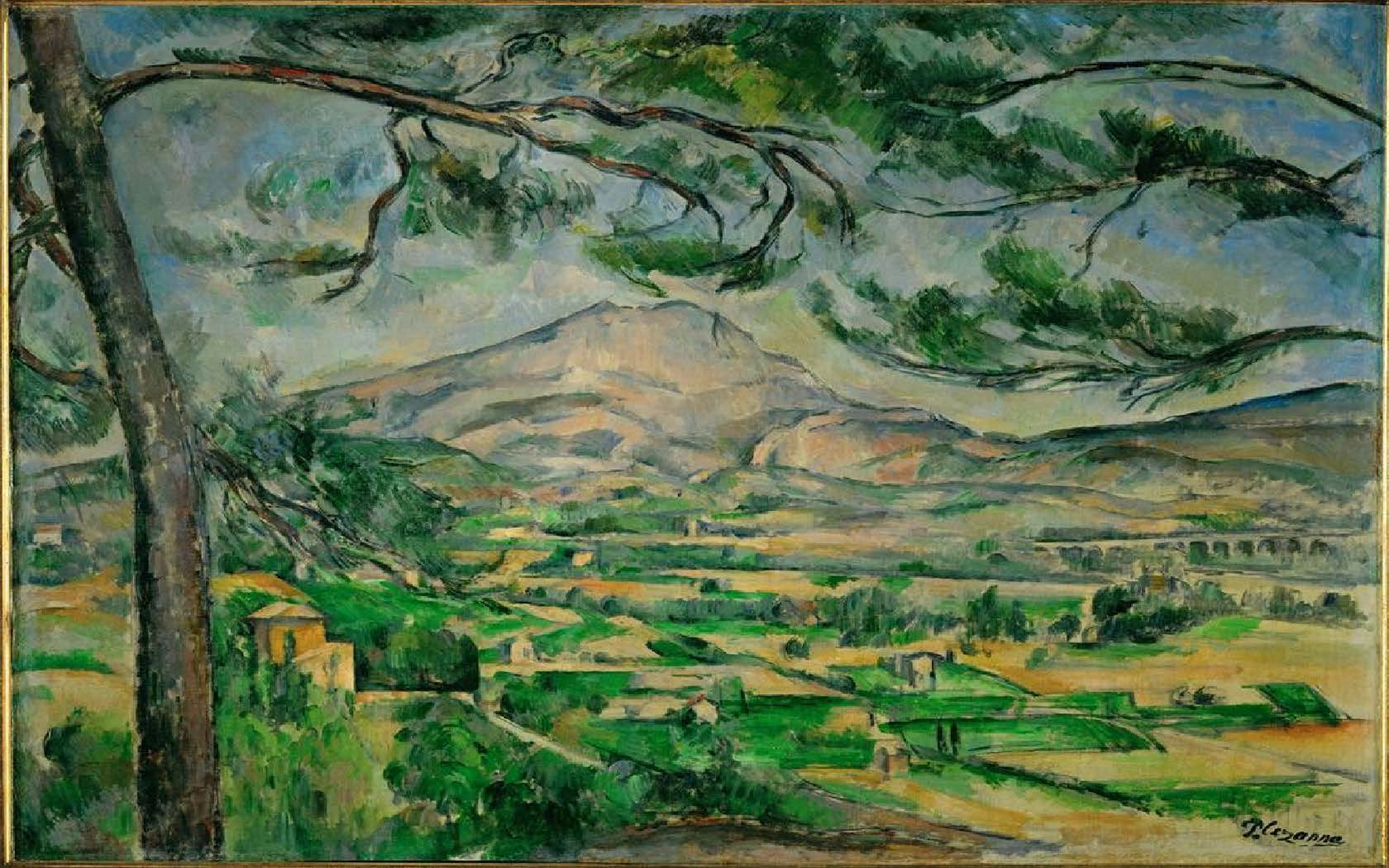
P. Cezanne

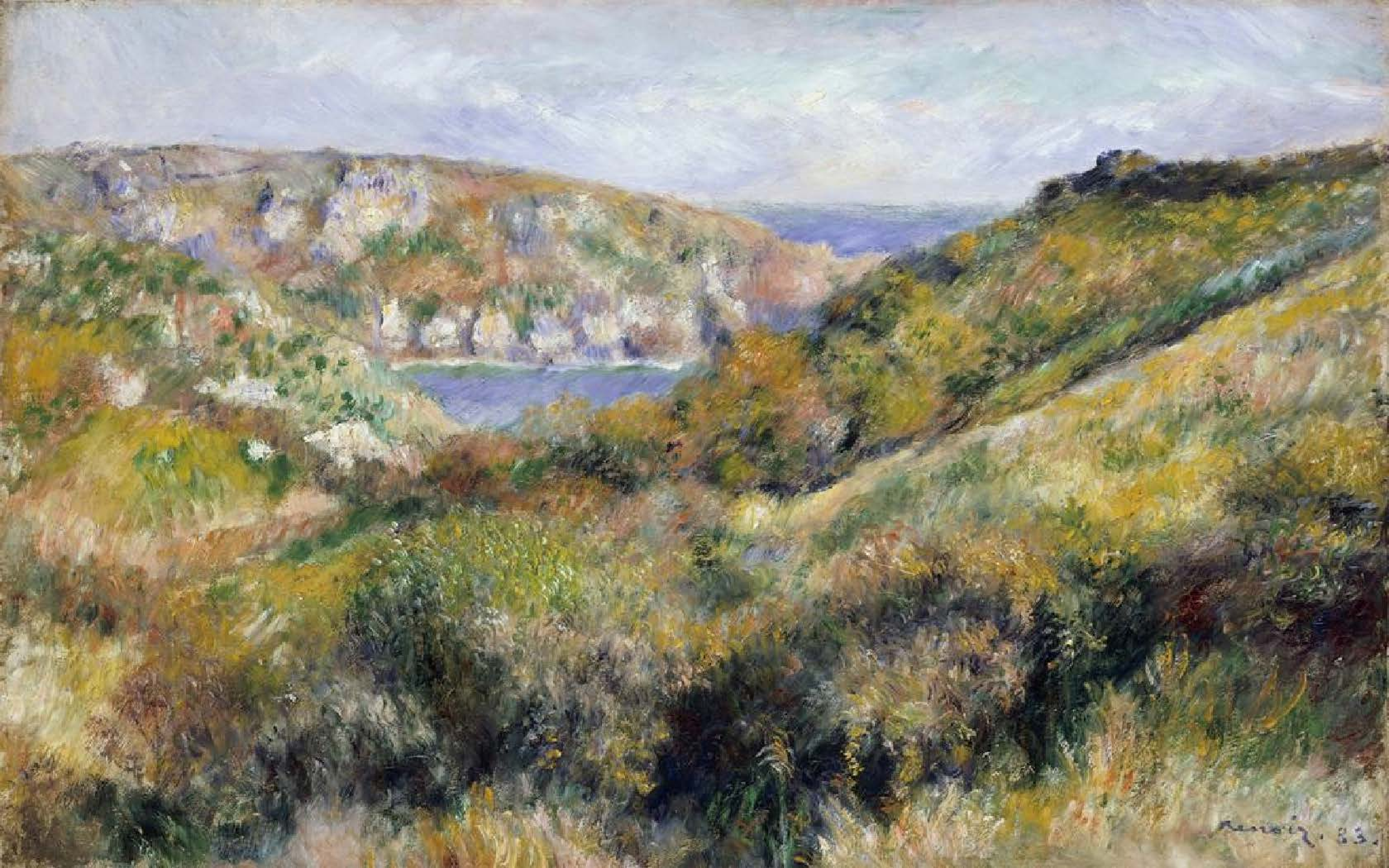
Renoir. 83.

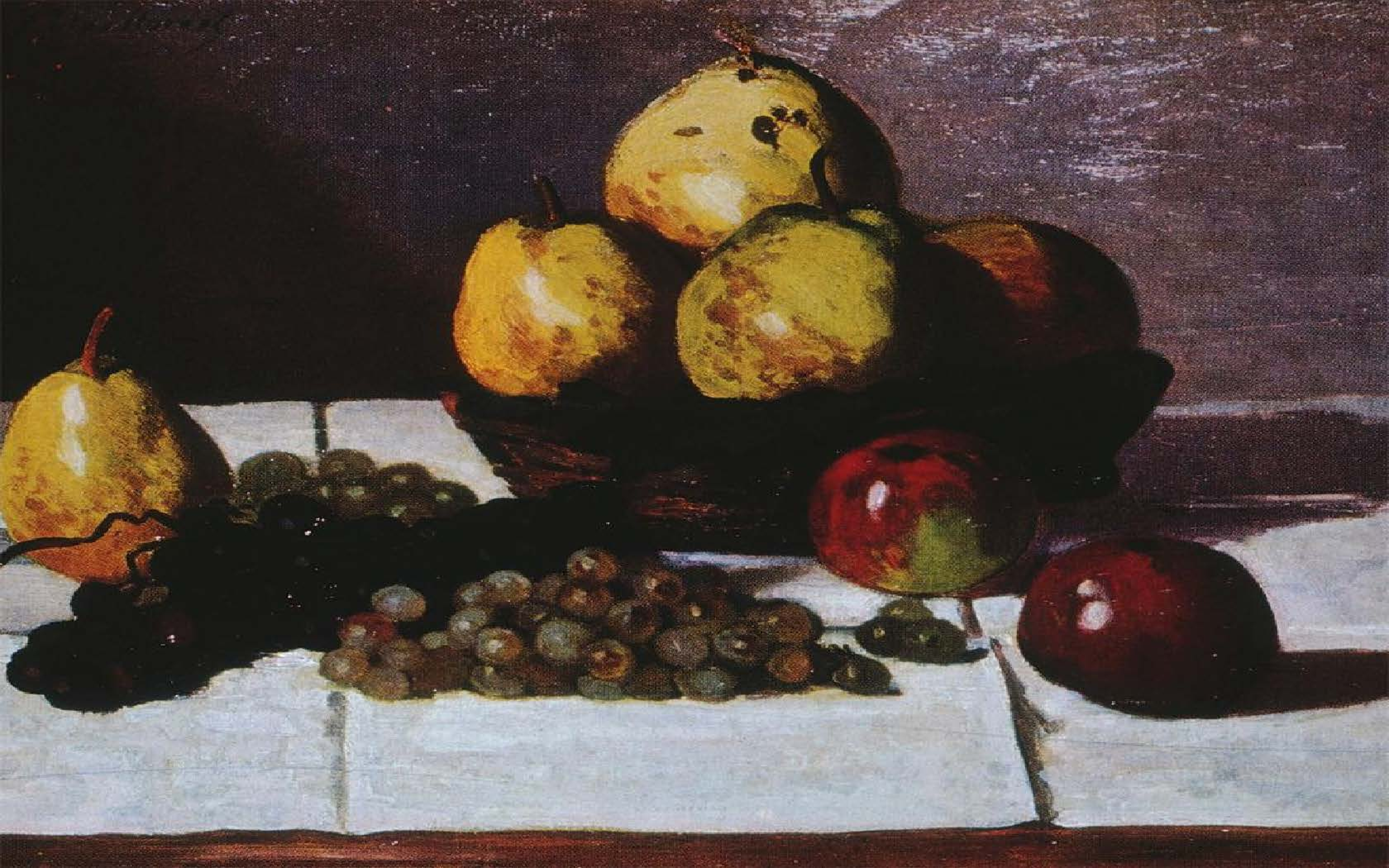

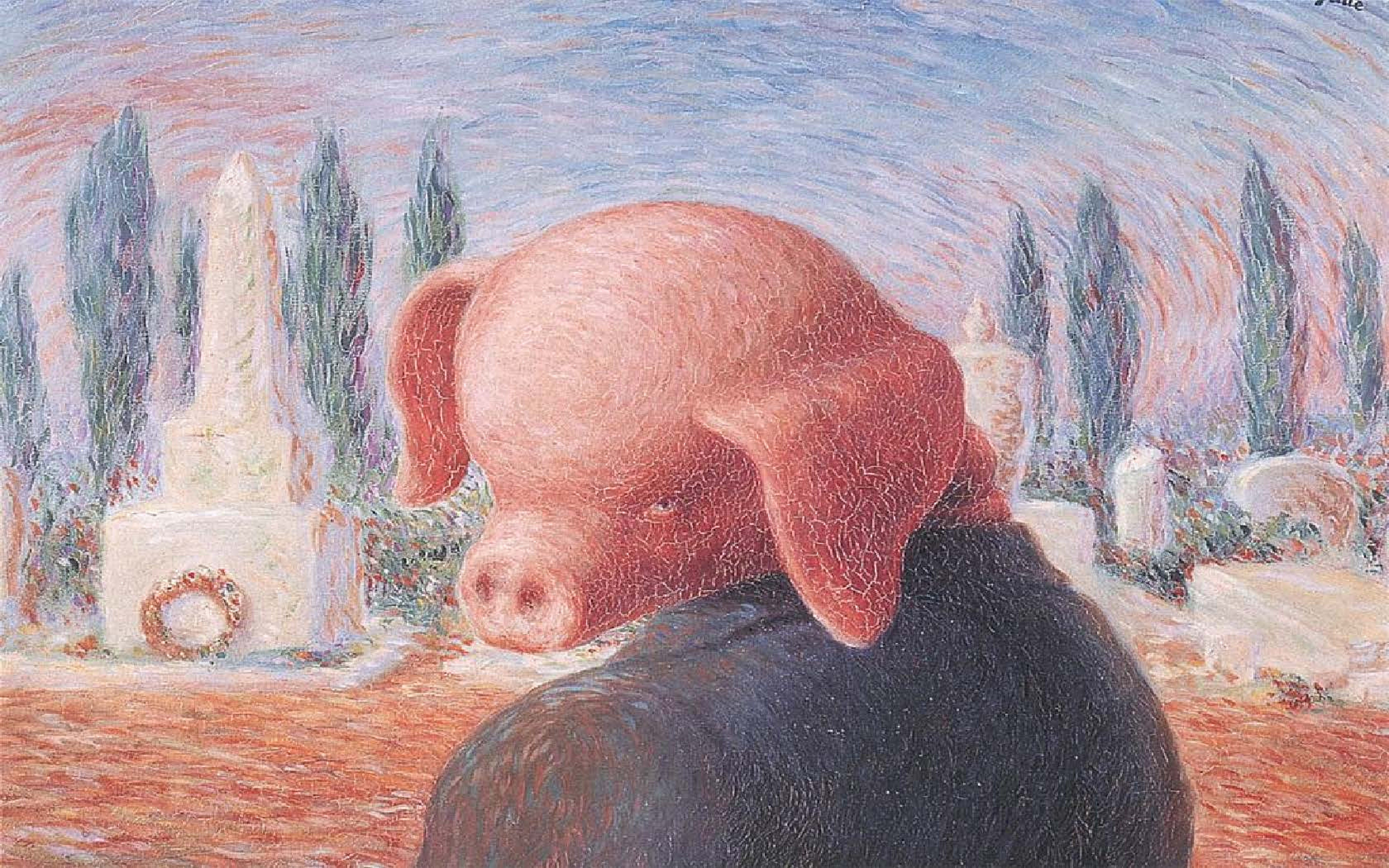

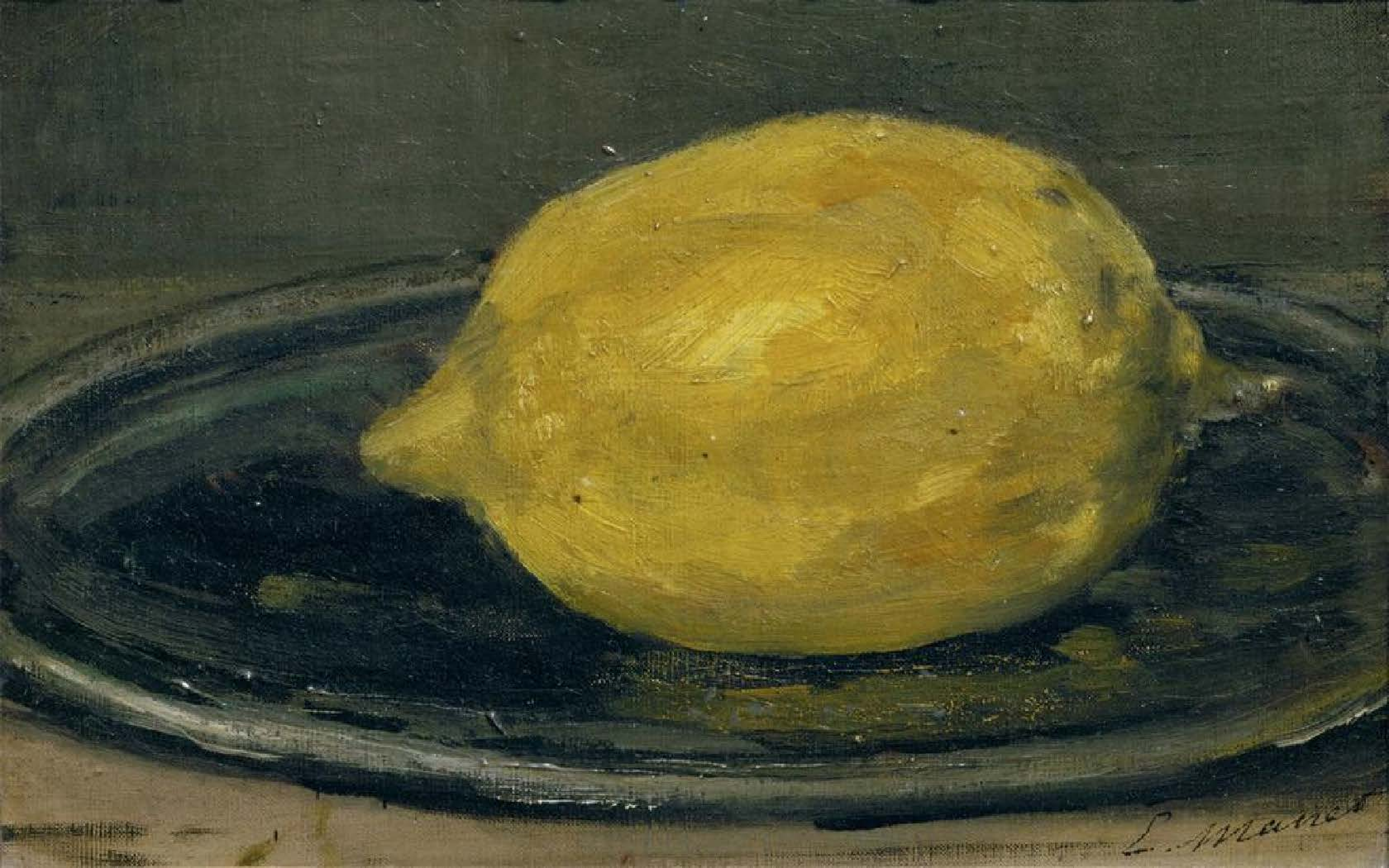
E. Manet

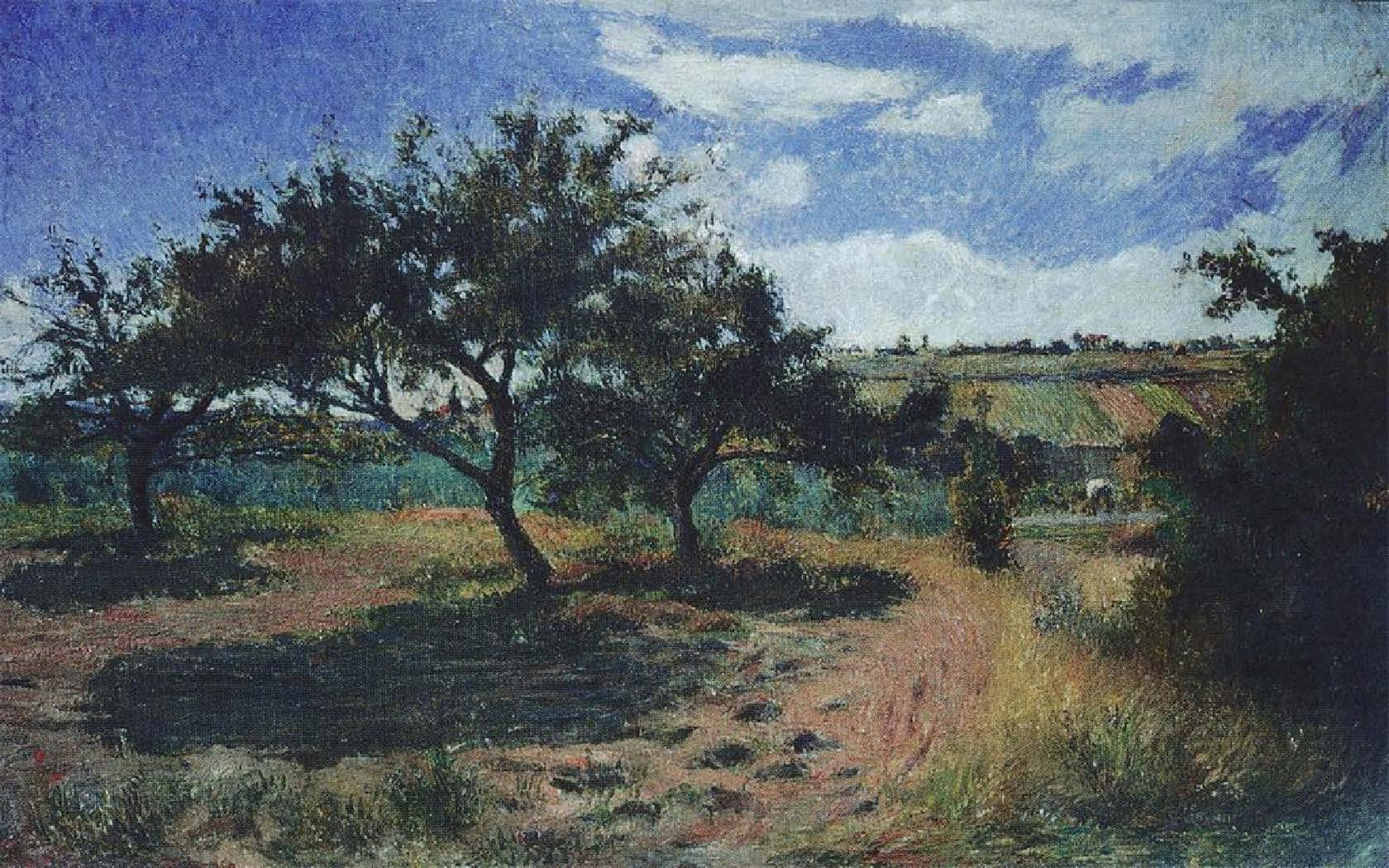

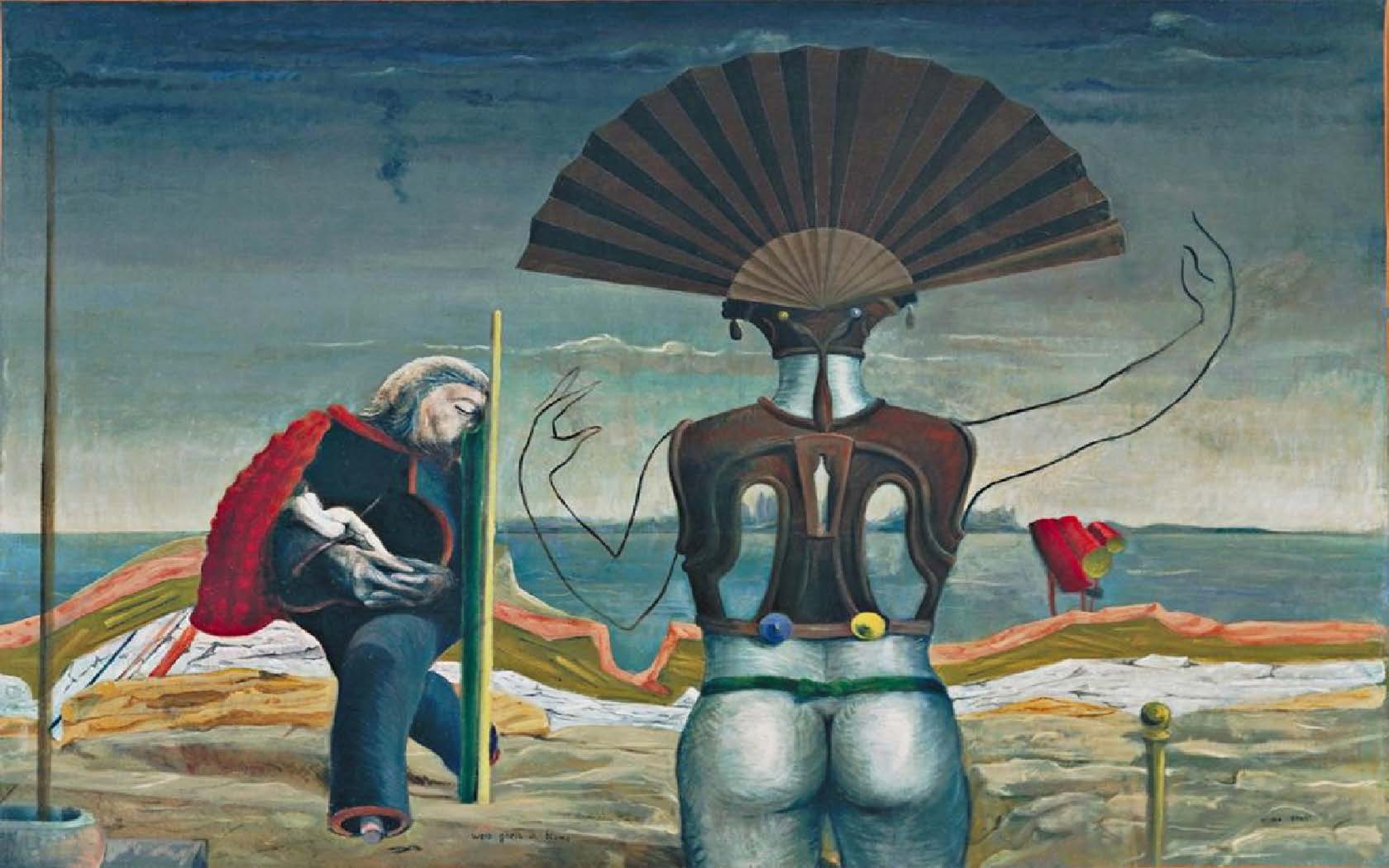

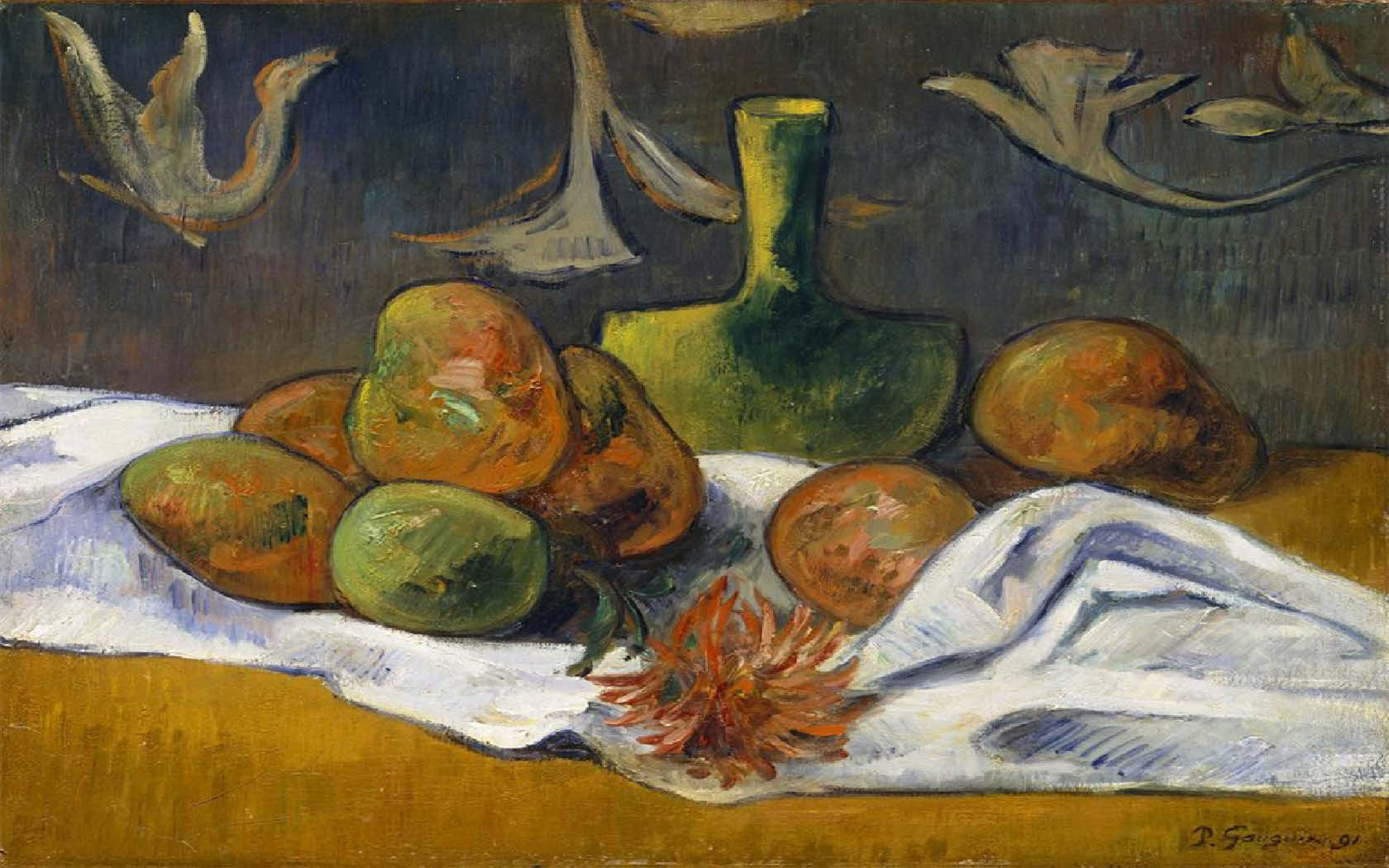
P. Gauguin 91

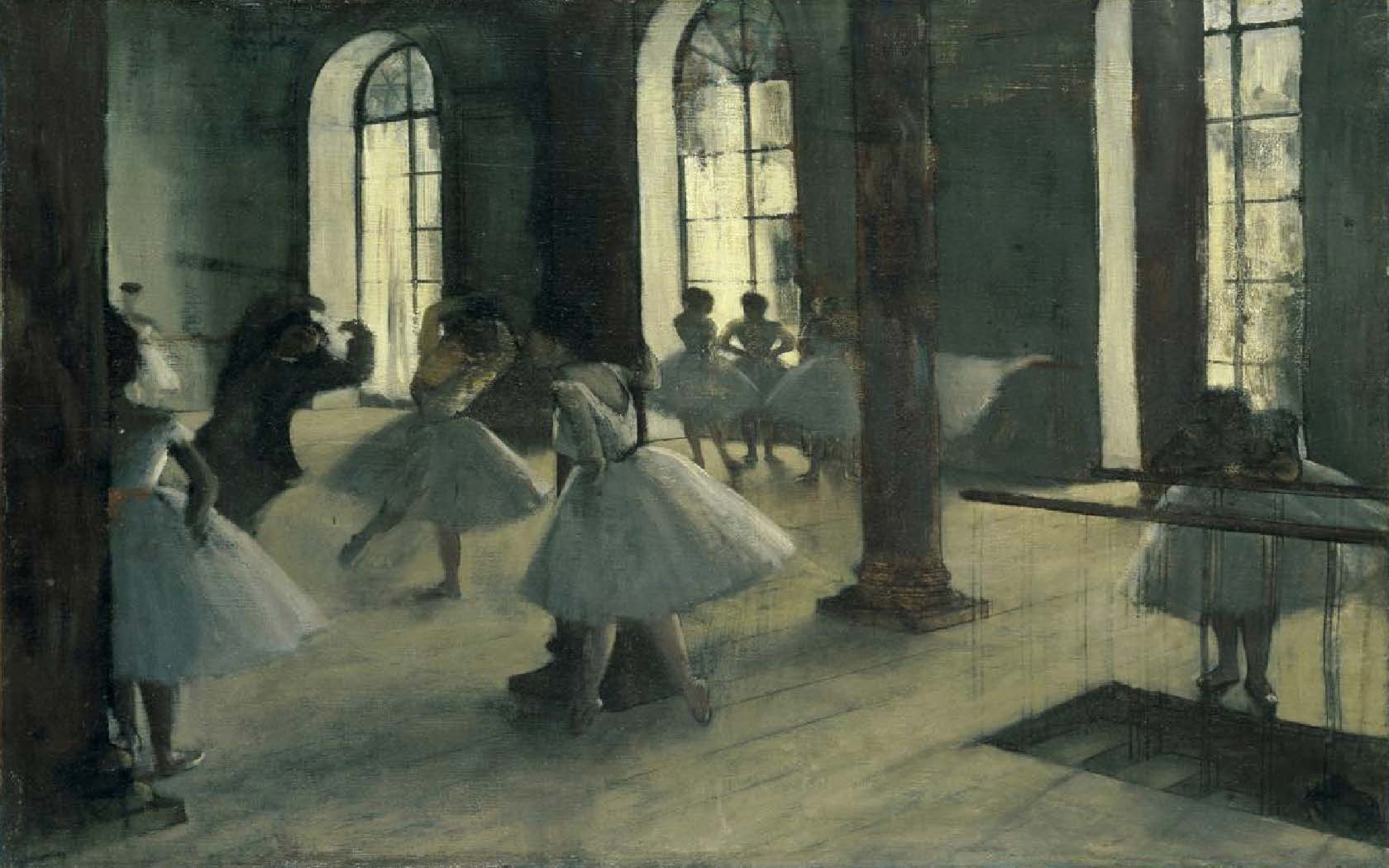

Supplement: Supplementary file 1 [file Data_Sheet_1.PDF]
