# Supplementary material for: Emotional Responses to Visual Art and Commercial Stimuli: Implications for Creativity and Aesthetics
Source: Front Psychol. 2019 Jan 22;10:14. doi: 10.3389/fpsyg.2019.00014 (PMC6349741; doi:10.3389/fpsyg.2019.00014)

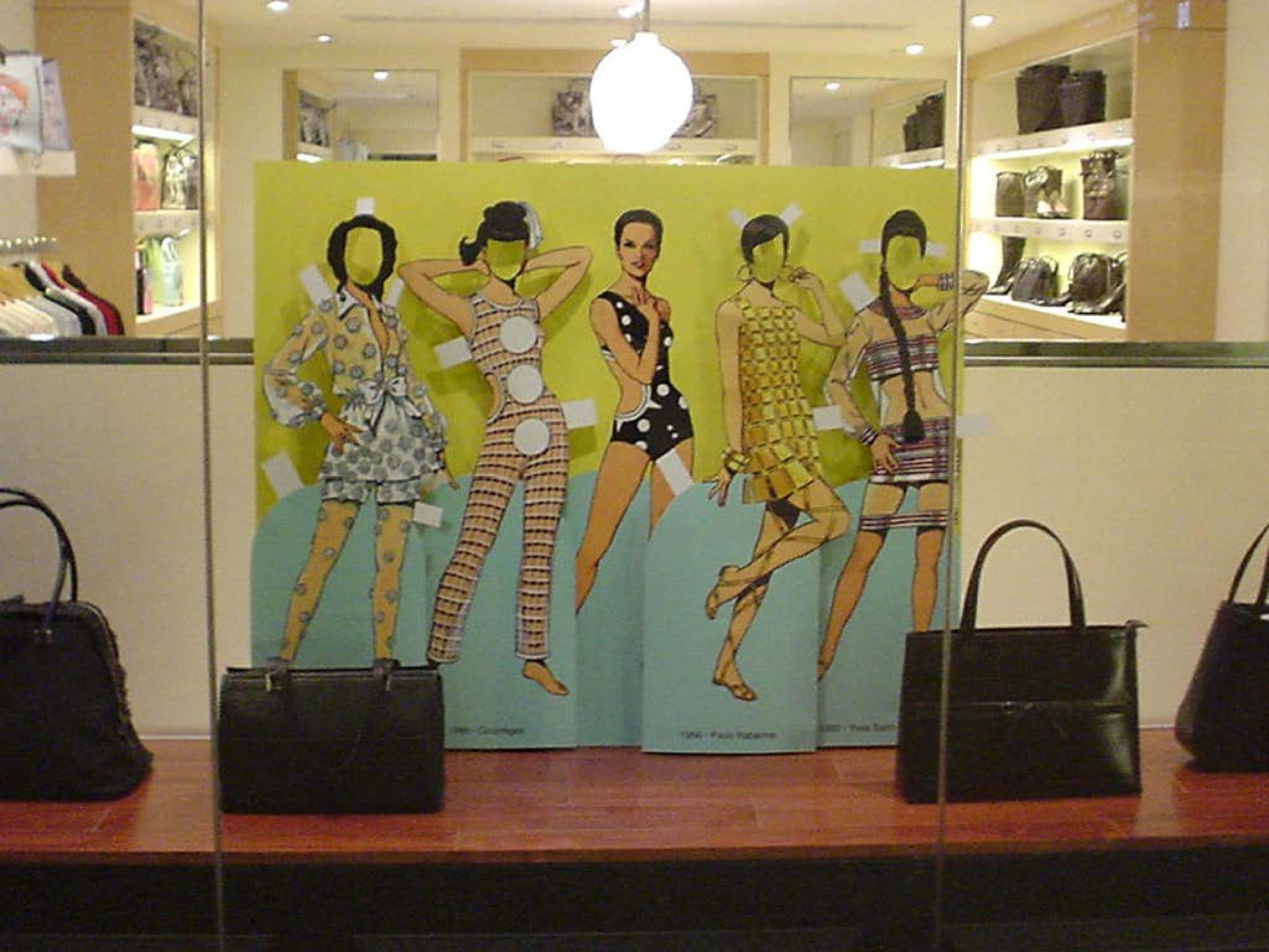

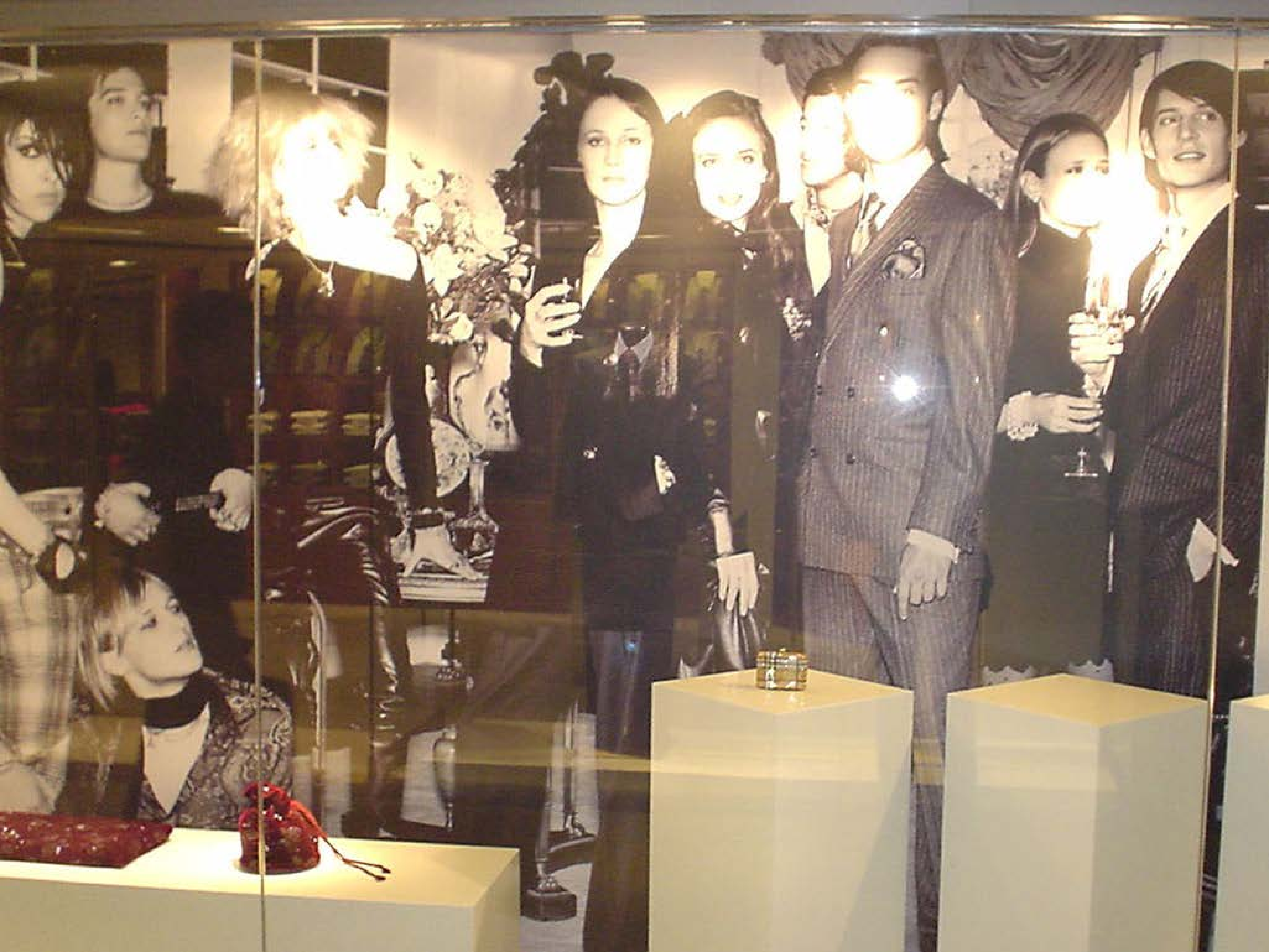

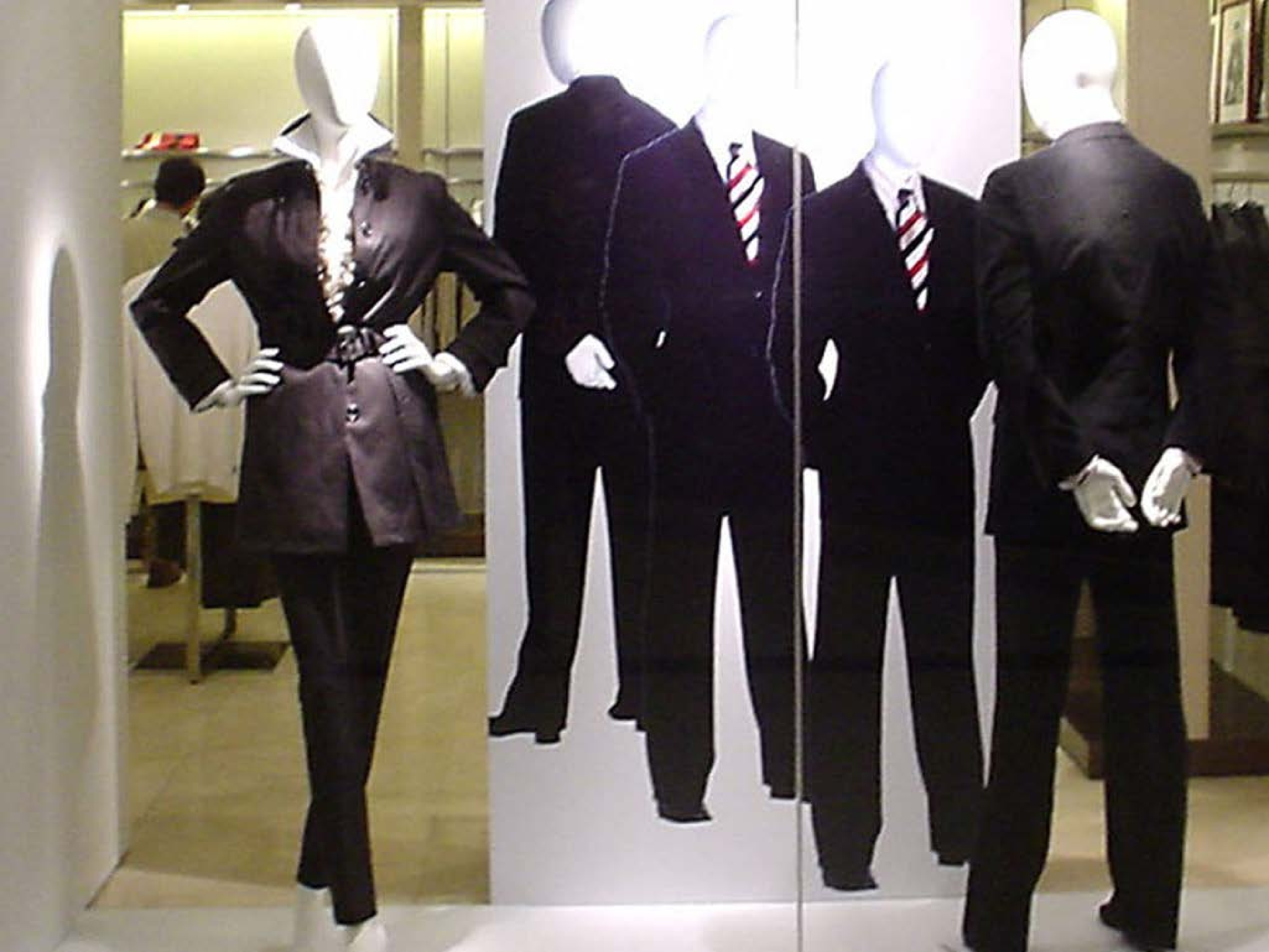

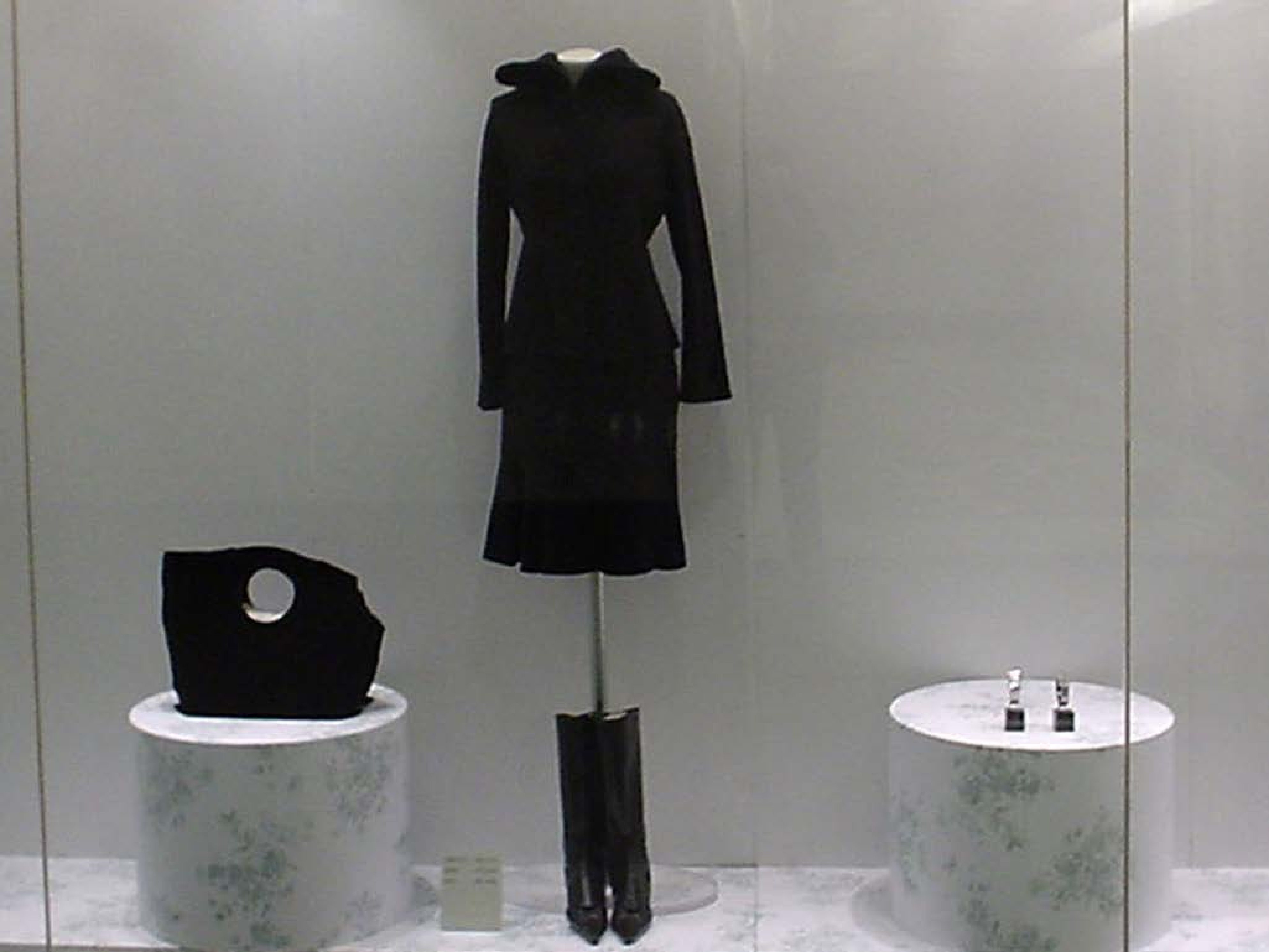

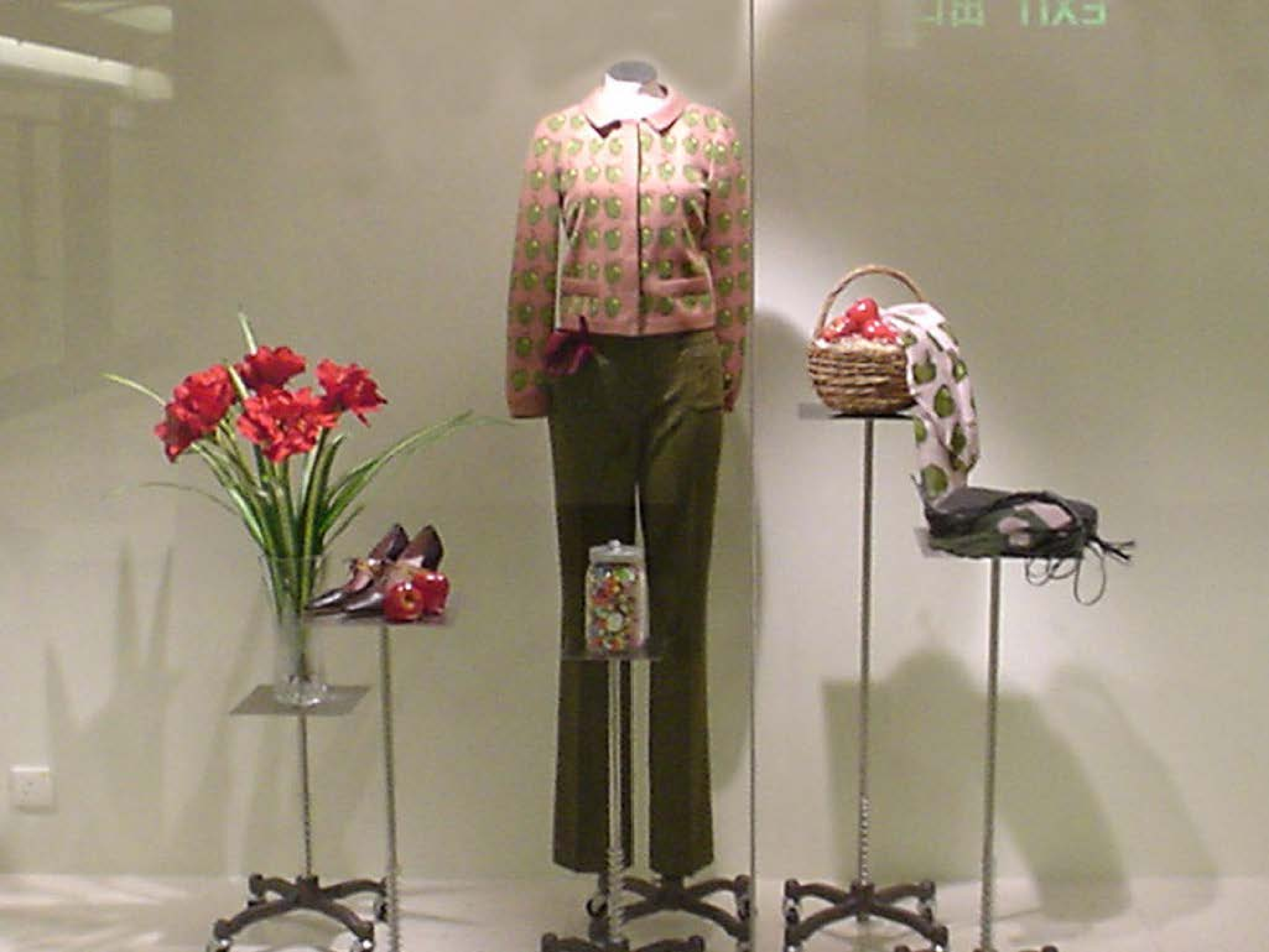

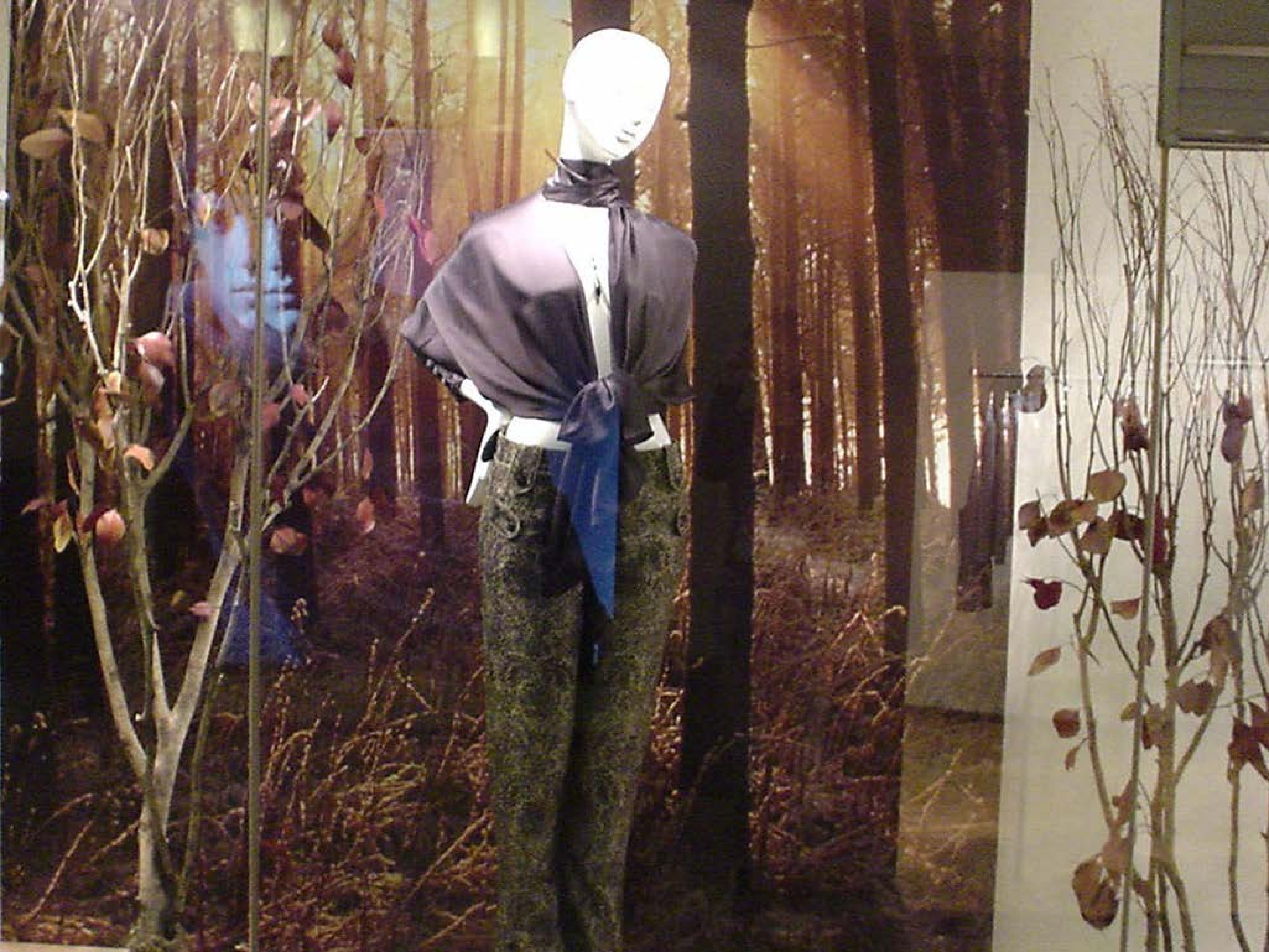

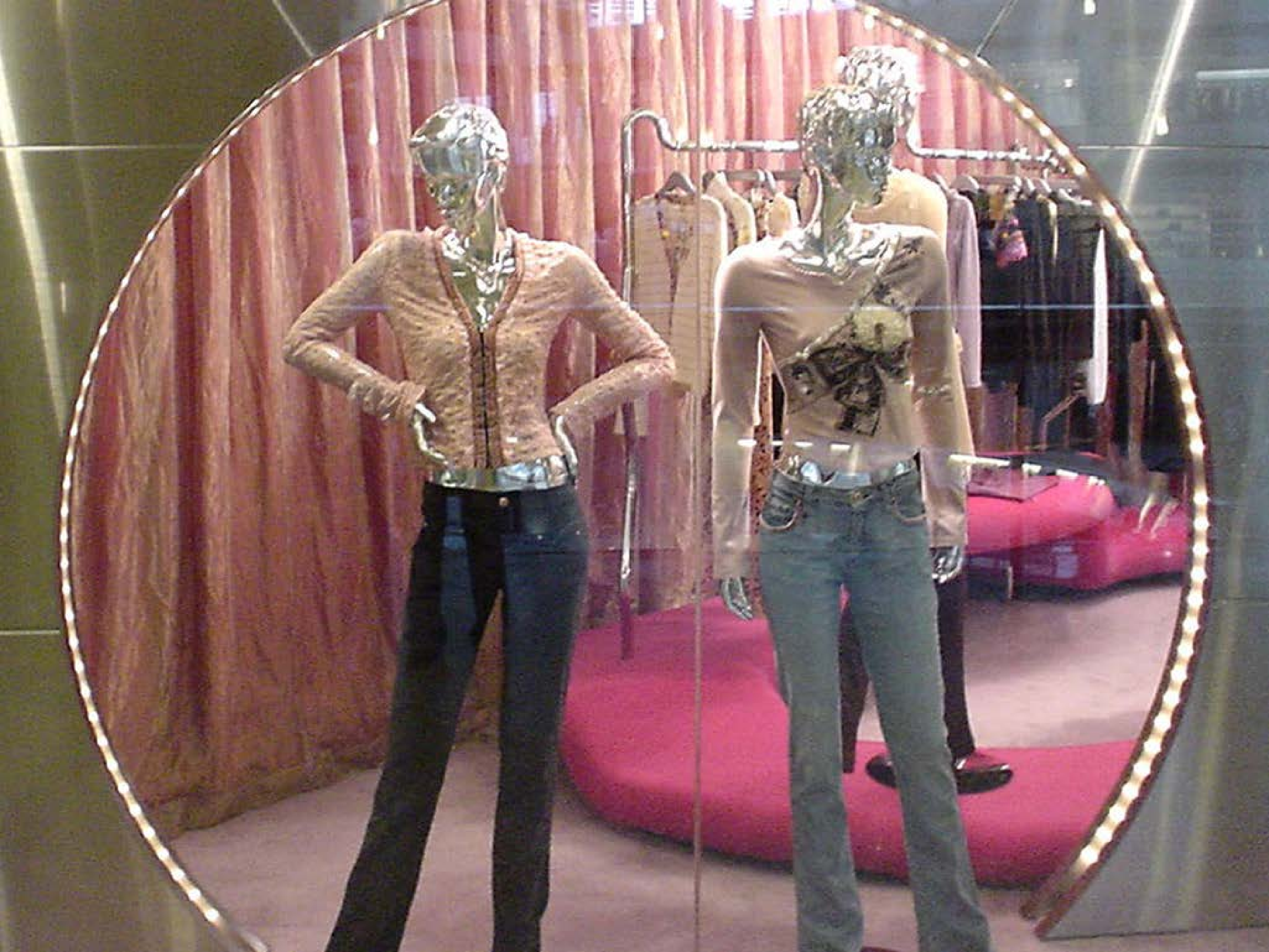

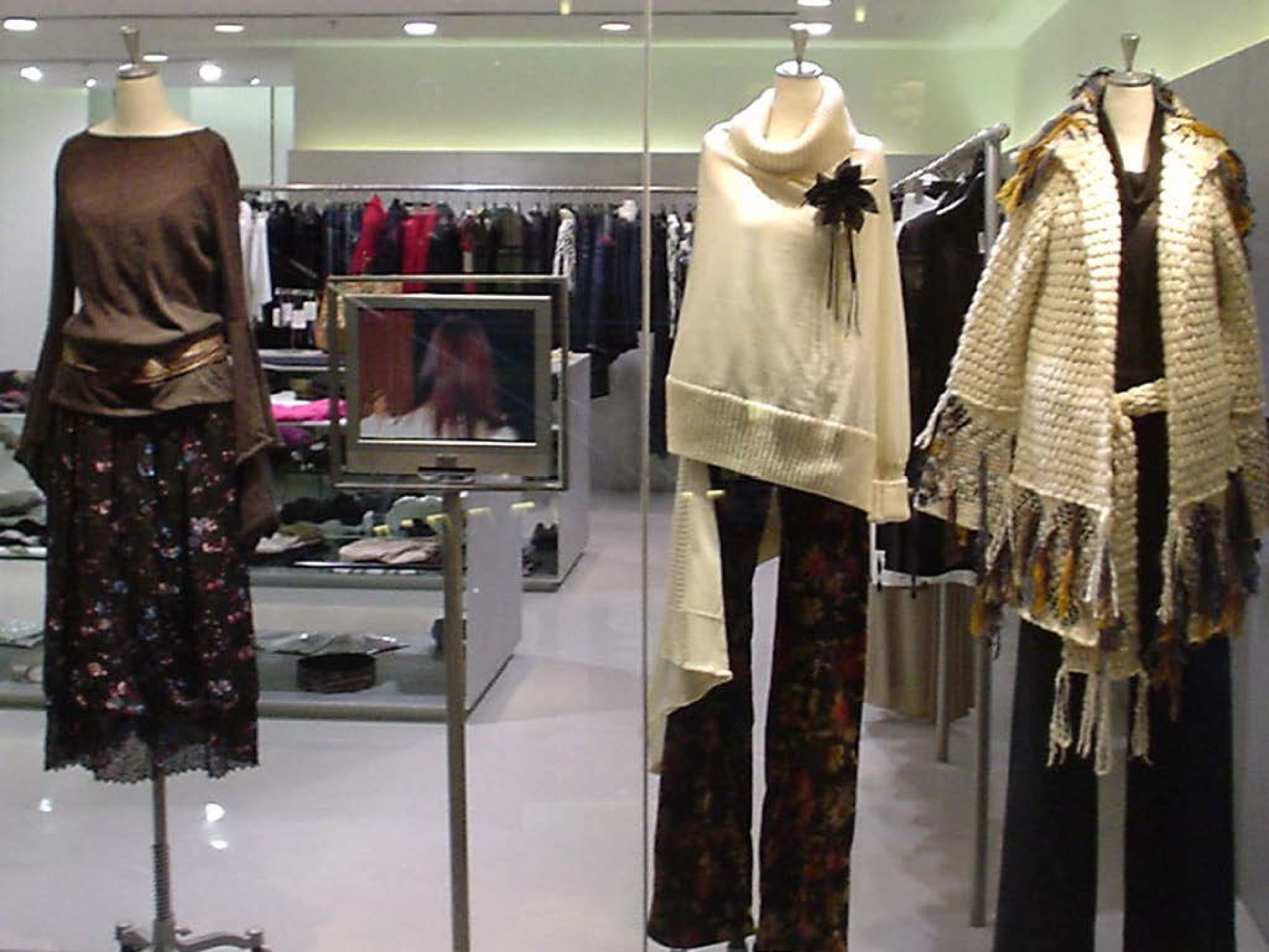

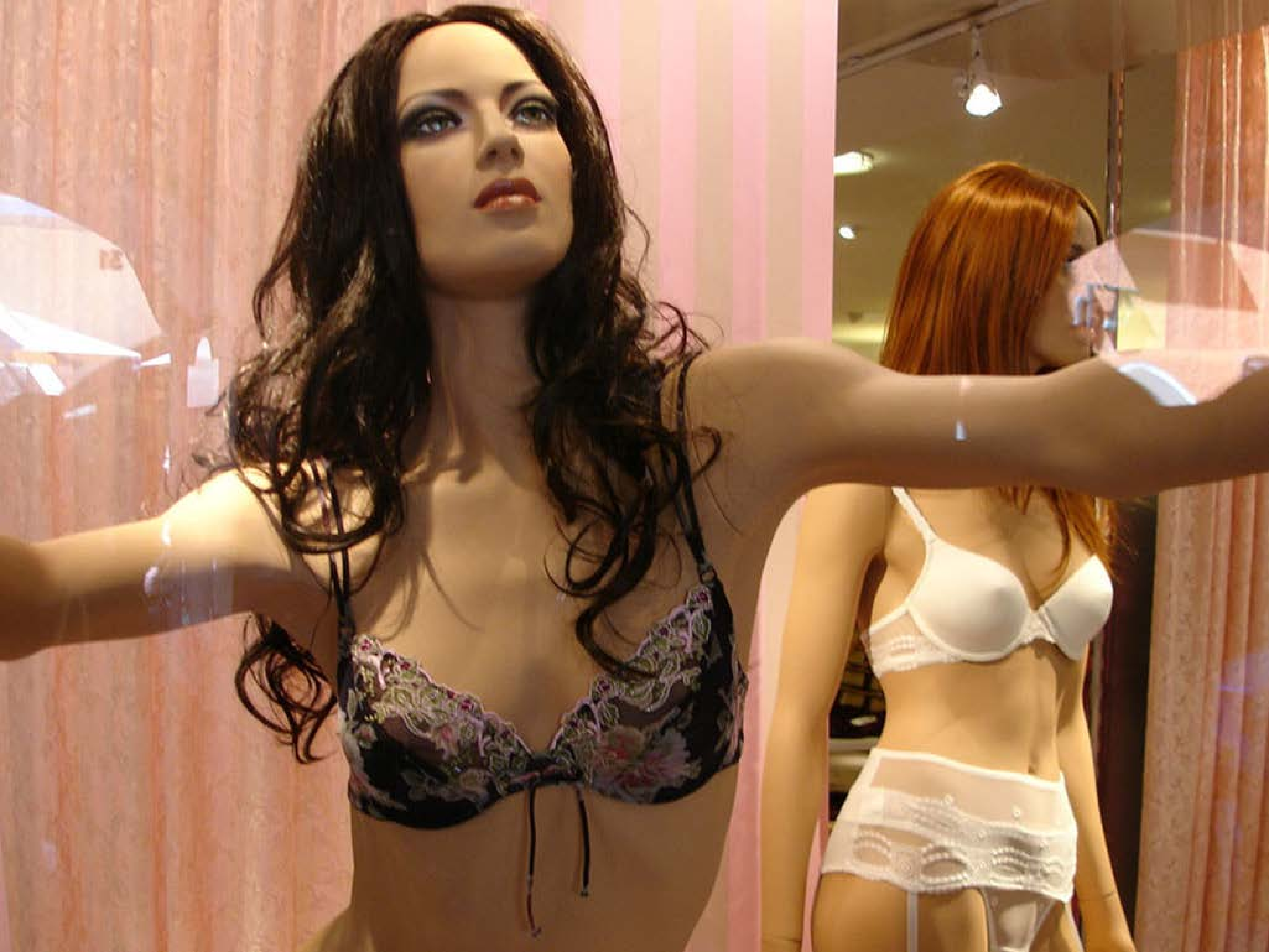

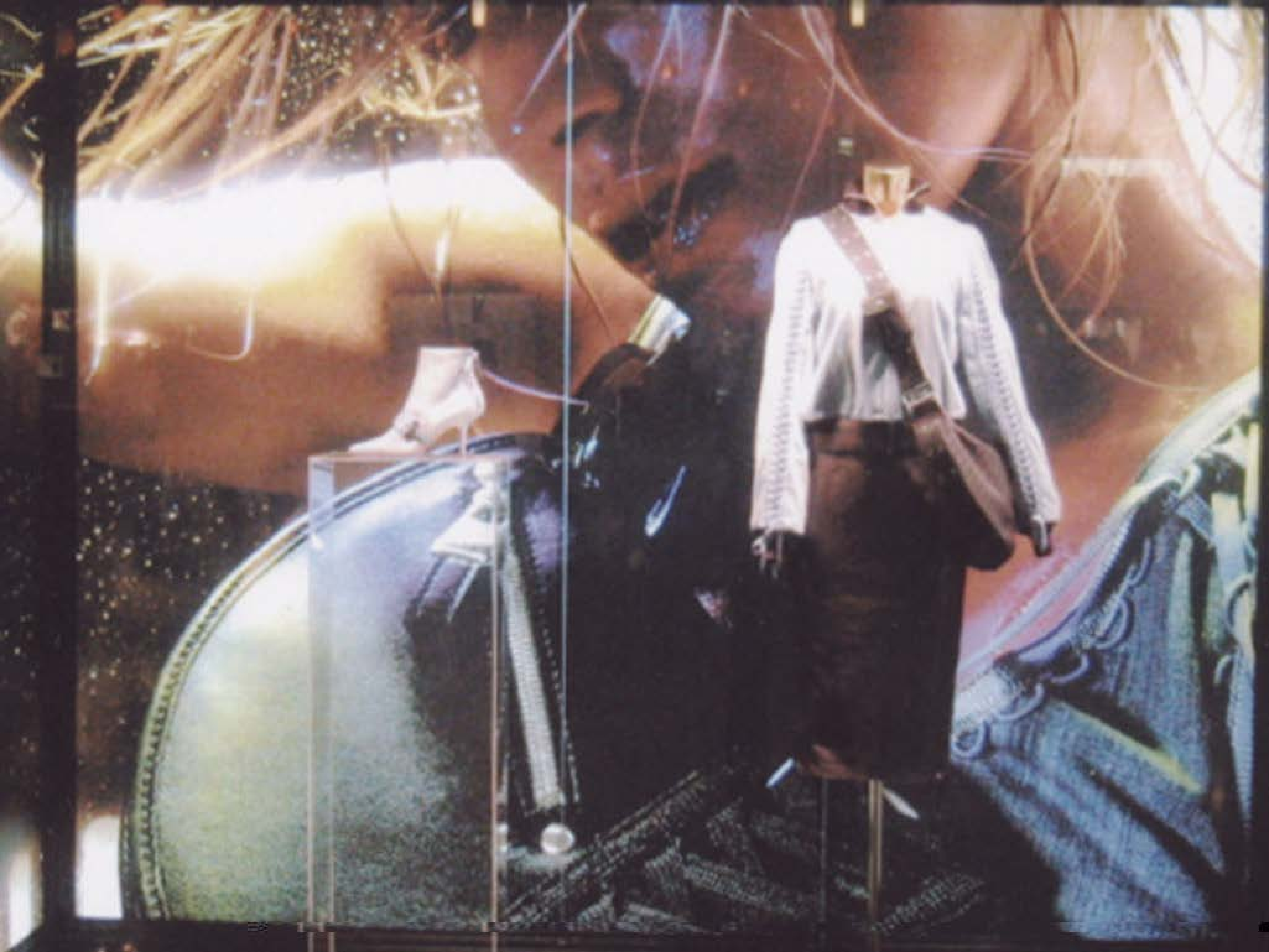

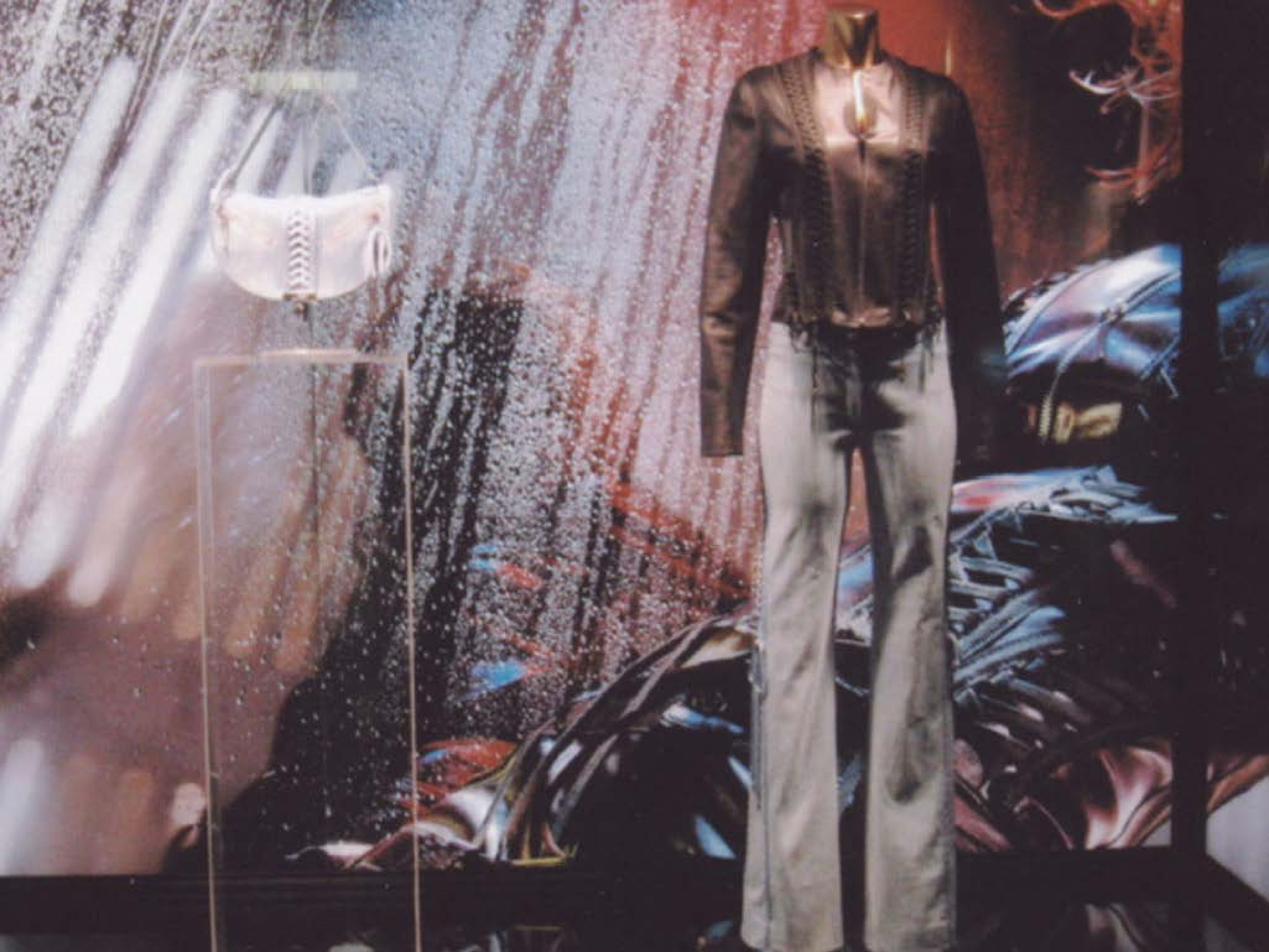

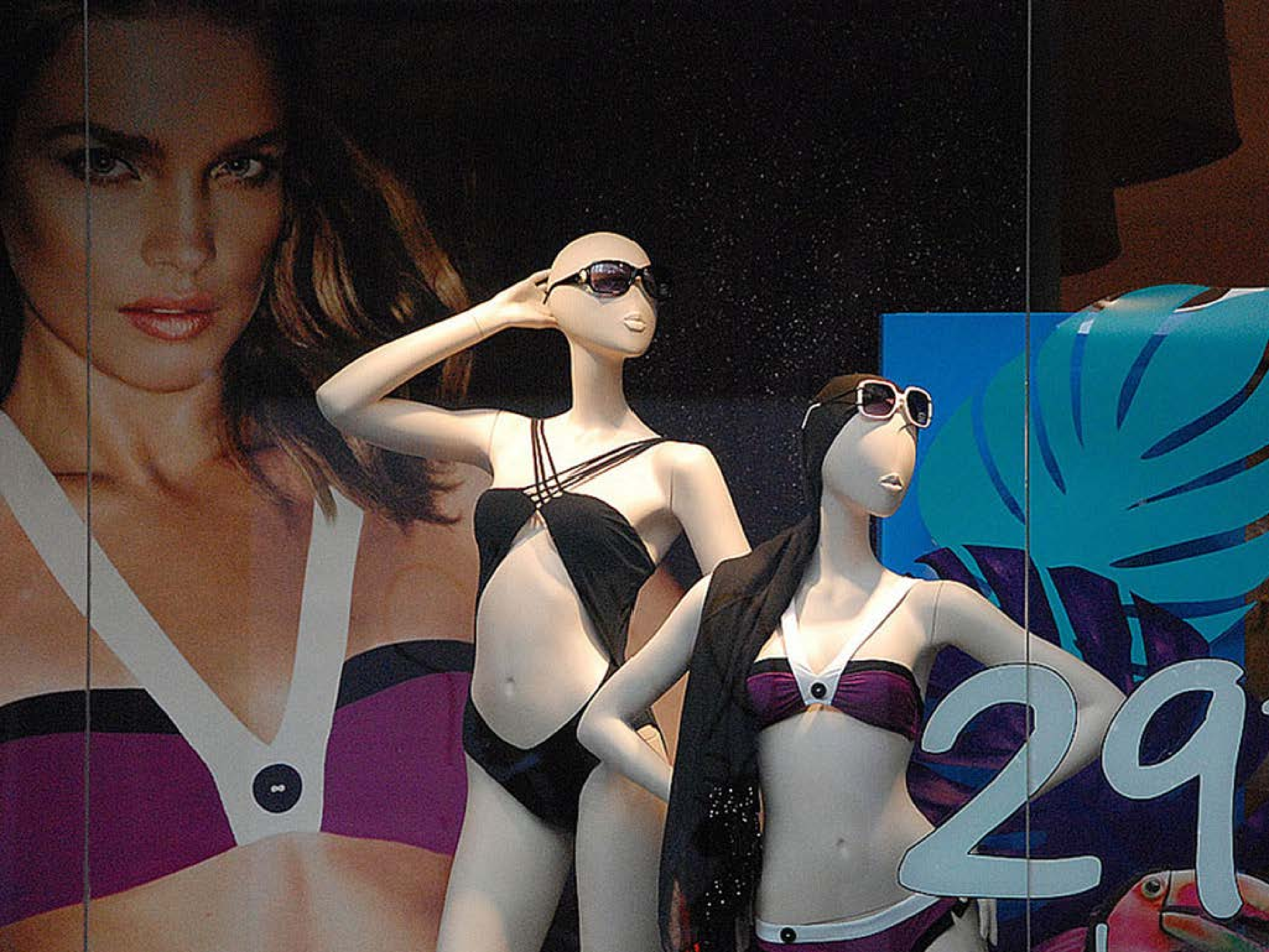
29

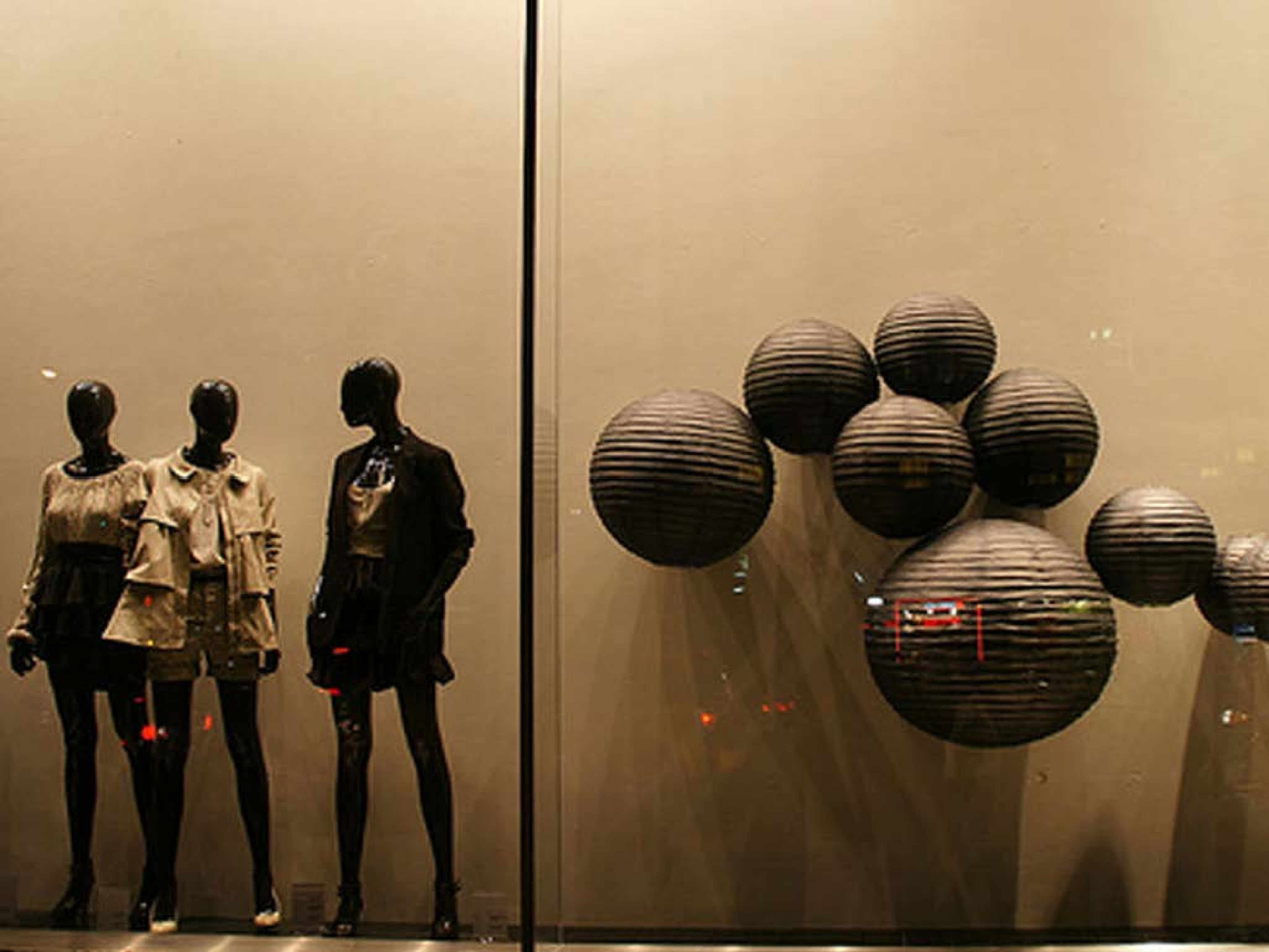

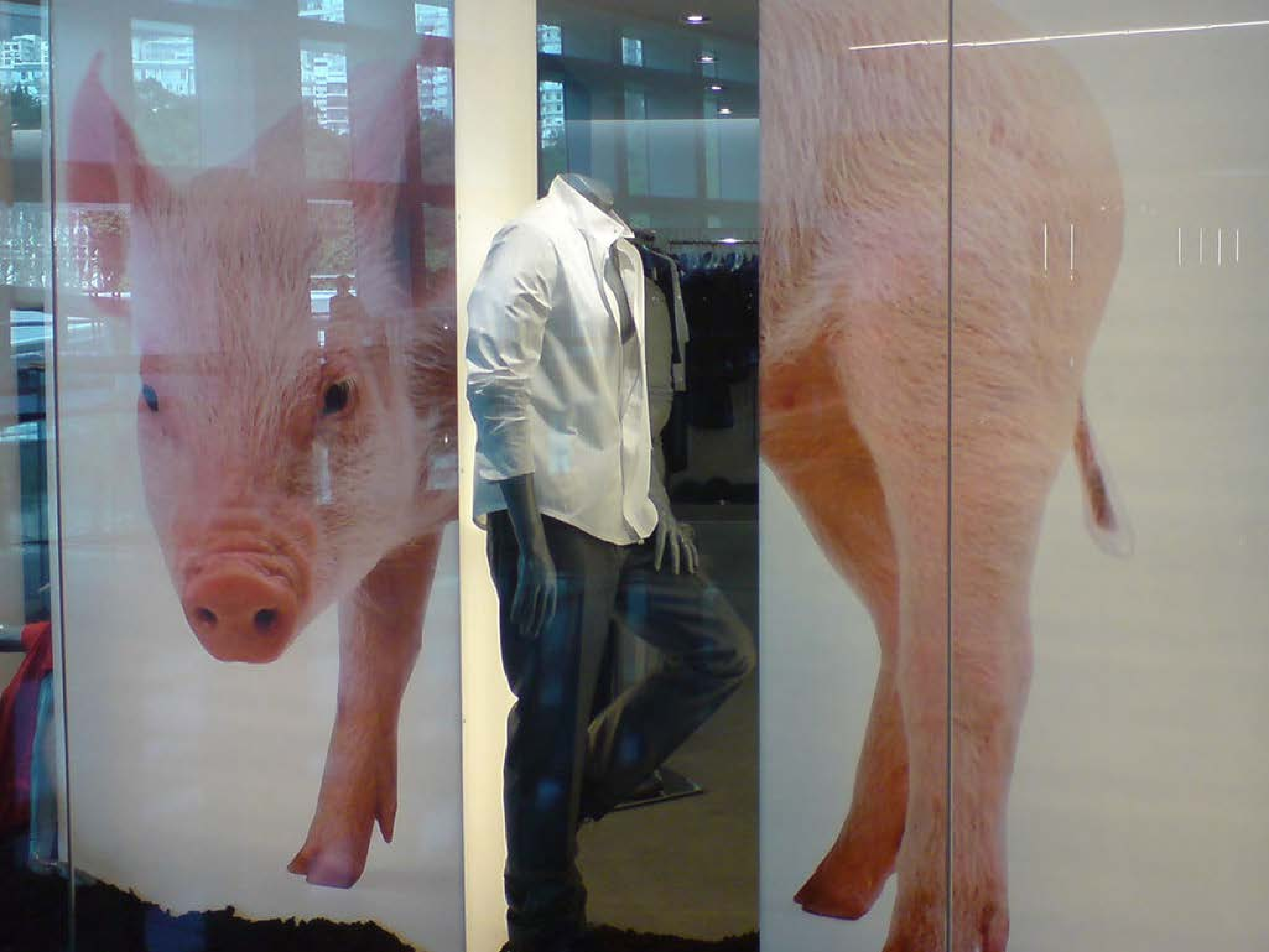

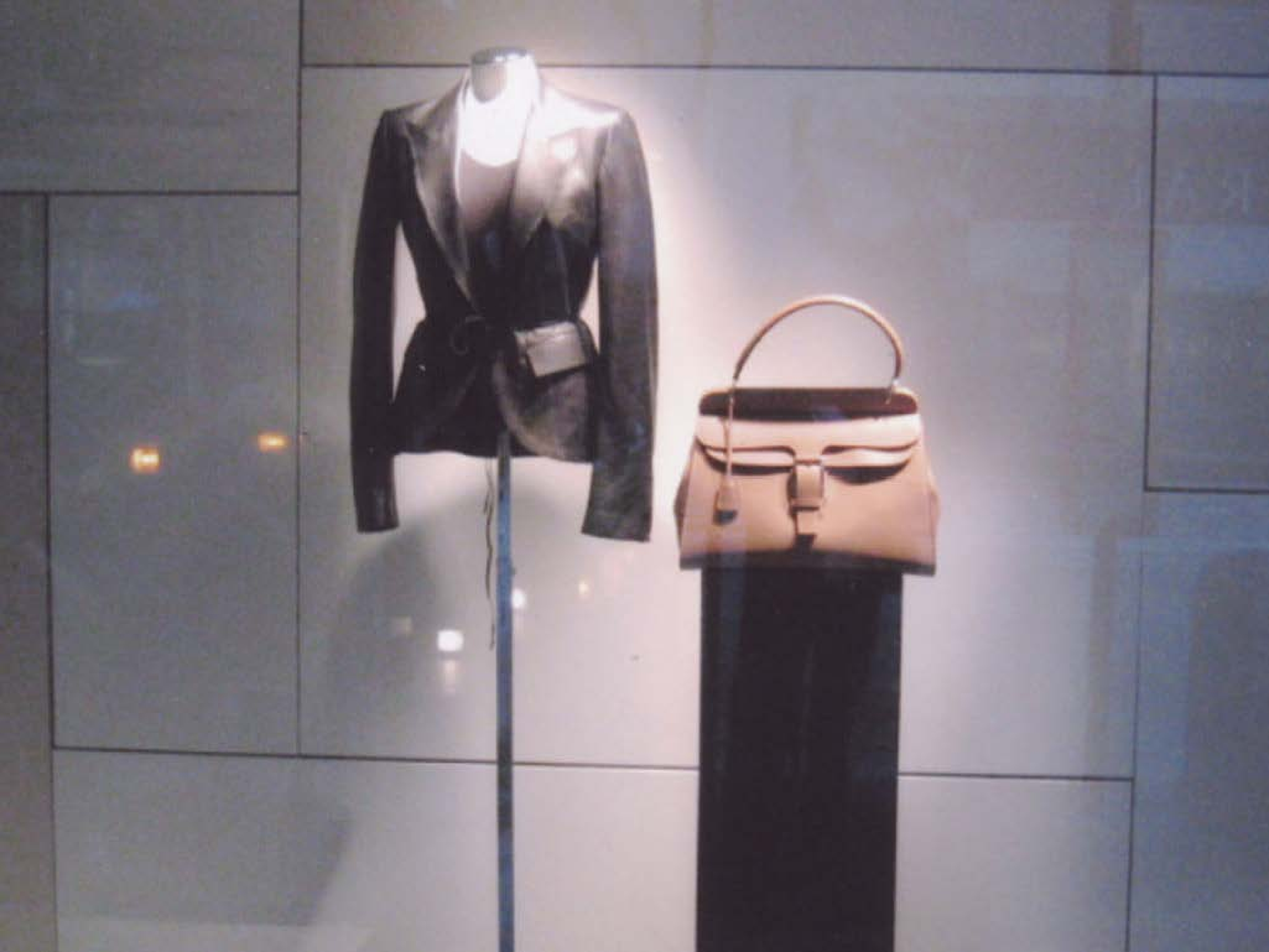

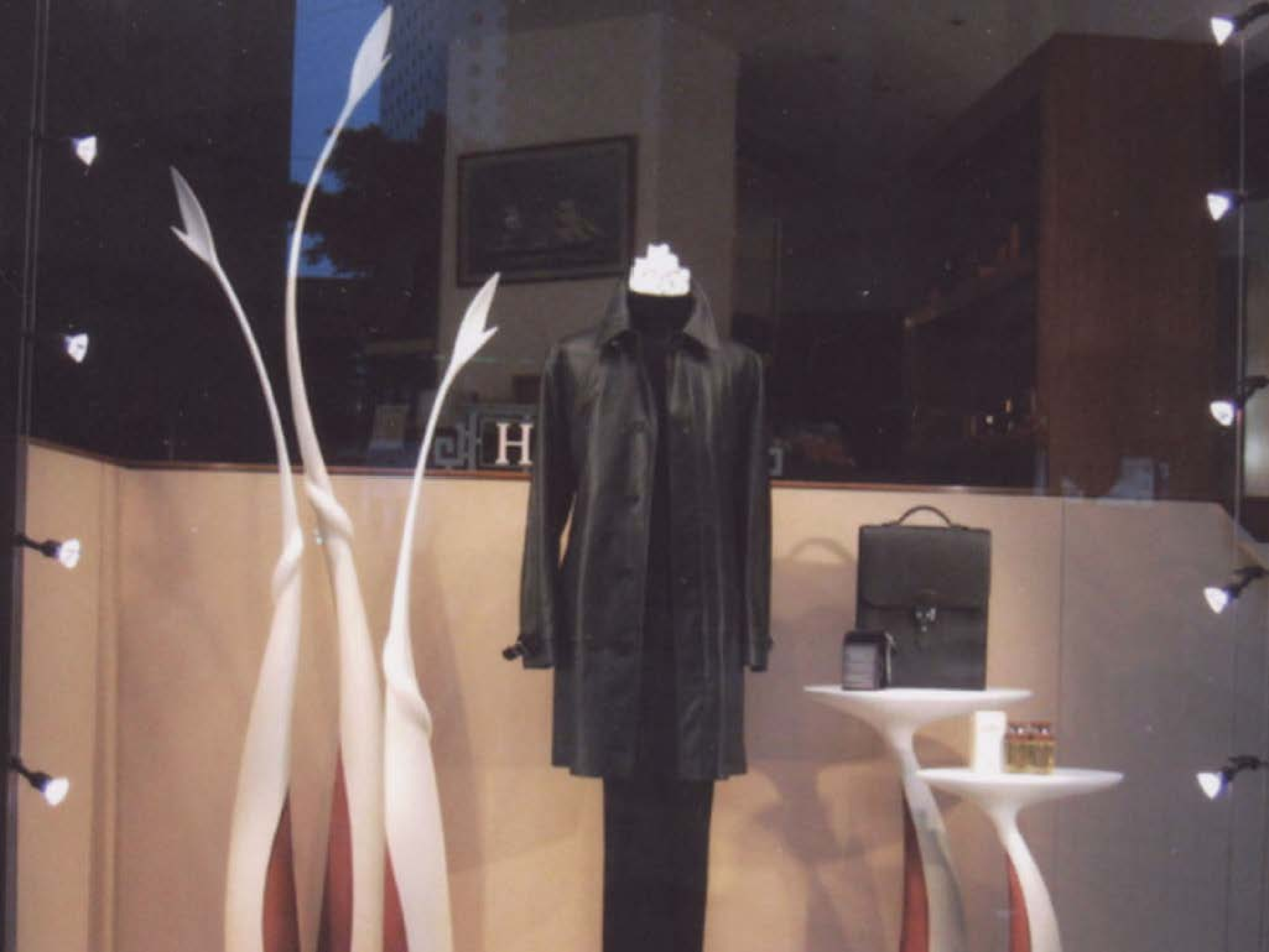

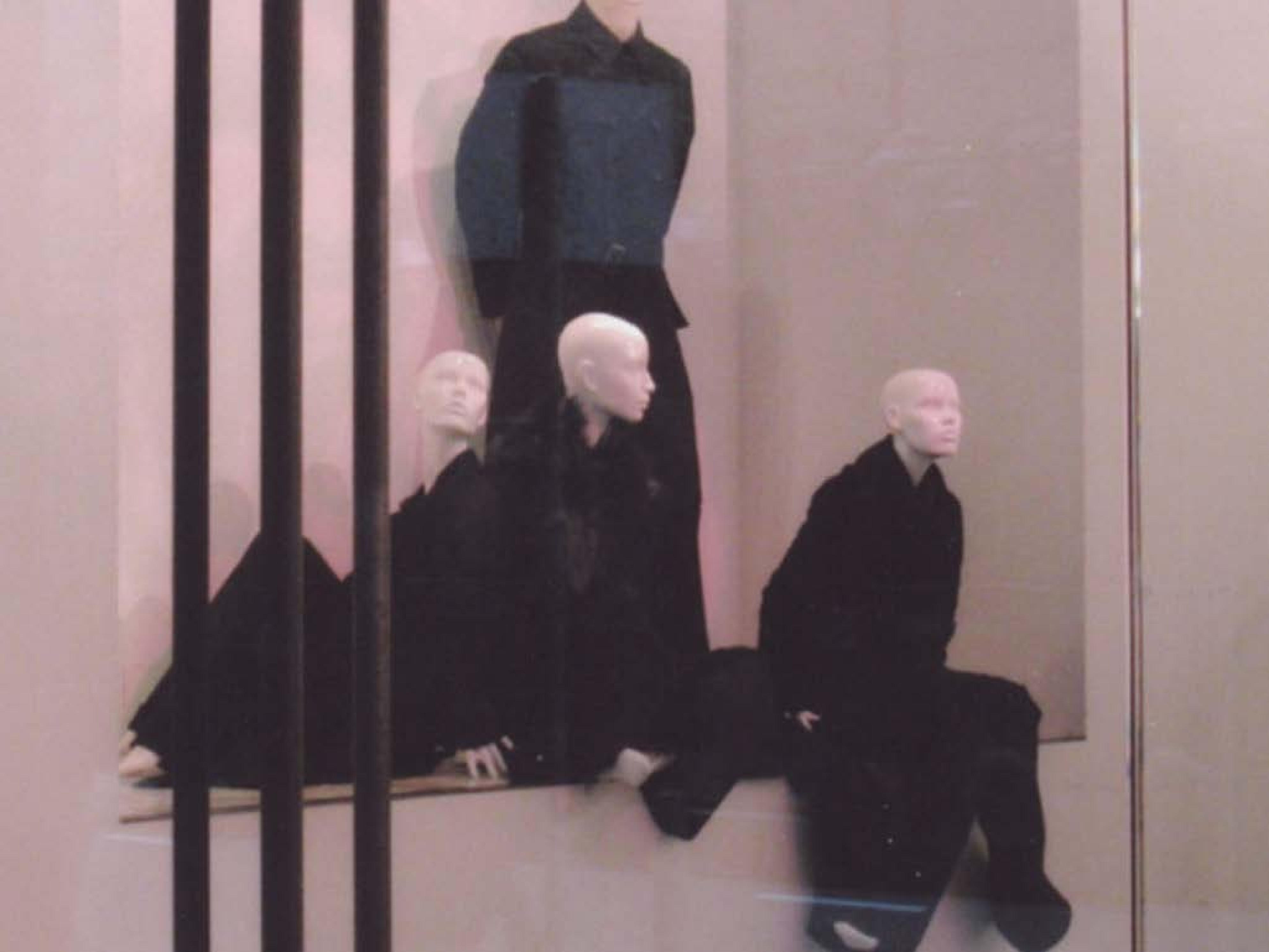

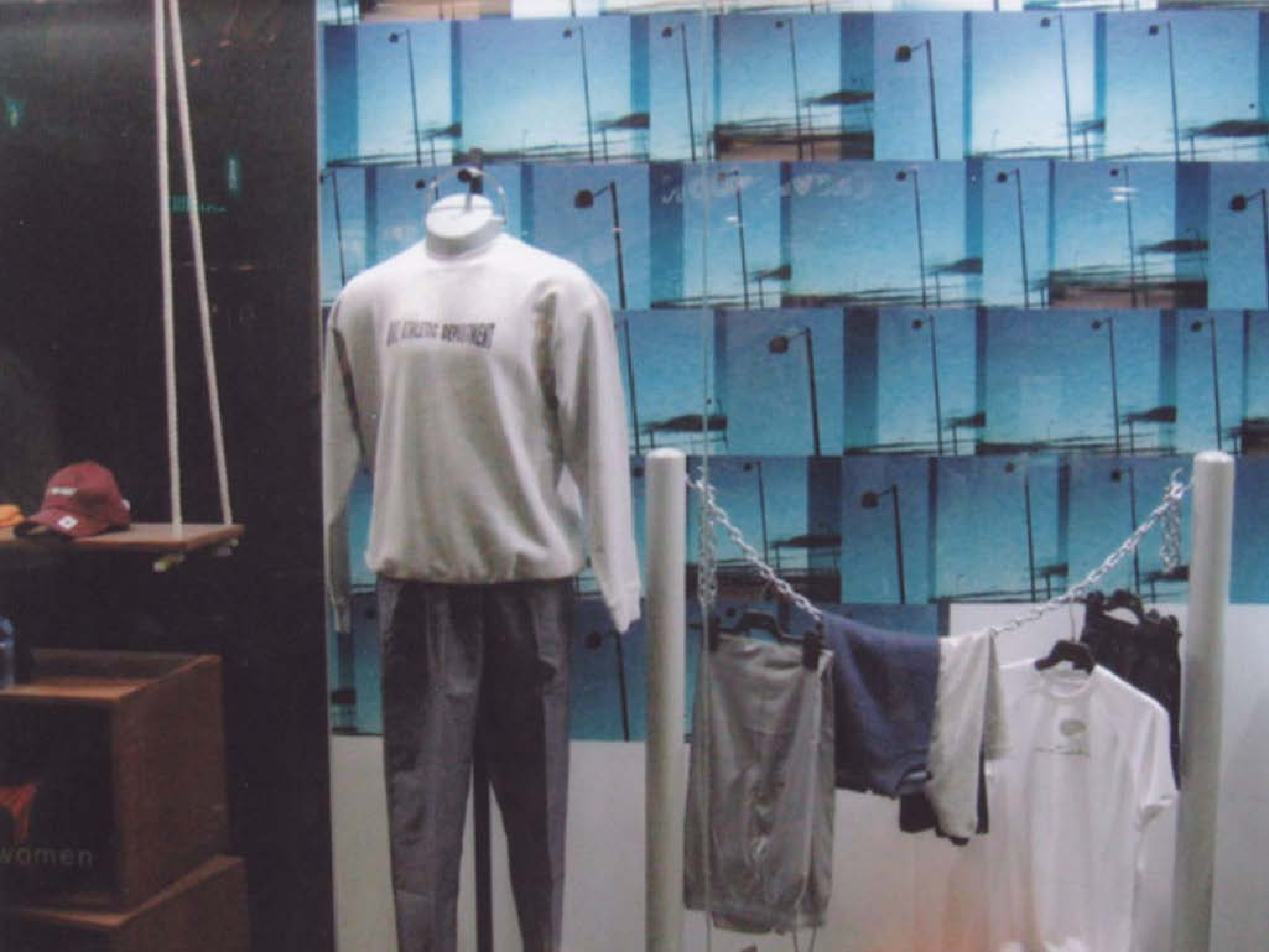
women

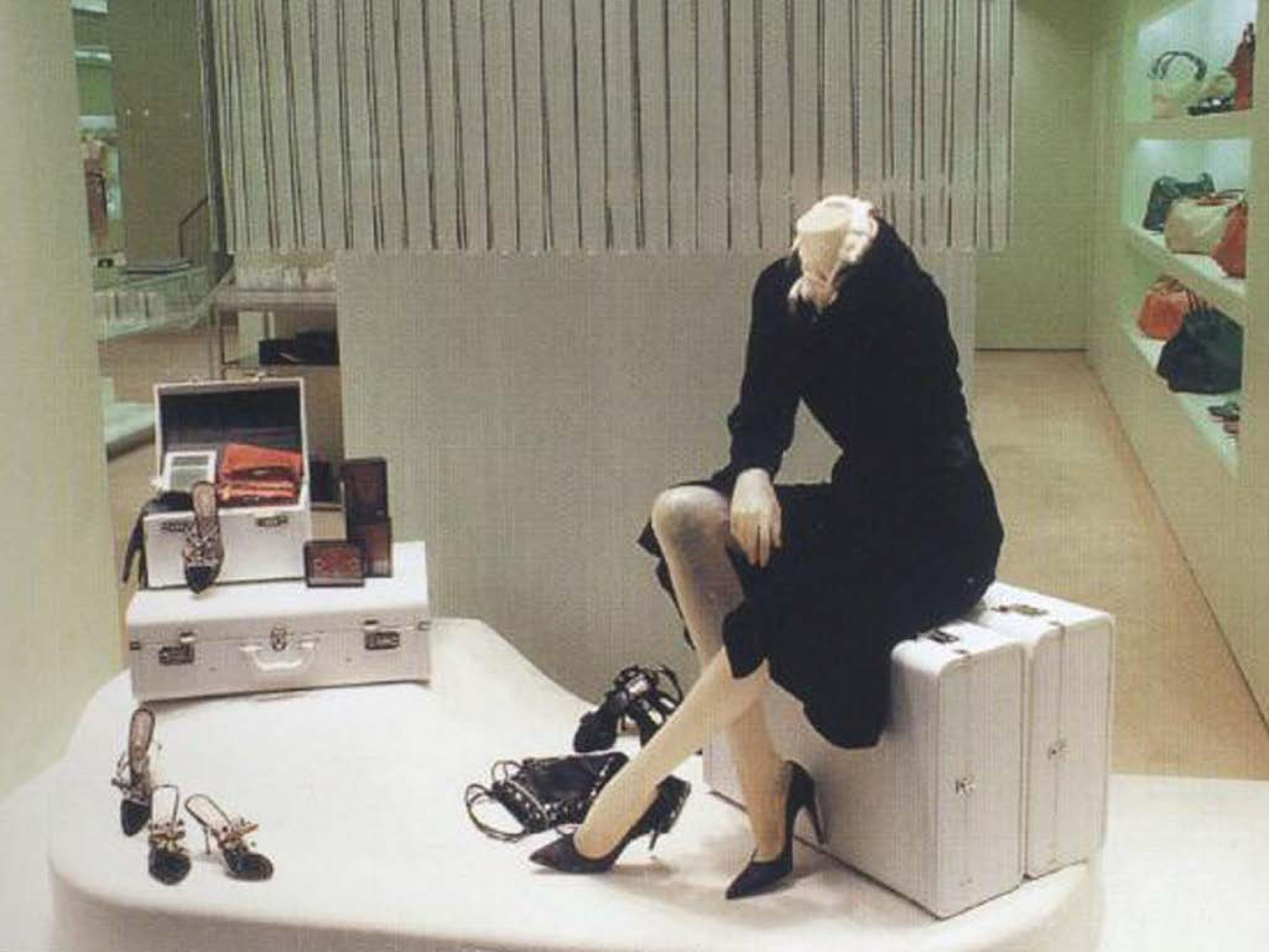

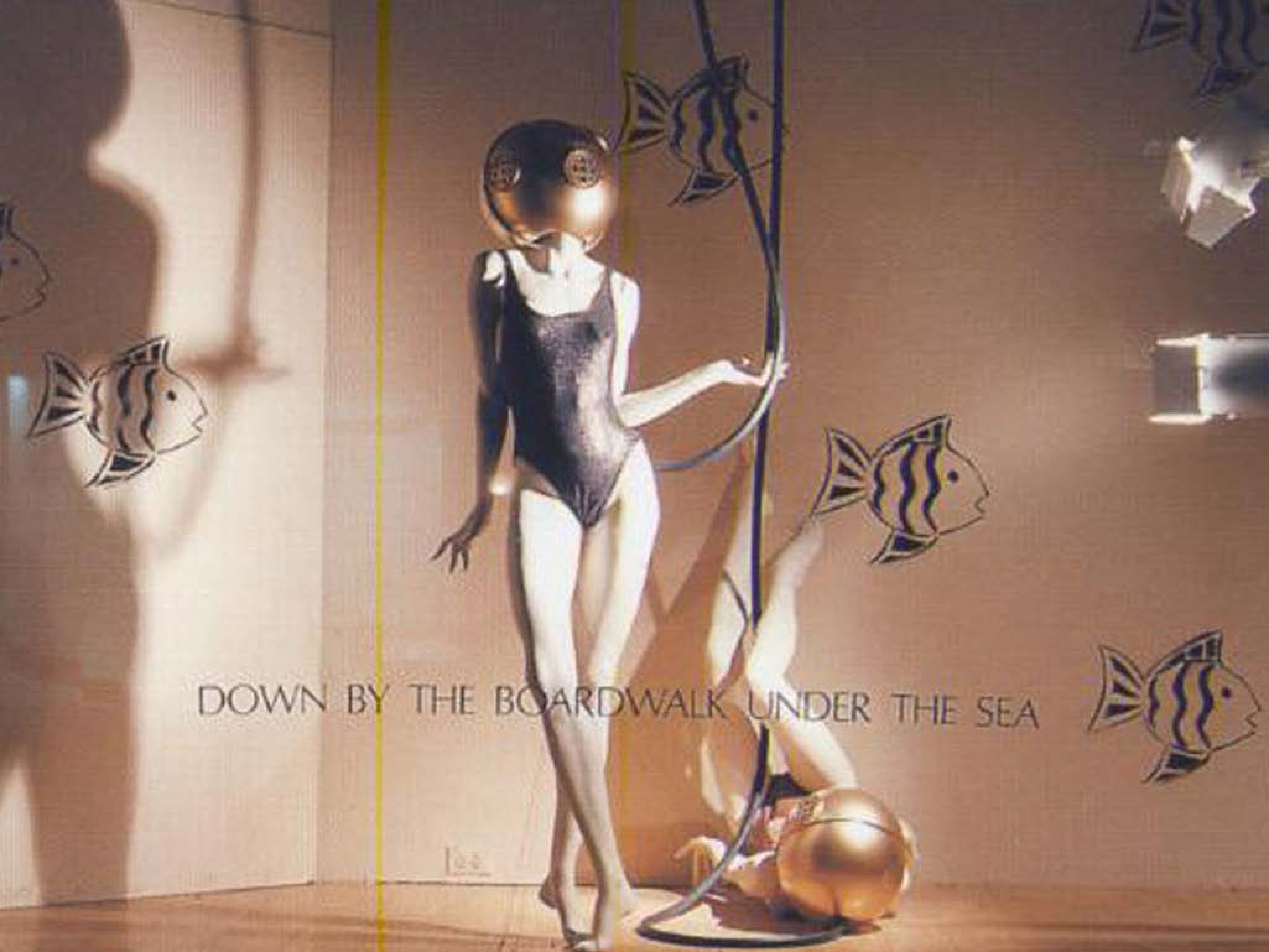
DOWN BY THE BOARDWALK UNDER THE SEA

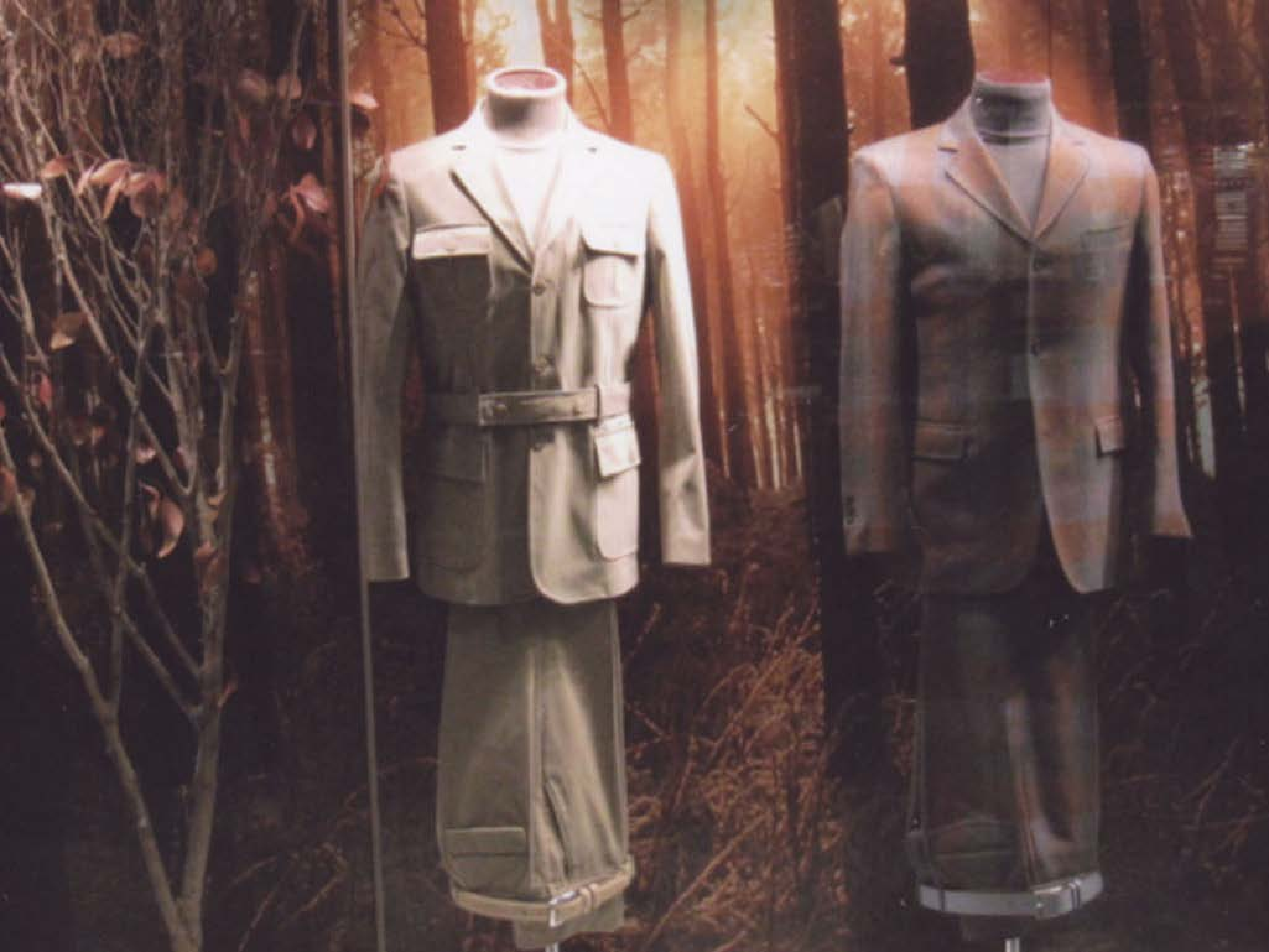

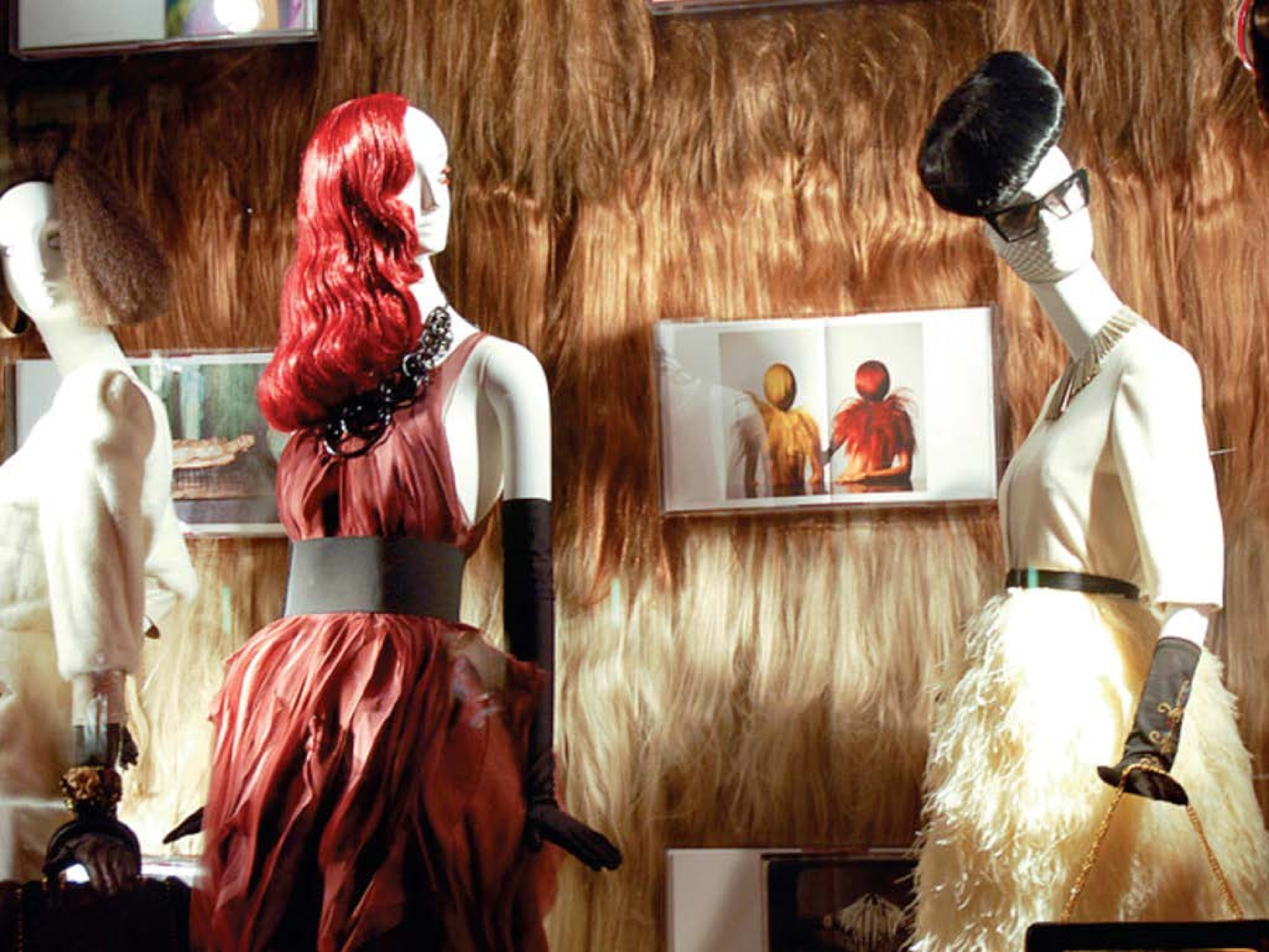

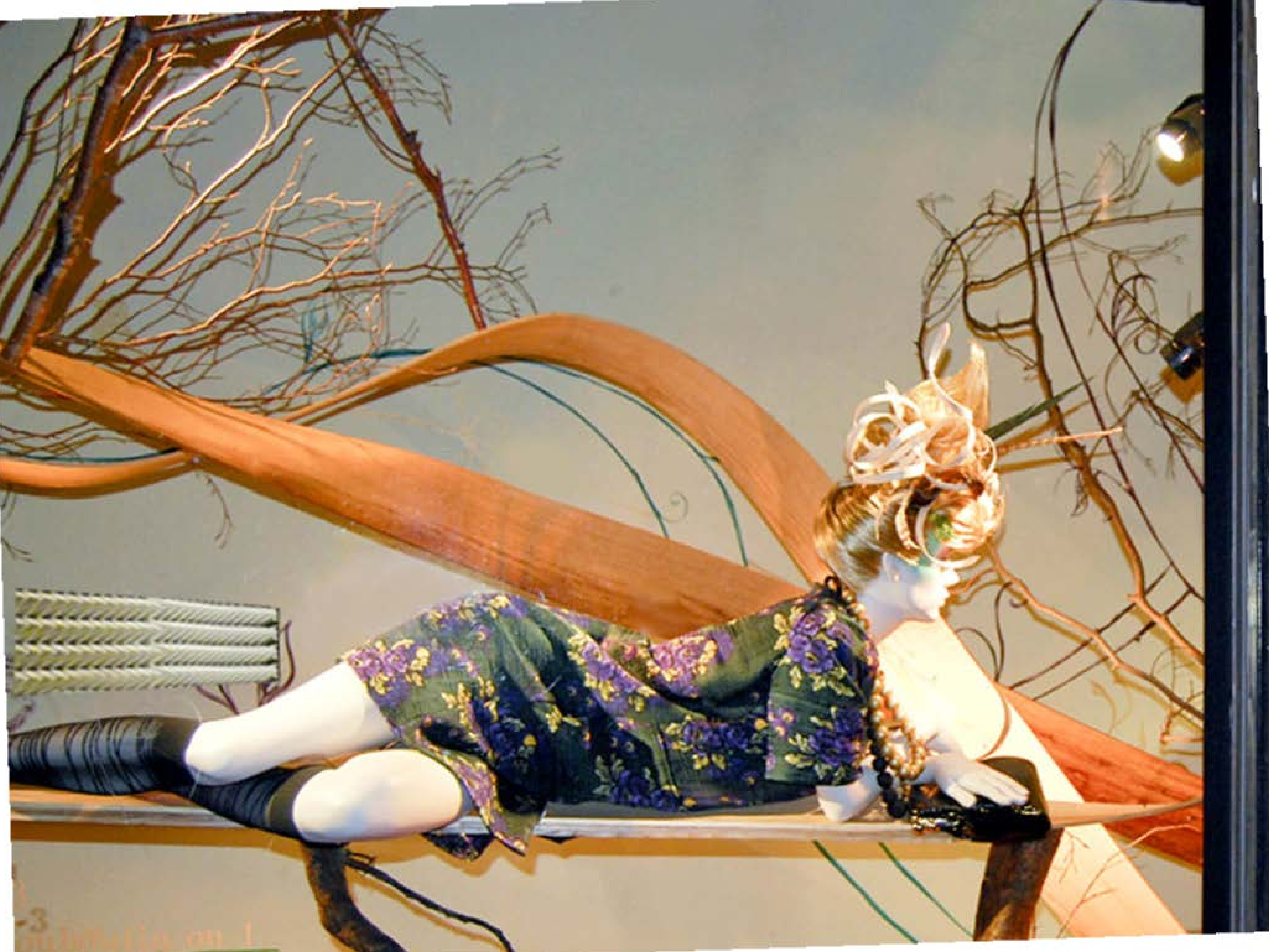

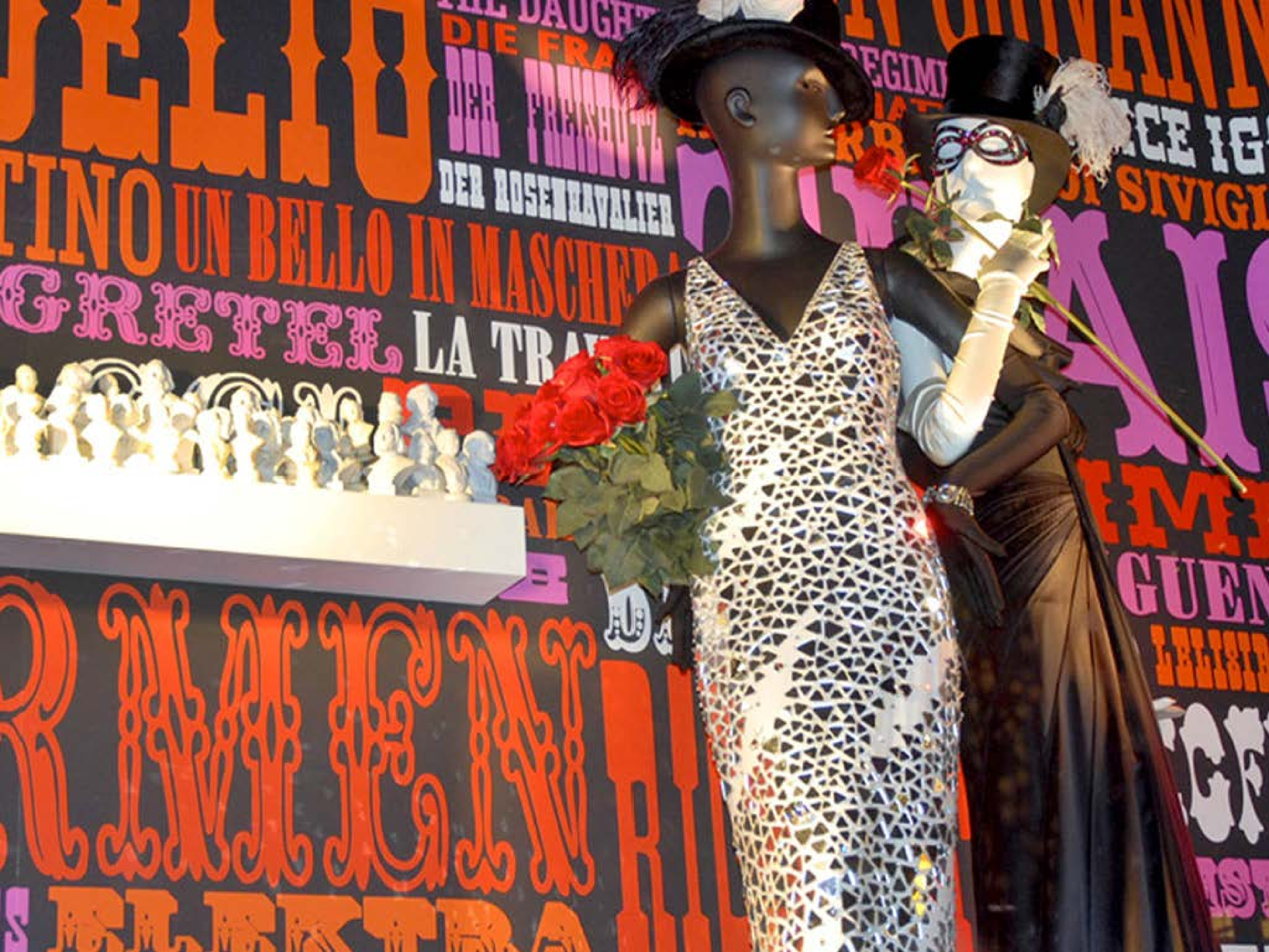
DIE FRA
DER FREISCHUTZ
DER ROSENKAVALIER
UN BELLO IN MASCHERA
GRETEL
LA TRAV

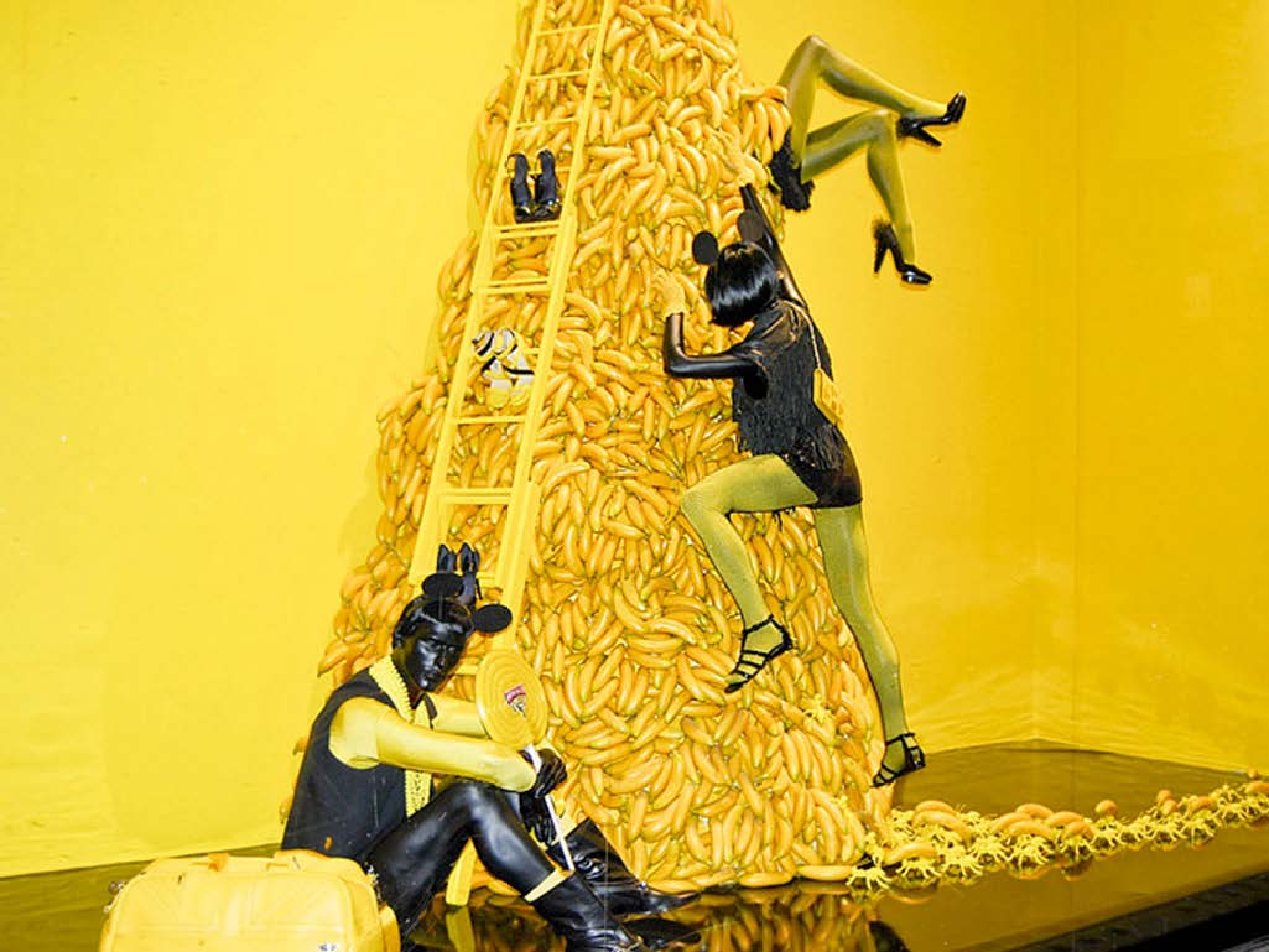

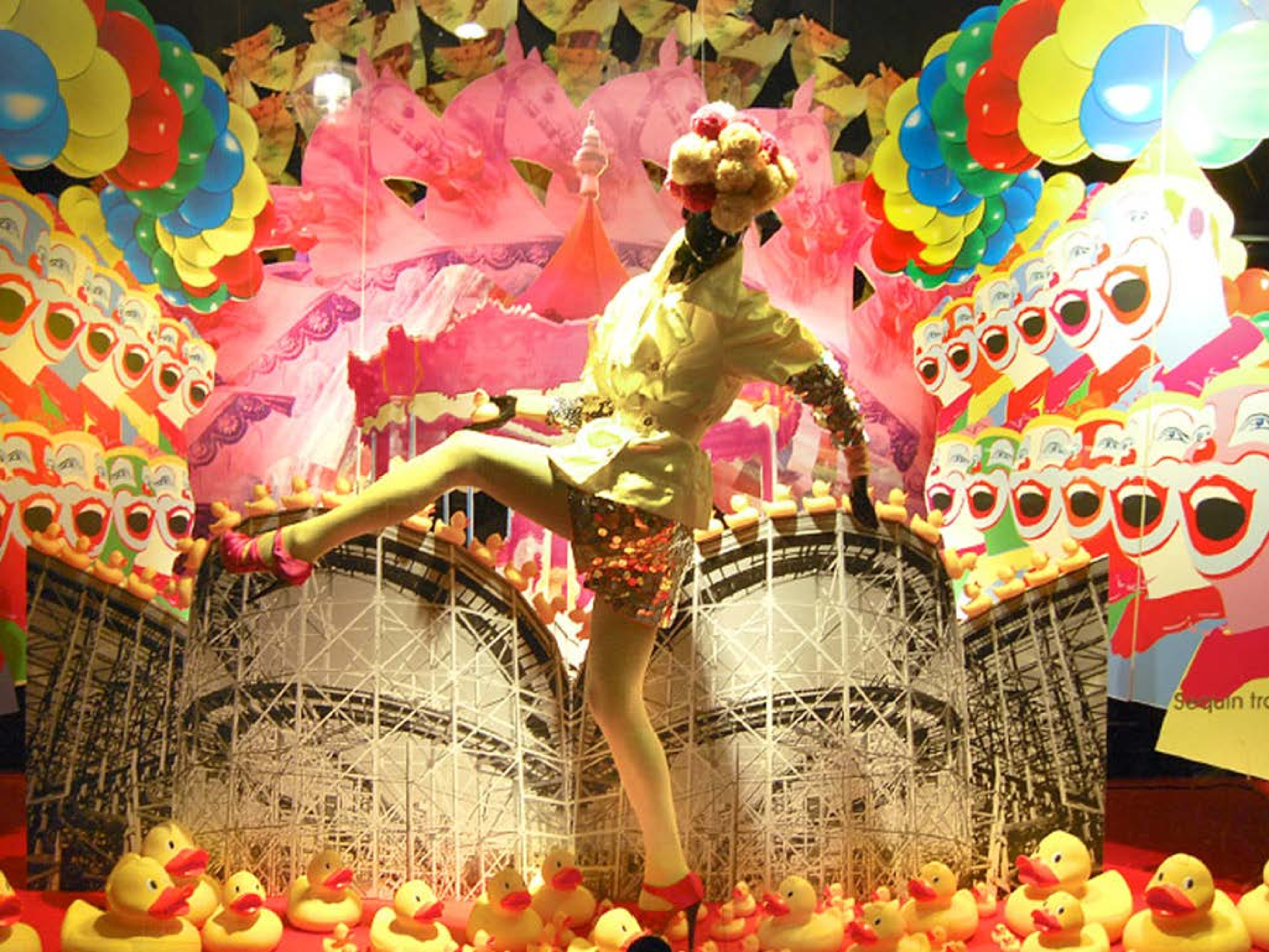
Sequin tro

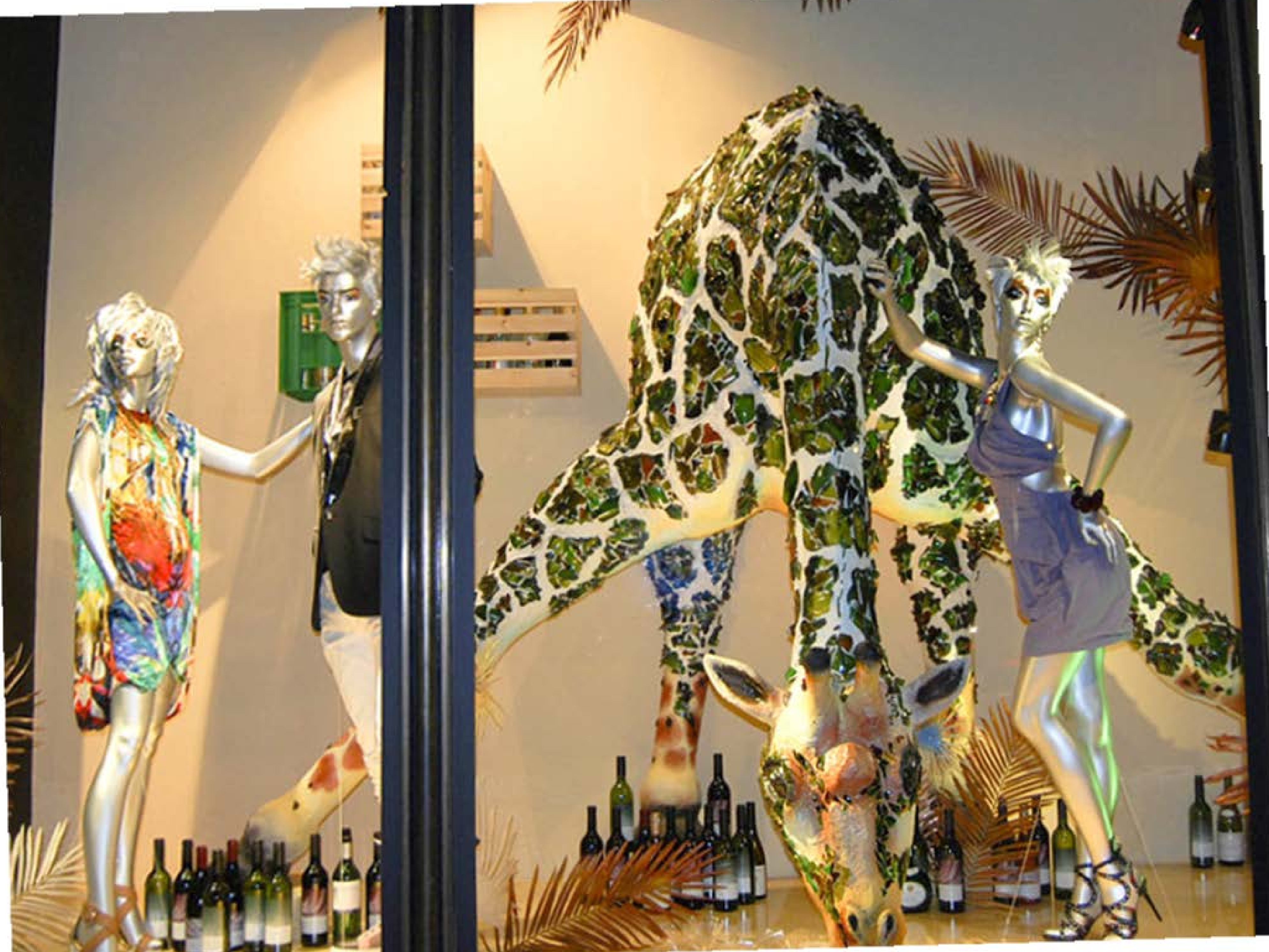

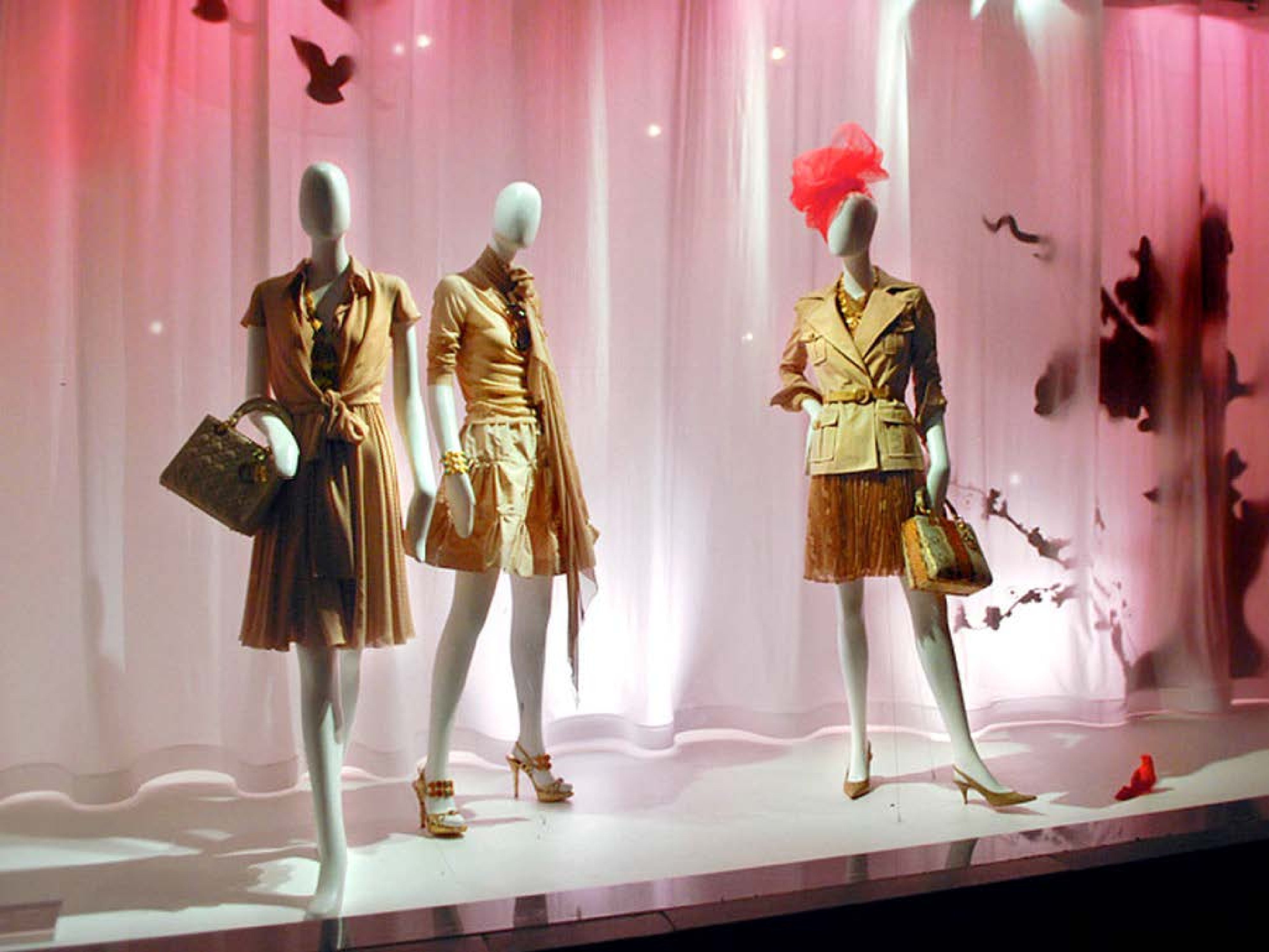

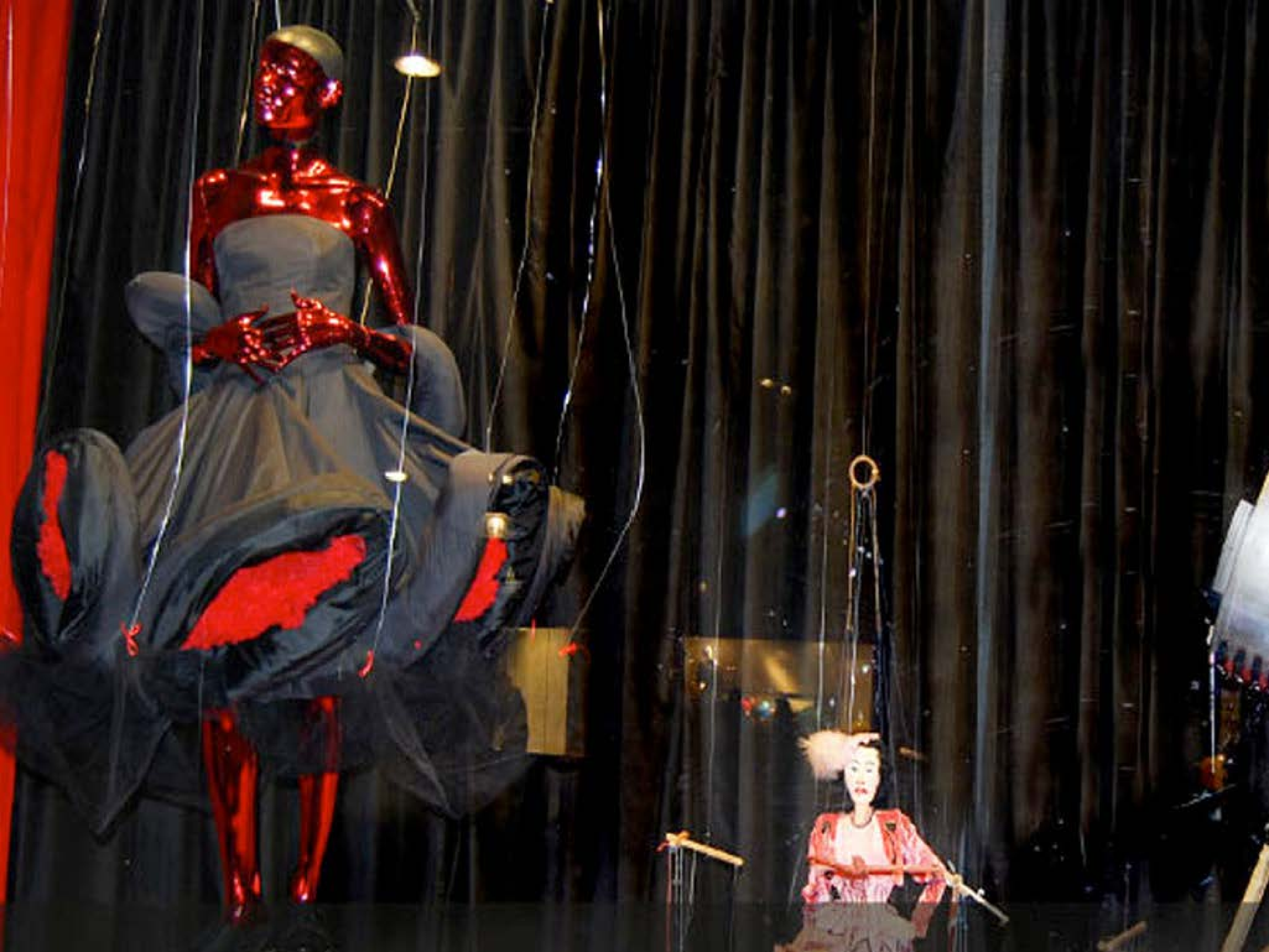

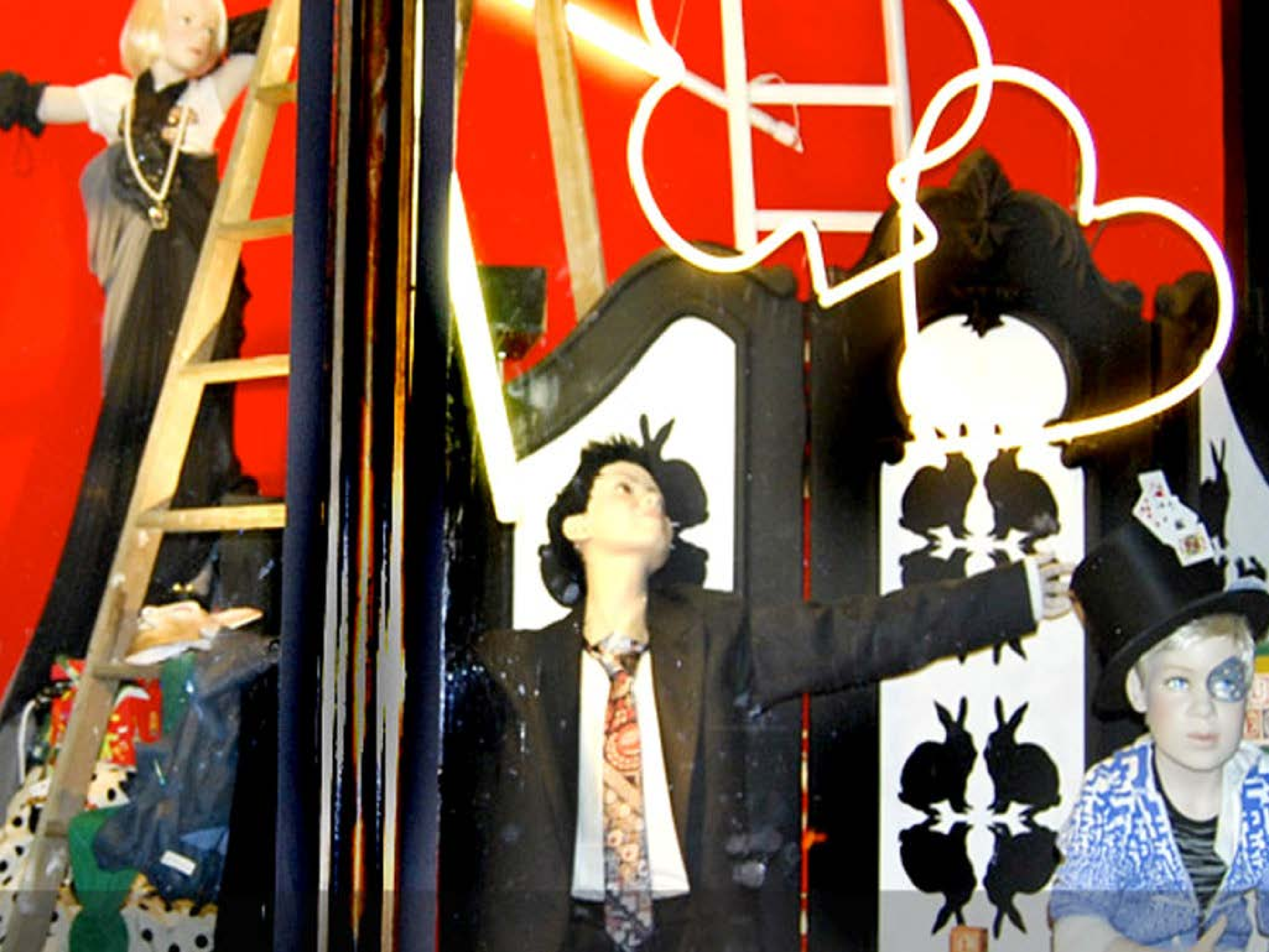

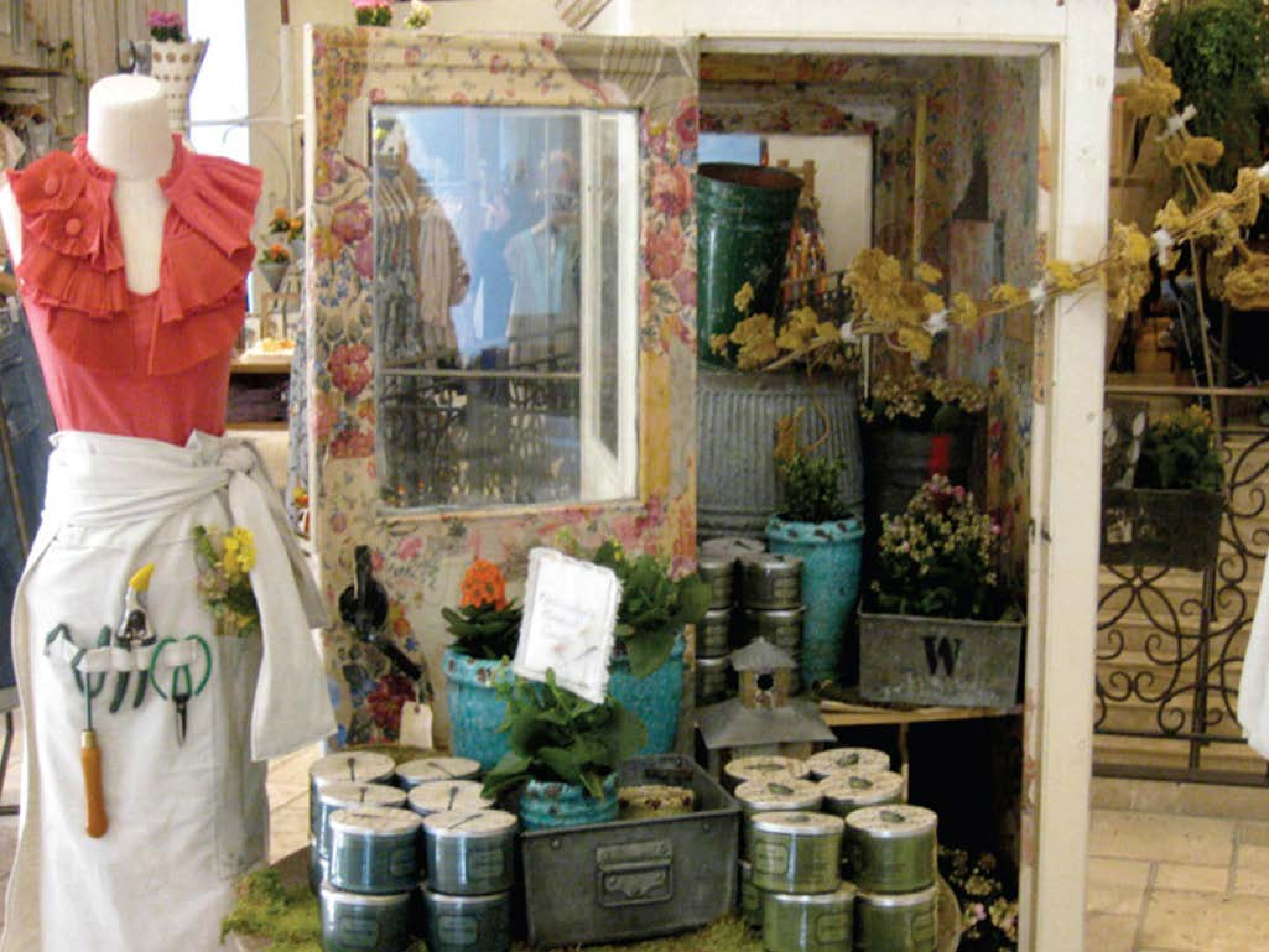

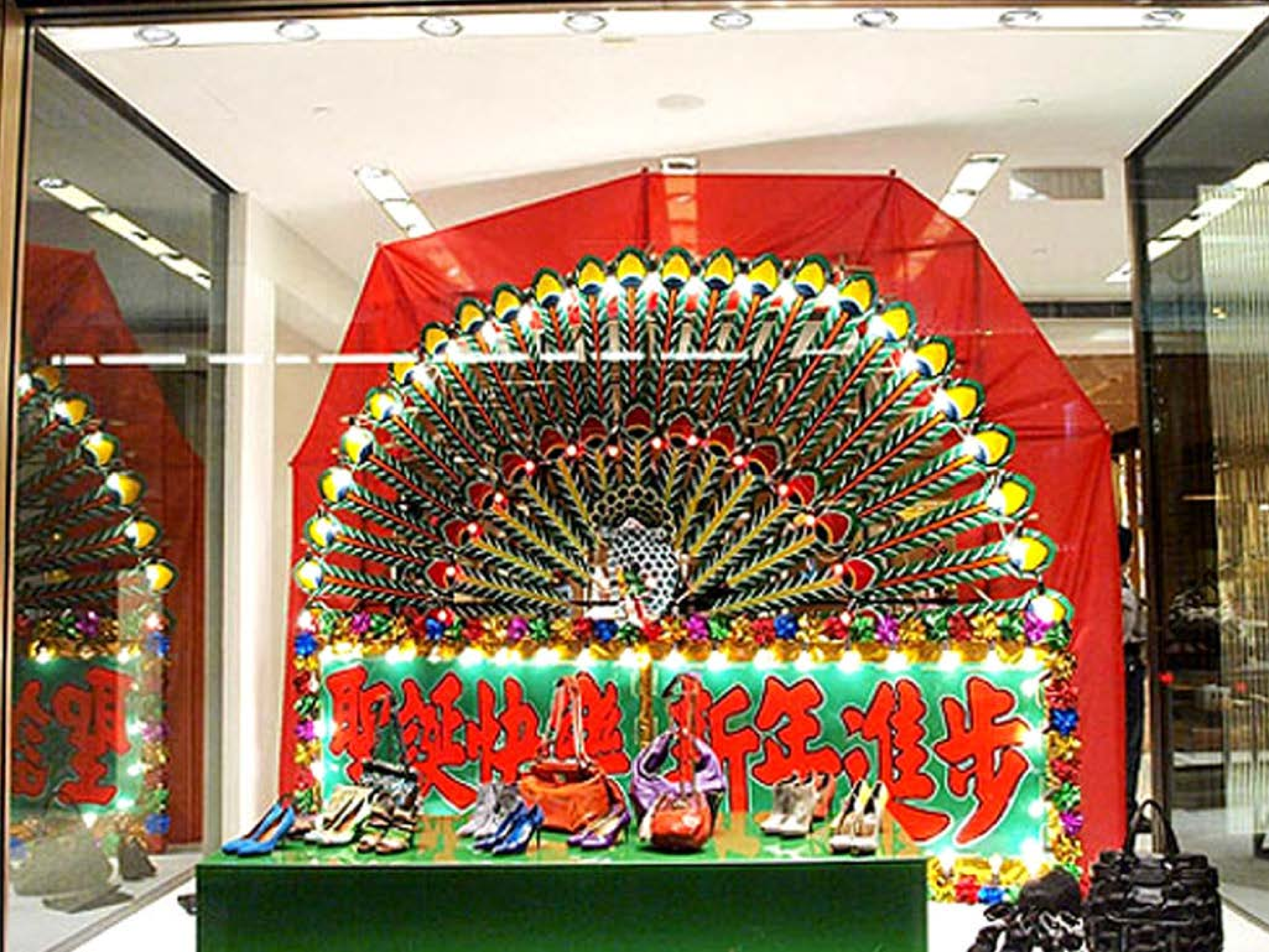
聖誕快樂 新年進步

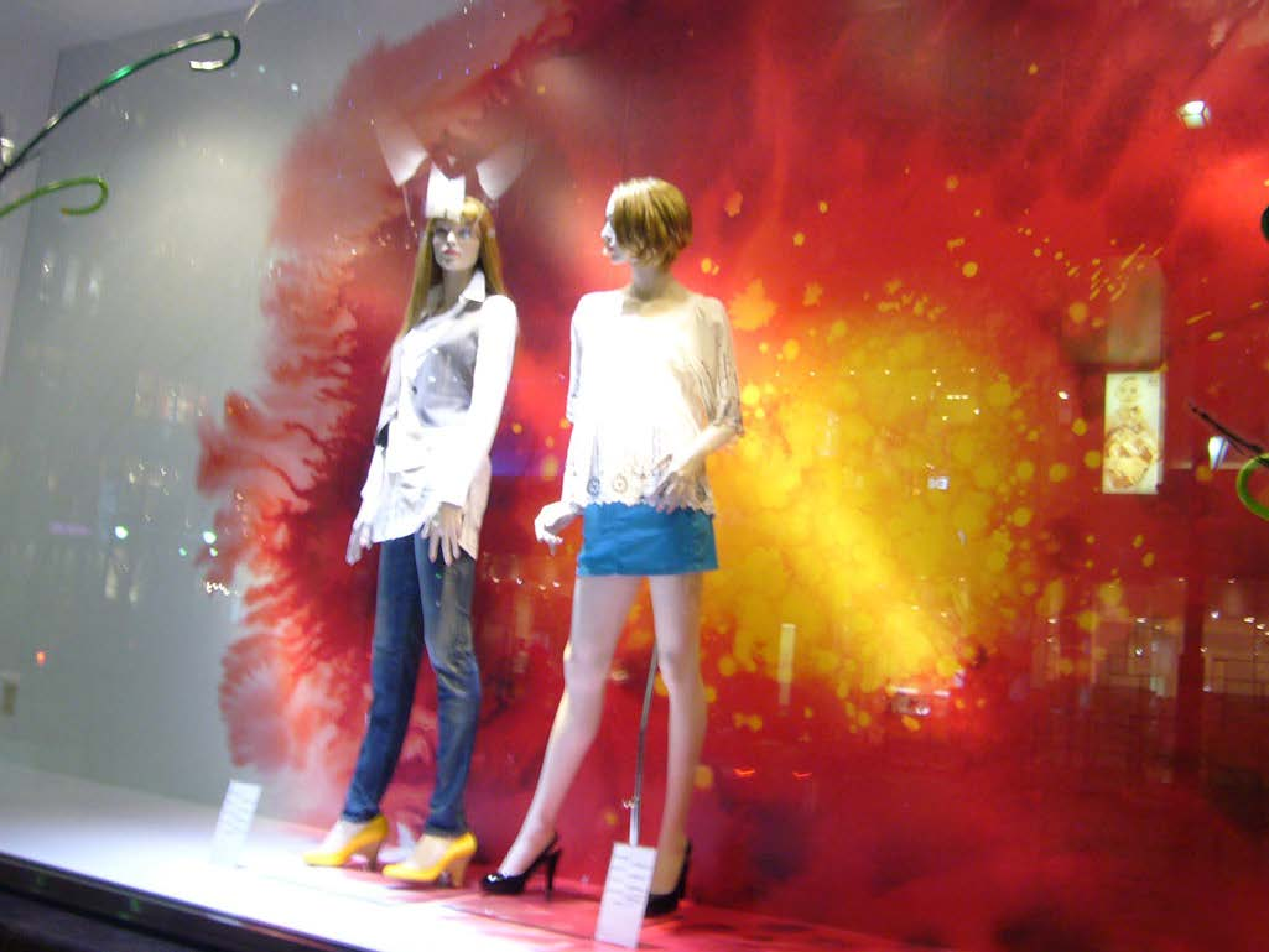

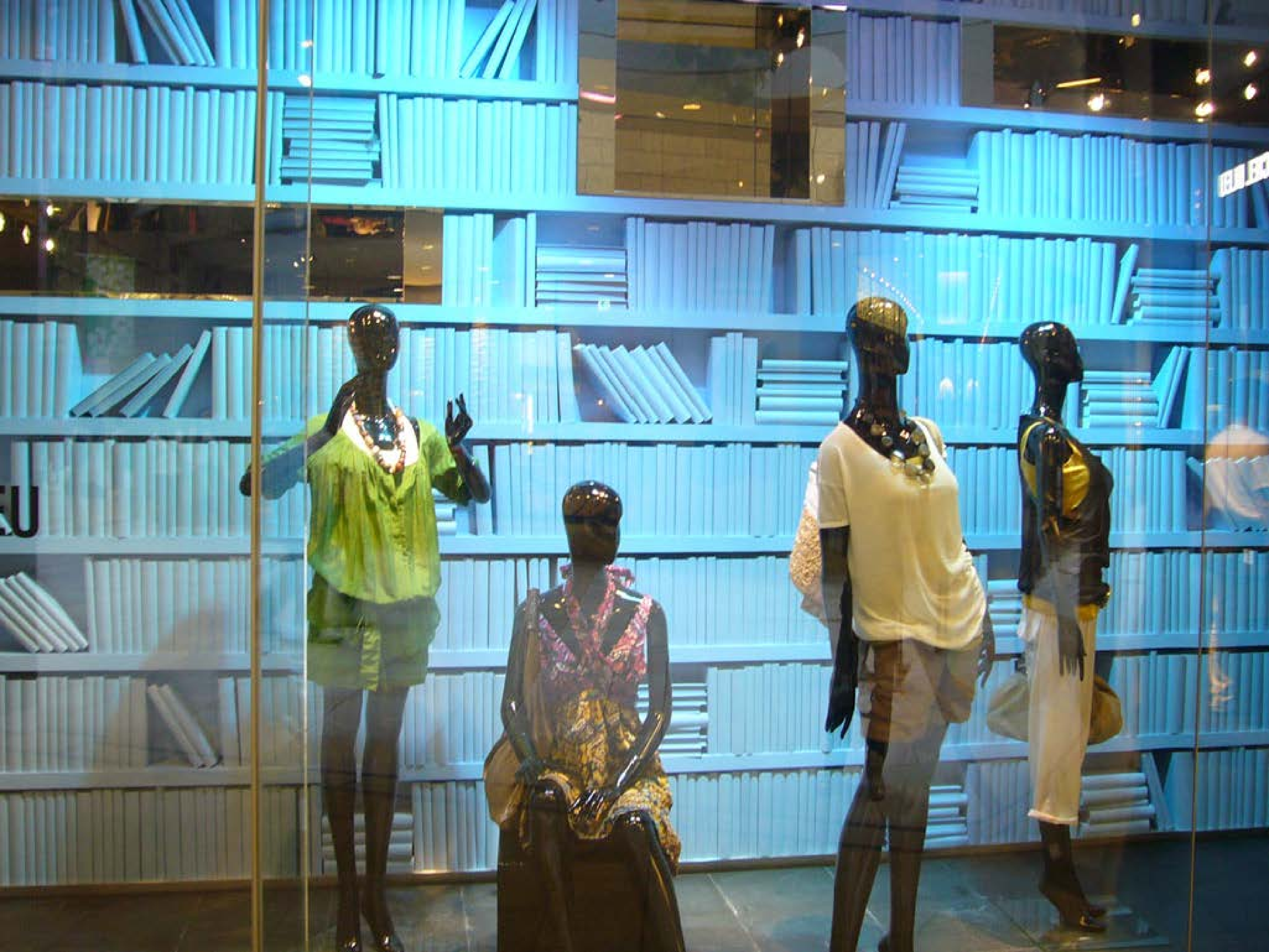

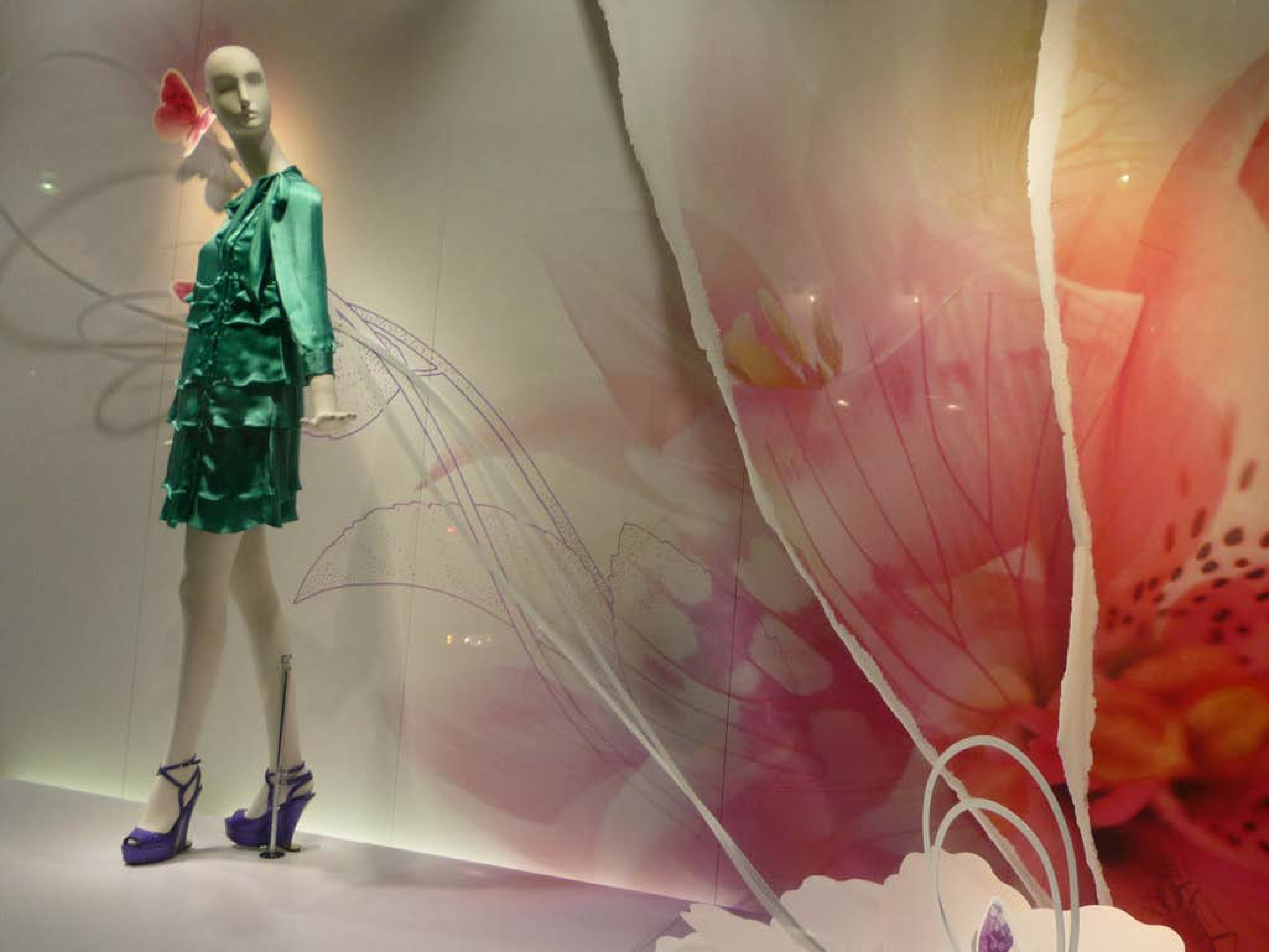

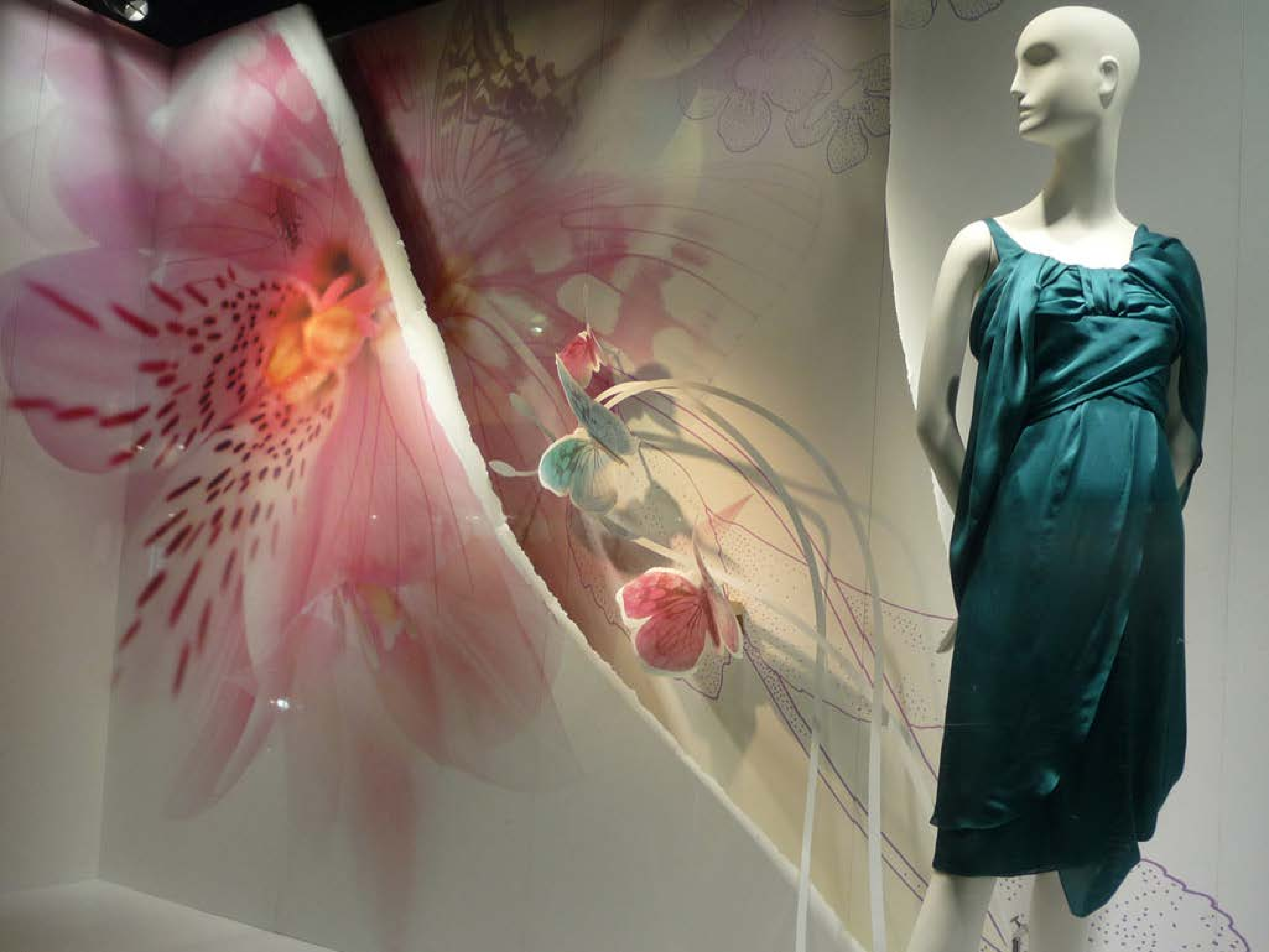

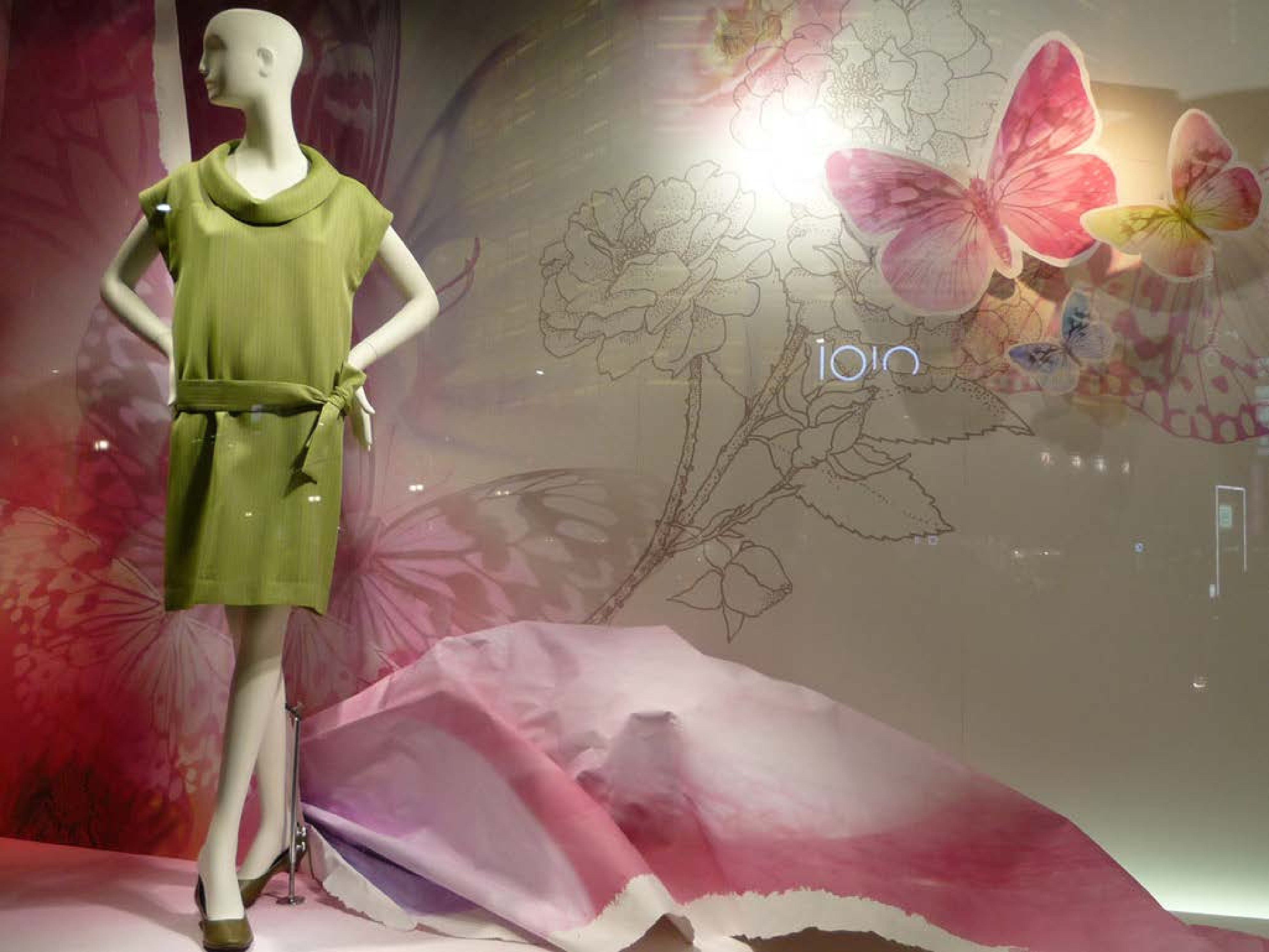
ioio

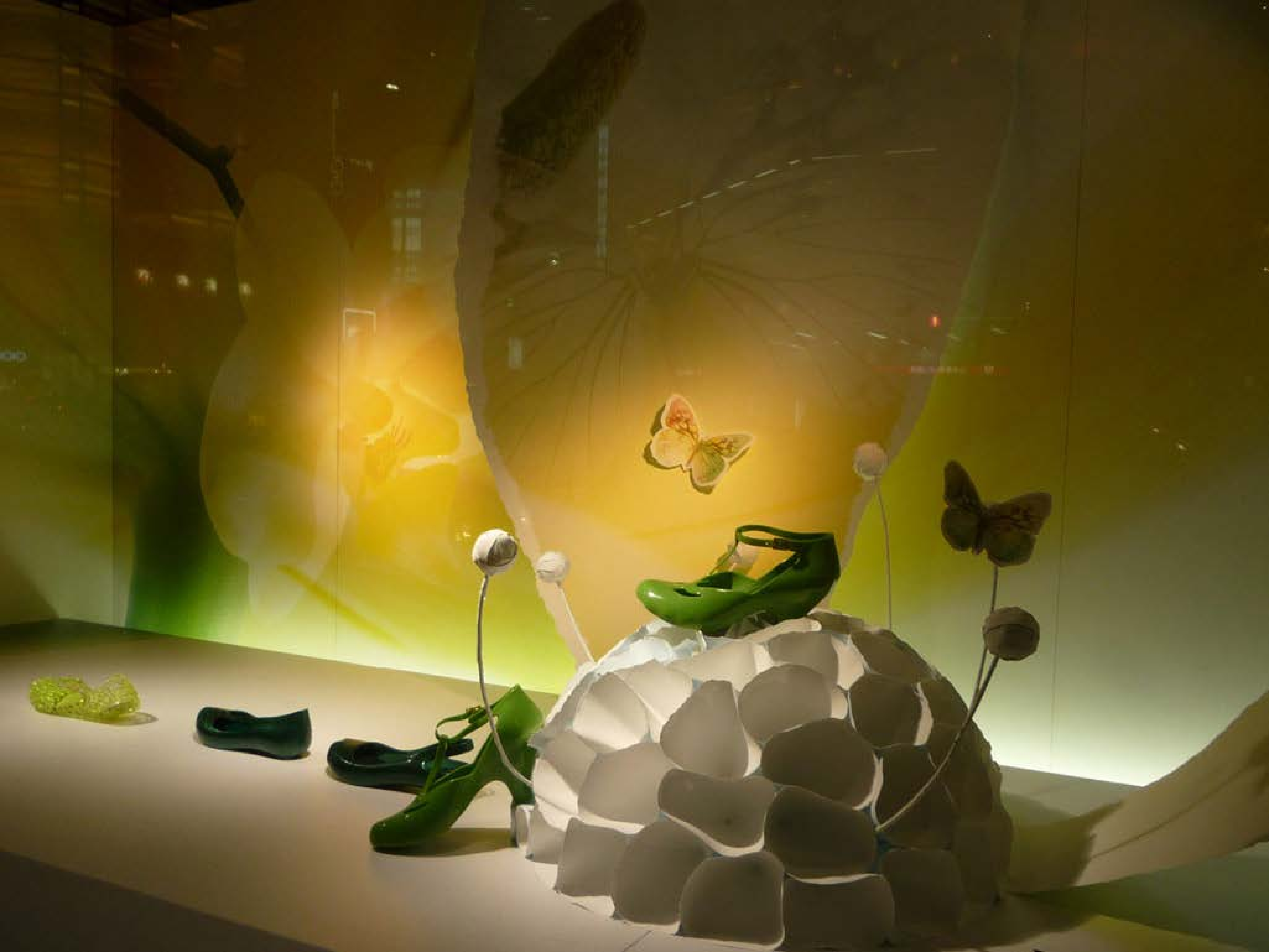

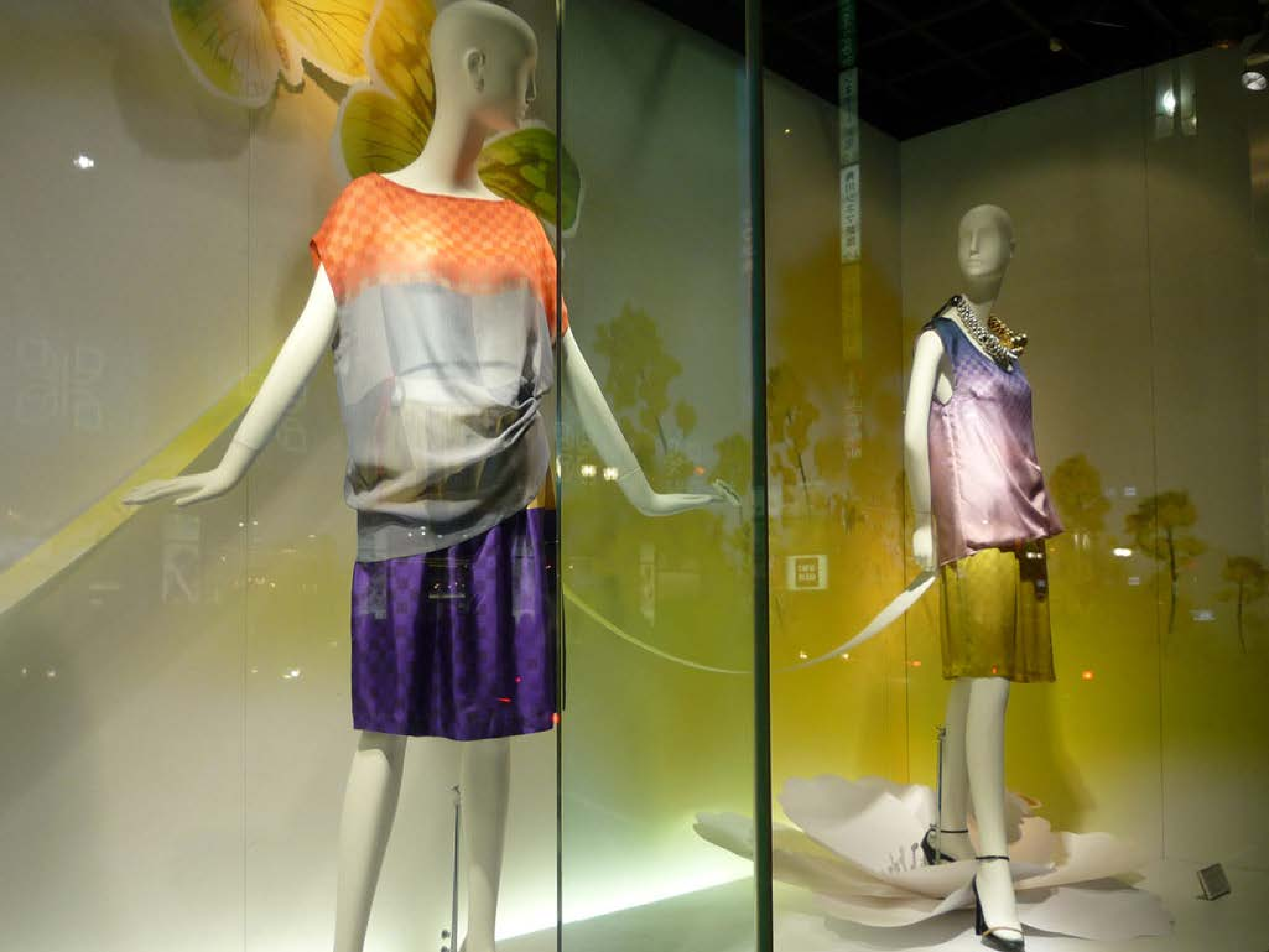

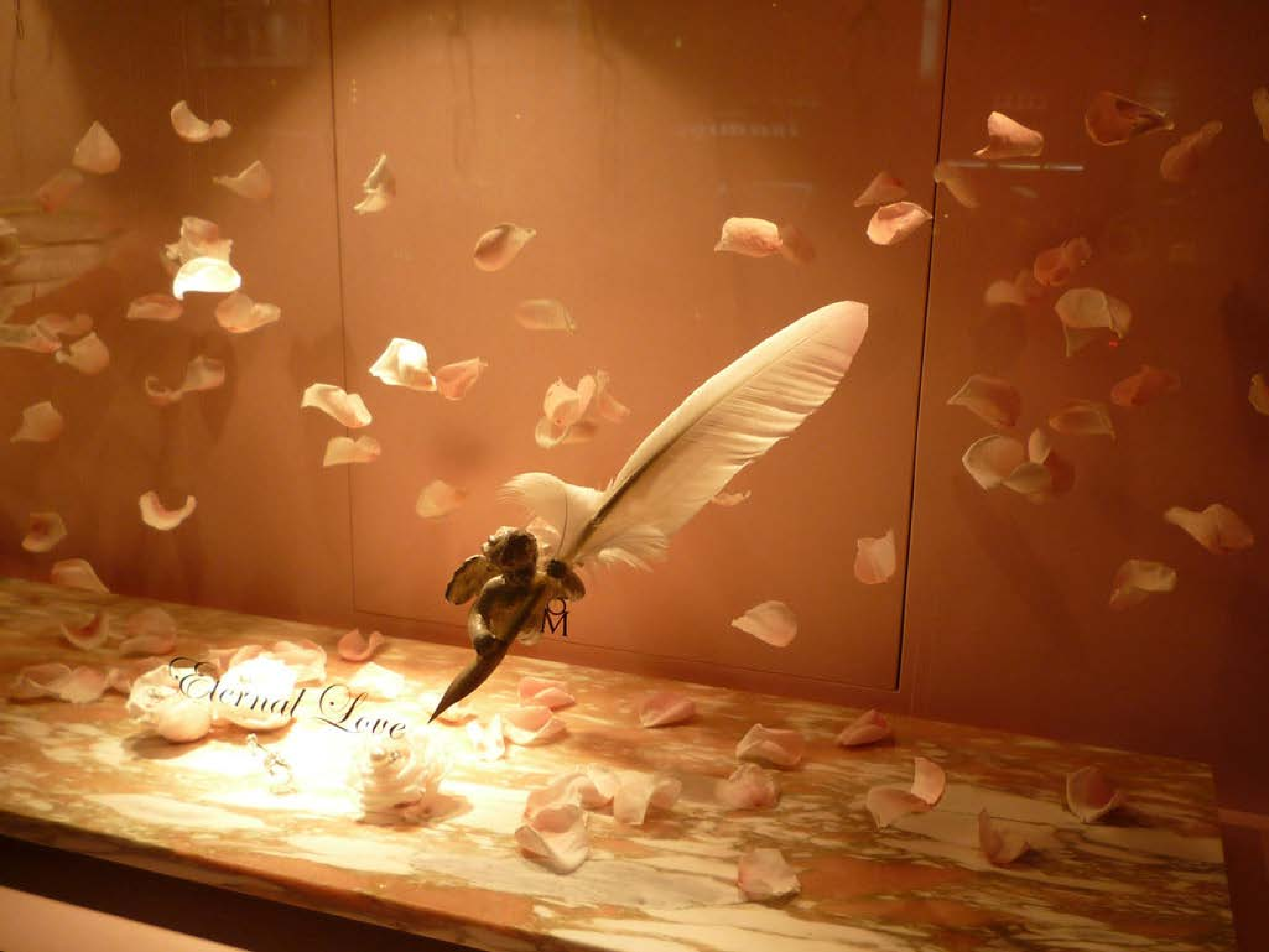
Eternal Love

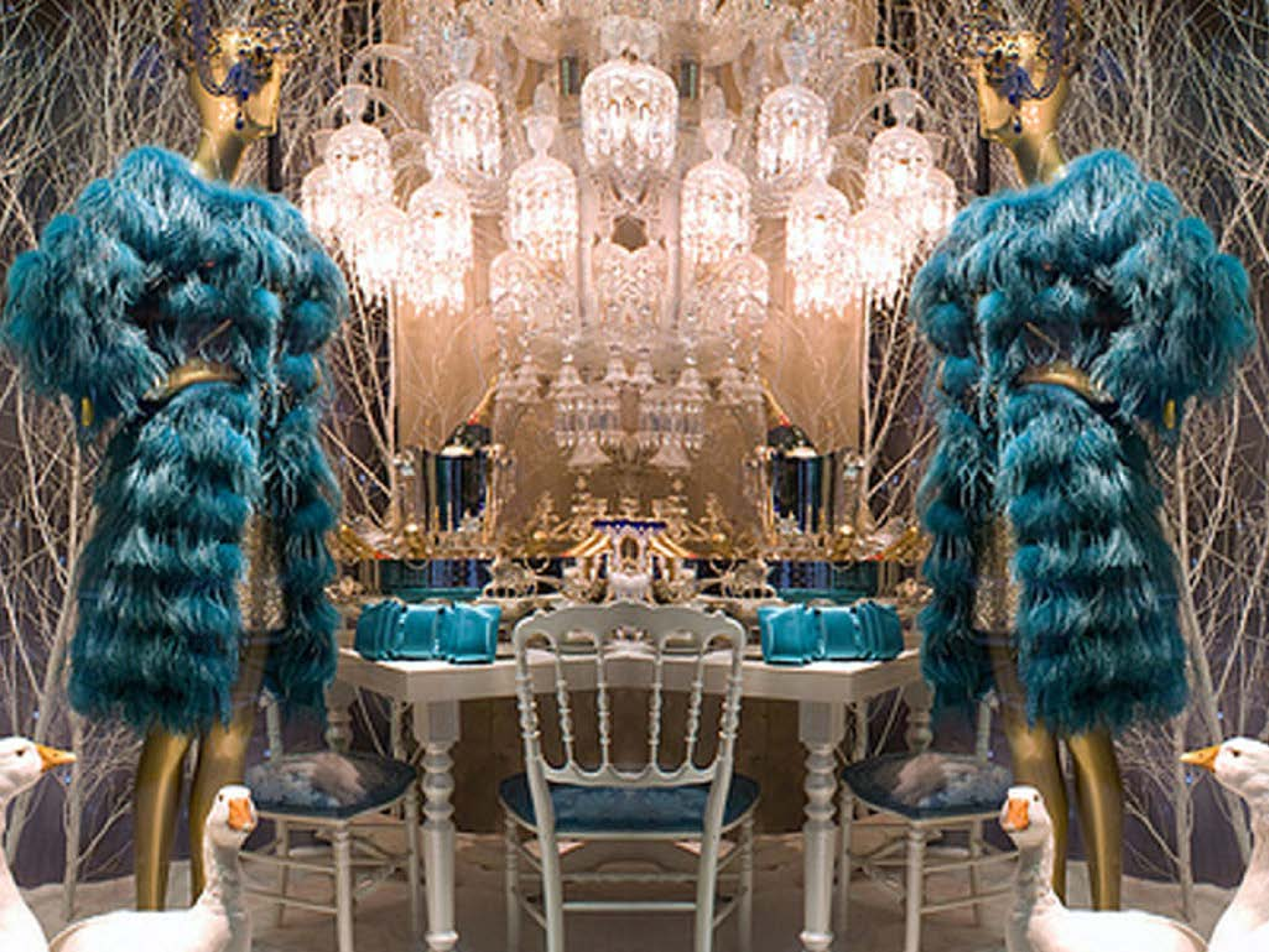

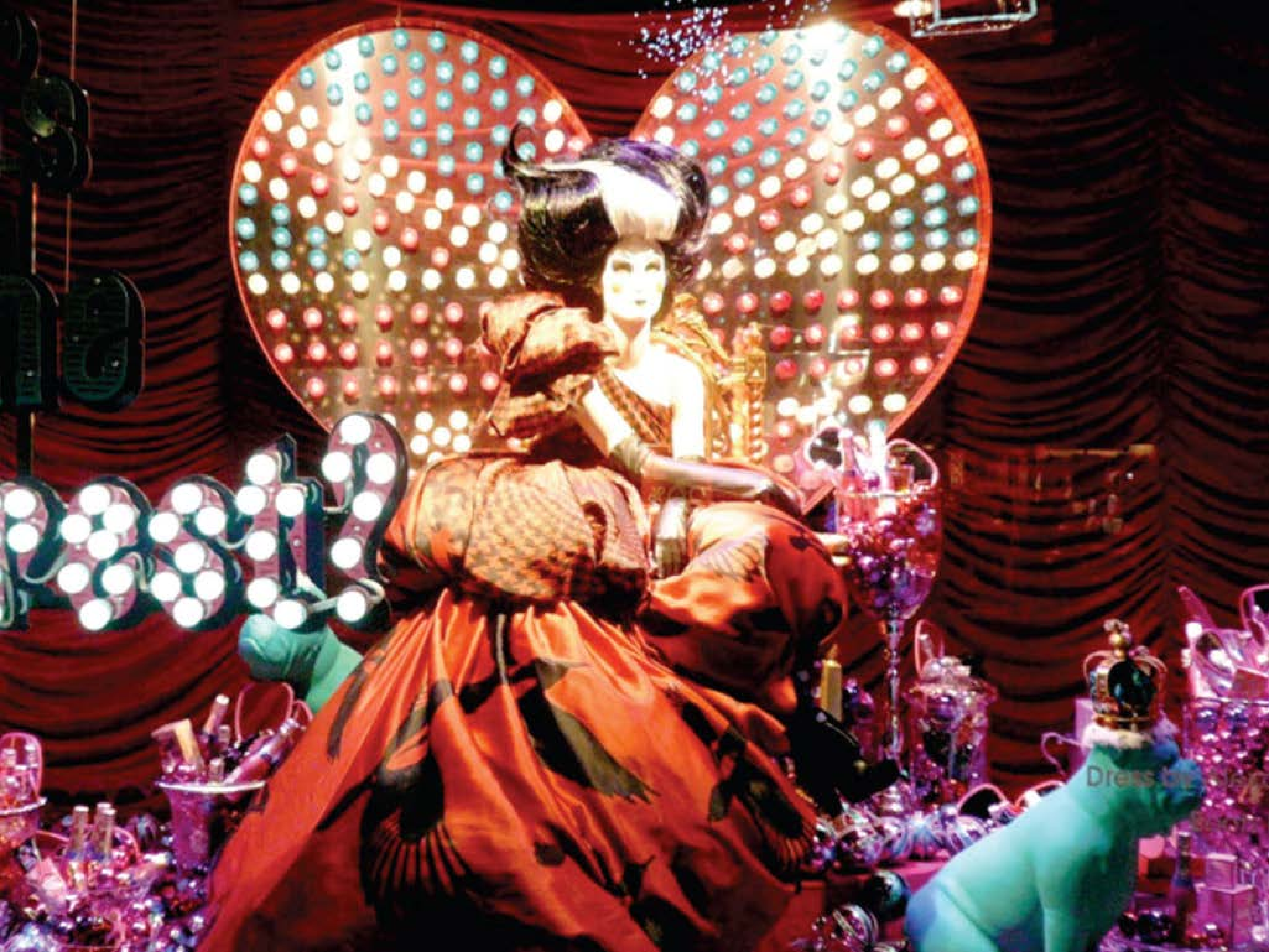
Dress by

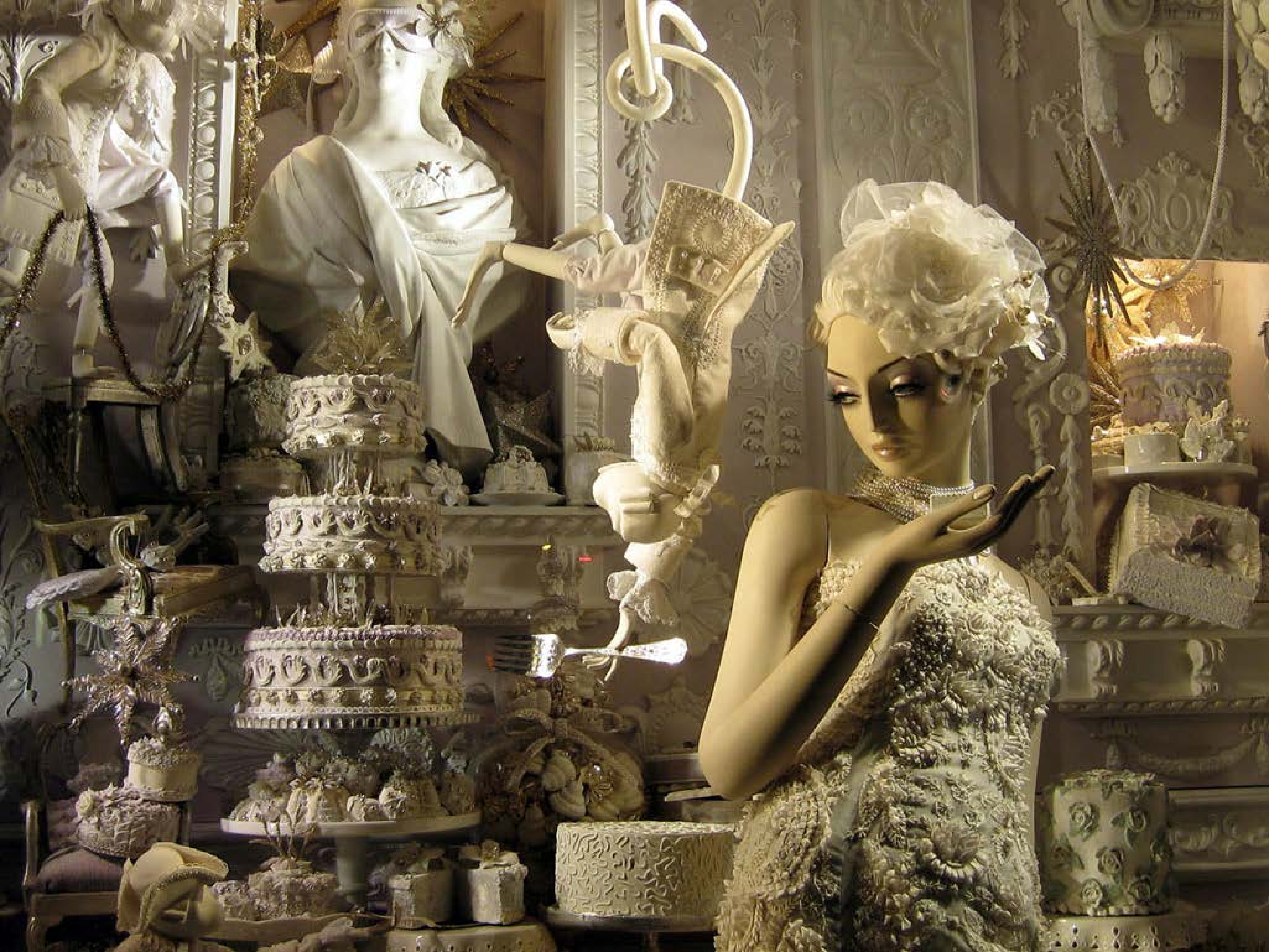

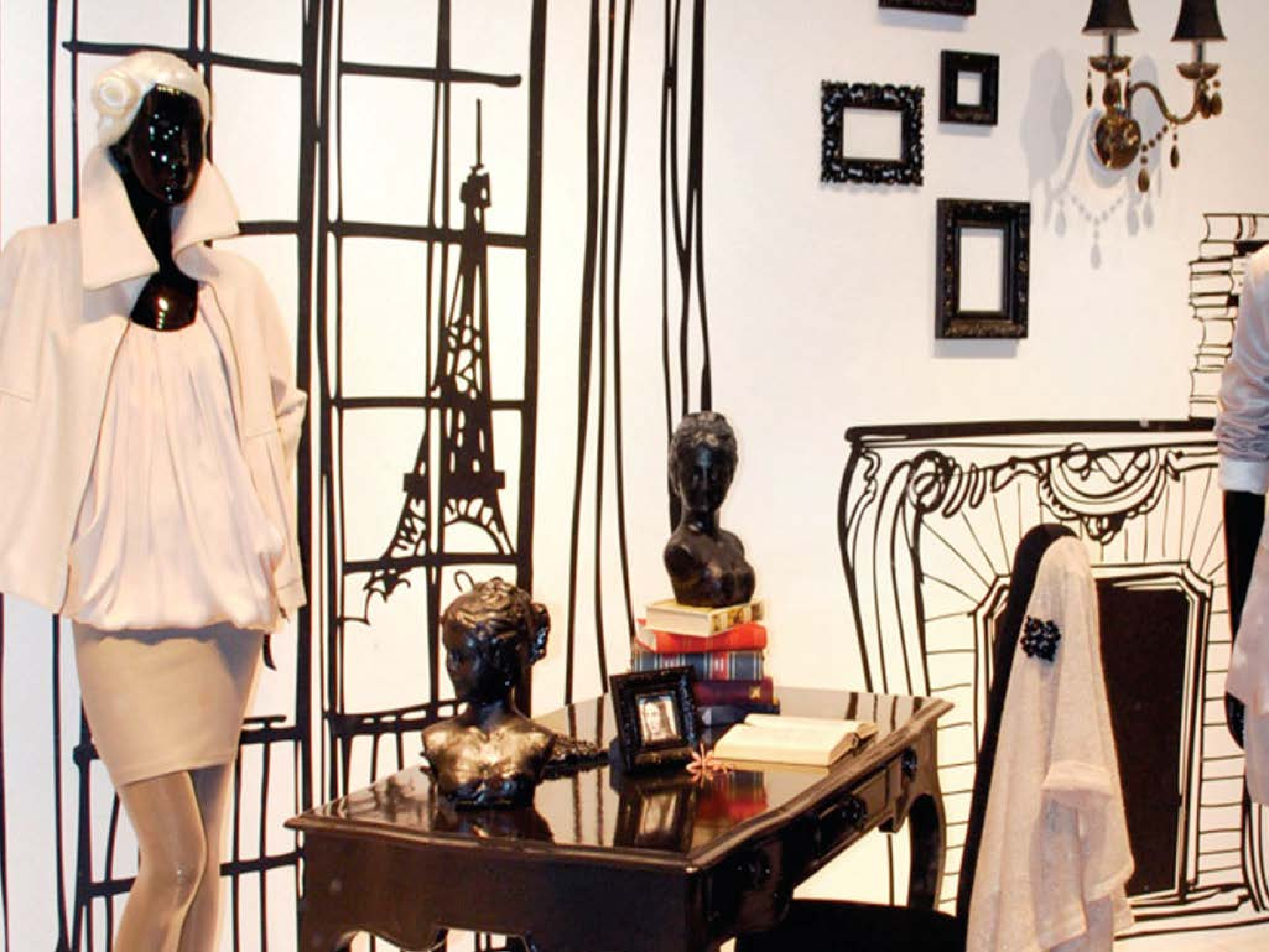

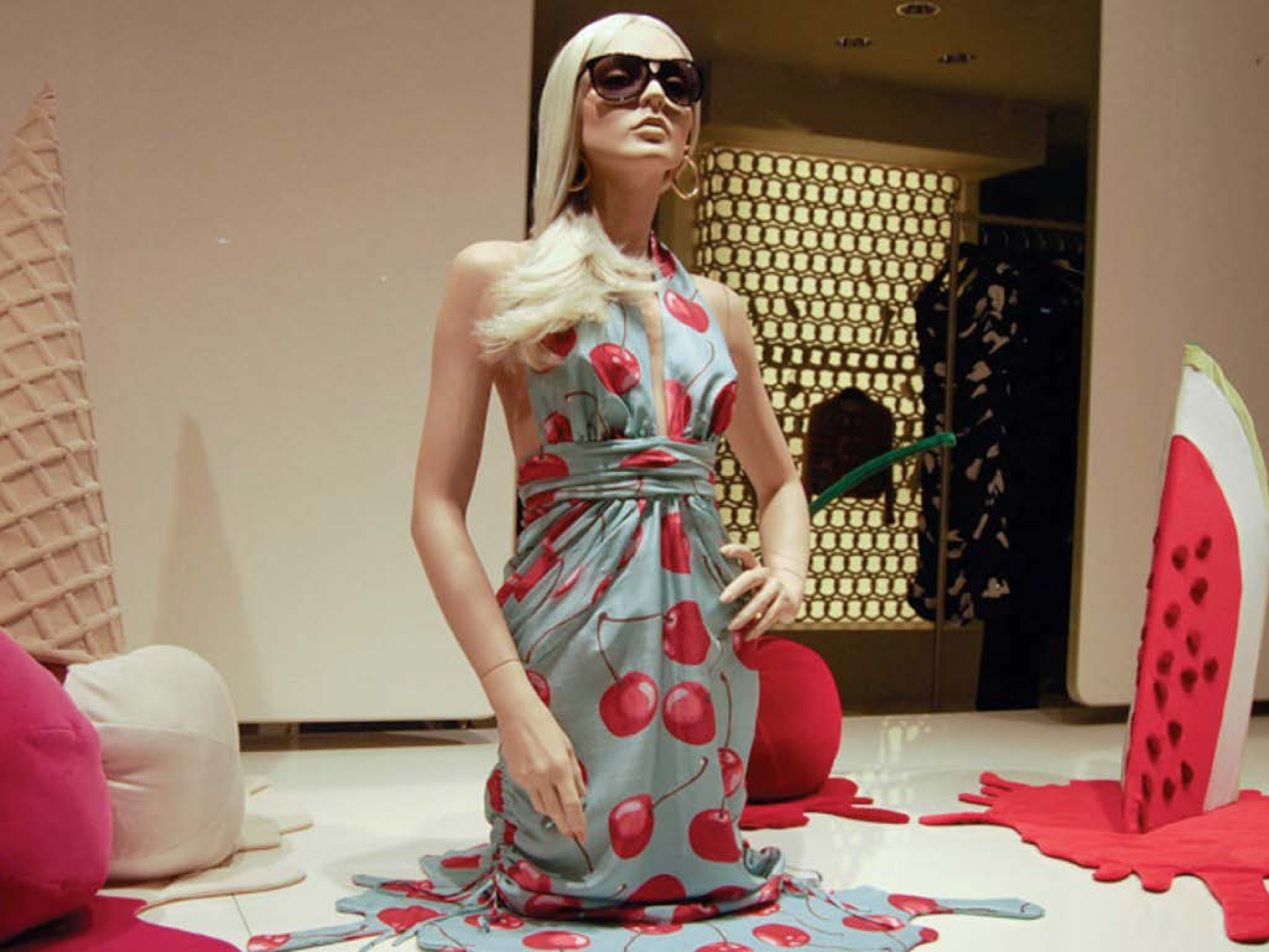

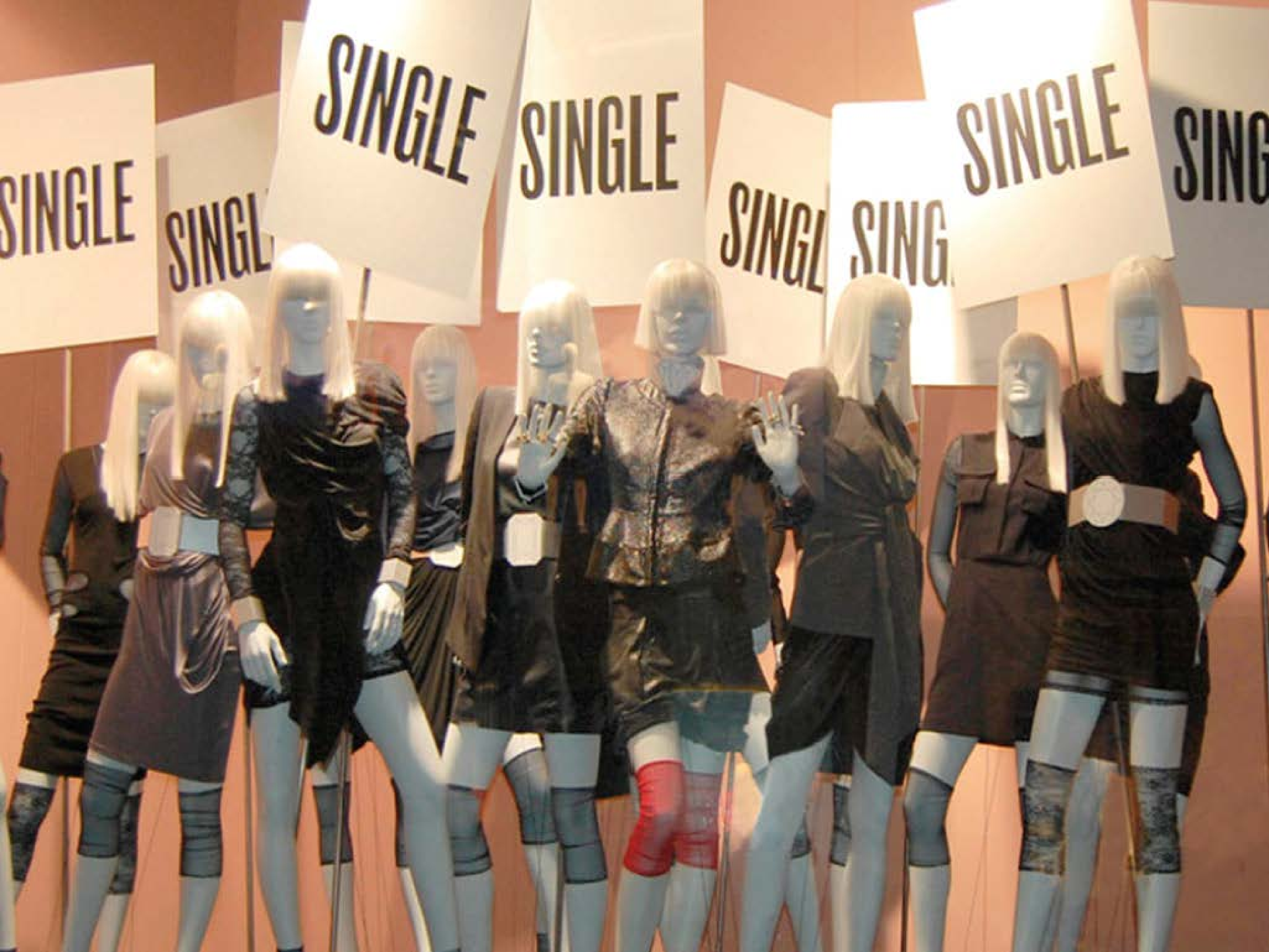
SINGLE
SINGL
SINGLE
SINGLE
SINGL
SING
SINGLE
SING

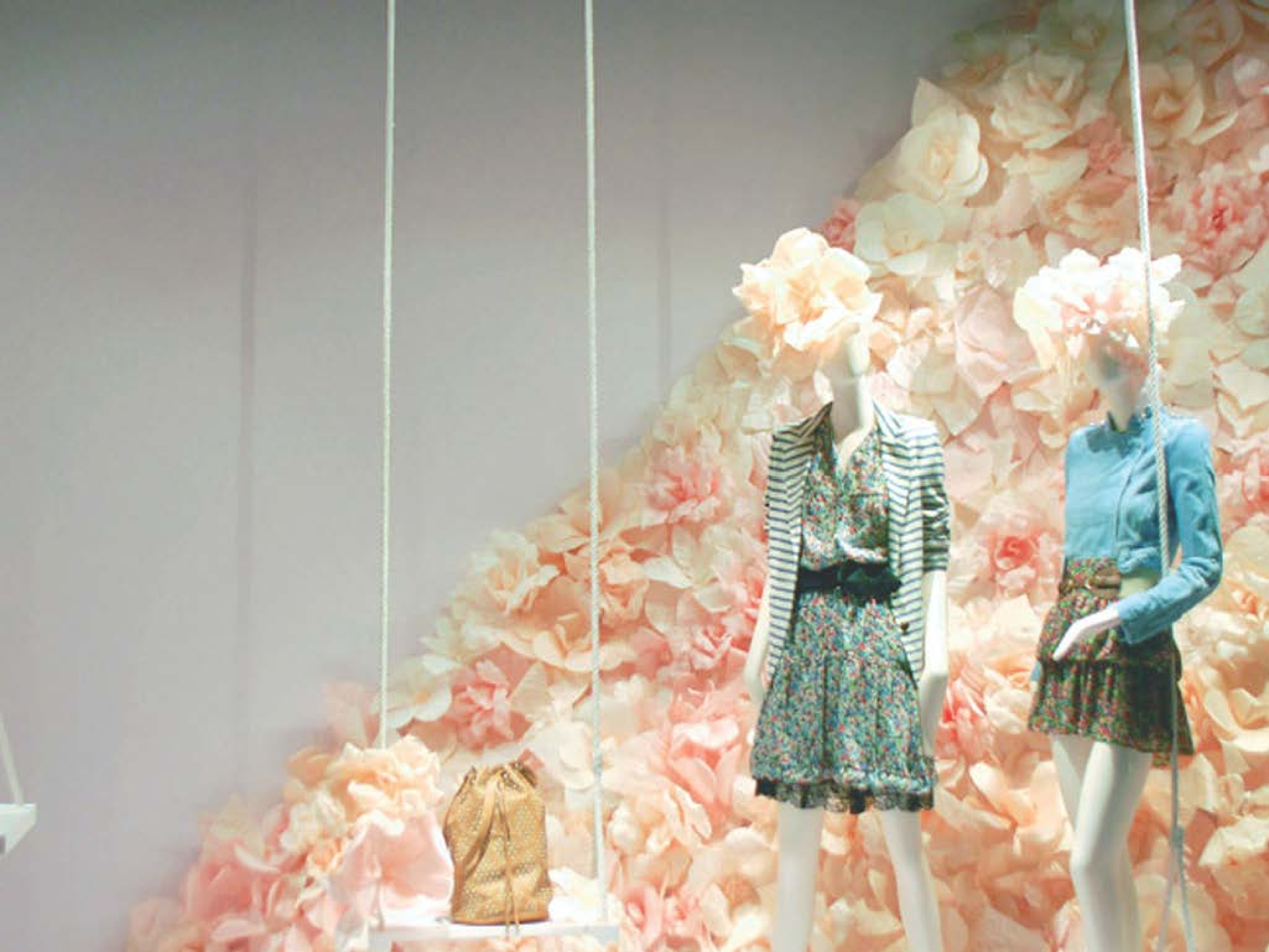

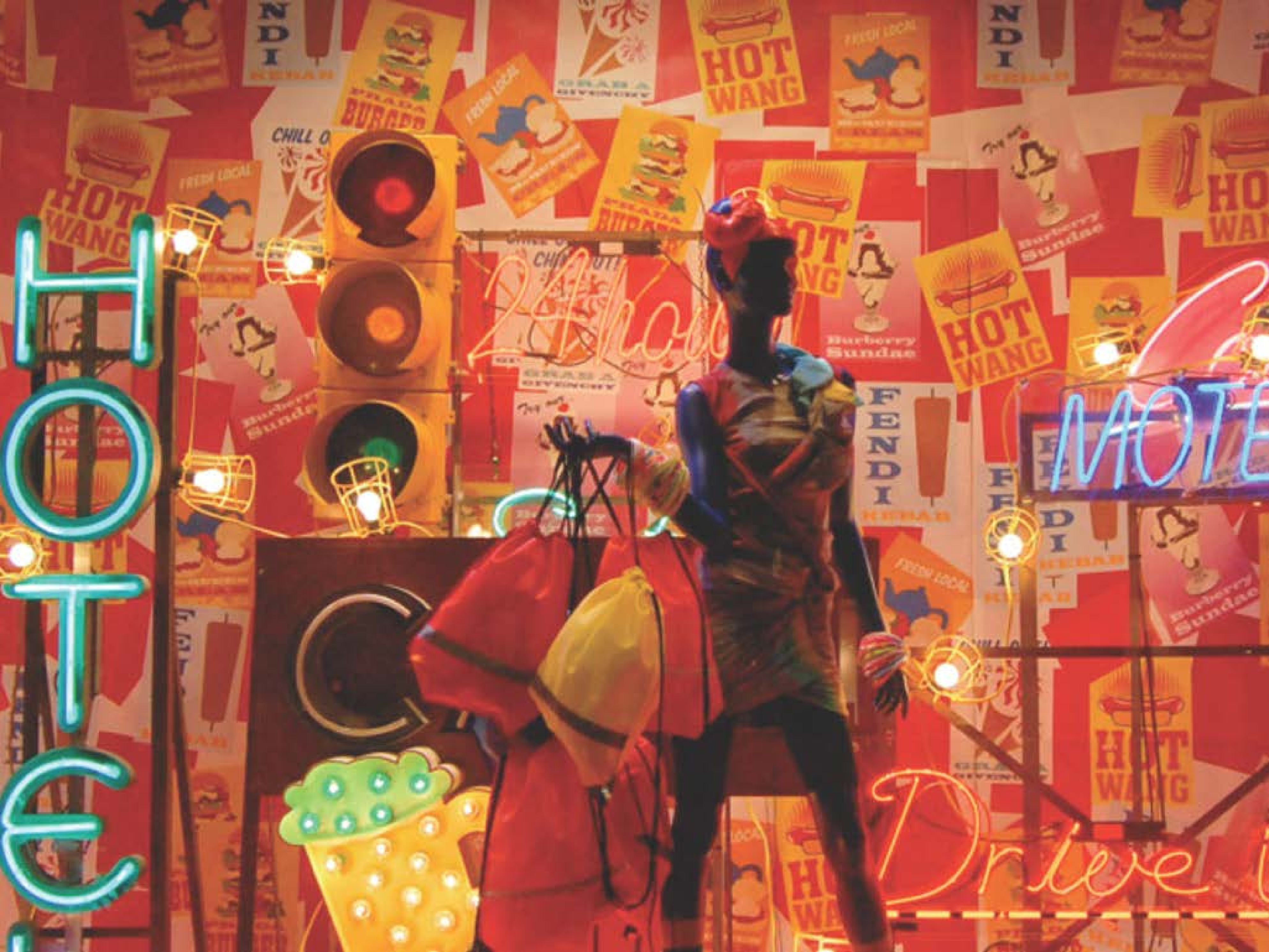
HOT WANG
PRADA BURGER
FENDI KEBAB
Burberry Sundae
FRESH LOCAL
GRAB A GIVENCHY
CHILL OUT!
MOTE
HOTE

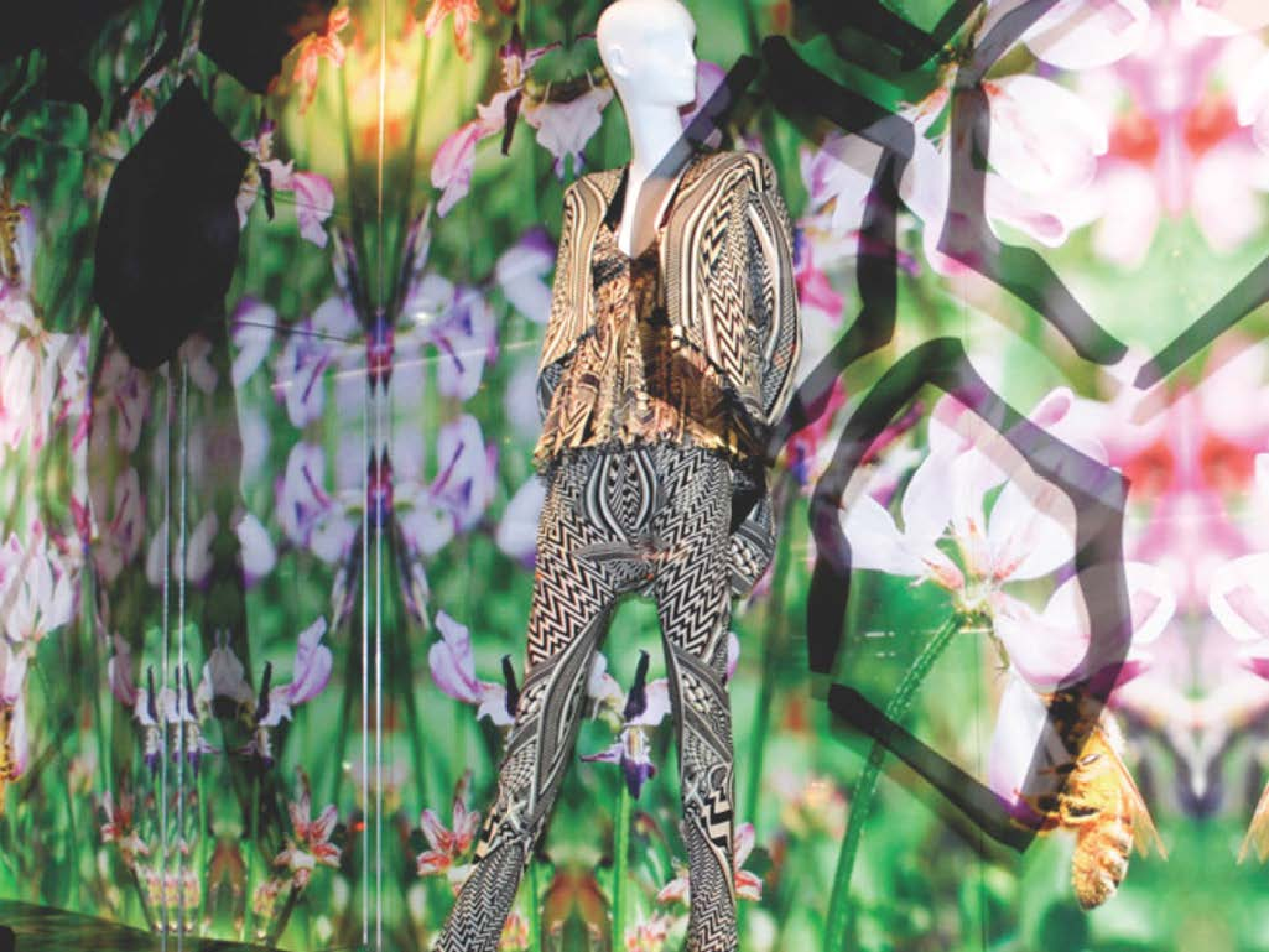

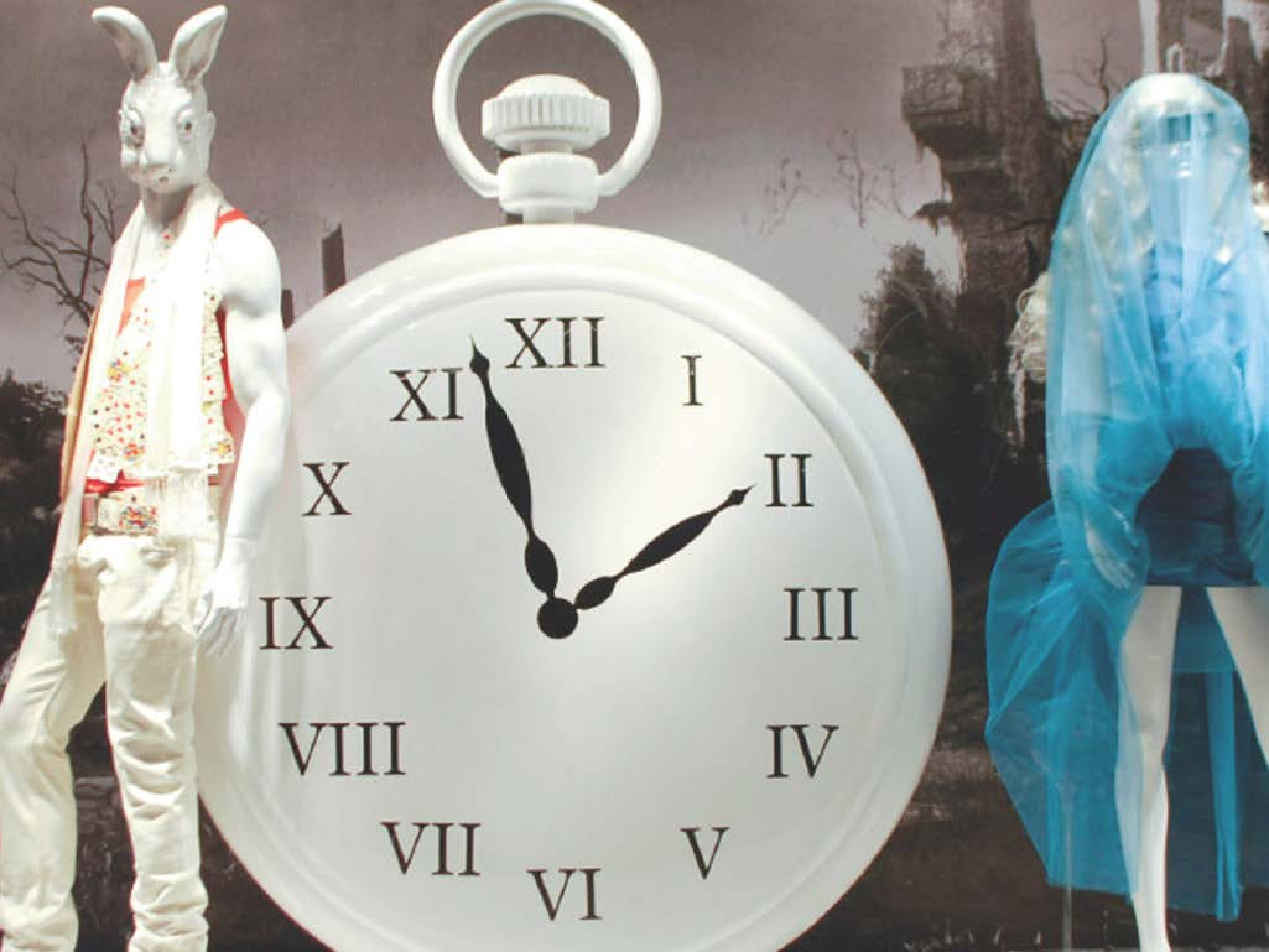
XII
I
II
III
IV
V
VI
VII
VIII
IX
X
XI

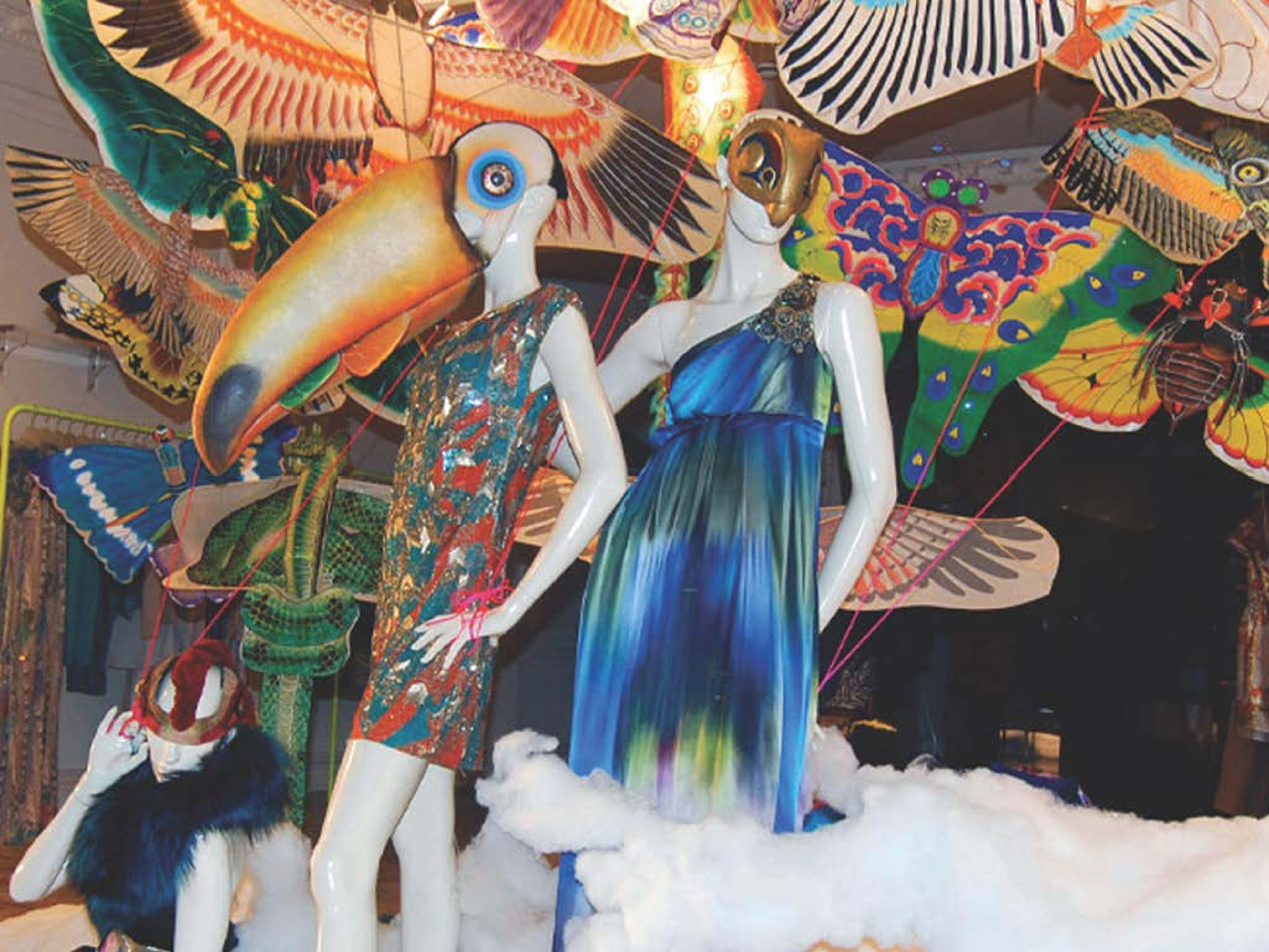

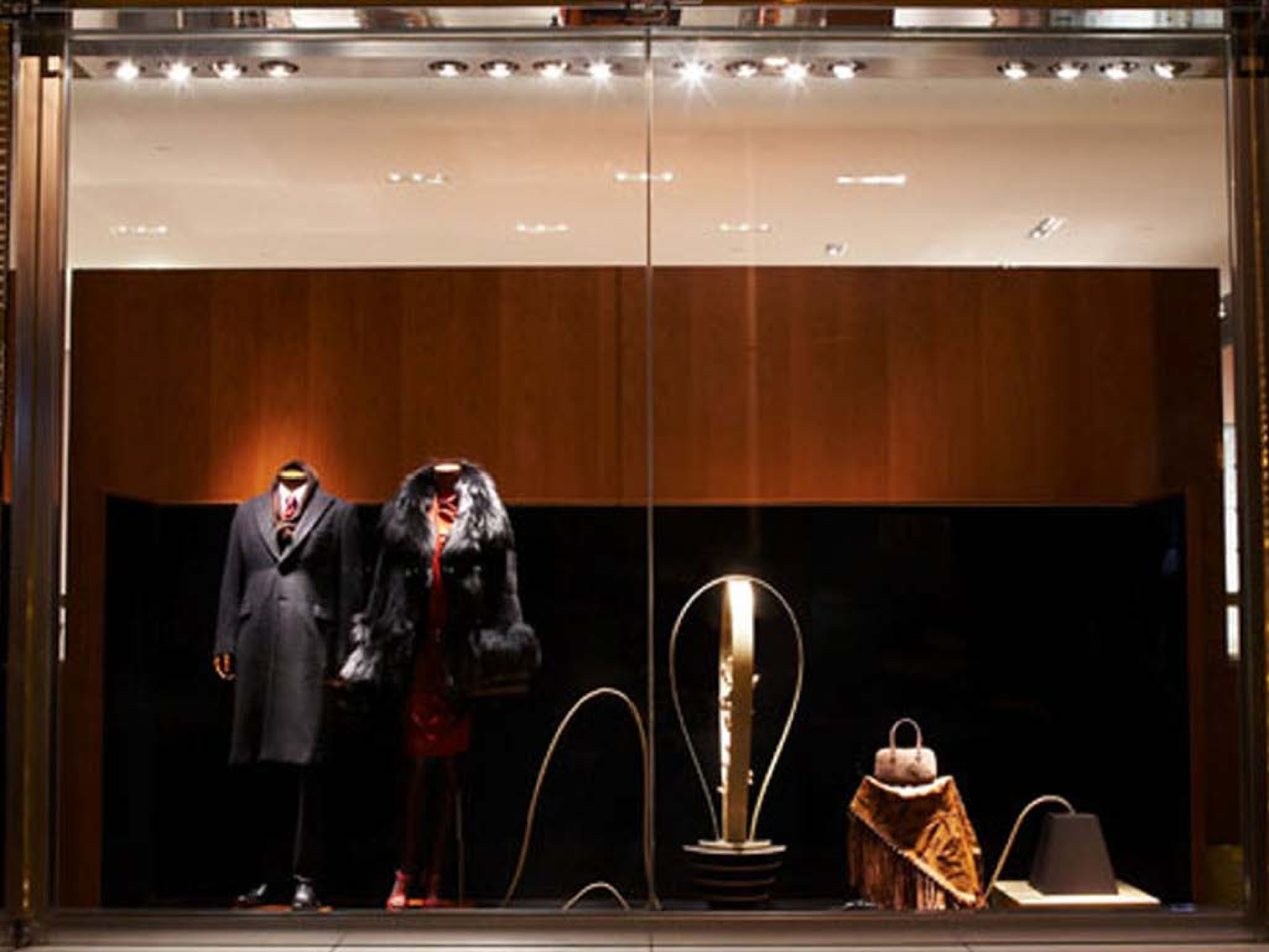

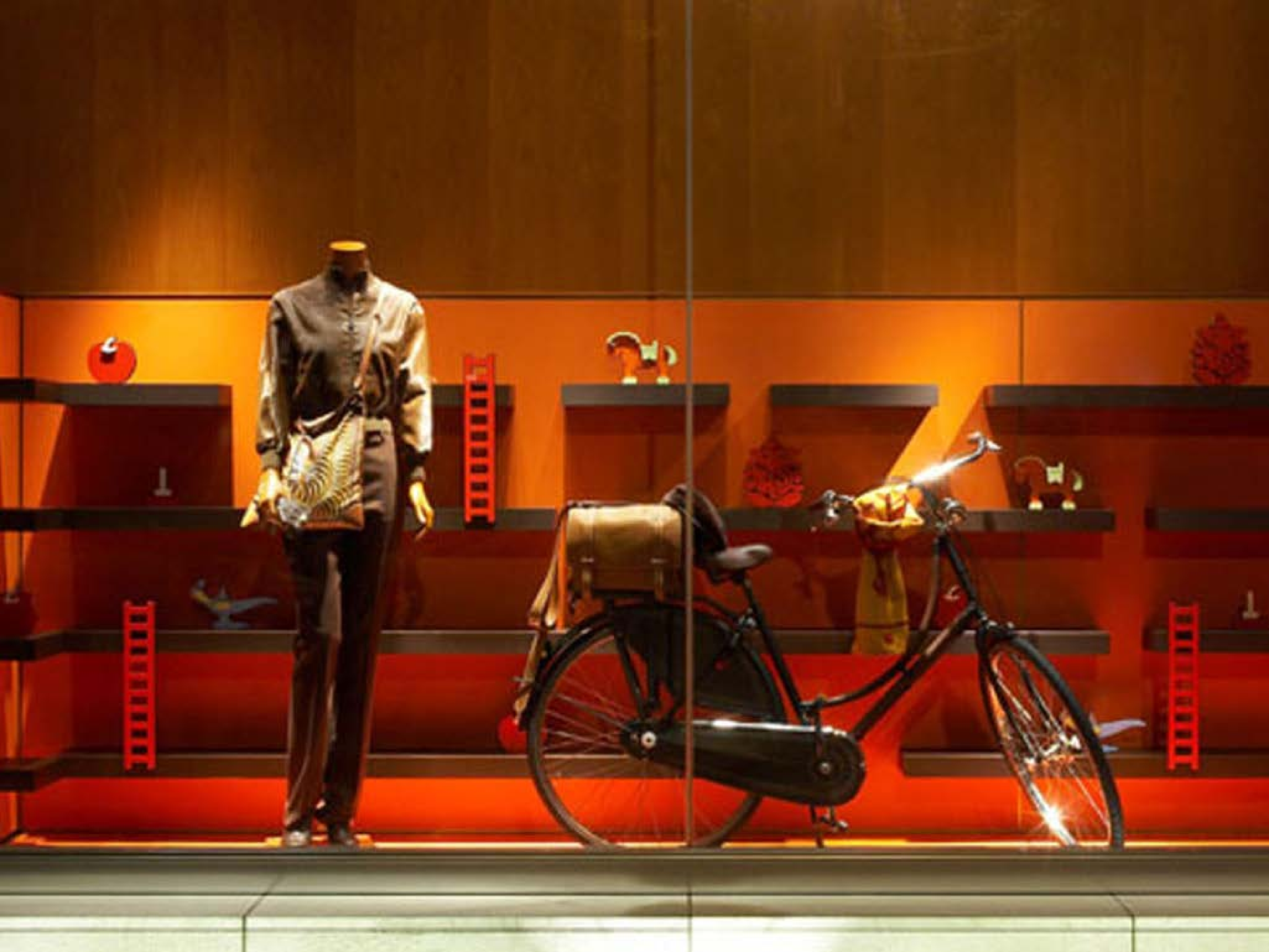

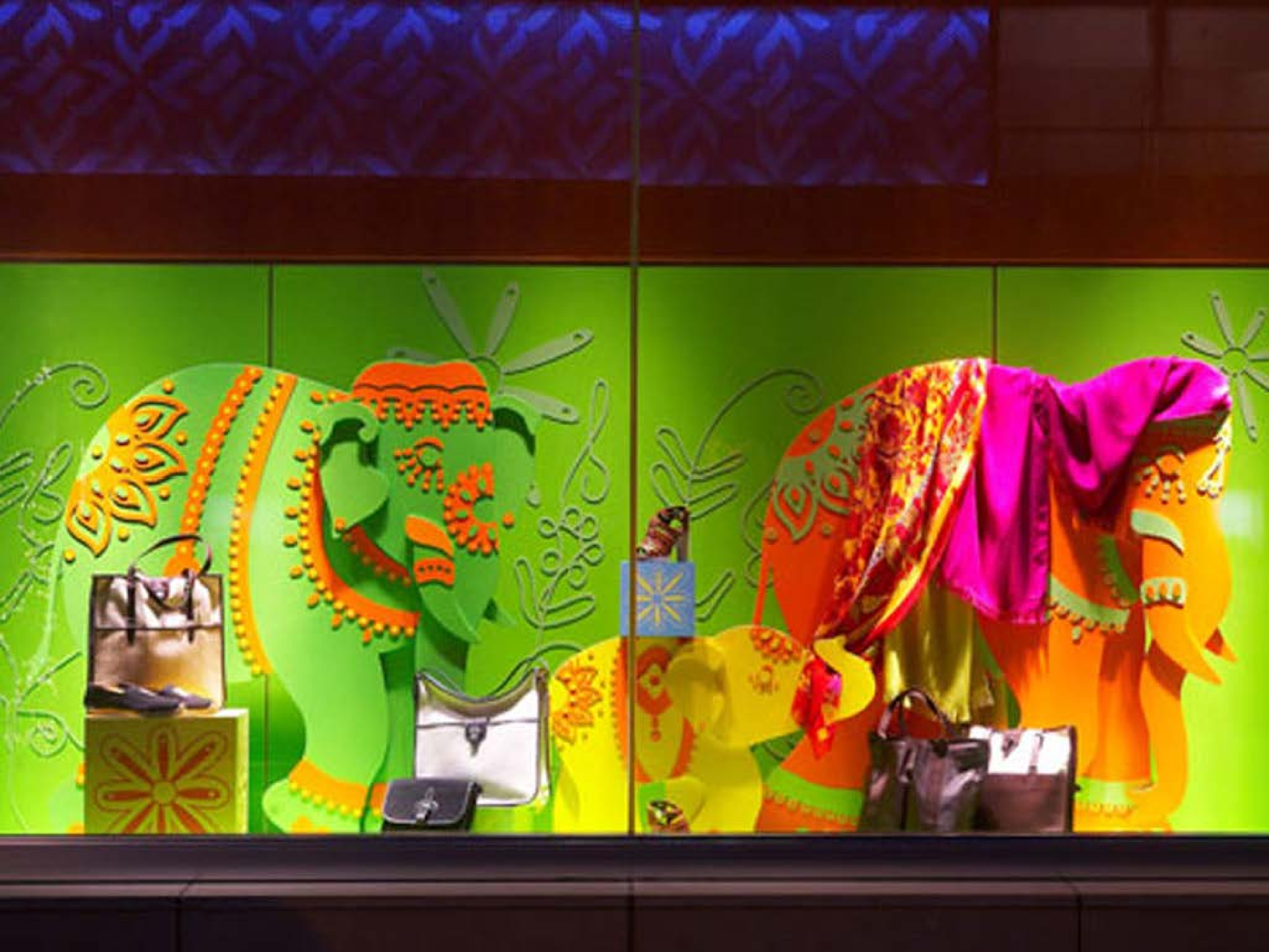

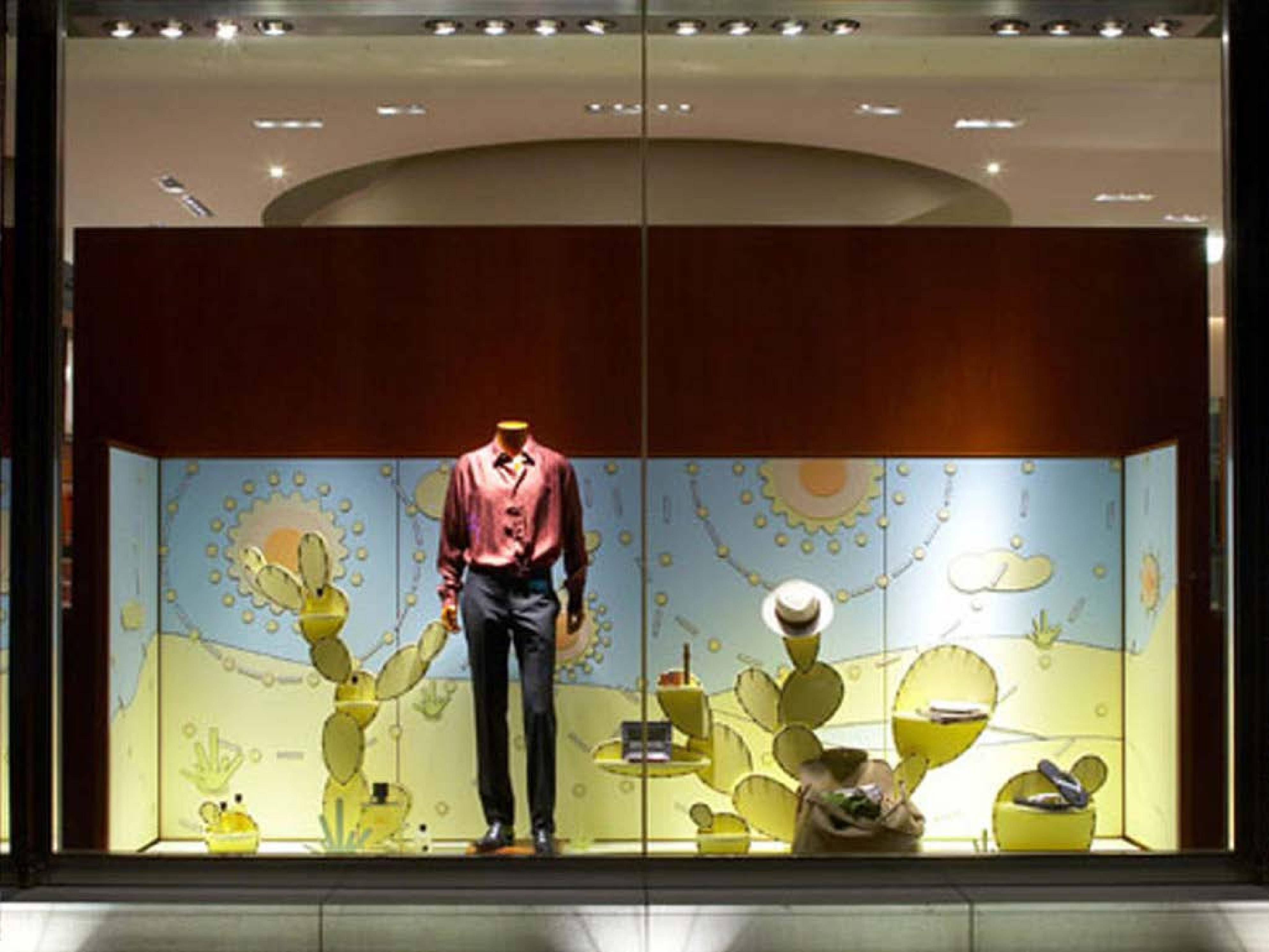

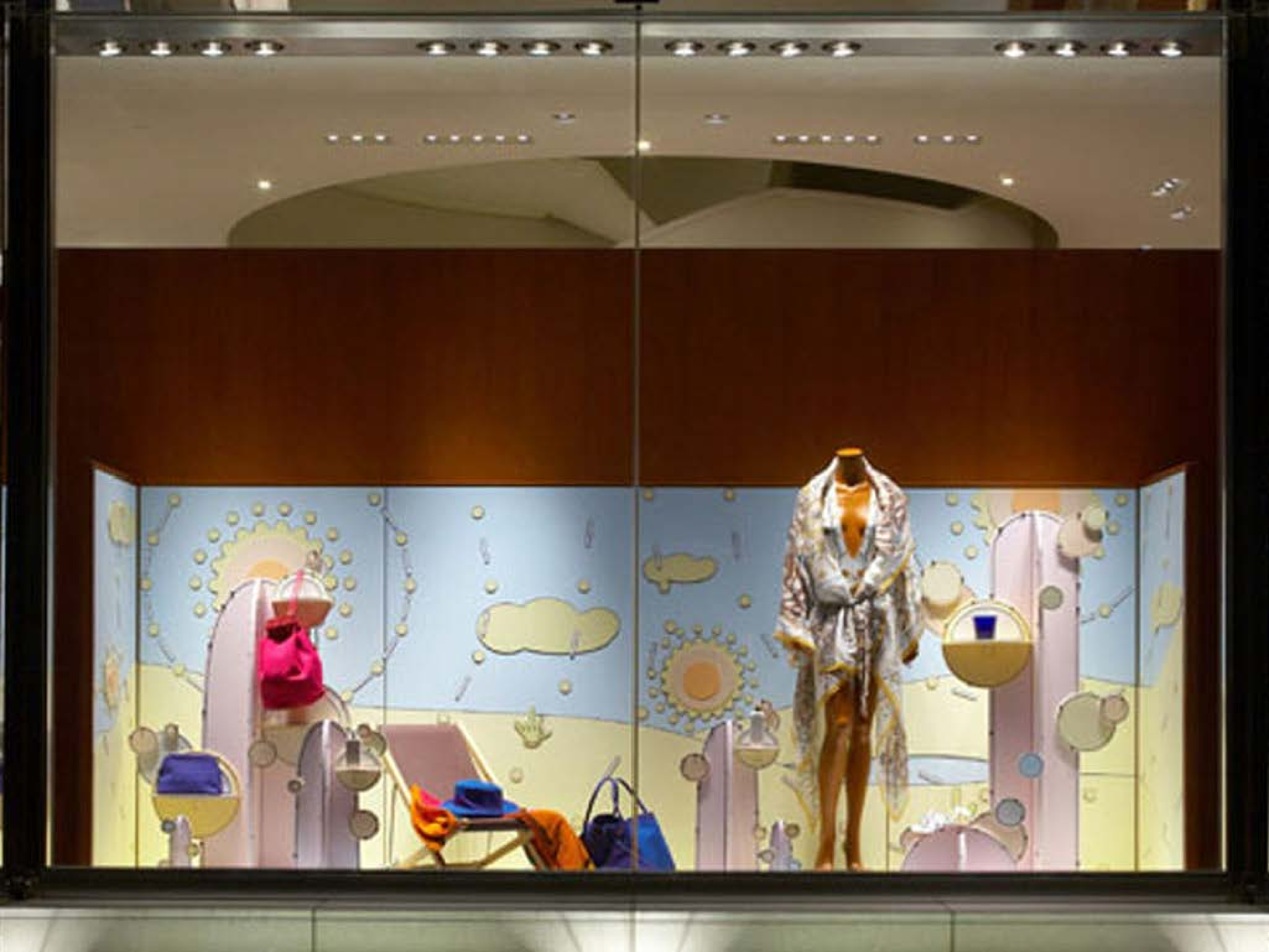

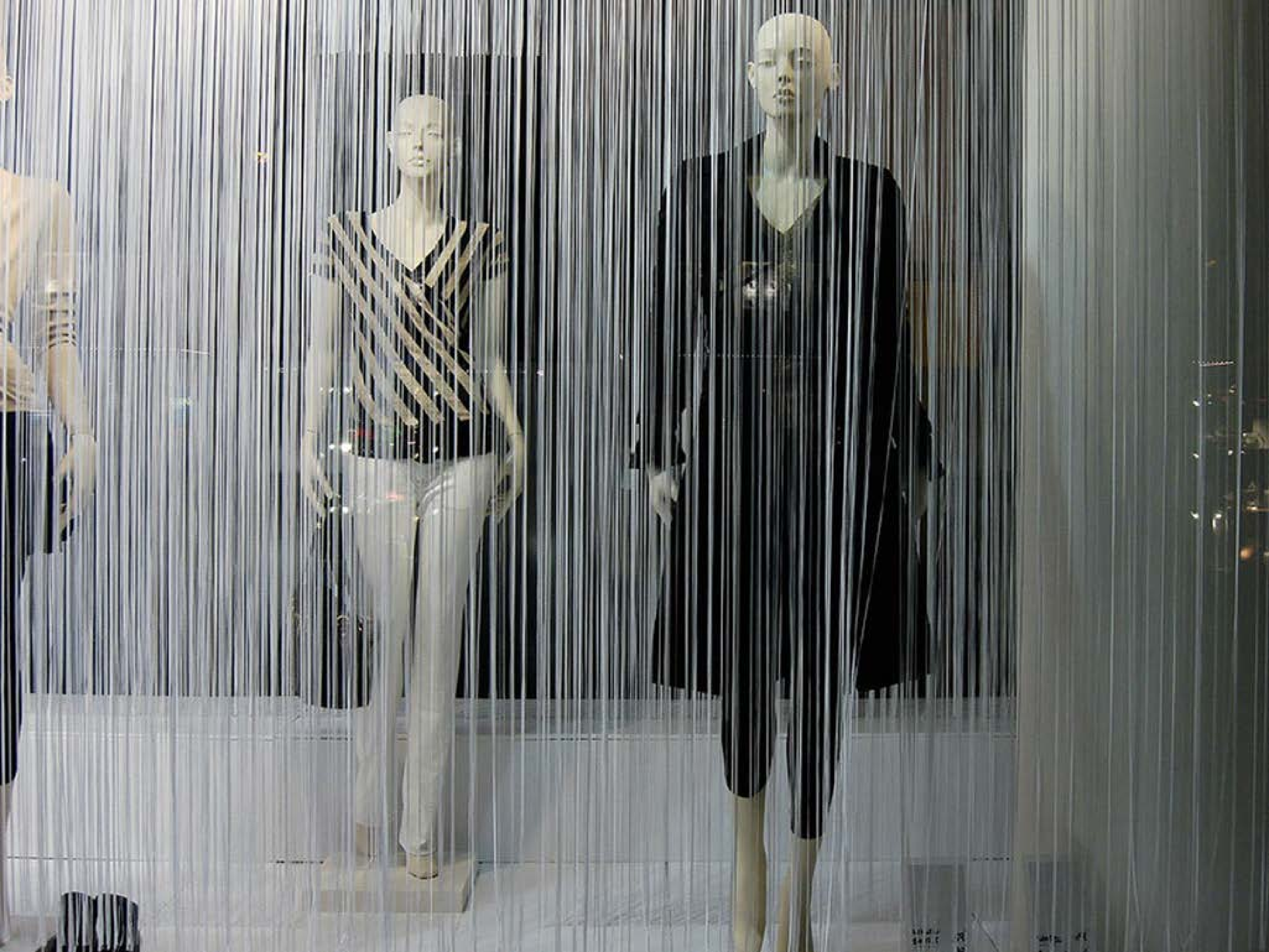

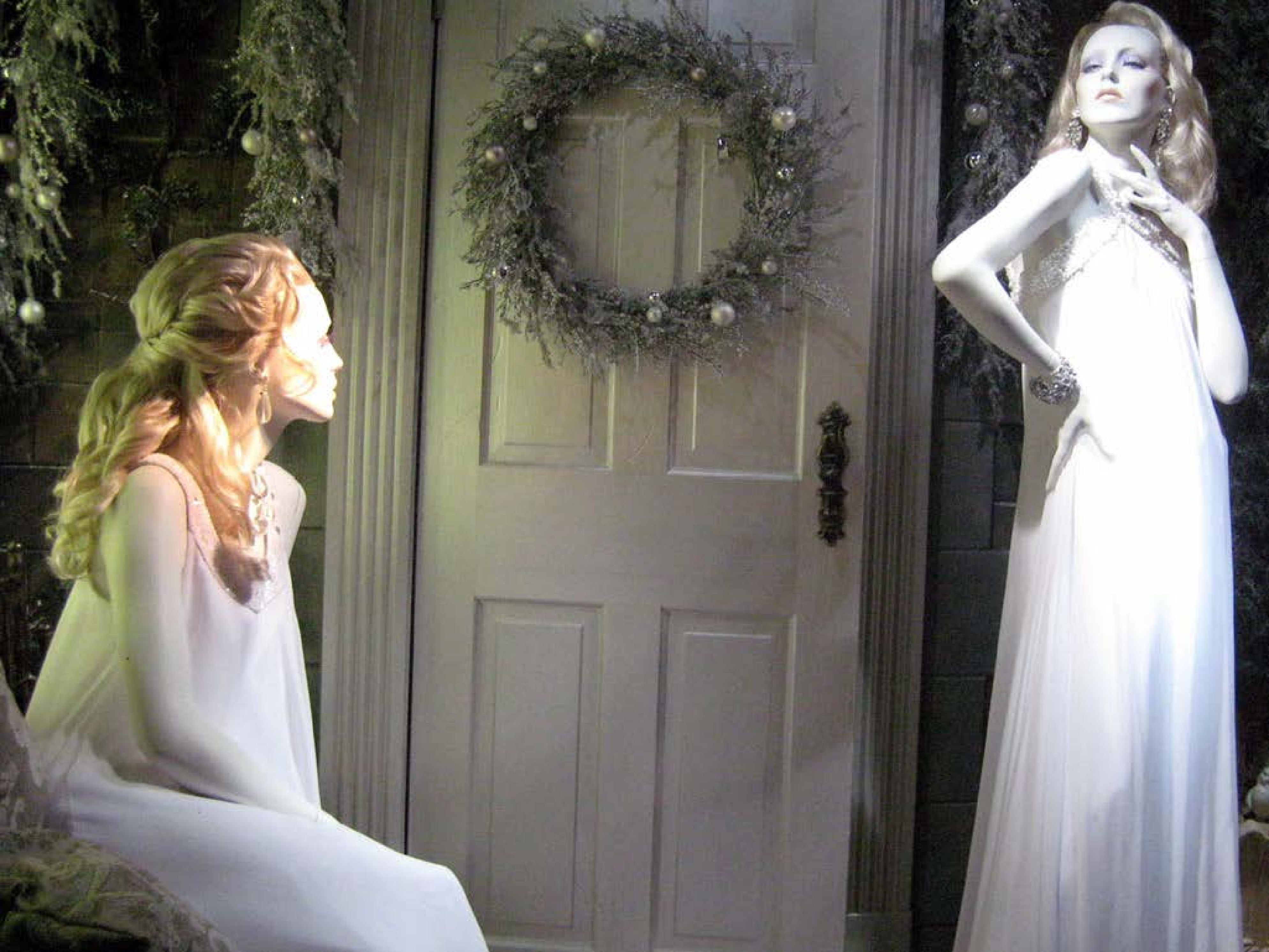

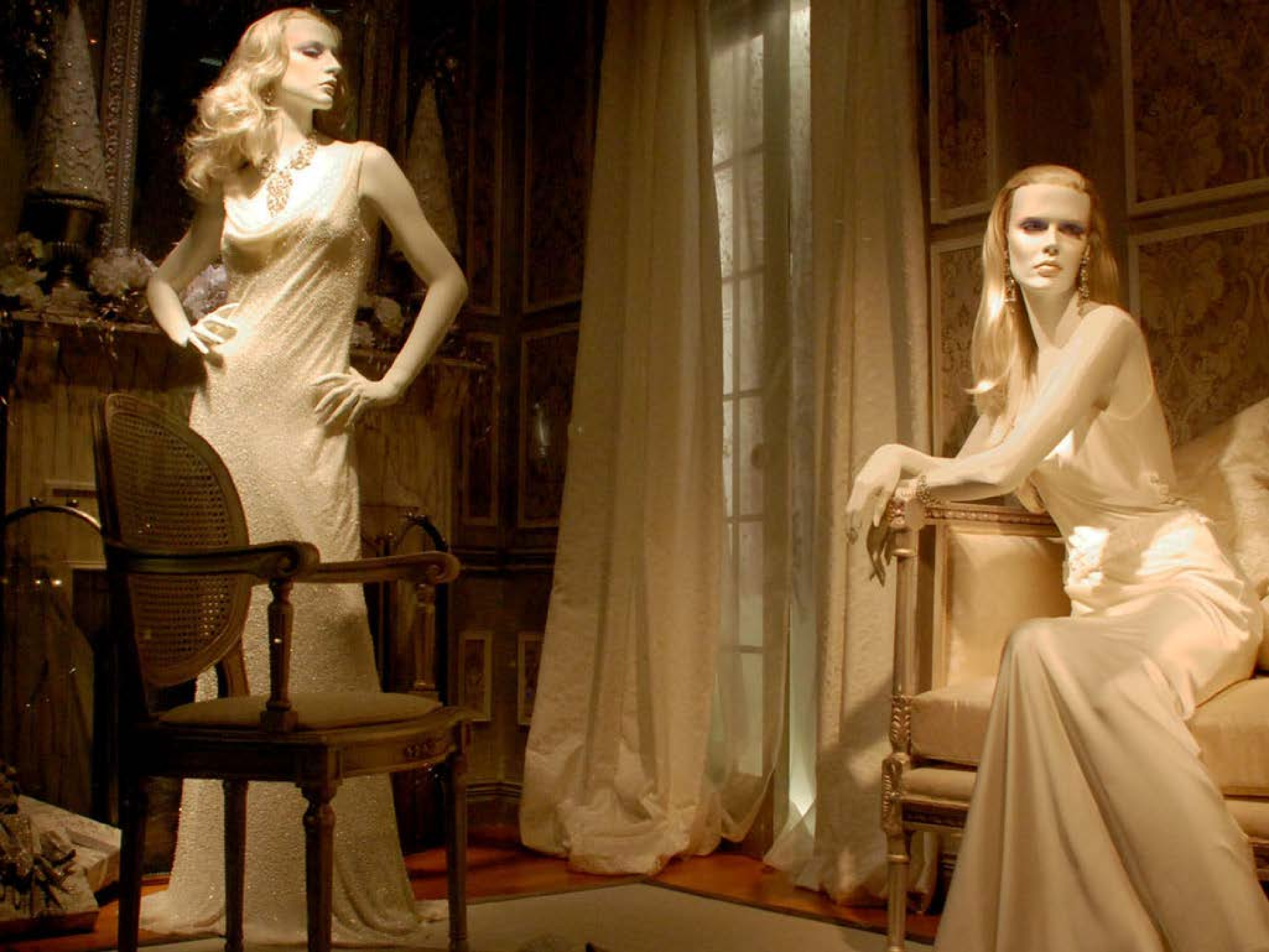

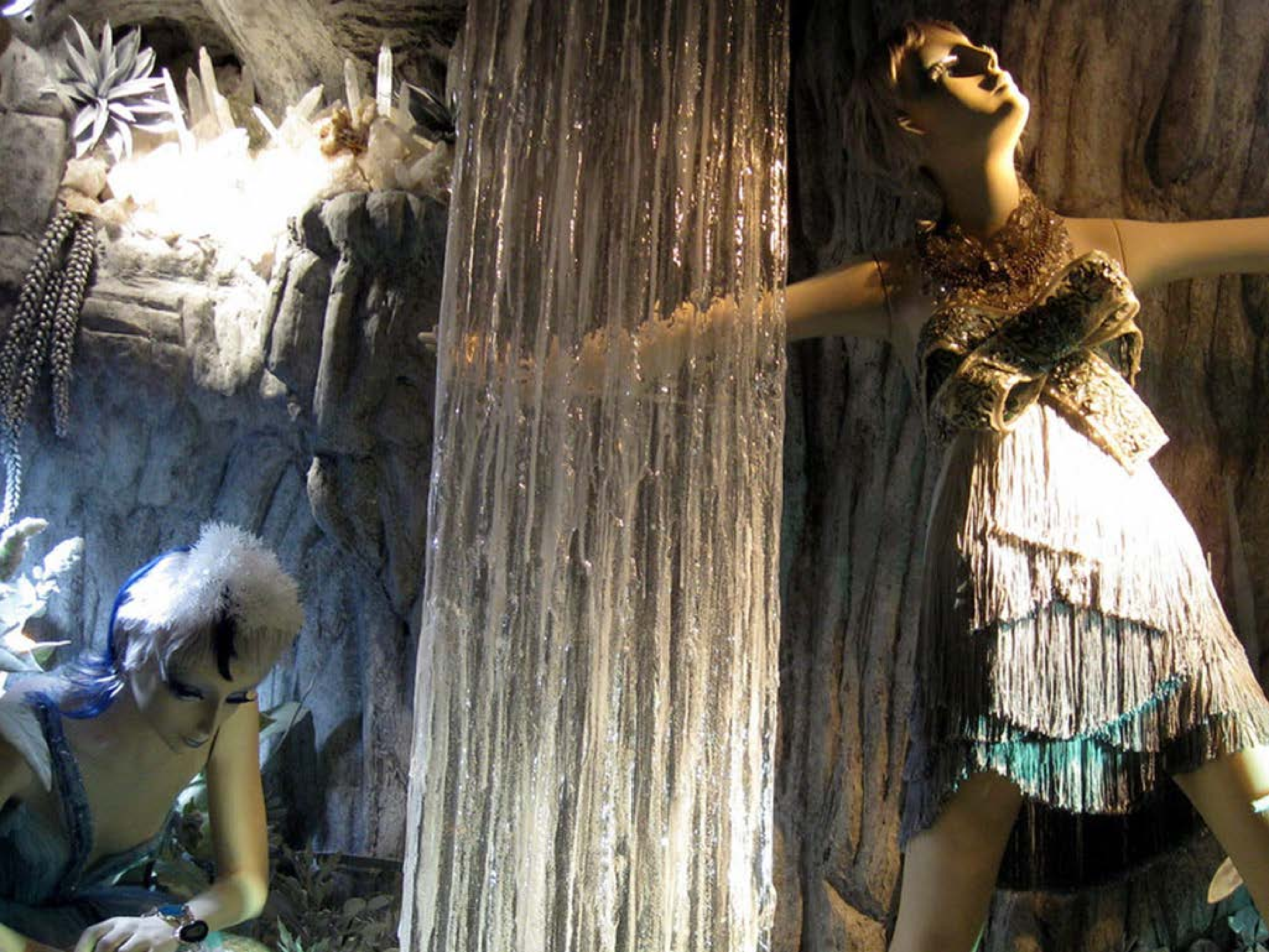

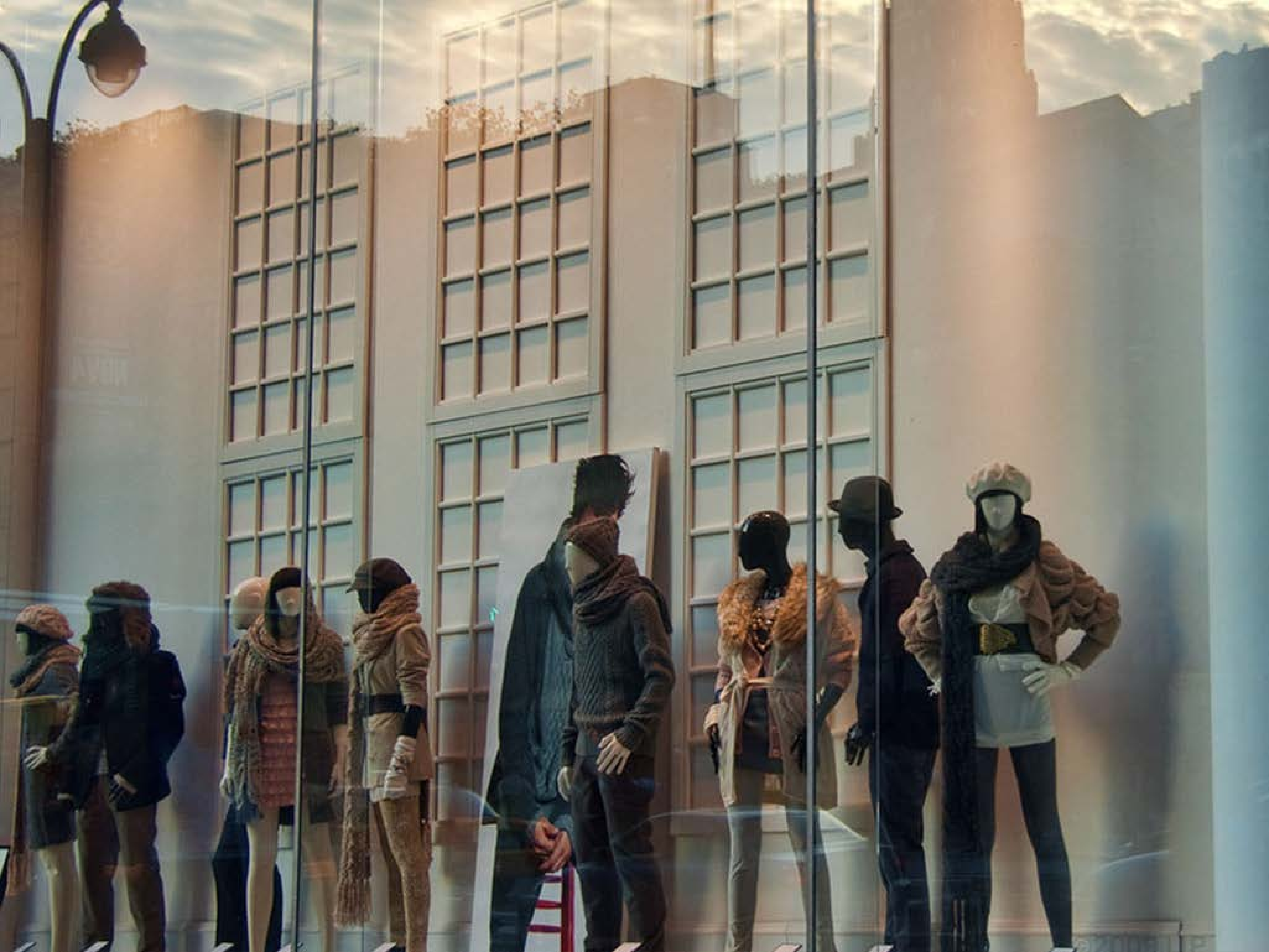

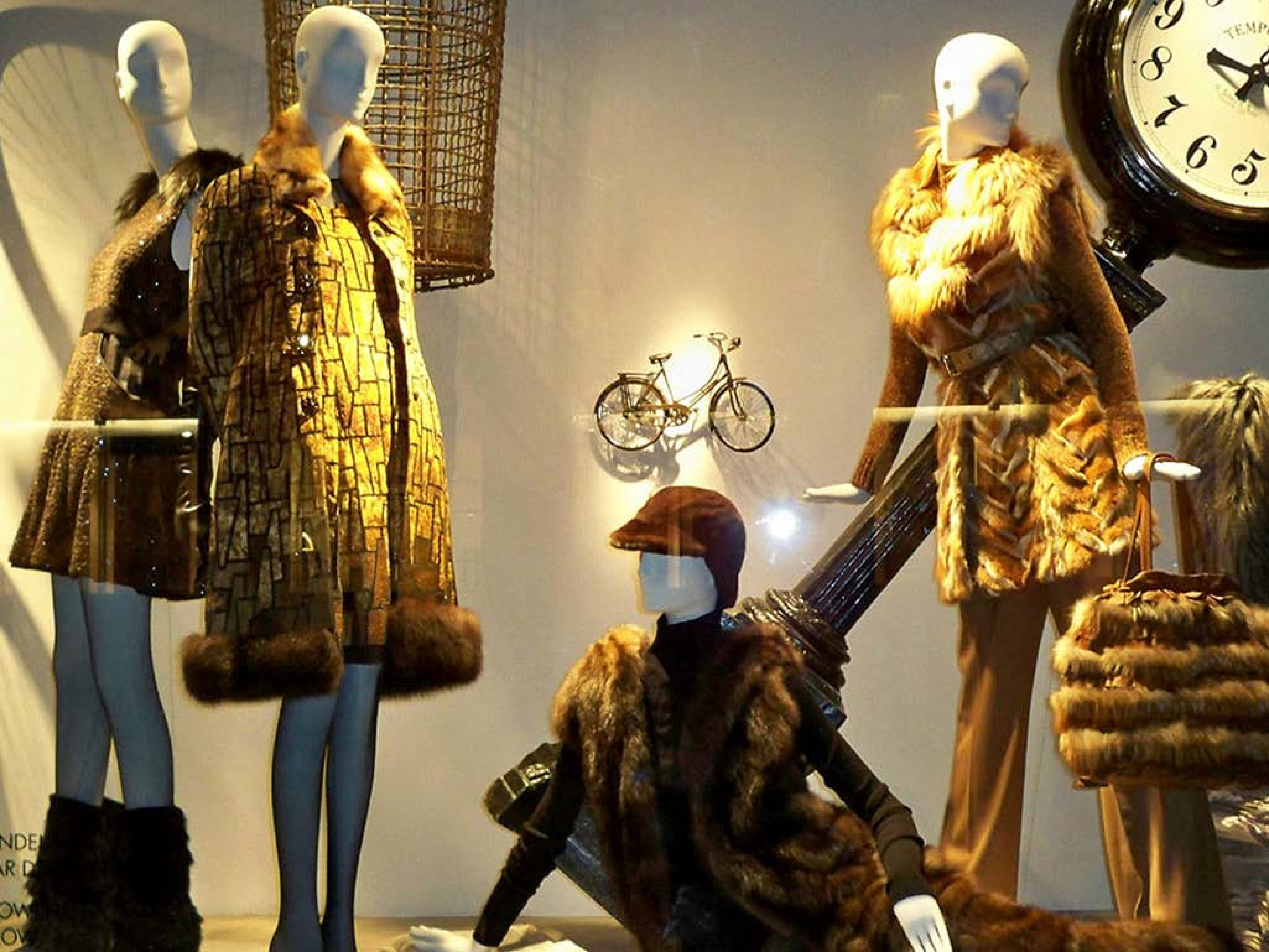

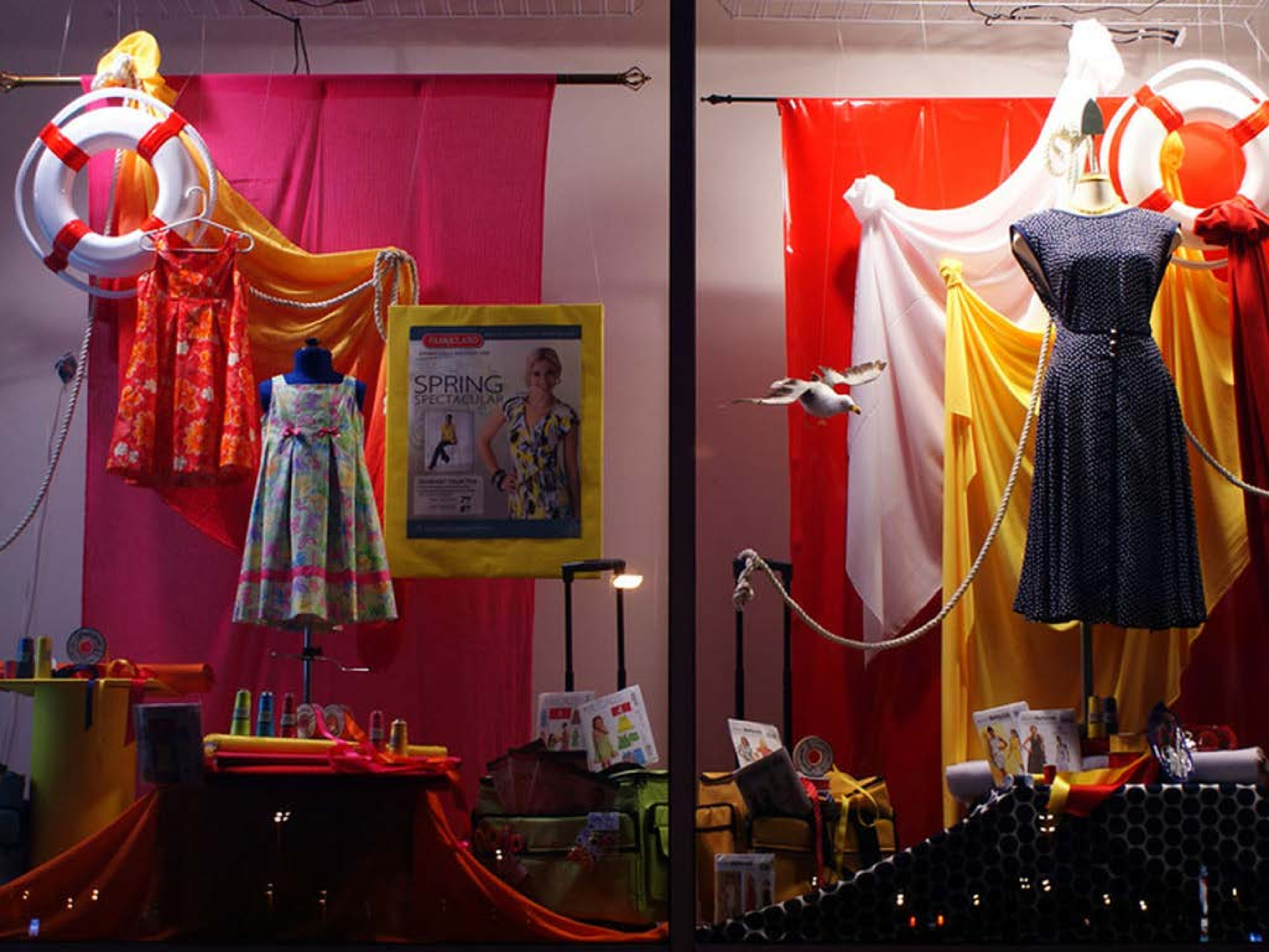
SPRING
SPECTACULAR

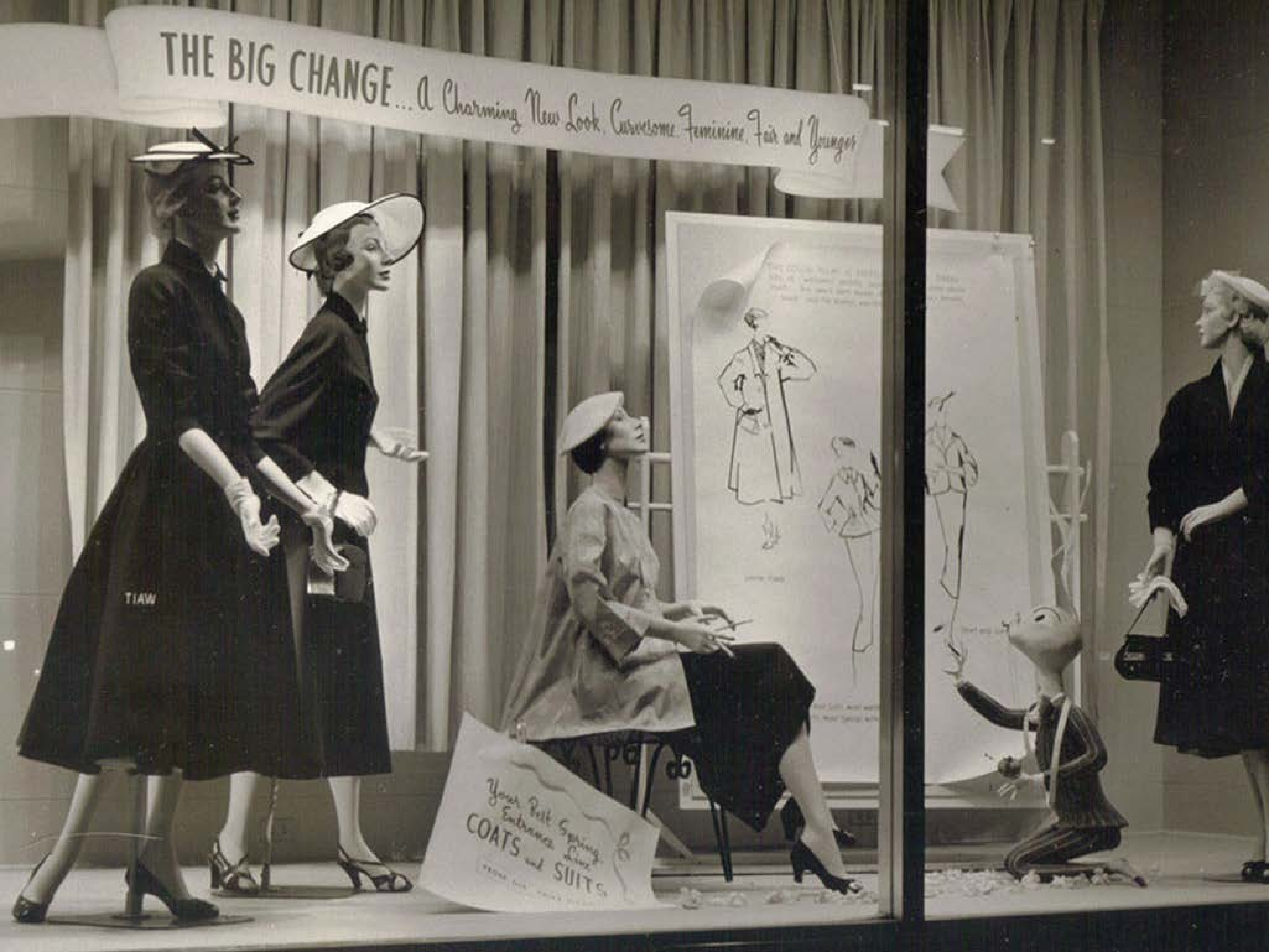
THE BIG CHANGE... a Charming New Look, Curvesome, Feminine, Fair and Younger
TIAW
Your Best Spring Entrance Line
COATS and SUITS

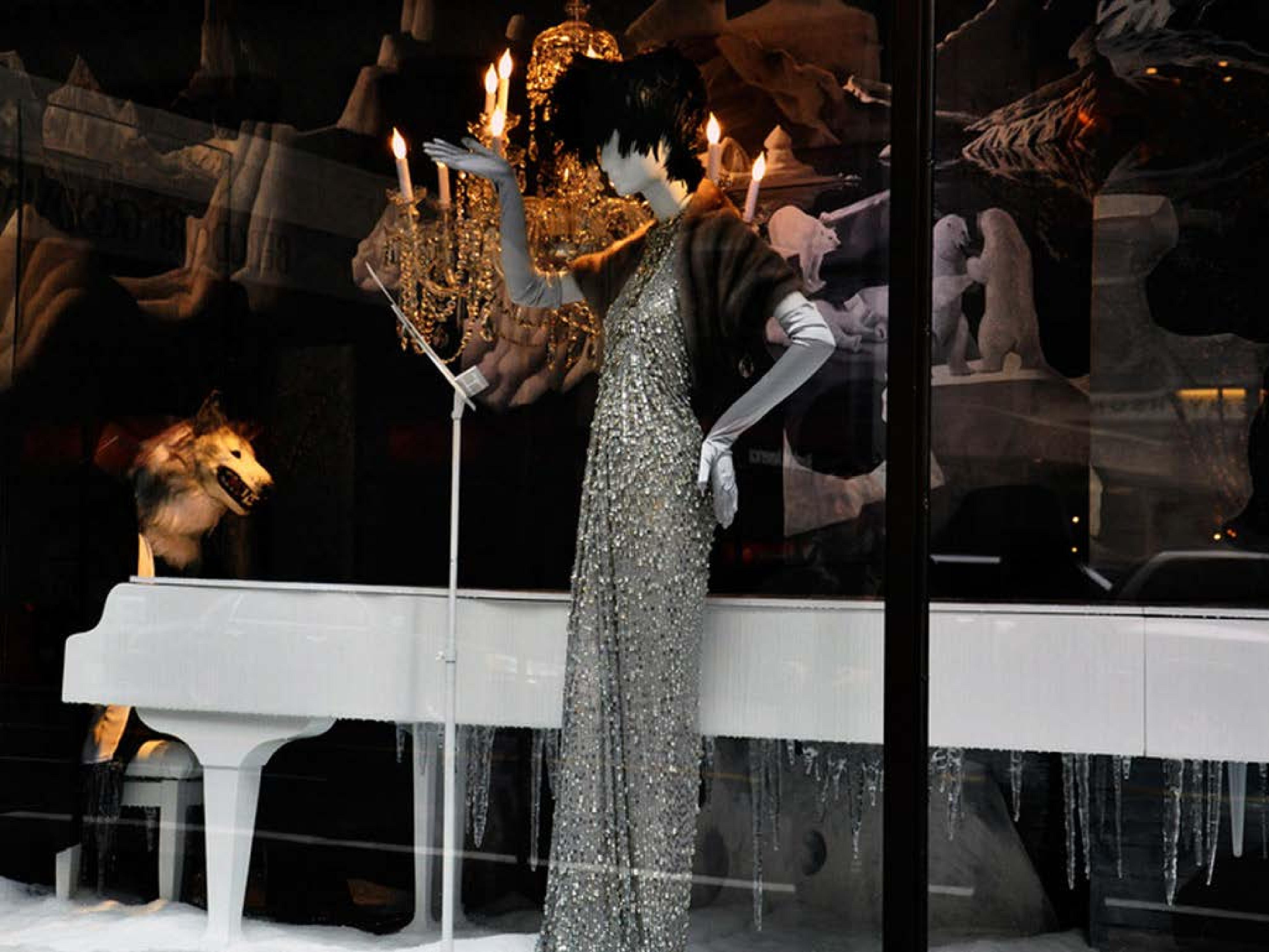

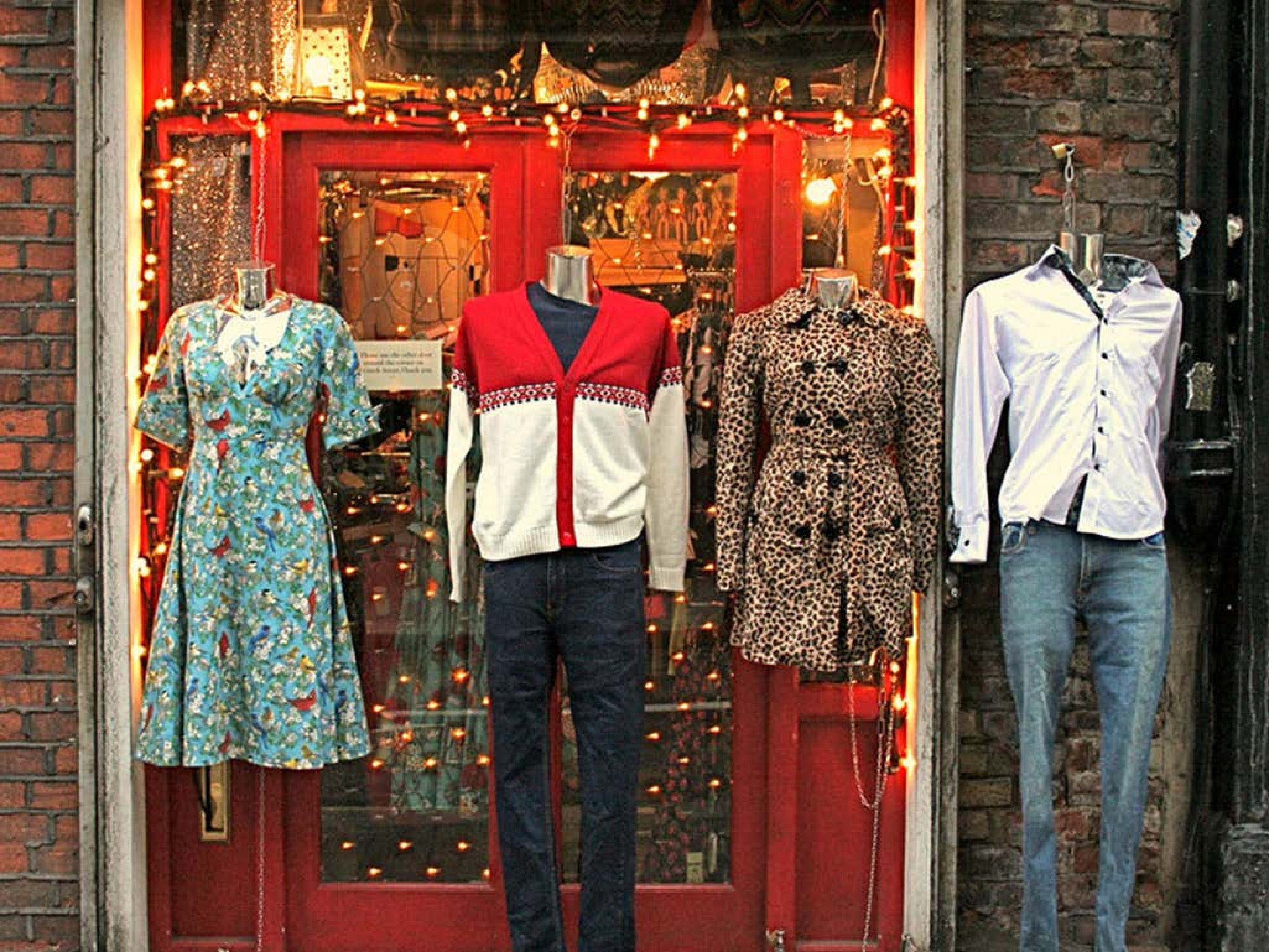

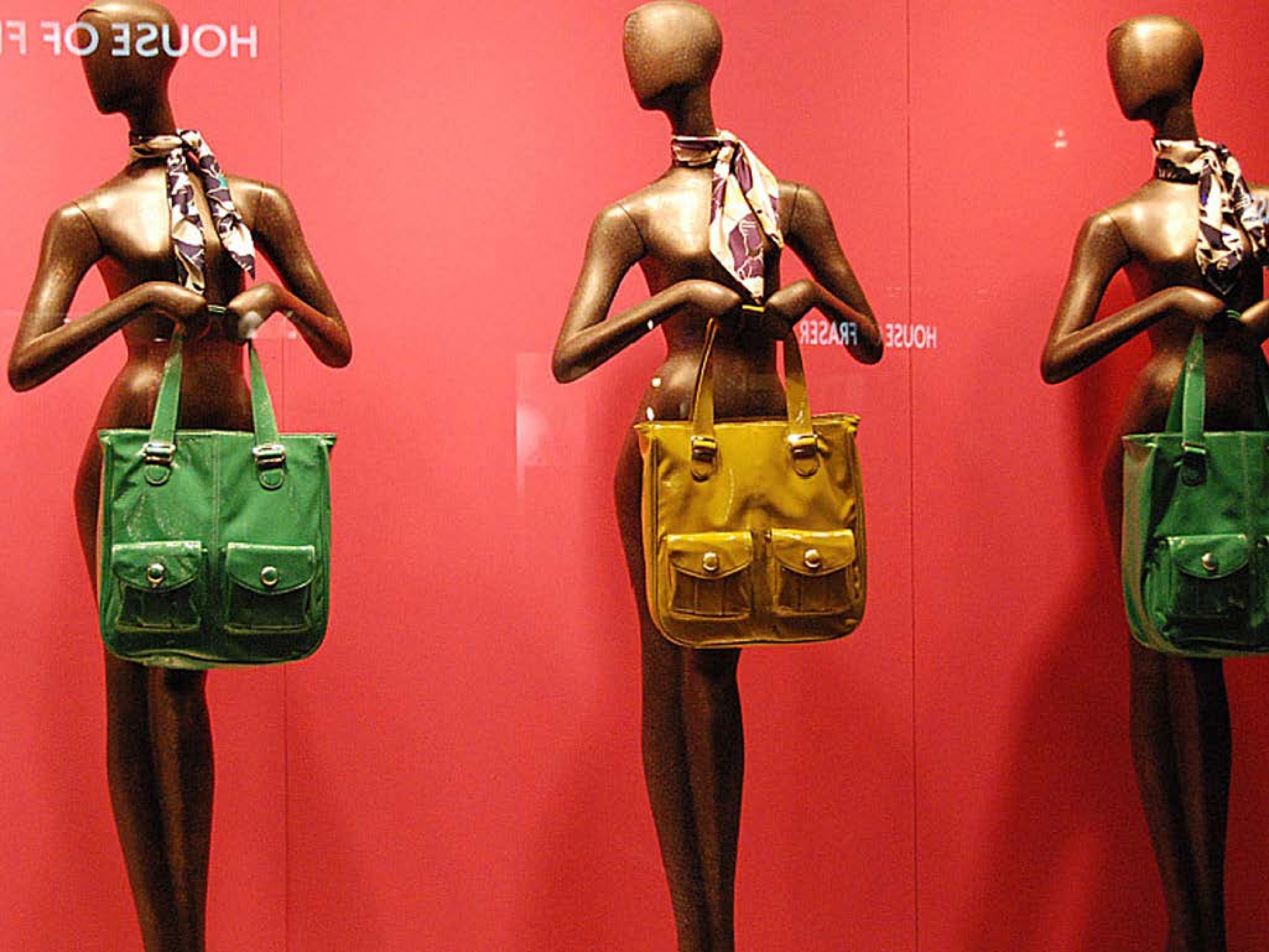
HOUSE OF
HOUSE
FRASER

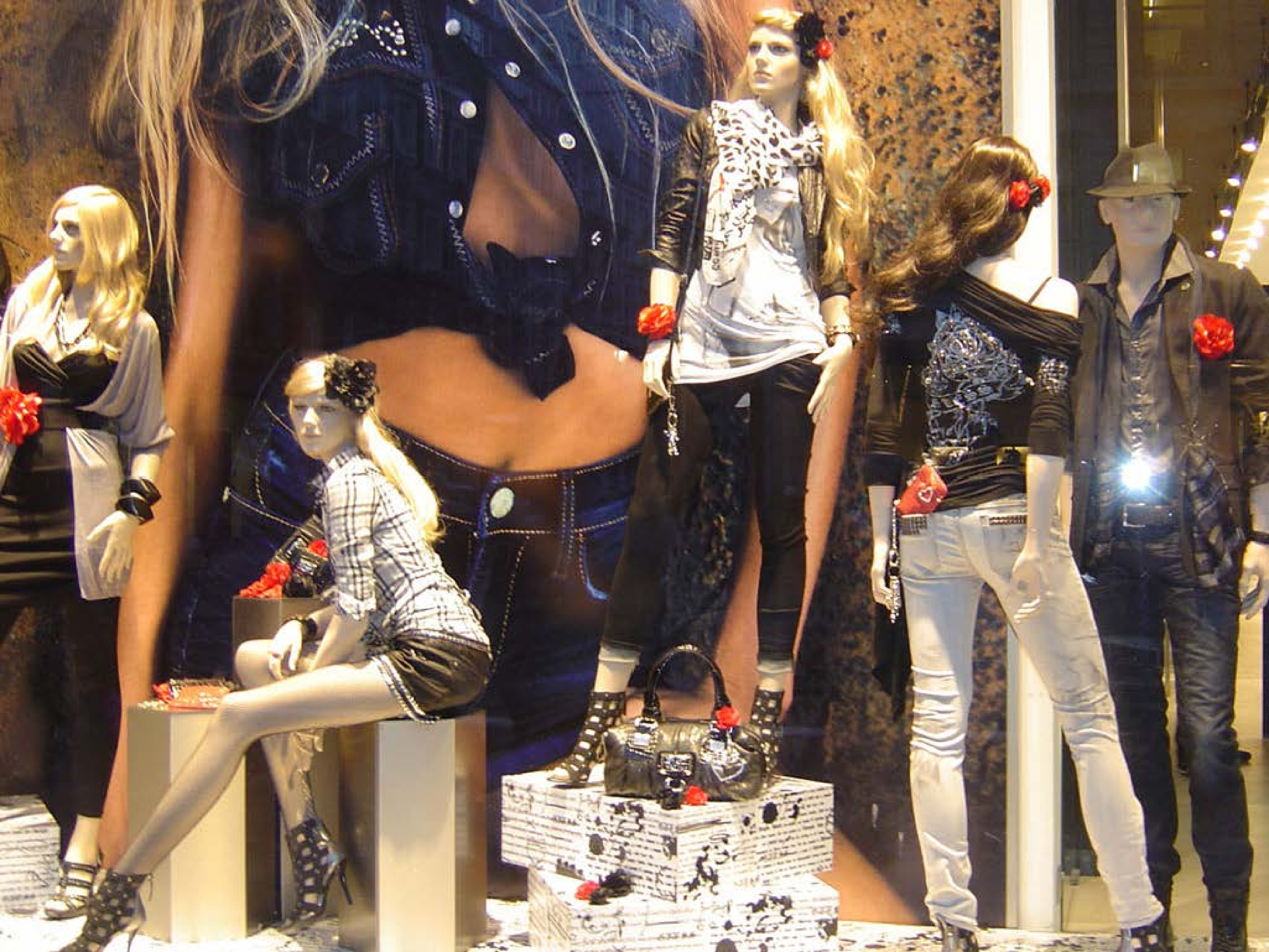

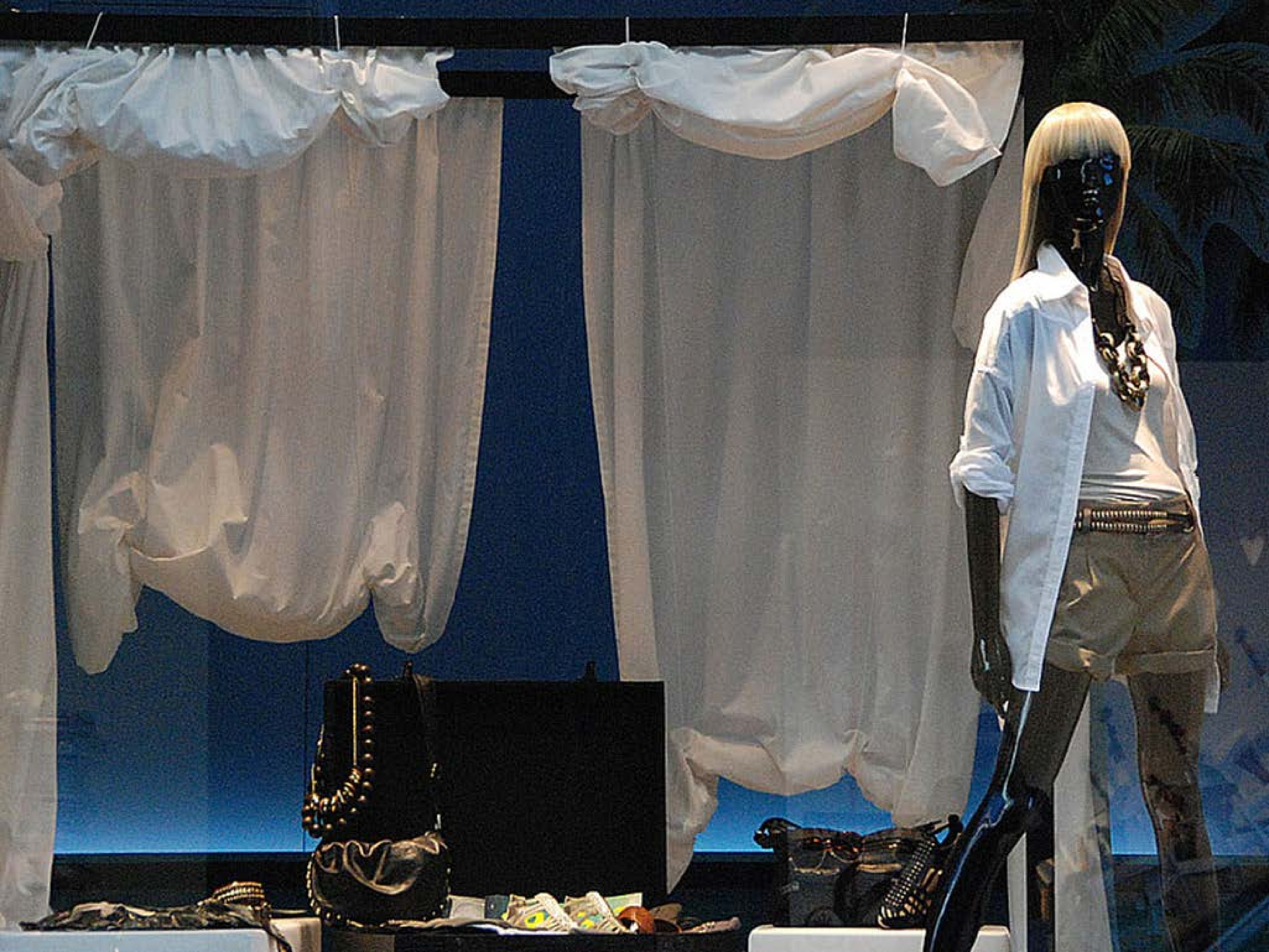

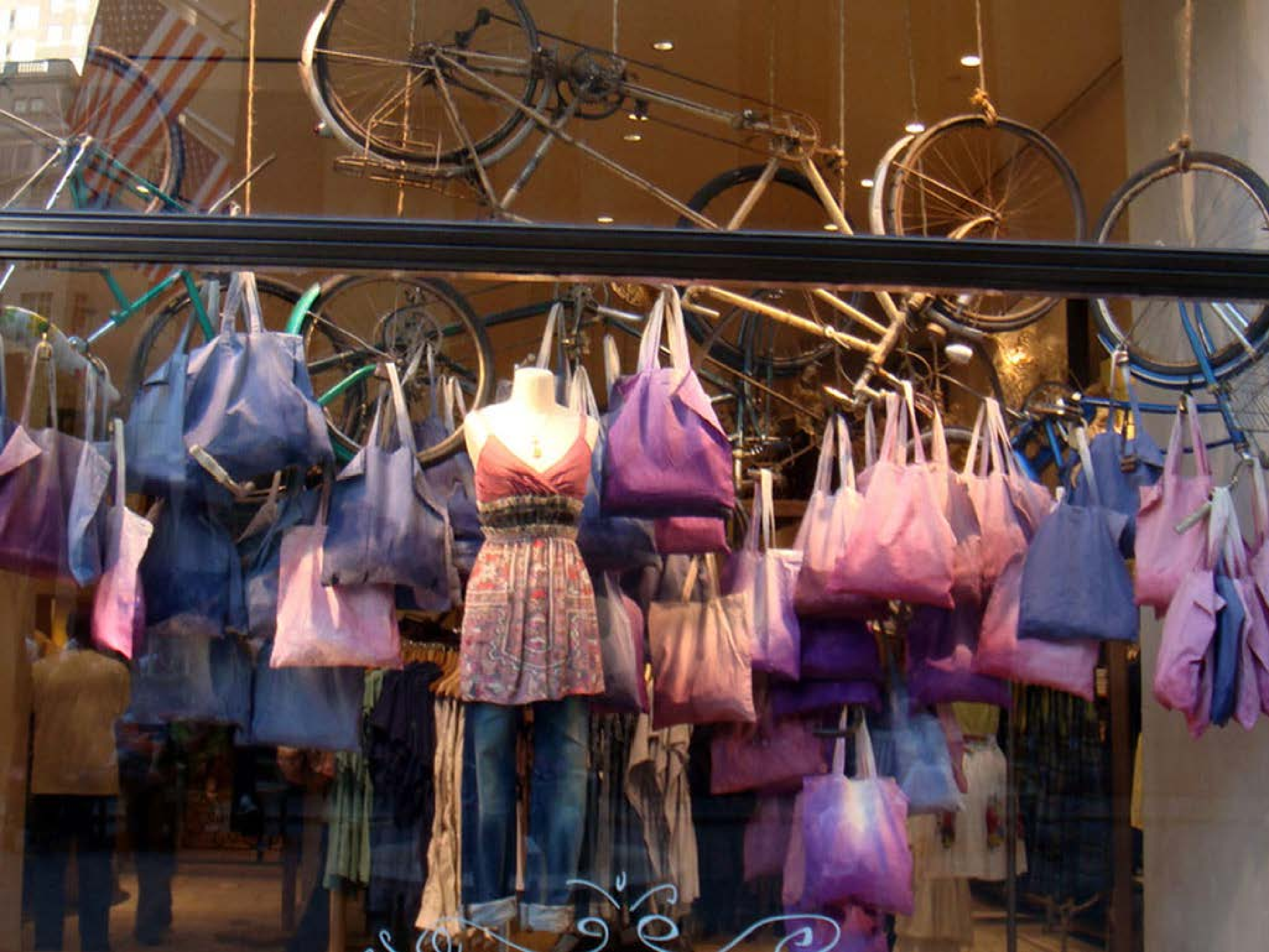

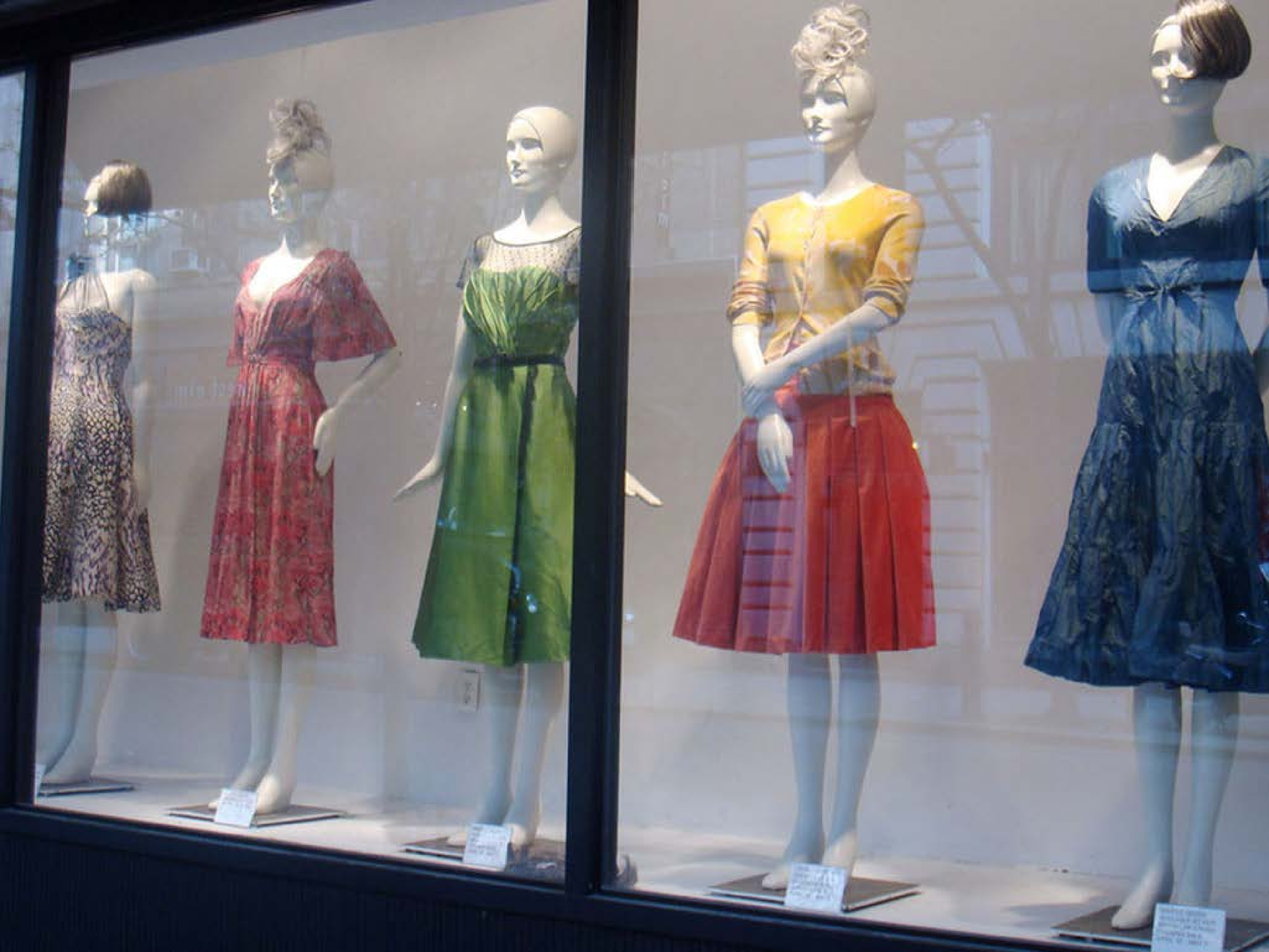

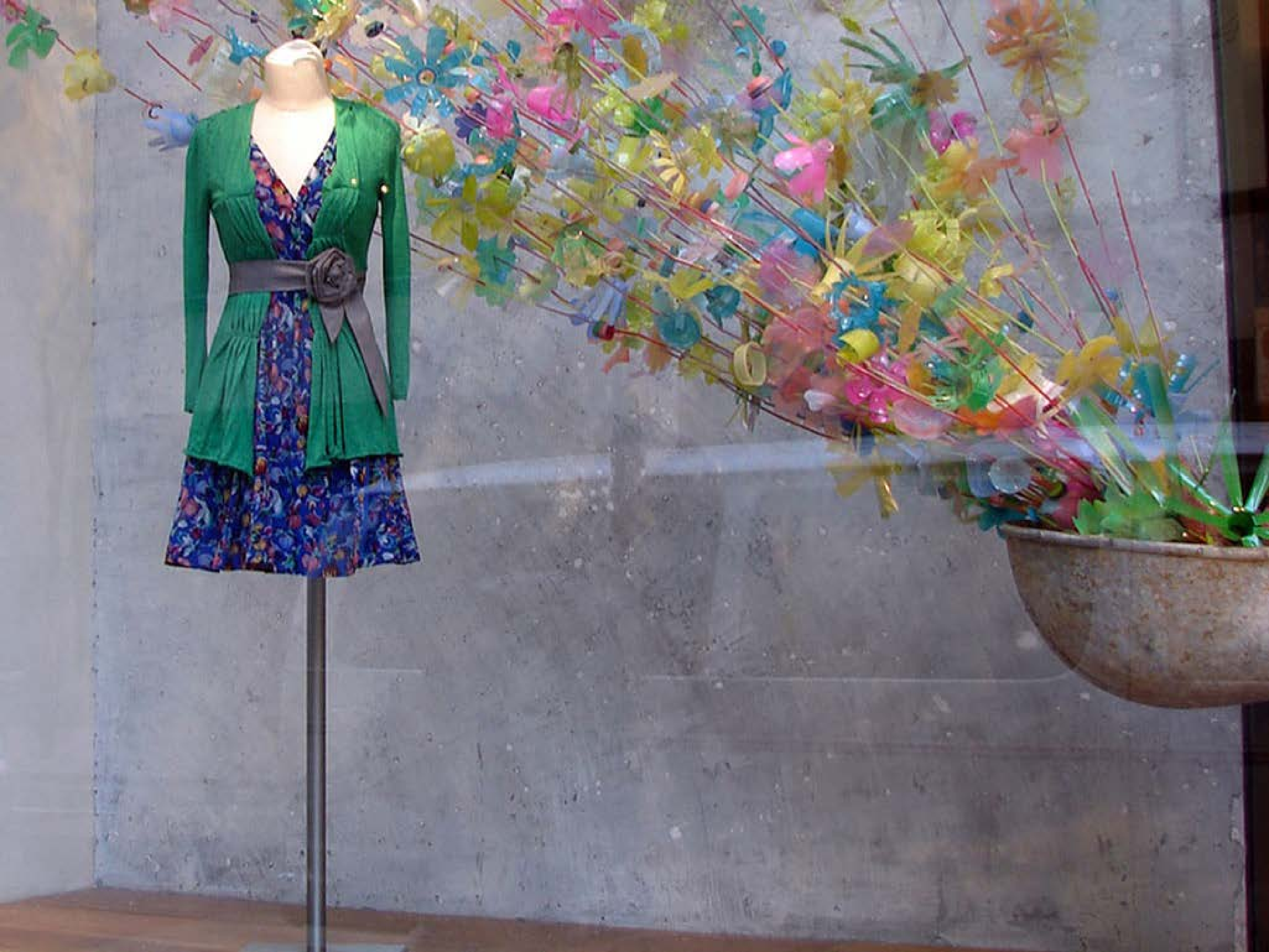

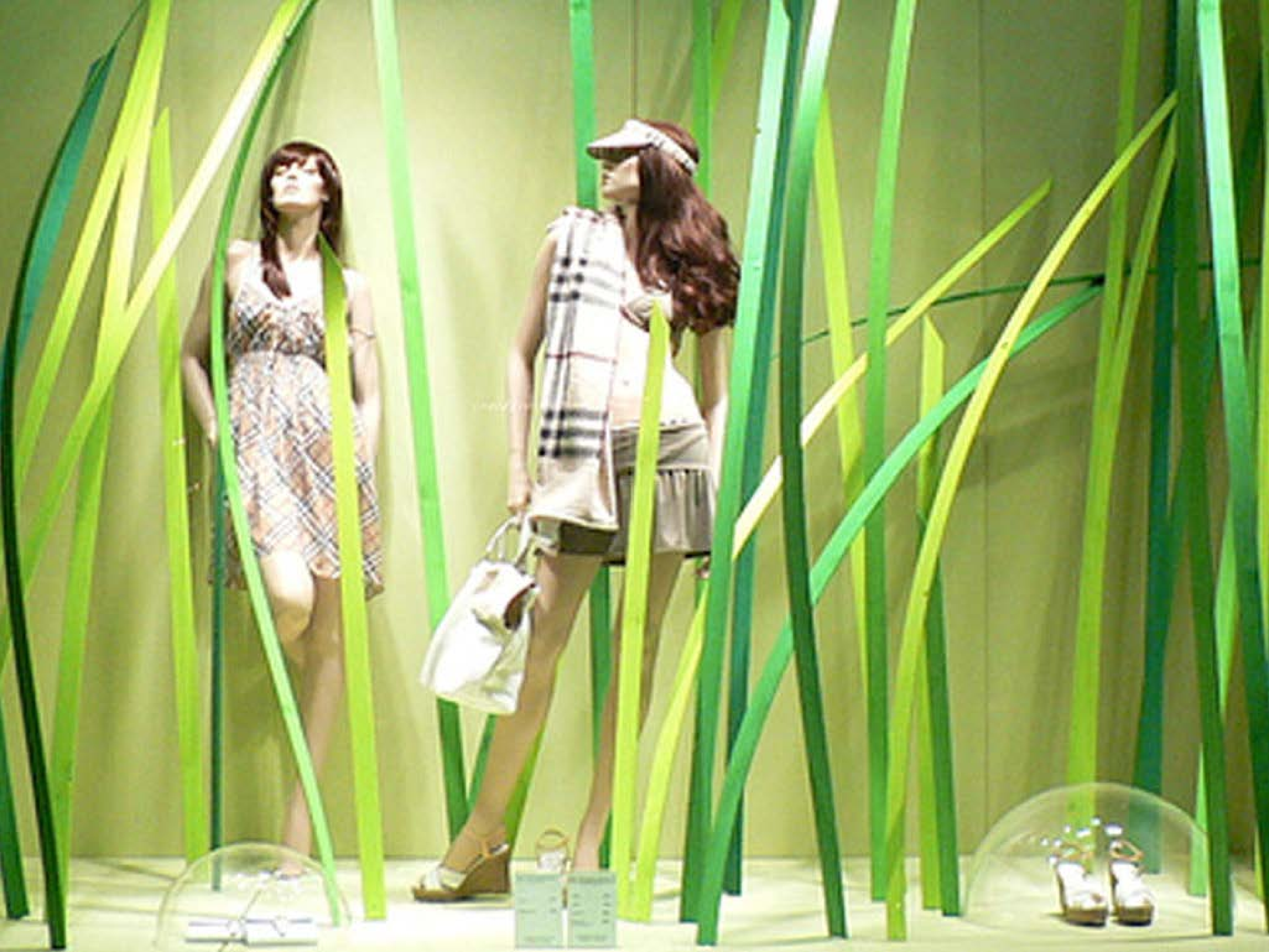

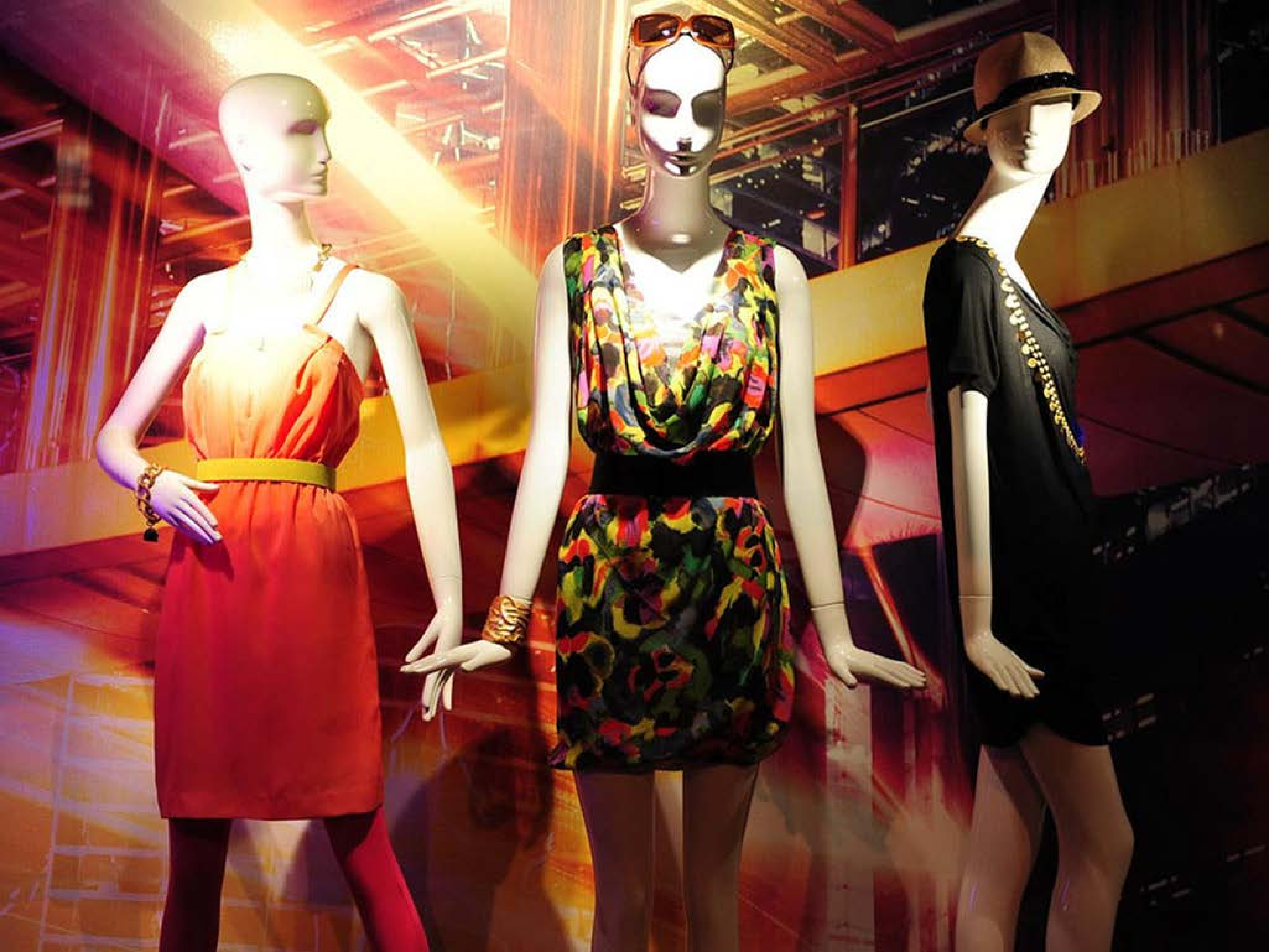

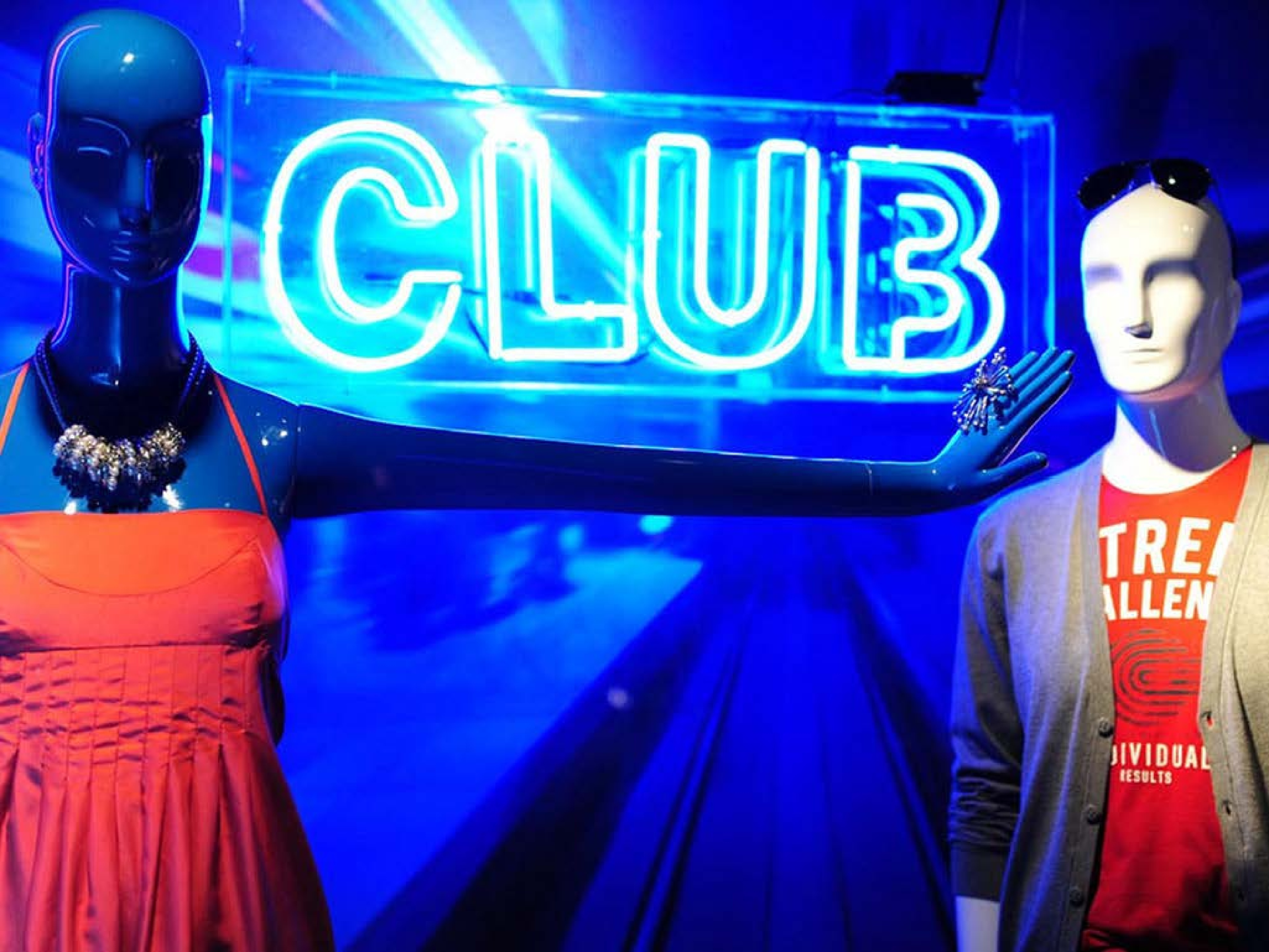
CLUB
RESULTS

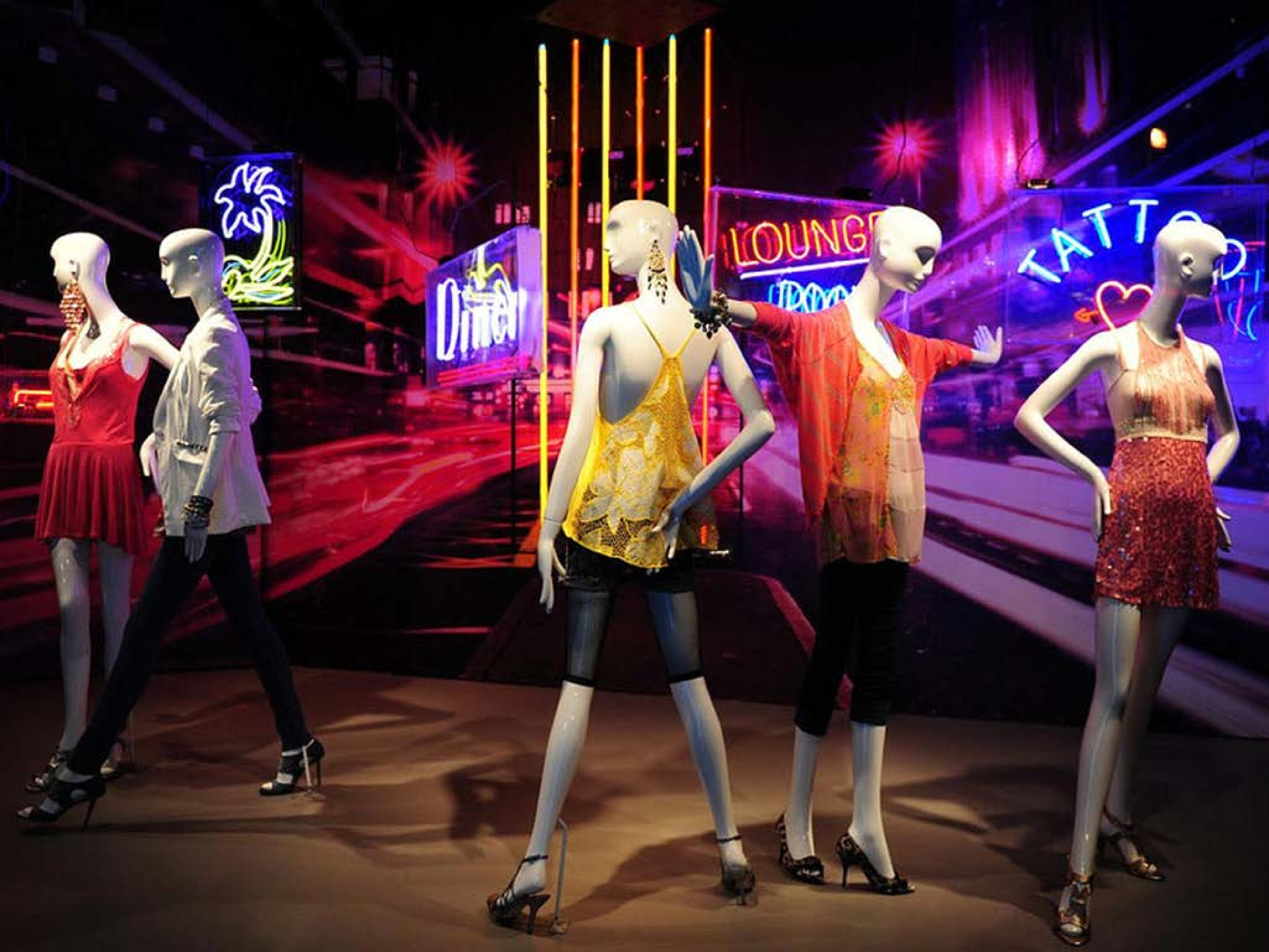
Diner
TATTO

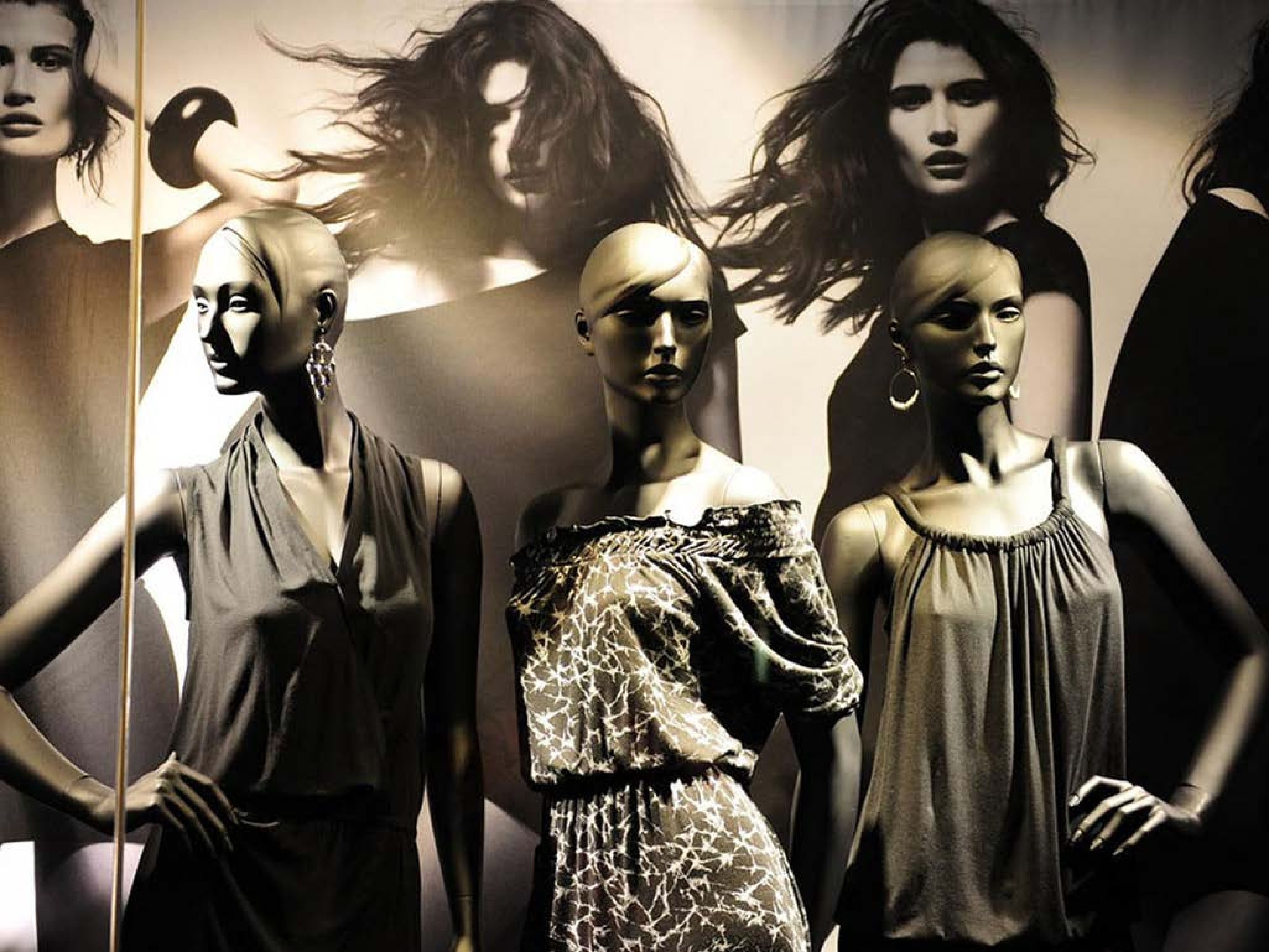

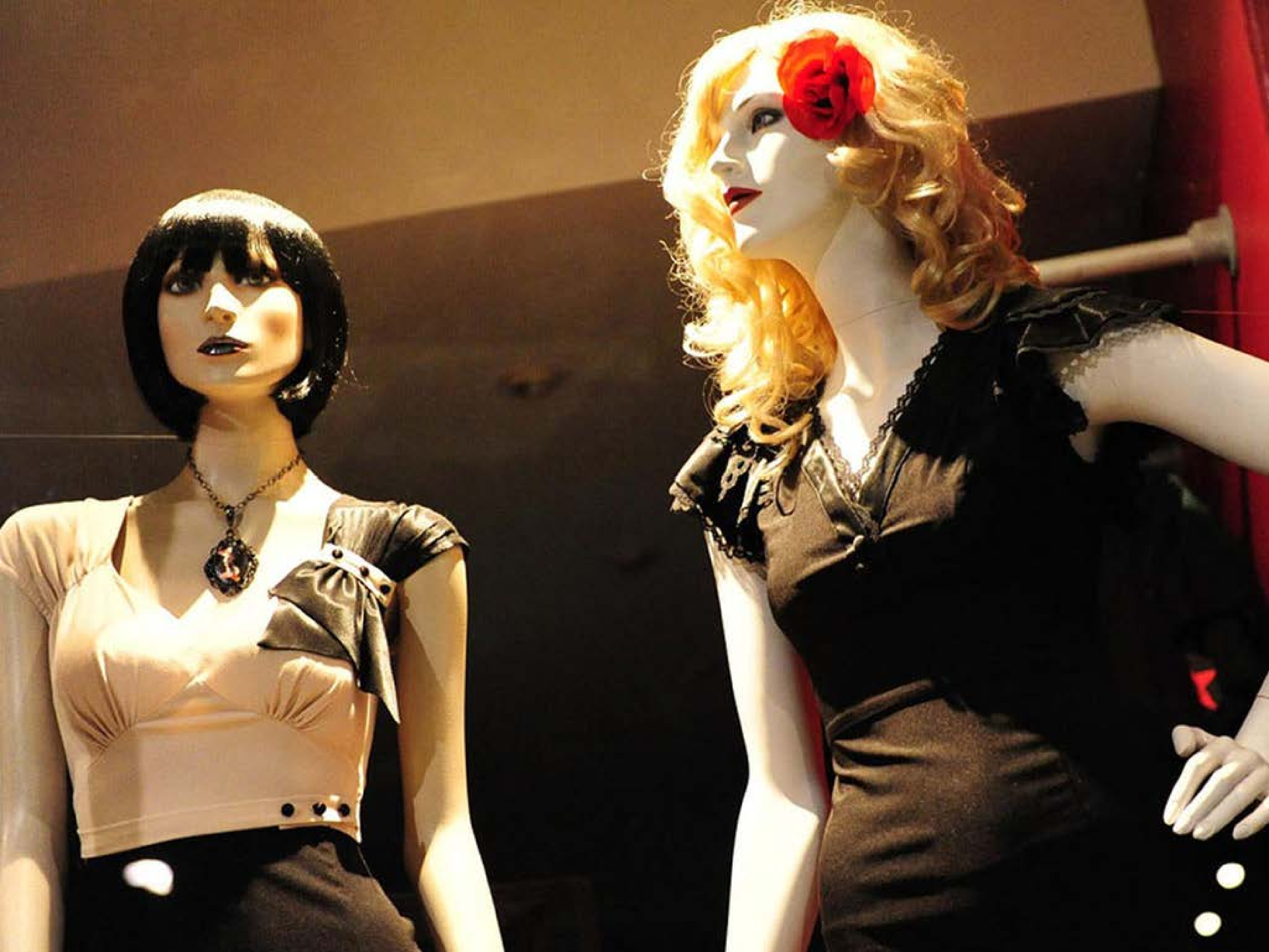

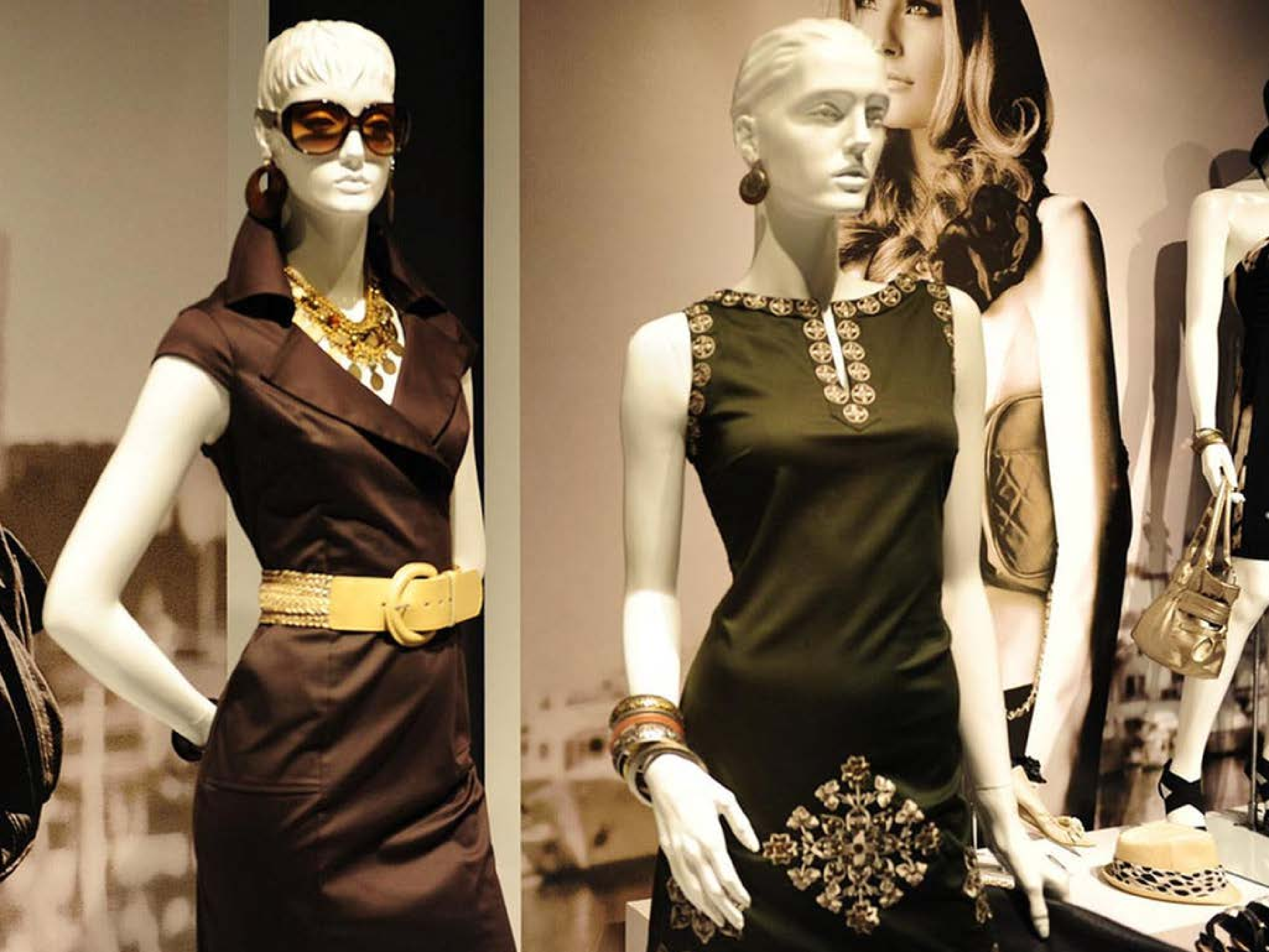

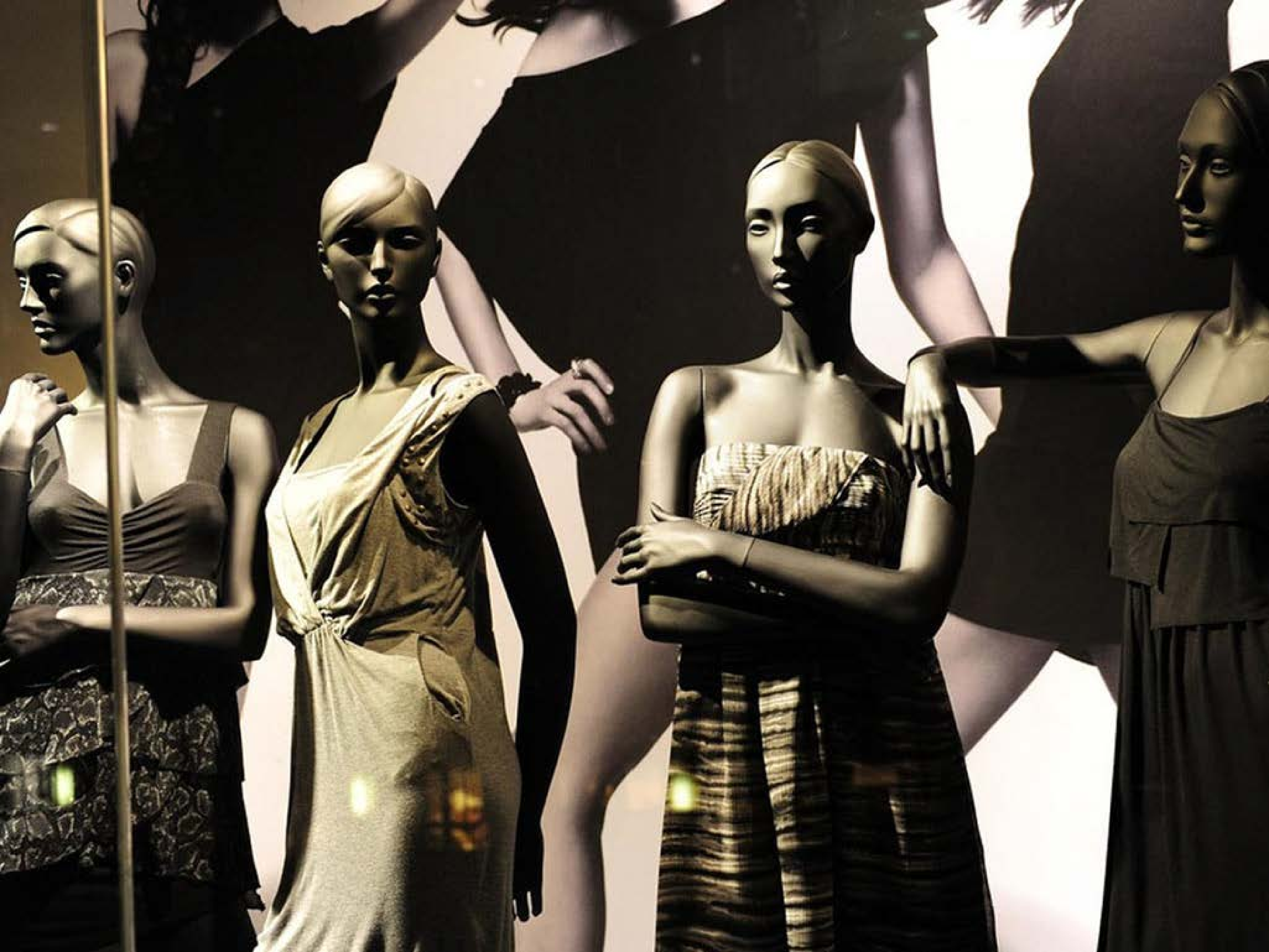

Supplement: Supplementary file 2 [file Data_Sheet_2.PDF]
